# Mechanism of drug-pairs Astragalus Mongholicus–Largehead Atractylodes on treating knee osteoarthritis investigated by GEO gene chip with network pharmacology and molecular docking

**DOI:** 10.1097/MD.0000000000038699

**Published:** 2024-07-05

**Authors:** Hui Wang, Xinyou Zhao, Zixuan Wu

**Affiliations:** a Jinan Third People’s Hospital, Affiliated Jinan Third People’s Hospital of Jining Medical University, Jining, Shandong, China; b Yanzhou People’s Hospital, Jining Medical University, Jining, Shandong, China; c Hunan University of Traditional Chinese Medicine, Changsha, Hunan Province, China.

**Keywords:** Astragalus Mongholicus (AM, *bái zhú*), GEO difference analysis, *huáng qí*), Knee osteoarthritis, Largehead Atractylodes (LA), molecular docking, network pharmacology

## Abstract

Investigations into the therapeutic potential of Astragalus Mongholicus (AM, huáng qí) and Largehead Atractylodes (LA, bái zhú) reveal significant efficacy in mitigating the onset and progression of knee osteoarthritis (KOA), albeit with an elusive mechanistic understanding. This study delineates the primary bioactive constituents and their molecular targets within the AM–LA synergy by harnessing the comprehensive Traditional Chinese Medicine (TCM) network databases, including TCMSP, TCMID, and ETCM. Furthermore, an analysis of 3 gene expression datasets, sourced from the gene expression omnibus database, facilitated the identification of differential genes associated with KOA. Integrating these findings with data from 5 predominant databases yielded a refined list of KOA-associated targets, which were subsequently aligned with the gene signatures corresponding to AM and LA treatment. Through this alignment, specific molecular targets pertinent to the AM–LA therapeutic axis were elucidated. The construction of a protein-protein interaction network, leveraging the shared genetic markers between KOA pathology and AM–LA intervention, enabled the identification of pivotal molecular targets via the topological analysis facilitated by CytoNCA plugins. Subsequent GO and KEGG enrichment analyses fostered the development of a holistic herbal-ingredient-target network and a core target-signal pathway network. Molecular docking techniques were employed to validate the interaction between 5 central molecular targets and their corresponding active compounds within the AM–LA complex. Our findings suggest that the AM–LA combination modulates key biological processes, including cellular activity, reactive oxygen species modification, metabolic regulation, and the activation of systemic immunity. By either augmenting or attenuating crucial signaling pathways, such as MAPK, calcium, and PI3K/AKT pathways, the AM–LA dyad orchestrates a comprehensive regulatory effect on immune-inflammatory responses, cellular proliferation, differentiation, apoptosis, and antioxidant defenses, offering a novel therapeutic avenue for KOA management. This study, underpinned by gene expression omnibus gene chip analyses and network pharmacology, advances our understanding of the molecular underpinnings governing the inhibitory effects of AM and LA on KOA progression, laying the groundwork for future explorations into the active components and mechanistic pathways of TCM in KOA treatment.

## 1. Introduction

Knee osteoarthritis (KOA) manifests as a chronic arthropathy distinguished by the degeneration of knee cartilage, remodeling of subchondral bone, and synovial inflammation, culminating in diminished joint mobility, pain, and stiffness.^[1]^ Contemporary medical discourse posits that KOA’s etiology may intertwine with genetic, biological, and biomechanical vectors, significantly impairing human quality of life.^[2]^ Predominantly afflicting the middle-aged and elderly demographic, the incidence of KOA has surged in parallel with the aging population, elongated life expectancy, and escalating body mass index trends.^[3]^ Conventional Western medicinal strategies predominantly pivot around nonsteroidal anti-inflammatory drugs and joint replacement surgery.^[4]^ Despite their symptomatic palliation, these interventions often precipitate adverse gastrointestinal and cardiovascular events, underscoring a pressing need for alternative therapeutics.^[5]^ In this context, Traditional Chinese Medicine (TCM) has garnered escalating recognition for its unique therapeutic benefits.^[6–8]^ Esteemed for millennia in the annals of Chinese medical practice, TCM’s holistic approach, characterized by its multicomponent, multi-target, multi-pathway, and synergistic properties, presents a promising avenue for the efficacious prevention and management of KOA. The rich tapestry of TCM embodies a profound potential for integrative health strategies, meriting profound exploration and application in mitigating the global burden of KOA.^[9]^

**Figure 1.**
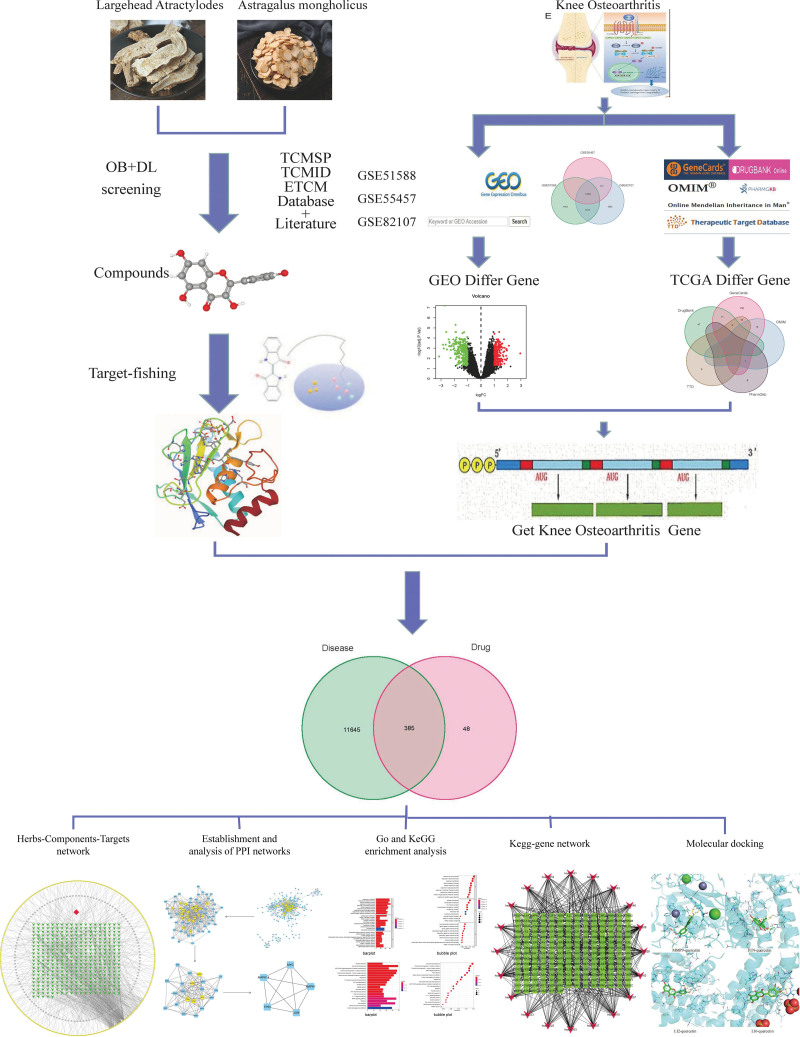
Framework based on an integration strategy of network pharmacology.

Investigating the efficacy of TCM components in mitigating joint degeneration presents a burgeoning frontier in TCM research. Numerous studies have delved into the mechanisms underlying KOA by examining how TCM’s active constituents modulate the immune-inflammatory cascade, encompassing the inhibition of chondrocyte apoptosis, amelioration of the cellular microenvironment, and regulation of signaling pathways. Central to TCM’s diagnostic framework is the notion that spleen deficiency and dampness contribute significantly to KOA’s etiology and progression.^[10]^ Within this paradigm, Astragalus Mongholicus (AM) is reputed for vitalizing Qi, while Largehead Atractylodes (LA) is celebrated for its spleen-tonifying and dampness-resolving virtues. Notably, Shenling Baizhu Powder has been heralded as a bespoke formulation for KOA treatment, yet the clinical exploration of AM and LA’s synergistic potential remains scant.^[11,12]^ Emerging evidence underscores AM and LA’s anti-inflammatory prowess, albeit investigations into their collective role and mechanistic contributions towards decelerating KOA progression are sparse. By harnessing the vast repositories of TCM network databases, this study aspires to elucidate the principal active components and their target actions within TCM. Leveraging differential gene expressions from the gene expression omnibus (GEO) gene chip and intersecting drug-disease genes, we aim to construct a comprehensive action network. This endeavor will facilitate the establishment of a herbal medicine-component-target network and a core target-signal pathway network. Employing molecular docking technology to validate key compound-target interactions offers a novel vista into the drug treatment mechanisms of KOA. As depicted in Figure 1, this methodological framework promises to crystallize our understanding of TCM’s therapeutic mechanisms, thereby enriching the reservoir of knowledge for foundational research and clinical praxis in the realm of KOA treatment.

## 2. Materials and methods

### 2.1. Construction of a database of in main active ingredients

#### 2.1.1. Development of an active ingredient database

The inaugural phase of our investigation entailed the scrupulous assembly of a comprehensive database cataloguing the key bioactive constituents of AM and LA. This crucial step was achieved by harnessing the capabilities of the TCMSP, an indispensable tool for the identification of AM and LA’s active molecules. The selection of these molecules was rigorously based on established ADME criteria, adhering to optimal toxicological profiles with an oral bioavailability (OB) of ≥30% and a DL score of ≥0.18,^[13]^ as highlighted in the latest pharmacological discourse. For compounds not meeting these criteria or absent from the TCMSP, we expanded our search to include the TCMID, ETCM, and a comprehensive review of the current literature on the bioactive elements of AM and LA. These compounds were further authenticated by referencing their molecular structures in the PubChem and PharmGKB databases. To guarantee the consistency of our dataset, the protein identifiers for the AM and LA compounds were standardized using the UniProt database, facilitated by Perl scripting. This meticulous approach not only solidified the foundation of our database but was pivotal in setting the stage for the intricate analyses that followed, ultimately enhancing our understanding of the therapeutic efficacy inherent in AM and LA.

#### 2.2. Establishment of a KOA-related targets database

Initial steps involved sourcing messenger RNA differential expression microdata for KOA versus normal groups from the GEO database, specifically series GSE51588, GSE55457, and GSE82107. These datasets were integrated via Perl software, followed by the utilization of Sva and Limma packages in R (version 4.1.0) for a consolidated analysis and batch normalization of data. Genes exhibiting an adjusted *P*-value < .05 and a log2(fold change) > 1 or log2(fold change) < −1 were classified as significantly differentially expressed, denoting KOA-related targets. VENN diagrams, depicting the overlap of datasets from the 3 chips, were generated using the VENN package in R. Additional target identification employed the keyword “KOA” in databases such as GeneCards, PharmGKB, OMIM, TTD, and DrugBank. The compilation of targets from these 5 databases, integrated via R software, alongside GEO differential analysis, facilitated the creation of a comprehensive KOA target database, refined through the VENN package in R to exclude redundant disease targets.

### *2.*3*. Protein–Protein Interaction (PPI) network formation*

Building on the aforementioned analyses, the intersection of core active ingredient targets from AM and LA with KOA disease targets yielded a consolidated compound target set. This intersection was visualized using a VENN diagram via the VENN tool. To elucidate the functional relationships among differentially expressed genes (DEGs), a protein–protein interaction (PPI) network was constructed through the STRING database, specifying “Homo sapiens” as the organism and setting the confidence level threshold at 0.900, to analyze the drug-disease gene interactions.

### 2.4. Synthesis of an “H-C-T” network

Leveraging the PPI network previously delineated, an intricate “Herbs-Components-Targets” (H-C-T) network specific to the treatment of KOA by AM and LA was architectured using the Cytoscape 3.7.2 software. The construction of this network was informed by critical topological features, with the CytoNCA plugin facilitating the selection of 3 paramount centrality measures to identify the pivotal targets within the AM–LA therapeutic axis: degree centrality (DC), cellular components (CC), and betweenness centrality (BC). The quantitative thresholds for these centrality metrics were established based on an extensive review of the literature, with nodes exhibiting a DC twice the median value being earmarked for inclusion. Conversely, the median values for BC and closeness centrality were employed as benchmarks to refine the selection of core targets. This methodology underscored the nodes’ topological significance within the network, reflecting their pivotal roles and influence within the holistic framework and ensuring the precision in identifying the core targets integral to the therapeutic efficacy of AM and LA against KOA.^[14]^

### 2.5. Functional annotation via GO and KEGG pathway analyses

To elucidate the biological functions and delineate the signaling pathways intertwined with the differential expression landscape, comprehensive gene ontology (GO) and Kyoto Encyclopedia of Genes and Genomes (KEGG) analyses were conducted. Utilizing the robust R statistical environment, we embarked on an exploration of how fluctuations in HLF expression influence biological processes (BPs), molecular functions (MFs), and CC, thereby providing a deeper understanding of the molecular mechanisms underpinning these variations.

### 2.6. Validation of active components and target interactions through molecular docking

In our study, molecular docking was employed to validate the interaction between the principal active components and the core targets identified within the PPI network topology. The structural configurations of these core targets were meticulously sourced from the UniProt database, with selection criteria prioritizing crystal structures elucidated via X-ray crystallography for their superior resolution. These configurations were further corroborated by accessing the RCSB PDB database to obtain their precise crystallographic structures. Subsequent to acquiring the 2-dimensional molecular structures of 6 pivotal active components from the PubChem database, these structures underwent optimization using the Chem3D software to ensure their readiness for docking simulations. The SYBYL 2.0 software suite played a critical role in the assessment of the binding affinity and activity between the active components and their respective targets, setting a threshold TotalScore >3 as a criterion for advancing to detailed docking analysis. For the docking procedure, the crystal structures were prepared using PyMOL 2.4 software, which involved steps such as dehydration, hydrogenation, and ligand separation, thereby optimizing them for docking. The AutoDockTools 1.5.6 package facilitated the construction of precise docking grids for each target, ensuring the specificity of the interaction analyses. The docking simulations were executed using the Vina 1.1.2 software, with a focus on identifying molecules that exhibited the lowest binding energy, indicative of a stable and significant interaction. These molecules were then compared against the original ligands to assess the fidelity of binding and to analyze the nature of intermolecular interactions, including hydrophobic interactions, cation-π, anion-π, π–π stacking, and hydrogen bonding.

## 3. Results

### 3.1. Prediction and analysis of targets for AM and LA

Employing the ADME criteria with an OB of≥30% and a DL score of ≥0.18, we identified 428 core active components for AM and 20 for LA, as cataloged in the TCMSP database (Appendix Tables 1 and 2, Supplemental Digital Content, http://links.lww.com/MD/N42, http://links.lww.com/MD/N43). An additional 24 active ingredients for AM and 6 for LA were discerned from the TCMID database, with 2 further LA ingredients derived from relevant literature. The SwissTargetPrediction and STITCH databases contributed to identifying 166 active components for AM and 56 for LA (Appendix Table 3, Supplemental Digital Content, http://links.lww.com/MD/N44), while the ETCM database yielded 30 active components for AM and 9 for LA (Appendix Table 4, Supplemental Digital Content, http://links.lww.com/MD/N45). Despite the absence of explicit OB and DL data for components sourced from the TCMID and ETCM databases and related literature, their inclusion was deemed pertinent. Ultimately, 9 core active components were distinguished (refer to Table 1 or Appendix Table 5, Supplemental Digital Content, http://links.lww.com/MD/N46). Canonical SMILES numbers of these core active components facilitated the identification of 670 targets through target fishing, integrating data from the TCMSP, TCMID, ETCM, and STITCH databases (Appendix Tables 2–4, Supplemental Digital Content http://links.lww.com/MD/N43, http://links.lww.com/MD/N44, http://links.lww.com/MD/N45).

**Table 1:** Core components of AM–LA.

PubChem ID	Name	OB	DL	Source
5281654	Isorhamnetin	49.60	0.31	TCMSP
15689652	7-O-methylisomucronulatol	74.69	0.30	TCMSP
5280378	Formononetin	69.67	0.21	TCMSP
442811	Mucronulatol	NA	NA	ETCM
5280448	Calycosin	47.75	0.24	TCMSP
5280863	Kaempferol	41.88	0.24	TCMSP
5280343	Quercetin	46.43	0.28	TCMSP
3917	(*S*)-5,7-Dihydroxy-2-phenylchroman-4-one, pinocembrin	NA	NA	TCMID
2723872	Fructose	NA	NA	ETCM

AM = Astragalus Mongholicus, LA = Largehead Atractylodes, OB = oral bioavailability.

### 3.2. Search, identification, and analysis of DEGs

**Figure 2.**
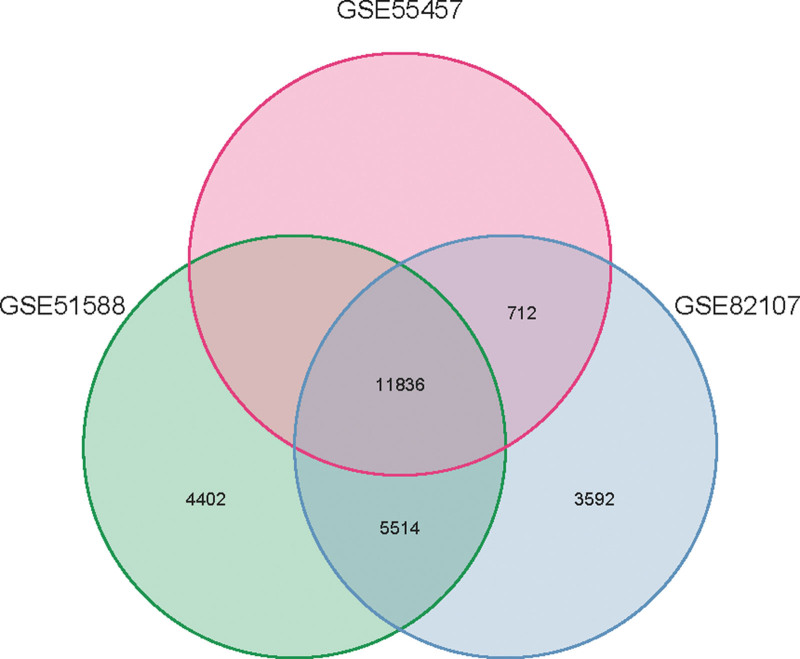
Combined genes VNN maps of 3 GEO chips. GEO = gene expression omnibus.

**Figure 3.**
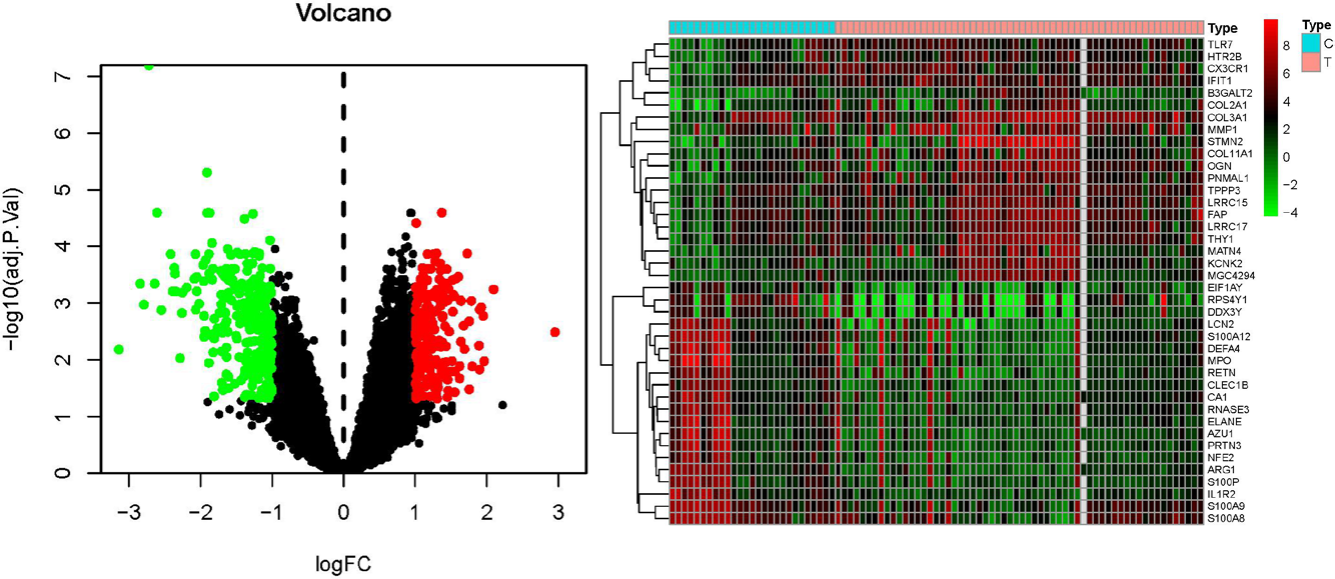
Differential genes volcano map and heat map jointly analyzed by 3 GEO chips. mRNA from normal group and KOA group. GEO = gene expression omnibus, KOA = knee osteoarthritis, mRNA = messenger RNA.

Data comprising 27 healthy samples and 60 KOA samples were acquired from the GEO database, distributed across series GSE51588 (40/10), GSE55457 (10/10), and GSE82107 (10/7). Utilizing R 4.1.0, a VENN diagram was constructed (Fig. 2), isolating 11,836 genes. Setting a threshold of *P* < .05 and an absolute log2 fold change (FC) > 1, 488 DEGs were elucidated using R, subsequently visualized through both volcano and heat maps (Fig. 3). Among these, 215 (45.05%) exhibited upregulation, whereas 273 (54.95%) were downregulated. A subset of 40 genes, potentially integral to the pathogenesis of KOA, was highlighted for further investigation (Appendix Table 6, Supplemental Digital Content, http://links.lww.com/MD/N47).

### 3.3. Compilation of KOA-related targets

**Figure 4.**
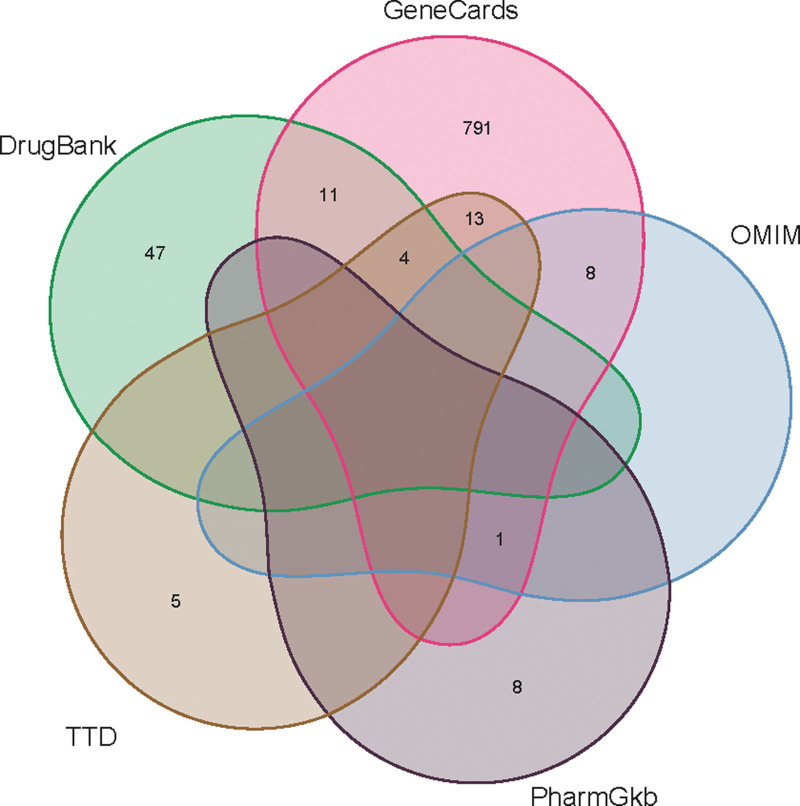
Combined genes VNN maps of 5 databases.

Target data was amassed from several databases: GeneCards (3114 targets), TTD (22), OMIM (9), PharmGKB (9), and DrugBank (80). Filtering based on a relevance score below 1 and merging results from these databases culminated in identifying 888 KOA-related targets. Integration of these targets with GEO-derived differential genes yielded a comprehensive set of 12,078 KOA-related gene targets (Fig. 4).

### 3.4. Analysis of the “H-C-T” network

**Figure 5.**
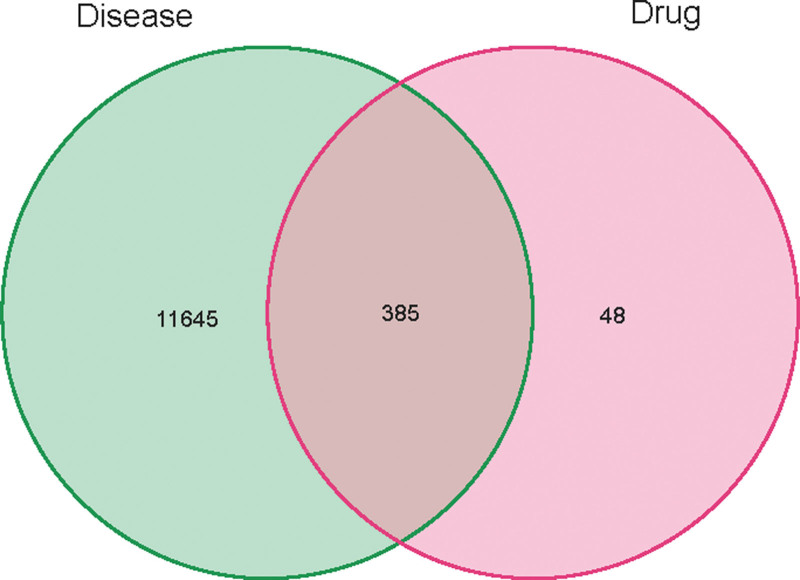
VENN diagram of the targets in KOA and AM–LA. AM = Astragalus Mongholicus, KOA = knee osteoarthritis, LA = Largehead Atractylodes.

**Figure 6.**
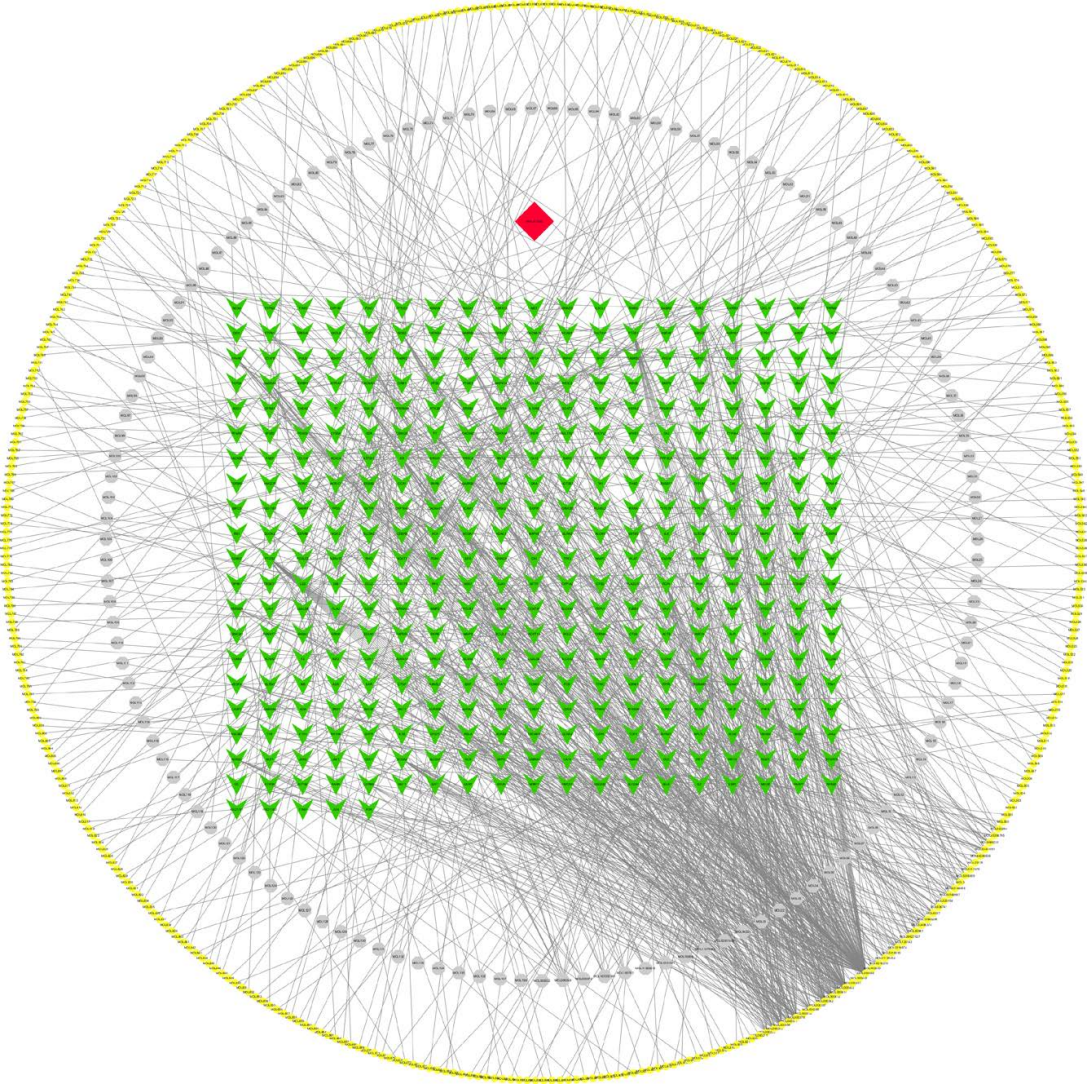
Herb-ingredients-targets (H-I-T) network. Yellow node represents AM, gray nodes represent targets of LA, red node represents multi-drug, green nodes represent core active compounds of AM–LA. AM = Astragalus Mongholicus, LA = Largehead Atractylodes.

**Figure 7.**
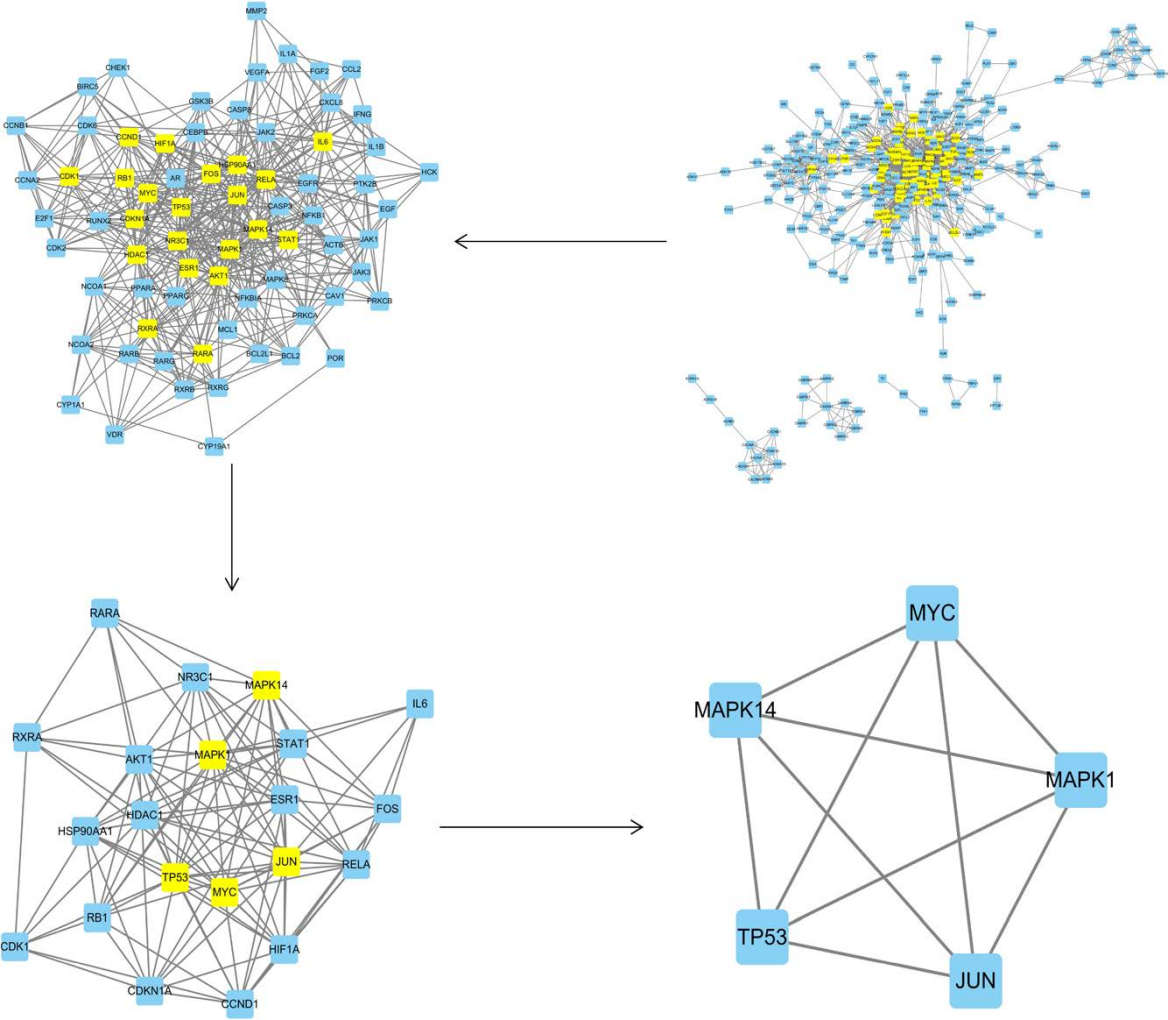
The process of topological screening for the PPI network. PPI = protein–protein interaction.

In this study, we meticulously matched the core active component targets of AM and LA drug pairs with disease targets of KOA, resulting in the identification of 296 unique AM and LA drug pairs and KOA compound targets (Fig. 5 and Appendix Table 7, Supplemental Digital Content, http://links.lww.com/MD/N48). This comprehensive set of compound targets was input into the STRING database for analysis, from which disconnected targets were excluded to construct a PPI network. Utilizing BisoGenet within the Cytoscape 3.7.2 framework revealed a network comprising 7806 nodes and 183,378 edges. The intricate “H-C-T” network specific to KOA was thus established, encapsulating 296 nodes and 1276 edges (Fig. 6; Appendix Tables 8 and 9, Supplemental Digital Content, http://links.lww.com/MD/N49, http://links.lww.com/MD/N50). Subsequent analysis, guided by network topology characteristics and facilitated by the CytoNCA plugin, enabled the distillation of 6 core targets from the network. This selection process was informed by the application of DC, BC, and CC, specifically targeting those with values thrice the median for DC and at the median for BC and CC. Through this rigorous screening, we identified MYC, TP53, MAPK1, MAPK14, and JUN as principal effector genes within the AM and LA combination treatment for KOA (Table 2 or Appendix Table 10, Supplemental Digital Content, http://links.lww.com/MD/N51). This analysis underscores the potential of MYC, TP53, MAPK1, MAPK14, and JUN as pivotal genes in the therapeutic landscape of AM and LA for KOA management. Their identification as “Key Targets” (Fig. 7) marks a significant milestone in our research, offering novel insights into the molecular mechanisms underpinning the efficacy of these TCM components in combating KOA.

**Table 2:** Information on 6 core targets.

SUID	Name	Protein name	Betweenness	Closeness	Degree
4140	TP53	Cellular tumor antigen p53	16.42787539	0.833333333	16
4403	MAPK1	Mitogen-activated protein kinase 1	25.1888422	0.8	15
4169	JUN	Transcription factor AP-1	16.39710975	0.8	15
4342	MYC	MYC proto-oncogene protein	11.55887446	0.769230769	14
4266	MAPK14	Mitogen-activated protein kinase 14	8.677849928	0.714285714	12

### 3.5. GO and KEGG enrichment analysis

**Figure 8.**
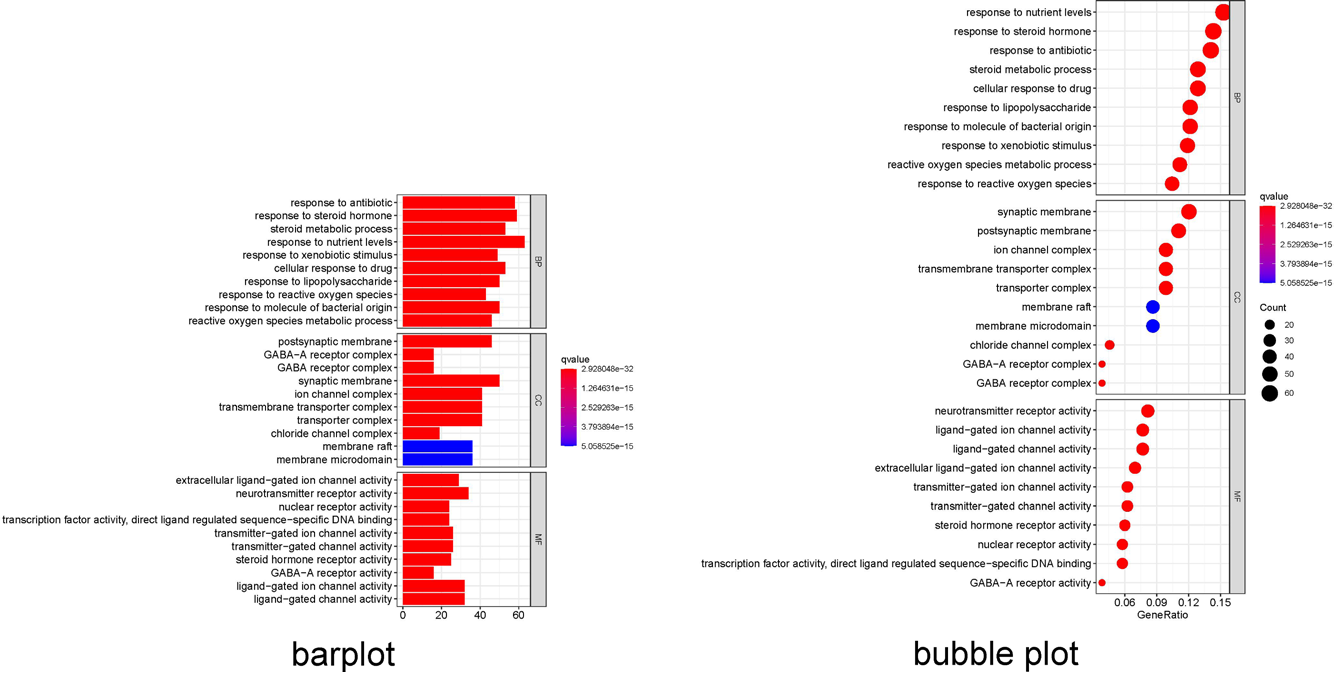
The GO enrichment analysis of core nodes. Including cellular components, molecular functions, and biological processes. GO = gene ontology.

**Figure 9.**
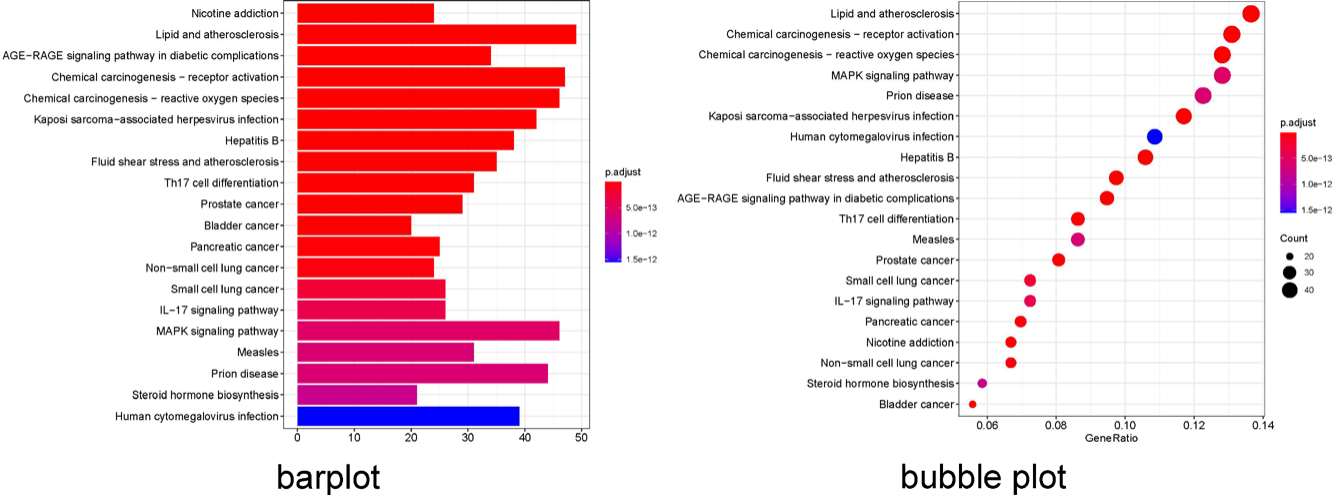
The KEGG enrichment analysis of core nodes. KEGG = Kyoto encyclopedia of genes and genomes.

**Figure 10.**
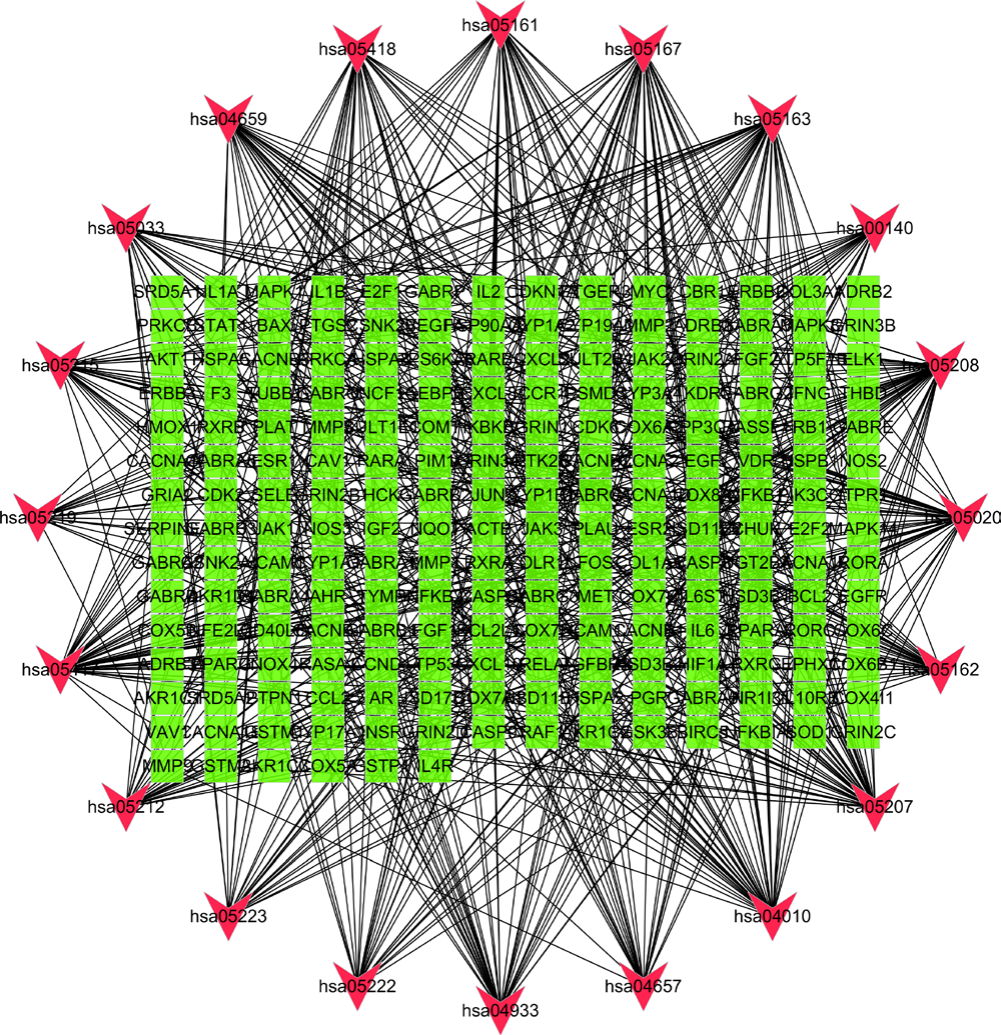
KEGG pathway enrichment analysis of the target-pathway network. The red nodes represent the pathways, whereas the green nodes represent the targets involved in these pathways. The edges represent the interactions between the targets and the pathways, and the node size is proportional to the degree of interaction. KEGG = Kyoto encyclopedia of genes and genomes.

GO enrichment analysis identified 2946 core targets involved in MF, CC, and BP. In terms of MF, drug-pair of AM–LA treatment of KOA mainly involves channel activity (GO: 0015267), passive transmembrane transporter activity (GO: 0022803), ion channel activity (GO: 0005216), substrate-specific channel activity (GO: 0022838), ion gated channel activity (GO: 0022839), gated channel activity (GO: 0022836). The BP mainly involves the response to nutrient levels (GO: 0031667), response to steroid hormone (GO: 0048545), response to antibiotic (GO: 0046677), regulation of membrane potential (GO: 0042391), response to oxidative stress (GO: 0006979). The cellular composition mainly involves the synaptic membrane (GO: 0097060), postsynaptic membrane (GO: 0045211), ion channel complex (GO: 0034702), transmembrane transporter complex (GO: 1902495), transporter complex (GO: 1990351; Appendix Tables 11–13, Supplemental Digital Content, http://links.lww.com/MD/N52, http://links.lww.com/MD/N53, http://links.lww.com/MD/N54). We selected the first 20 functional enrichment processes to draw a bubble diagram and a bar plot, as shown in Figure 8. In addition, we identified the main signaling pathways involved in the treatment of KOA by KEGG enrichment analysis, and screened the first 20 pathways related to KOA and significantly enriched (FDR < 0.05), including Pathways of neurodegeneration - multiple diseases (hsa05022), Alzheimer disease (hsa05010), Lipid and atherosclerosis (hsa05417), Neuroactive ligand–receptor interaction (hsa04080), MAPK signaling pathway (hsa04010), PI3K–AKT pathway (hsa04151) among others. The network diagram of “Core targets-Signal pathways” (Figs. 9 and 10) was constructed (Appendix Table 14, Supplemental Digital Content, http://links.lww.com/MD/N55).

### 3.6. Components-targets docking analysis

**Figure 11.**
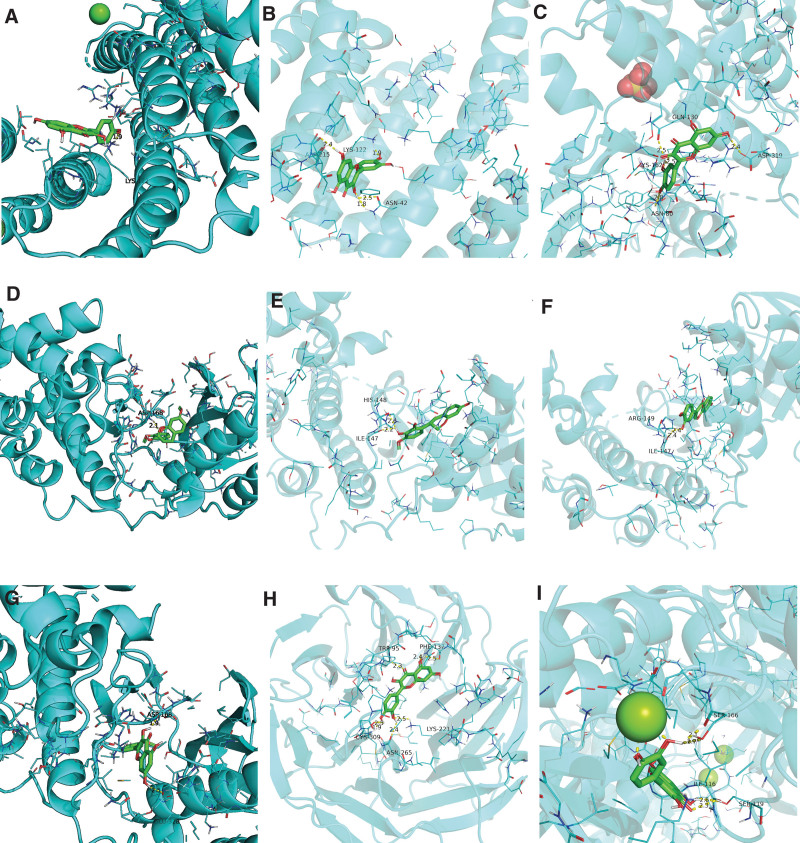
Schematic 3D representation that the molecular docking model, active sites, and binding distances. (A) JUN: kaempferol-6y3v; (B) JUN: quercetin-6y3v; (C) MAPK1: quercetin-4zzn; (D) MAPK14: 7-O-methylisomucronulatol-3k3i; (E) MAPK14: Mucronulatol-3k3i; (F) MAPK14: Calycosin-3k3i; (G) MAPK14: isorhamnetin-3k3i; (H) MYC: quercetin-4y7r; (I) TP53: quercetin-6wqx.

The 5 core targets were docked out of the PPI network topology. 6y3v (JUN, containing ligands), 4zzn (MAPK1, containing ligands), 3k3i (MAPK14, containing ligands), 4y7r (MYC, containing ligands), 6wqx (TP53, containing ligands) were obtained. Evaluation of the binding strength and activity of active components and targets by SYBYL2.0 software showed that the TotalScore of formononetin was 2.6282 less than 3. So molecular docking of formononetin was not was done. The affinity energies of the molecules were JUN: kaempferol-6y3v were −6.3 kcal/mol, JUN: quercetin-6y3v were −6.4 kcal/mol, MAPK1: quercetin-4zzn were -−7.5 kcal/mol, MAPK14: 7-O-methylisomucronulatol were −7.9 kcal/mol, MAPK14: Mucronulatol were −7.8 kcal/mol, MAPK14: Calycosin were −7.8 kcal/mol, MAPK14: isorhamnetin were −8.2 kcal/mol, MYC: quercetin-4y7r were −8.0 kcal/mol, TP53: quercetin-6wqx were −8.0 kcal/mol (Appendix Figures 15–21, Supplemental Digital Content, http://links.lww.com/MD/N56, http://links.lww.com/MD/N57, http://links.lww.com/MD/N58, http://links.lww.com/MD/N59, http://links.lww.com/MD/N60, http://links.lww.com/MD/N61, http://links.lww.com/MD/N62). Molecular docking results of JUN (kaempferol) indicated that there was 1 hydrogen bond with LYS-122 at the distance of 1.9 pm. Consequences of JUN (quercetin) indicated that there were 2 hydrogen bonds with ASN-42 at the distances of 2.5 and 1.8 pm. There was 1 hydrogen bond with ASP-215 and LYS-122 at the distances of 2.4 and 1.9 pm, respectively. The molecular docking result of MAPK1 (quercetin) showed that there was 1 hydrogen bond with GLN-130, LYS-162, ASN-80, and ASP-319 at the distances of 2.5, 2.5, 2.8, and 2.4 pm, respectively. The molecular docking consequence of MAPK14 (7-O-methylisomucronulatol) indicated that there was 1 hydrogen bond with ASP-168 at the distances of 2.1 pm. Results of MAPK14 (Mucronulatol) indicated that there was 1 hydrogen bond with HIS-148 and ILE-147 at the distances of 2.6 and 2.2 pm. Results of MAPK14 (Calycosin) indicated that there was 1 hydrogen bond with ARG-149 and ILE-147 at the distances of 2.4 and 2.4 pm. Results of MAPK14 (isorhamnetin) indicated that there was 1 hydrogen bond with ASP-168 and GLU-328 at the distances of 1.9 and 2.5 pm. The consequences of MYC (quercetin) molecular docking showed that there were 2 hydrogen bonds with PHE-137 at a distance of 2.4 and 2.5 pm. There were 2 hydrogen bonds with CYS-309 at a distance of 1.9 and 2.5 pm. There was 1 hydrogen bond with TRP-95, ASN-265, and LYS-221 at the distances of 2.3, 2.4, and 2.5 pm. Results of TP53 (quercetin) molecular docking showed that there were 2 hydrogen bonds with SER-166 at distances of 2.1 and 2.7 pm. There was 1 hydrogen bond with ILE-116 and SER-119 at the distances of 2.6 and 2.3 pm (Fig. 11A–I; Table 3 or Appendix Figures 15–21, Supplemental Digital Content, http://links.lww.com/MD/N56, http://links.lww.com/MD/N57, http://links.lww.com/MD/N58, http://links.lww.com/MD/N59, http://links.lww.com/MD/N60, http://links.lww.com/MD/N61, http://links.lww.com/MD/N62). It can be seen that quercetin and kaempferol are the main active ingredients of AM–LA drug-pairs in treating KOA.

**Table 3:** The results of drug and disease molecular docking.

Ingredient	Molecular formula	Molecular weight (g/mol)	Crystal structure	Target	Affinity energy (kJ/mol)
Kaempferol	C_15_H_10_O_6_	286.24	6y3v	JUN	−6.3
Quercetin	C_15_H_10_O_7_	302.23			−6.4
Quercetin	C_15_H_10_O_7_	302.23	4zzn	MAPK1	−7.5
7-O-methylisomucronulatol	C_18_H_20_O_5_	316.3	3k3i	MAPK14	−7.9
Mucronulatol	C_17_H_18_O_5_	302.32			−7.8
Calycosin	C_16_H_12_O_5_	284.26			−7.8
Isorhamnetin	C_16_H_12_O_7_	316.26			−8.2
Quercetin	C_15_H_10_O_7_	302.23	4y7r	MYC	−8.0
Quercetin	C_15_H_10_O_7_	302.23	6wqx	TP53	−8.0

## 4. Discussion

KOA is categorized within TCM as “XI BI,” a syndrome characterized by a dual nature of deficiency and excess, stemming from internal organ debility and the encroachment of external pathogenic factors such as wind, cold, and dampness. The etiology of KOA, as understood by TCM, often involves dietary habits skewed towards cold, fatty, sweet, and rich foods. Over time, these dietary preferences, compounded by exposure to external dampness and dietary excesses, disrupt the balance of spleen and stomach functions, leading to dampness entrapment within these organs. Consequently, the disease manifests predominantly as a syndrome of damp evil.^[15]^ In TCM therapeutic strategies, revitalizing the body’s vital energy and expelling dampness are considered pivotal for restoring health and vigor. TCM posits a profound connection between immunity and the concept of Wei Qi, which is believed to originate from the essence of water and grain processed by the spleen and stomach. Therefore, the AM–LA drug pairs are theorized to influence the body’s immune function significantly, thereby modulating the chronic inflammation characteristic of KOA. This perspective underscores the holistic approach of TCM in addressing the complex interplay between diet, environmental factors, organ health, and disease progression, highlighting the therapeutic potential of AM and LA in managing KOA through immune regulation and dampness alleviation.^[16]^

AM, esteemed for its medicinal root, boasts an illustrious heritage in traditional medicine, first documented in the seminal “Shen Nong’s Classic of the Materia Medica,” where it was classified as a substance of the highest efficacy.^[17]^ Esteemed for its qi-enhancing, exterior-securing, diuretic, detoxifying, and abscess-healing properties, contemporary pharmacological investigations have unveiled AM’s hepatoprotective qualities, its capacity to elevate white blood cell counts, and its significant enhancement of the mononuclear macrophage system alongside leukocyte phagocytosis.^[18]^ LA, a perennial herb belonging to the Atractylodes genus of the Compositae family, also finds its roots in the venerable “Shen Nong’s Classic of the Materia Medica.” It was distinguished from *Atractylodes lancea* by Tao Hongjing in the “Collected Commentaries on ‘Shen Nong’s Classic of the Materia Medica’” during the Liang Dynasty, noted for its spleen-invigorating, qi-replenishing, and diuretic effects. In the realm of evidence-based clinical research, both AM and LA have been recognized for their potential in mitigating KOA, with studies highlighting the significant role their active components play in decelerating KOA’s progression. However, research into the synergistic effects and mechanisms underlying their combined use remains scant, marking an area ripe for scientific exploration. This gap underscores a critical need for deeper investigations into how the integration of AM and LA can harness their full therapeutic potential against KOA, paving the way for innovative treatment paradigms rooted in the rich legacy of traditional medicine.

The paradigm of TCM has evolved over millennia, establishing a distinct theoretical foundation and methodology for herbal utilization. Central to its practice is the principle of compatibility, wherein herbs are synergistically combined to enhance efficacy and mitigate toxicity.^[19]^ Despite this long-standing tradition, contemporary clinical research examining the synergistic effects and mechanisms of herbal combinations remains sparse. This study seeks to bridge this gap by employing cutting-edge network pharmacology techniques to dissect the complex interplay between drugs and their biological targets. Through comprehensive database queries spanning genes, proteins, diseases, and drugs, coupled with empirical data, we aim to construct a detailed “drug-gene-target-disease” network. This approach endeavors to reconstitute the balance within biological networks through pharmacological interventions, specifically investigating the impact of AM–LA combinations on KOA treatment.^[20]^ Moreover, the holistic and integrative nature of network pharmacology research aligns seamlessly with TCM’s diagnostic and therapeutic principles, reflecting the tradition’s emphasis on multicomponent, multi-pathway, and multi-target synergies.^[21]^ Thus, exploring the mechanisms of AM–LA drug pairs in KOA treatment through network pharmacology, augmented by differential gene expression analysis from the GEO database, holds paramount significance. It promises to enrich our understanding and provide valuable insights for foundational research and clinical practices, marking a significant stride towards integrating traditional wisdom with contemporary scientific methodologies.

In our investigation, the TCMSP, the TCMID, and the ETCM, alongside an extensive review of existing literature, were employed to delineate the active constituents of AM and LA. Through meticulous ADME profiling and literature analysis, 9 pivotal active ingredients were identified, among which quercetin and kaempferol emerged as the most influential due to their extensive target actions, with 133 and 51 targets respectively. The significance of these components was further corroborated by mechanistic studies, underscoring their critical role in the context of KOA. Kaempferol is noted for its anti-inflammatory, antioxidant, and antitumor properties.^[22]^ Its mechanism of action encompasses modulation of transcription factor activity, pro-inflammatory enzymes, inflammation-associated genes, and matrix metalloproteinases (MMPs).^[23]^ Notably, kaempferol’s anti-inflammatory efficacy is attributed to its regulation of the p38 signaling pathway, MAPK-related extracellular signal-regulated kinase, and activation of the PI3K/AKT/mTOR signaling cascade.^[24]^ Furthermore, kaempferol has been documented to mitigate inflammatory cytokine levels, such as IL-1β, by inhibiting the NF-κB pathway and counteracting IL-1κ-induced pro-inflammatory responses in osteoarthritis chondrocytes.^[25]^ Quercetin, on the other hand, exhibits anti-inflammatory, analgesic, antiplatelet, and anticoagulant effects. It attenuates TNF-α and IL-1 secretion by inhibiting the phosphorylation of AKT and NF-κB p65.^[26]^ Notably, quercetin has been observed to enhance t-PA expression via p38MAPK pathway activation, contributing to thrombosis delay and circulation improvement in KOA-affected areas.^[27]^ Additionally, quercetin’s regulatory effect on the NF-κB pathway modulates MMP-13 and TIMP-1 gene expression in chondrocytes, thereby balancing oxidative stress responses, diminishing extracellular matrix degradation, and preserving articular cartilage. Moreover, evidence suggests that the pharmacological actions of AM–LA compounds extend to regulating immune-inflammatory responses, enhancing hemodynamics, and bolstering immune system activity.^[28]^ This comprehensive analysis highlights the multifaceted mechanisms by which quercetin and kaempferol, as key components of AM and LA, contribute to the therapeutic management of KOA, providing a solid foundation for further exploration of their synergistic potential in TCM formulations.^[29]^

In this investigation, we meticulously harnessed 3 gene expression datasets from the GEO database and 5 distinct databases to pinpoint targets for the AM and LA treatment of KOA, identifying MYC, TP53, MAPK1, MAPK14, and JUN as pivotal genes. MYC is implicated in osteoclastogenesis and the metabolic reprogramming of osteoclasts, primarily by inducing the expression of estrogen receptor-related receptor α, thus promoting osteoclast formation.^[30,31]^ TP53, renowned as a tumor suppressor gene, has been increasingly recognized for its role in modulating the innate immune system and serving as an antigen within the adaptive immune response.^[32]^ MAPK1 and MAPK14, both members of the MAPK family, play critical roles in cell growth, differentiation, inflammation, and adaptation to environmental stress, with their elevated expression linked to cartilage degradation in both in vivo and in vitro models.^[33,34]^ JUN regulates cellular differentiation, proliferation, and apoptosis, notably inhibiting TNF-α-induced apoptosis in synergy with NF-κB, thereby modulating inflammatory-immune responses.^[35,36]^ This suggests that AM–LA may influence KOA development through interventions targeting inflammation, apoptosis, and cell metabolism. To elucidate the potential mechanisms by which AM–LA drug pairs might treat KOA, we conducted GO and KEGG enrichment analyses on the core targets. These analyses revealed significant contributions of the core targets to BPs, cellular components, and MFs, identifying 123 cellular components such as the synaptic membrane, postsynaptic membrane, and ion channel complex. Furthermore, 288 MFs were enriched, predominantly encompassing channel activity, passive transmembrane transporter activity, and ion channel activity. The BPs enrichment encompassed 2535 items, notably involving responses to nutrient levels, steroid hormones, and antibiotics. In terms of cellular composition, 39 items were enriched, focusing on plasma membrane components and the oxidation-reduction process. Additionally, 20 KOA-related pathways were identified, leading to the construction of a “target-pathway” network that encompasses immune regulation, classical inflammation, multiple cancer pathways, and retroactive ligand–receptor interaction, alongside cell proliferation and apoptosis, underscoring the multifaceted mechanism by which AM–LA drug pairs may exert their therapeutic effects on KOA.

In this research, we have meticulously delineated the signaling pathways most significantly associated with the therapeutic targets of AM and LA drug pairs, identifying the MAPK pathway as the most closely related, followed by the PI3K–AKT signaling pathway, calcium signaling pathway, and pathways involved in immune-inflammatory regulation and cancer. The MAPK signaling pathway, comprising a consortium of serine/threonine protein kinases, is activated by diverse extracellular stimuli to orchestrate key cellular functions including growth, differentiation, adaptation to environmental stresses, and inflammation.^[33]^ Specifically, within the context of KOA, inflammatory mediators can trigger the MAPK pathway, elevating MMPs such as MMP1, thereby compromising articular chondrocytes.^[37]^ This pathway also modulates the proliferation, differentiation, migration, and apoptosis of synoviocytes and chondrocytes through the regulation of intracellular and extracellular ion metabolism and gene transcription.^[38]^ In the realm of inflammation and immunity, cellular stimulation leads to the phosphorylation of upstream kinases in the MAPK cascade, culminating in the activation of P38MAPK, ERKMAPK, and JNKMAPK. This activation facilitates MAPK signal transduction, participating in the inflammatory process and upregulating pro-inflammatory cytokines such as TNF-α and IL-6 in inflammatory cells.^[39]^ The PI3K/AKT signaling pathway, frequently activated in tumorigenesis, plays a pivotal role in apoptosis inhibition, cell proliferation, and cell cycle regulation by modulating downstream effector molecules.^[40,41]^ This pathway is crucial for the homeostasis of articular chondrocytes, with evidence suggesting its inhibition in KOA patients. Furthermore, the NF-κB pathway, regulated by PI3K/AKT, is intimately linked to inflammation.^[42]^ The TNF, associated with the inflammatory phenotype of KOA and the NF-κB pathway, underscores the therapeutic potential of inhibiting the TNF pathway in alleviating KOA progression and enhancing patient quality of life.^[43]^

In our study, beyond the previously elucidated MAPK and PI3K–AKT signaling pathways, several other pathways have been identified as instrumental in the therapeutic action of AM and LA against KOA. These include: Calcium Signaling Pathway: this pathway plays a pivotal role in translating physical and chemical stimuli into biological signals, influencing chondrocyte differentiation.^[44]^ Calcium ions have been found to regulate joint nutrition supply, osmotic pressure, and chondrocyte apoptosis, highlighting their significance in joint homeostasis.^[45]^ AGE-RAGE Signaling Pathway: The interaction between AGEs and their receptor RAGE activates signaling cascades such as NF-κB, MAPK, or JAK/STAT.^[46,47]^ This activation contributes to the pathophysiological processes in chronic age-related diseases and diabetic complications, implicating its role in inflammation, proliferation, and cellular mobility within the context of KOA.^[48,49]^ TNF Signaling Pathway: Central to cellular growth, proliferation, inflammation, and immunity, the TNF pathway, upon activation, induces NF-κB nuclear translocation. This activation cascades into the production and release of pro-inflammatory cytokines (TNF-α, IL-8, IL-6), exacerbating the body’s inflammatory response.^[50]^ IL-17 Signaling Pathway: IL-17, closely associated with autoimmune diseases, plays a critical role in the inflammatory response and cytokine production post-tissue injury.^[51]^ Research by Li et al^[52]^ highlighted that the JNK/c-Jun and p38/c-Fos pathways, activated by IL-17A, upregulate COX-2 expression and PGE2 production, thereby mediating inflammation in tissue damage. HIF-1 Signaling Pathway: The HIF-1 pathway facilitates interactions between synovial fibroblasts and T-B cells, inducing inflammatory cytokines and antibodies that expedite KOA progression.^[53]^ HIF-1, a crucial factor in angiogenesis and immune regulation, also supports chondrocyte and synovial cell repair under hypoxic conditions through its involvement in angiogenesis, cell cycle regulation, and energy metabolism.^[54]^ Moreover, immuno-inflammatory signaling pathways such as NF-κB, p53, and Toll-like receptor pathways play significant roles in AM and LA’s therapeutic approach to KOA. Additionally, signaling pathways related to chemical carcinogenesis-receptor activation, reactive oxygen species, microRNAs in cancer, prostate cancer, and other cancer pathways, though less frequently discussed in KOA research, present novel avenues for exploration.

In this study, molecular docking techniques were utilized to evaluate the interaction strength between small-molecule ligands of compounds and their protein receptors, with the premise that a lower binding energy signifies a more stable and high-affinity ligand–receptor interaction. The docking results revealed that the minimum binding energies across all compound-receptor pairs were below −5.0 kcal/mol, underscoring an optimal binding efficiency and a robust affinity between the corresponding ligands and receptors. The analysis of the AM and LA drug pairs in modulating differentially expressed target genes associated with KOA revealed several key insights: core target genes such as MYC, TP53, MAPK1, MAPK14, and JUN are concurrently influenced by multiple active components, indicating a multifaceted regulation of these genes by diverse bioactive molecules. Active components, notably kaempferol and quercetin, interact with several target genes simultaneously, illustrating their multifunctional role in modulating the pathophysiology of KOA through cross-target gene interactions. Specifically, quercetin was found to target 10 differential genes, with 3 being downregulated and 7 upregulated. This suggests that certain active components within the AM–LA drug pairs exert both inhibitory and stimulatory effects on gene expression, indicating a bidirectional regulatory capacity on the gene expression landscape associated with KOA. Thus, the AM–LA drug pairs exhibit a nuanced and bidirectional approach to modulating the pathological underpinnings of KOA, leveraging multiple active components and target genes to influence the disease’s progression and manifestation.

## 5. Conclusion

In conclusion, the AM–LA drug combination orchestrates the therapeutic management of KOA through a multifaceted approach that encompasses multiple targets, components, and signaling pathways. This regulatory mechanism is characterized by its cooperative and bidirectional nature. The pharmacological efficacy of AM–LA is mediated by modulating the expression of key targets such as MYC, TP53, MAPK1, MAPK14, and JUN, alongside the activation or suppression of immune-inflammatory and oncogenic signaling pathways, including the MAPK, calcium, and PI3K/AKT pathways. This comprehensive modulation spans immune-inflammatory responses, cellular proliferation, differentiation, apoptosis, and the antioxidant stress response, underscoring the complex interplay at work in mitigating KOA. While this study lays a foundational theoretical basis and proposes novel research directions, it acknowledges inherent limitations that pave the way for future exploratory avenues. Directions for improvement include: pursuing targeted basic research on the core components of the AM–LA drug combination, informed by network pharmacology findings, through avenues such as serum pharmacology and the pharmacological analysis of active compound prescription components. Conducting further clinical trials to evaluate the drug combination’s efficacy and suitability for the long-term management of patients with KOA. These steps are crucial for translating theoretical insights into practical therapeutic strategies, advancing our understanding and treatment of KOA.

## Author contributions

**Investigation:** Xinyou Zhao.

**Methodology:** Hui Wang, Zixuan Wu.

**Project administration:** Hui Wang.

**Software:** Zixuan Wu.

**Writing – original draft:** Hui Wang, Zixuan Wu.

**Writing – review & editing:** Hui Wang, Xinyou Zhao.

## Supplementary Material
