# Supplementary material for: Mechanism of drug-pairs Astragalus Mongholicus–Largehead Atractylodes on treating knee osteoarthritis investigated by GEO gene chip with network pharmacology and molecular docking
Source: Medicine (Baltimore). 2024 Jul 5;103(27):e38699. doi: 10.1097/MD.0000000000038699 (PMC11224889; doi:10.1097/MD.0000000000038699)
Supplement: Supplementary file 3 [file medi-103-e38699-s003.doc]

# Appendix 3

## **The active ingredients from TCMID database and relevant** **literature**

**Table S3. The active ingredients from TCMID database and relevant literature**

| Drug | MolId | MolName | Symbol | Source |
| --- | --- | --- | --- | --- |
| Largehead Atractylodes | MOL16666 | Herb alcohol | TRPM8 | literature |
| Largehead Atractylodes | MOL16666 | Herb alcohol | CA2 | literature |
| Largehead Atractylodes | MOL16666 | Herb alcohol | CA1 | literature |
| Largehead Atractylodes | MOL16666 | Herb alcohol | CA4 | literature |
| Largehead Atractylodes | MOL16666 | Herb alcohol | ESR2 | literature |
| Largehead Atractylodes | MOL16666 | Herb alcohol | SHBG | literature |
| Largehead Atractylodes | MOL16666 | Herb alcohol | ESR1 | literature |
| Largehead Atractylodes | MOL16666 | Herb alcohol | UGT2B7 | literature |
| Largehead Atractylodes | MOL94225 | Taraxerol acetate | PTGES | literature |
| Largehead Atractylodes | MOL94225 | Taraxerol acetate | HSD11B1 | literature |
| Largehead Atractylodes | MOL94225 | Taraxerol acetate | CES2 | literature |
| Largehead Atractylodes | MOL94225 | Taraxerol acetate | PTPN1 | literature |
| Largehead Atractylodes | MOL94225 | Taraxerol acetate | CYP17A1 | literature |
| Largehead Atractylodes | MOL94225 | Taraxerol acetate | TAS2R31 | literature |
| Largehead Atractylodes | MOL94225 | Taraxerol acetate | CYP19A1 | literature |
| Largehead Atractylodes | MOL101351161 | (+)-eudesma-4(15),7(11)-dien-8-one | SRD5A1 | TCMID |
| Largehead Atractylodes | MOL101351161 | (+)-eudesma-4(15),7(11)-dien-8-one | SRD5A2 | TCMID |
| Largehead Atractylodes | MOL101351161 | (+)-eudesma-4(15),7(11)-dien-8-one | CYP19A1 | TCMID |
| Largehead Atractylodes | MOL101351161 | (+)-eudesma-4(15),7(11)-dien-8-one | TRPA1 | TCMID |
| Largehead Atractylodes | MOL101351161 | (+)-eudesma-4(15),7(11)-dien-8-one | SERPINA6 | TCMID |
| Largehead Atractylodes | MOL101351161 | (+)-eudesma-4(15),7(11)-dien-8-one | SHBG | TCMID |
| Largehead Atractylodes | MOL101351161 | (+)-eudesma-4(15),7(11)-dien-8-one | NR1I2 | TCMID |
| Largehead Atractylodes | MOL160782 | 4-ethoxycarbonyl-2-quinolone | MAPK8 | TCMID |
| Largehead Atractylodes | MOL160782 | 4-ethoxycarbonyl-2-quinolone | CHEK1 | TCMID |
| Largehead Atractylodes | MOL160782 | 4-ethoxycarbonyl-2-quinolone | PDE7A | TCMID |
| Largehead Atractylodes | MOL160782 | 4-ethoxycarbonyl-2-quinolone | GRM4 | TCMID |
| Largehead Atractylodes | MOL160782 | 4-ethoxycarbonyl-2-quinolone | NOTUM | TCMID |
| Largehead Atractylodes | MOL160782 | 4-ethoxycarbonyl-2-quinolone | GCK | TCMID |
| Largehead Atractylodes | MOL11368212 | atractylenolide | CYP19A1 | TCMID |
| Largehead Atractylodes | MOL11368212 | atractylenolide | EPHX1 | TCMID |
| Largehead Atractylodes | MOL11368212 | atractylenolide | PGR | TCMID |
| Largehead Atractylodes | MOL11368212 | atractylenolide | TBXAS1 | TCMID |
| Largehead Atractylodes | MOL11368212 | atractylenolide | PPARG | TCMID |
| Largehead Atractylodes | MOL5321047 | atractylodin | SLC6A3 | TCMID |
| Largehead Atractylodes | MOL5321047 | atractylodin | CTSC | TCMID |
| Largehead Atractylodes | MOL5321047 | atractylodin | CTSF | TCMID |
| Largehead Atractylodes | MOL5321047 | atractylodin | CTSK | TCMID |
| Largehead Atractylodes | MOL5321047 | atractylodin | CTSS | TCMID |
| Largehead Atractylodes | MOL11379068 | hinesol | CYP19A1 | TCMID |
| Largehead Atractylodes | MOL11379068 | hinesol | 5HTT | TCMID |
| Largehead Atractylodes | MOL11379068 | hinesol | CYP17A1 | TCMID |
| Largehead Atractylodes | MOL11379068 | hinesol | ESR1 | TCMID |
| Largehead Atractylodes | MOL11379068 | hinesol | CHRM2 | TCMID |
| Largehead Atractylodes | MOL11379068 | hinesol | ACHE | TCMID |
| Largehead Atractylodes | MOL11379068 | hinesol | NET | TCMID |
| Largehead Atractylodes | MOL11379068 | hinesol | CYP2C19 | TCMID |
| Largehead Atractylodes | MOL52931419 | jurubine | CDK1 | TCMID |
| Largehead Atractylodes | MOL52931419 | jurubine | PSENEN | TCMID |
| Largehead Atractylodes | MOL52931419 | jurubine | VEGFA | TCMID |
| Largehead Atractylodes | MOL52931419 | jurubine | FGF1 | TCMID |
| Largehead Atractylodes | MOL52931419 | jurubine | FGF2 | TCMID |
| Largehead Atractylodes | MOL52931419 | jurubine | HPSE | TCMID |
| Largehead Atractylodes | MOL52931419 | jurubine | HSP90AA1 | TCMID |
| Largehead Atractylodes | MOL52931419 | jurubine | LGALS4 | TCMID |
| Largehead Atractylodes | MOL52931419 | jurubine | LGALS3 | TCMID |
| Largehead Atractylodes | MOL52931419 | jurubine | LGALS8 | TCMID |
| Astragalus mongholicus | MOL162933 | (6ar, 11ar)-10-hydroxy-3, 9-dimethoxypterocarpane | PTPN1 | TCMID |
| Astragalus mongholicus | MOL162933 | (6ar, 11ar)-10-hydroxy-3, 9-dimethoxypterocarpane | ESR1 | TCMID |
| Astragalus mongholicus | MOL162933 | (6ar, 11ar)-10-hydroxy-3, 9-dimethoxypterocarpane | ESR2 | TCMID |
| Astragalus mongholicus | MOL162933 | (6ar, 11ar)-10-hydroxy-3, 9-dimethoxypterocarpane | CYP19A1 | TCMID |
| Astragalus mongholicus | MOL162933 | (6ar, 11ar)-10-hydroxy-3, 9-dimethoxypterocarpane | RPS6KA5 | TCMID |
| Astragalus mongholicus | MOL162933 | (6ar, 11ar)-10-hydroxy-3, 9-dimethoxypterocarpane | MAOA | TCMID |
| Astragalus mongholicus | MOL162933 | (6ar, 11ar)-10-hydroxy-3, 9-dimethoxypterocarpane | GSK3B | TCMID |
| Astragalus mongholicus | MOL162933 | (6ar, 11ar)-10-hydroxy-3, 9-dimethoxypterocarpane | EIF4A1 | TCMID |
| Astragalus mongholicus | MOL5318279 | 2-hydroxy-3-methoxystrychnine | NEK1 | TCMID |
| Astragalus mongholicus | MOL5318279 | 2-hydroxy-3-methoxystrychnine | JAK3 | TCMID |
| Astragalus mongholicus | MOL5318279 | 2-hydroxy-3-methoxystrychnine | JAK1 | TCMID |
| Astragalus mongholicus | MOL5318279 | 2-hydroxy-3-methoxystrychnine | JAK2 | TCMID |
| Astragalus mongholicus | MOL5318279 | 2-hydroxy-3-methoxystrychnine | ADRB3 | TCMID |
| Astragalus mongholicus | MOL21125454 | 20(r)-21,24-cyclo-3beta,25-dihydroxyl-dammar-23(24)-en-21-one | NPC1L1 | TCMID |
| Astragalus mongholicus | MOL21125454 | 20(r)-21,24-cyclo-3beta,25-dihydroxyl-dammar-23(24)-en-21-one | HSD11B1 | TCMID |
| Astragalus mongholicus | MOL21125454 | 20(r)-21,24-cyclo-3beta,25-dihydroxyl-dammar-23(24)-en-21-one | CYP19A1 | TCMID |
| Astragalus mongholicus | MOL21125454 | 20(r)-21,24-cyclo-3beta,25-dihydroxyl-dammar-23(24)-en-21-one | NR3C1 | TCMID |
| Astragalus mongholicus | MOL21125454 | 20(r)-21,24-cyclo-3beta,25-dihydroxyl-dammar-23(24)-en-21-one | SERPINA6 | TCMID |
| Astragalus mongholicus | MOL21125454 | 20(r)-21,24-cyclo-3beta,25-dihydroxyl-dammar-23(24)-en-21-one | SHBG | TCMID |
| Astragalus mongholicus | MOL21125454 | 20(r)-21,24-cyclo-3beta,25-dihydroxyl-dammar-23(24)-en-21-one | PTPN1 | TCMID |
| Astragalus mongholicus | MOL21125454 | 20(r)-21,24-cyclo-3beta,25-dihydroxyl-dammar-23(24)-en-21-one | PGR | TCMID |
| Astragalus mongholicus | MOL5318035 | 20-hexadecanoylingenol | IL1B | TCMID |
| Astragalus mongholicus | MOL5318035 | 20-hexadecanoylingenol | GSTM1 | TCMID |
| Astragalus mongholicus | MOL5318035 | 20-hexadecanoylingenol | VAV1 | TCMID |
| Astragalus mongholicus | MOL5318035 | 20-hexadecanoylingenol | ATP2A1 | TCMID |
| Astragalus mongholicus | MOL5316874 | 3,5-dimethoxystilbene | NQO2 | TCMID |
| Astragalus mongholicus | MOL5316874 | 3,5-dimethoxystilbene | PTGS1 | TCMID |
| Astragalus mongholicus | MOL5316874 | 3,5-dimethoxystilbene | CYP1B1 | TCMID |
| Astragalus mongholicus | MOL5316874 | 3,5-dimethoxystilbene | PTGS2 | TCMID |
| Astragalus mongholicus | MOL5316874 | 3,5-dimethoxystilbene | TUBB1 | TCMID |
| Astragalus mongholicus | MOL5316874 | 3,5-dimethoxystilbene | ESR1 | TCMID |
| Astragalus mongholicus | MOL5316874 | 3,5-dimethoxystilbene | AHR | TCMID |
| Astragalus mongholicus | MOL5316874 | 3,5-dimethoxystilbene | ABCB1 | TCMID |
| Astragalus mongholicus | MOL5316874 | 3,5-dimethoxystilbene | CYP19A1 | TCMID |
| Astragalus mongholicus | MOL5316874 | 3,5-dimethoxystilbene | CYP1A1 | TCMID |
| Astragalus mongholicus | MOL125142 | 6-dimethoxy-isoflavane | TYR | TCMID |
| Astragalus mongholicus | MOL125142 | 6-dimethoxy-isoflavane | SLC5A2 | TCMID |
| Astragalus mongholicus | MOL125142 | 6-dimethoxy-isoflavane | SLC5A1 | TCMID |
| Astragalus mongholicus | MOL125142 | 6-dimethoxy-isoflavane | SLC29A1 | TCMID |
| Astragalus mongholicus | MOL125142 | 6-dimethoxy-isoflavane | PTGS1 | TCMID |
| Astragalus mongholicus | MOL125142 | 6-dimethoxy-isoflavane | MMP8 | TCMID |
| Astragalus mongholicus | MOL29927927 | acetic acid | CA2 | TCMID |
| Astragalus mongholicus | MOL29927927 | acetic acid | CA1 | TCMID |
| Astragalus mongholicus | MOL29927927 | acetic acid | AHR | TCMID |
| Astragalus mongholicus | MOL29927927 | acetic acid | CA7 | TCMID |
| Astragalus mongholicus | MOL29927927 | acetic acid | CA12 | TCMID |
| Astragalus mongholicus | MOL29927927 | acetic acid | CA14 | TCMID |
| Astragalus mongholicus | MOL29927927 | acetic acid | CA9 | TCMID |
| Astragalus mongholicus | MOL60961 | adeninenucleoside | DPP4 | TCMID |
| Astragalus mongholicus | MOL60961 | adeninenucleoside | ADK | TCMID |
| Astragalus mongholicus | MOL60961 | adeninenucleoside | HSPA8 | TCMID |
| Astragalus mongholicus | MOL60961 | adeninenucleoside | HSPA5 | TCMID |
| Astragalus mongholicus | MOL60961 | adeninenucleoside | ADA | TCMID |
| Astragalus mongholicus | MOL60961 | adeninenucleoside | AHCYL2 | TCMID |
| Astragalus mongholicus | MOL53486374 | cycloastragenol | GBA | TCMID |
| Astragalus mongholicus | MOL53486374 | cycloastragenol | PSENEN | TCMID |
| Astragalus mongholicus | MOL53486374 | cycloastragenol | GC | TCMID |
| Astragalus mongholicus | MOL53486374 | cycloastragenol | CCR1 | TCMID |
| Astragalus mongholicus | MOL53486374 | cycloastragenol | EPHX1 | TCMID |
| Astragalus mongholicus | MOL53486374 | cycloastragenol | F2R | TCMID |
| Astragalus mongholicus | MOL53486374 | cycloastragenol | CA2 | TCMID |
| Astragalus mongholicus | MOL13943286 | cyclosieversigenin | GBA | TCMID |
| Astragalus mongholicus | MOL13943286 | cyclosieversigenin | PSENEN | TCMID |
| Astragalus mongholicus | MOL13943286 | cyclosieversigenin | GC | TCMID |
| Astragalus mongholicus | MOL13943286 | cyclosieversigenin | CCR1 | TCMID |
| Astragalus mongholicus | MOL13943286 | cyclosieversigenin | EPHX1 | TCMID |
| Astragalus mongholicus | MOL13943286 | cyclosieversigenin | F2R | TCMID |
| Astragalus mongholicus | MOL13943286 | cyclosieversigenin | CA2 | TCMID |
| Astragalus mongholicus | MOL6037 | folicacid | TYMS | TCMID |
| Astragalus mongholicus | MOL6037 | folicacid | DHFR | TCMID |
| Astragalus mongholicus | MOL6037 | folicacid | FOLR1 | TCMID |
| Astragalus mongholicus | MOL6037 | folicacid | FOLR2 | TCMID |
| Astragalus mongholicus | MOL6037 | folicacid | HDAC6 | TCMID |
| Astragalus mongholicus | MOL6037 | folicacid | SLC46A1 | TCMID |
| Astragalus mongholicus | MOL6037 | folicacid | HDAC8 | TCMID |
| Astragalus mongholicus | MOL6037 | folicacid | HDAC1 | TCMID |
| Astragalus mongholicus | MOL6037 | folicacid | FPGS | TCMID |
| Astragalus mongholicus | MOL636741 | gamma-sitosterol | HMGCR | TCMID |
| Astragalus mongholicus | MOL636741 | gamma-sitosterol | NPC1L1 | TCMID |
| Astragalus mongholicus | MOL636741 | gamma-sitosterol | CYP17A1 | TCMID |
| Astragalus mongholicus | MOL636741 | gamma-sitosterol | RORC | TCMID |
| Astragalus mongholicus | MOL636741 | gamma-sitosterol | CYP19A1 | TCMID |
| Astragalus mongholicus | MOL636741 | gamma-sitosterol | ESR2 | TCMID |
| Astragalus mongholicus | MOL636741 | gamma-sitosterol | ESR1 | TCMID |
| Astragalus mongholicus | MOL520159 | hexadecanoicacid | CA2 | TCMID |
| Astragalus mongholicus | MOL520159 | hexadecanoicacid | CA1 | TCMID |
| Astragalus mongholicus | MOL520159 | hexadecanoicacid | HSD11B1 | TCMID |
| Astragalus mongholicus | MOL520159 | hexadecanoicacid | FFAR1 | TCMID |
| Astragalus mongholicus | MOL520159 | hexadecanoicacid | PPARD | TCMID |
| Astragalus mongholicus | MOL60148697 | isoastragaloside i | PSENEN | TCMID |
| Astragalus mongholicus | MOL60148697 | isoastragaloside i | VEGFA | TCMID |
| Astragalus mongholicus | MOL60148697 | isoastragaloside i | FGF1 | TCMID |
| Astragalus mongholicus | MOL60148697 | isoastragaloside i | FGF2 | TCMID |
| Astragalus mongholicus | MOL60148697 | isoastragaloside i | HPSE | TCMID |
| Astragalus mongholicus | MOL60148697 | isoastragaloside i | RORC | TCMID |
| Astragalus mongholicus | MOL60148697 | isoastragaloside i | GLRA1 | TCMID |
| Astragalus mongholicus | MOL60148697 | isoastragaloside i | GLRA2 | TCMID |
| Astragalus mongholicus | MOL60148655 | isoastragaloside ii | PSENEN | TCMID |
| Astragalus mongholicus | MOL60148655 | isoastragaloside ii | HSP90AA1 | TCMID |
| Astragalus mongholicus | MOL60148655 | isoastragaloside ii | VEGFA | TCMID |
| Astragalus mongholicus | MOL60148655 | isoastragaloside ii | FGF1 | TCMID |
| Astragalus mongholicus | MOL60148655 | isoastragaloside ii | FGF2 | TCMID |
| Astragalus mongholicus | MOL60148655 | isoastragaloside ii | HPSE | TCMID |
| Astragalus mongholicus | MOL3 | isoastragaloside1, 3 | KDM4E | TCMID |
| Astragalus mongholicus | MOL3 | isoastragaloside1, 3 | KDM2A | TCMID |
| Astragalus mongholicus | MOL3 | isoastragaloside1, 3 | KDM6B | TCMID |
| Astragalus mongholicus | MOL3 | isoastragaloside1, 3 | PHF8 | TCMID |
| Astragalus mongholicus | MOL3 | isoastragaloside1, 3 | KDM5C | TCMID |
| Astragalus mongholicus | MOL3 | isoastragaloside1, 3 | ACHE | TCMID |
| Astragalus mongholicus | MOL3 | isoastragaloside1, 3 | BACE1 | TCMID |
| Astragalus mongholicus | MOL3 | isoastragaloside1, 3 | ESR2 | TCMID |
| Astragalus mongholicus | MOL3 | isoastragaloside1, 3 | CA1 | TCMID |
| Astragalus mongholicus | MOL3 | isoastragaloside1, 3 | CA9 | TCMID |
| Astragalus mongholicus | MOL5318869 | kumatakenin | NOX4 | TCMID |
| Astragalus mongholicus | MOL5318869 | kumatakenin | ABCB1 | TCMID |
| Astragalus mongholicus | MOL5318869 | kumatakenin | MCL1 | TCMID |
| Astragalus mongholicus | MOL5318869 | kumatakenin | ABCG2 | TCMID |
| Astragalus mongholicus | MOL5318869 | kumatakenin | ESR2 | TCMID |
| Astragalus mongholicus | MOL5318869 | kumatakenin | SLC22A12 | TCMID |
| Astragalus mongholicus | MOL5317378 | kumugansine a | MAOA | TCMID |
| Astragalus mongholicus | MOL5317378 | kumugansine a | CTSK | TCMID |
| Astragalus mongholicus | MOL5317378 | kumugansine a | CTSS | TCMID |
| Astragalus mongholicus | MOL5317378 | kumugansine a | GSK3B | TCMID |
| Astragalus mongholicus | MOL5317378 | kumugansine a | MPO | TCMID |
| Astragalus mongholicus | MOL5317378 | kumugansine a | TYMP | TCMID |
| Astragalus mongholicus | MOL5317378 | kumugansine a | CISD1 | TCMID |
| Astragalus mongholicus | MOL5317378 | kumugansine a | MAPK8 | TCMID |
| Astragalus mongholicus | MOL23135 | n-candicine | NMUR2 | TCMID |
| Astragalus mongholicus | MOL23135 | n-candicine | KDM4E | TCMID |
| Astragalus mongholicus | MOL23135 | n-candicine | NET | TCMID |
| Astragalus mongholicus | MOL23135 | n-candicine | OPRM1 | TCMID |
| Astragalus mongholicus | MOL44246636 | soyasapogenol b | PTPN1 | TCMID |
| Astragalus mongholicus | MOL44246636 | soyasapogenol b | CYP2C19 | TCMID |
| Astragalus mongholicus | MOL44246636 | soyasapogenol b | CYP19A1 | TCMID |
| Astragalus mongholicus | MOL44246636 | soyasapogenol b | ESR1 | TCMID |
| Astragalus mongholicus | MOL44246636 | soyasapogenol b | CHRM2 | TCMID |
| Astragalus mongholicus | MOL53301851 | sucrose | CDK1 | TCMID |
| Astragalus mongholicus | MOL53301851 | sucrose | HSP90AA1 | TCMID |
| Astragalus mongholicus | MOL53301851 | sucrose | VEGFA | TCMID |
| Astragalus mongholicus | MOL53301851 | sucrose | PSENEN | TCMID |
| Astragalus mongholicus | MOL53301851 | sucrose | FGF1 | TCMID |
| Astragalus mongholicus | MOL53301851 | sucrose | HPSE | TCMID |
| Astragalus mongholicus | MOL53301851 | sucrose | FGF2 | TCMID |
| Astragalus mongholicus | MOL53301851 | sucrose | LGALS4 | TCMID |
| Astragalus mongholicus | MOL53301851 | sucrose | LGALS3 | TCMID |
| Astragalus mongholicus | MOL53301851 | sucrose | LGALS8 | TCMID |
| Astragalus mongholicus | MOL9986231 | suffruticoside a | TDP1 | TCMID |
| Astragalus mongholicus | MOL9986231 | suffruticoside a | EPHX1 | TCMID |
| Astragalus mongholicus | MOL9986231 | suffruticoside a | CYP19A1 | TCMID |
| Astragalus mongholicus | MOL9986231 | suffruticoside a | SLC5A2 | TCMID |
| Astragalus mongholicus | MOL9986231 | suffruticoside a | SLC28A3 | TCMID |
| Astragalus mongholicus | MOL9986231 | suffruticoside a | SLC5A2 | TCMID |
| Astragalus mongholicus | MOL9986231 | suffruticoside a | ESR1 | TCMID |
| Astragalus mongholicus | MOL9986231 | suffruticoside a | PTPN2 | TCMID |
| Astragalus mongholicus | MOL45356795 | uridine | CDA | TCMID |
| Astragalus mongholicus | MOL45356795 | uridine | ADA | TCMID |
| Astragalus mongholicus | MOL45356795 | uridine | TK1 | TCMID |
| Astragalus mongholicus | MOL45356795 | uridine | OGT | TCMID |
| Astragalus mongholicus | MOL45356795 | uridine | CA12 | TCMID |
| Astragalus mongholicus | MOL222284 | β-sitosterol | HMGCR | TCMID |
| Astragalus mongholicus | MOL222284 | β-sitosterol | NPC1L1 | TCMID |
| Astragalus mongholicus | MOL222284 | β-sitosterol | CYP17A1 | TCMID |
| Astragalus mongholicus | MOL222284 | β-sitosterol | RORC | TCMID |
| Astragalus mongholicus | MOL222284 | β-sitosterol | CYP19A1 | TCMID |
| Astragalus mongholicus | MOL222284 | β-sitosterol | ESR2 | TCMID |
| Astragalus mongholicus | MOL222284 | β-sitosterol | ESR1 | TCMID |
