# Supplementary material for: Mechanism of drug-pairs Astragalus Mongholicus–Largehead Atractylodes on treating knee osteoarthritis investigated by GEO gene chip with network pharmacology and molecular docking
Source: Medicine (Baltimore). 2024 Jul 5;103(27):e38699. doi: 10.1097/MD.0000000000038699 (PMC11224889; doi:10.1097/MD.0000000000038699)
Supplement: Supplementary file 4 [file medi-103-e38699-s004.doc]

# Appendix 4

## **The active ingredients from ETCM database and relevant literature**

**Table S4. AM of the active ingredients from ETCM database.**

| Drug | MolId | MolName | Symbol |
| --- | --- | --- | --- |
| Astragalus mongholicus | MOL500 | (Trans-Trans)Farnesol,3,7,11-Trimethyldodeca-2,6,10-Trien-1-Ol,Farnesol | MAOB |
| Astragalus mongholicus | MOL501 | (Trans-Trans)Farnesol,3,7,11-Trimethyldodeca-2,6,10-Trien-1-Ol,Farnesol | NR1H4 |
| Astragalus mongholicus | MOL502 | 1,7,7-Trimethylbicyclo[2.2.1]Heptan-2-Ol,Borneol | AKR1D1 |
| Astragalus mongholicus | MOL503 | 1,7,7-Trimethylbicyclo[2.2.1]Heptan-2-Ol,Borneol | AR |
| Astragalus mongholicus | MOL504 | 1,7,7-Trimethylbicyclo[2.2.1]Heptan-2-Ol,Borneol | CACNA1C |
| Astragalus mongholicus | MOL505 | 1,7,7-Trimethylbicyclo[2.2.1]Heptan-2-Ol,Borneol | CACNA1D |
| Astragalus mongholicus | MOL506 | 1,7,7-Trimethylbicyclo[2.2.1]Heptan-2-Ol,Borneol | CACNA1F |
| Astragalus mongholicus | MOL507 | 1,7,7-Trimethylbicyclo[2.2.1]Heptan-2-Ol,Borneol | CACNA1S |
| Astragalus mongholicus | MOL508 | 1,7,7-Trimethylbicyclo[2.2.1]Heptan-2-Ol,Borneol | CACNB1 |
| Astragalus mongholicus | MOL509 | 1,7,7-Trimethylbicyclo[2.2.1]Heptan-2-Ol,Borneol | CACNB2 |
| Astragalus mongholicus | MOL510 | 1,7,7-Trimethylbicyclo[2.2.1]Heptan-2-Ol,Borneol | CACNB3 |
| Astragalus mongholicus | MOL511 | 1,7,7-Trimethylbicyclo[2.2.1]Heptan-2-Ol,Borneol | CACNB4 |
| Astragalus mongholicus | MOL512 | 1,7,7-Trimethylbicyclo[2.2.1]Heptan-2-Ol,Borneol | ESR1 |
| Astragalus mongholicus | MOL513 | 1,7,7-Trimethylbicyclo[2.2.1]Heptan-2-Ol,Borneol | ESR2 |
| Astragalus mongholicus | MOL514 | 1,7,7-Trimethylbicyclo[2.2.1]Heptan-2-Ol,Borneol | GABRA1 |
| Astragalus mongholicus | MOL515 | 1,7,7-Trimethylbicyclo[2.2.1]Heptan-2-Ol,Borneol | GABRA2 |
| Astragalus mongholicus | MOL516 | 1,7,7-Trimethylbicyclo[2.2.1]Heptan-2-Ol,Borneol | GABRA3 |
| Astragalus mongholicus | MOL517 | 1,7,7-Trimethylbicyclo[2.2.1]Heptan-2-Ol,Borneol | GABRA4 |
| Astragalus mongholicus | MOL518 | 1,7,7-Trimethylbicyclo[2.2.1]Heptan-2-Ol,Borneol | GABRA5 |
| Astragalus mongholicus | MOL519 | 1,7,7-Trimethylbicyclo[2.2.1]Heptan-2-Ol,Borneol | GABRA6 |
| Astragalus mongholicus | MOL520 | 1,7,7-Trimethylbicyclo[2.2.1]Heptan-2-Ol,Borneol | GABRB1 |
| Astragalus mongholicus | MOL521 | 1,7,7-Trimethylbicyclo[2.2.1]Heptan-2-Ol,Borneol | GABRB2 |
| Astragalus mongholicus | MOL522 | 1,7,7-Trimethylbicyclo[2.2.1]Heptan-2-Ol,Borneol | GABRB3 |
| Astragalus mongholicus | MOL523 | 1,7,7-Trimethylbicyclo[2.2.1]Heptan-2-Ol,Borneol | GABRD |
| Astragalus mongholicus | MOL524 | 1,7,7-Trimethylbicyclo[2.2.1]Heptan-2-Ol,Borneol | GABRE |
| Astragalus mongholicus | MOL525 | 1,7,7-Trimethylbicyclo[2.2.1]Heptan-2-Ol,Borneol | GABRG1 |
| Astragalus mongholicus | MOL526 | 1,7,7-Trimethylbicyclo[2.2.1]Heptan-2-Ol,Borneol | GABRG2 |
| Astragalus mongholicus | MOL527 | 1,7,7-Trimethylbicyclo[2.2.1]Heptan-2-Ol,Borneol | GABRG3 |
| Astragalus mongholicus | MOL528 | 1,7,7-Trimethylbicyclo[2.2.1]Heptan-2-Ol,Borneol | GABRP |
| Astragalus mongholicus | MOL529 | 1,7,7-Trimethylbicyclo[2.2.1]Heptan-2-Ol,Borneol | GABRQ |
| Astragalus mongholicus | MOL530 | 1,7,7-Trimethylbicyclo[2.2.1]Heptan-2-Ol,Borneol | HSD17B1 |
| Astragalus mongholicus | MOL531 | 1,7,7-Trimethylbicyclo[2.2.1]Heptan-2-Ol,Borneol | HSD17B11 |
| Astragalus mongholicus | MOL532 | 1,7,7-Trimethylbicyclo[2.2.1]Heptan-2-Ol,Borneol | IGHG2 |
| Astragalus mongholicus | MOL533 | 1,7,7-Trimethylbicyclo[2.2.1]Heptan-2-Ol,Borneol | LSS |
| Astragalus mongholicus | MOL534 | 1,7,7-Trimethylbicyclo[2.2.1]Heptan-2-Ol,Borneol | NR1I3 |
| Astragalus mongholicus | MOL535 | 1,7,7-Trimethylbicyclo[2.2.1]Heptan-2-Ol,Borneol | NR3C2 |
| Astragalus mongholicus | MOL536 | 1,7,7-Trimethylbicyclo[2.2.1]Heptan-2-Ol,Borneol | OPRK1 |
| Astragalus mongholicus | MOL537 | 1,7,7-Trimethylbicyclo[2.2.1]Heptan-2-Ol,Borneol | SULT2A1 |
| Astragalus mongholicus | MOL538 | 1,7,7-Trimethylbicyclo[2.2.1]Heptan-2-Ol,Borneol | TRPA1 |
| Astragalus mongholicus | MOL539 | 1,7,7-Trimethylbicyclo[2.2.1]Heptan-2-Ol,Borneol | TRPM8 |
| Astragalus mongholicus | MOL540 | 1,7,7-Trimethylbicyclo[2.2.1]Heptan-2-Ol,Borneol | TRPV3 |
| Astragalus mongholicus | MOL541 | 1,7,7-Trimethylbicyclo[2.2.1]Heptan-2-Ol,Borneol | VDR |
| Astragalus mongholicus | MOL542 | Camphor | AKR1D1 |
| Astragalus mongholicus | MOL543 | Camphor | NCOA1 |
| Astragalus mongholicus | MOL544 | Camphor | NR1I3 |
| Astragalus mongholicus | MOL545 | Camphor | RXRA |
| Astragalus mongholicus | MOL546 | Camphor | TRPA1 |
| Astragalus mongholicus | MOL547 | Camphor | TRPM8 |
| Astragalus mongholicus | MOL548 | Camphor | TRPV1 |
| Astragalus mongholicus | MOL549 | Camphor | TRPV3 |
| Astragalus mongholicus | MOL550 | 1-Isopropyl-4-Methylcyclohex-3-Enol,4-Terpineol | VDR |
| Astragalus mongholicus | MOL551 | (1R,2R,4R)-1,7,7-Trimethylbicyclo[2.2.1]Heptan-2-Ol,Isoborneol,L-Isoborneol | AKR1D1 |
| Astragalus mongholicus | MOL552 | (1R,2R,4R)-1,7,7-Trimethylbicyclo[2.2.1]Heptan-2-Ol,Isoborneol,L-Isoborneol | AR |
| Astragalus mongholicus | MOL553 | (1R,2R,4R)-1,7,7-Trimethylbicyclo[2.2.1]Heptan-2-Ol,Isoborneol,L-Isoborneol | CACNA1C |
| Astragalus mongholicus | MOL554 | (1R,2R,4R)-1,7,7-Trimethylbicyclo[2.2.1]Heptan-2-Ol,Isoborneol,L-Isoborneol | CACNA1D |
| Astragalus mongholicus | MOL555 | (1R,2R,4R)-1,7,7-Trimethylbicyclo[2.2.1]Heptan-2-Ol,Isoborneol,L-Isoborneol | CACNA1F |
| Astragalus mongholicus | MOL556 | (1R,2R,4R)-1,7,7-Trimethylbicyclo[2.2.1]Heptan-2-Ol,Isoborneol,L-Isoborneol | CACNA1S |
| Astragalus mongholicus | MOL557 | (1R,2R,4R)-1,7,7-Trimethylbicyclo[2.2.1]Heptan-2-Ol,Isoborneol,L-Isoborneol | CACNB1 |
| Astragalus mongholicus | MOL558 | (1R,2R,4R)-1,7,7-Trimethylbicyclo[2.2.1]Heptan-2-Ol,Isoborneol,L-Isoborneol | CACNB2 |
| Astragalus mongholicus | MOL559 | (1R,2R,4R)-1,7,7-Trimethylbicyclo[2.2.1]Heptan-2-Ol,Isoborneol,L-Isoborneol | CACNB3 |
| Astragalus mongholicus | MOL560 | (1R,2R,4R)-1,7,7-Trimethylbicyclo[2.2.1]Heptan-2-Ol,Isoborneol,L-Isoborneol | CACNB4 |
| Astragalus mongholicus | MOL561 | (1R,2R,4R)-1,7,7-Trimethylbicyclo[2.2.1]Heptan-2-Ol,Isoborneol,L-Isoborneol | ESR1 |
| Astragalus mongholicus | MOL562 | (1R,2R,4R)-1,7,7-Trimethylbicyclo[2.2.1]Heptan-2-Ol,Isoborneol,L-Isoborneol | ESR2 |
| Astragalus mongholicus | MOL563 | (1R,2R,4R)-1,7,7-Trimethylbicyclo[2.2.1]Heptan-2-Ol,Isoborneol,L-Isoborneol | GABRA1 |
| Astragalus mongholicus | MOL564 | (1R,2R,4R)-1,7,7-Trimethylbicyclo[2.2.1]Heptan-2-Ol,Isoborneol,L-Isoborneol | GABRA2 |
| Astragalus mongholicus | MOL565 | (1R,2R,4R)-1,7,7-Trimethylbicyclo[2.2.1]Heptan-2-Ol,Isoborneol,L-Isoborneol | GABRA3 |
| Astragalus mongholicus | MOL566 | (1R,2R,4R)-1,7,7-Trimethylbicyclo[2.2.1]Heptan-2-Ol,Isoborneol,L-Isoborneol | GABRA4 |
| Astragalus mongholicus | MOL567 | (1R,2R,4R)-1,7,7-Trimethylbicyclo[2.2.1]Heptan-2-Ol,Isoborneol,L-Isoborneol | GABRA5 |
| Astragalus mongholicus | MOL568 | (1R,2R,4R)-1,7,7-Trimethylbicyclo[2.2.1]Heptan-2-Ol,Isoborneol,L-Isoborneol | GABRA6 |
| Astragalus mongholicus | MOL569 | (1R,2R,4R)-1,7,7-Trimethylbicyclo[2.2.1]Heptan-2-Ol,Isoborneol,L-Isoborneol | GABRB1 |
| Astragalus mongholicus | MOL570 | (1R,2R,4R)-1,7,7-Trimethylbicyclo[2.2.1]Heptan-2-Ol,Isoborneol,L-Isoborneol | GABRB2 |
| Astragalus mongholicus | MOL571 | (1R,2R,4R)-1,7,7-Trimethylbicyclo[2.2.1]Heptan-2-Ol,Isoborneol,L-Isoborneol | GABRB3 |
| Astragalus mongholicus | MOL572 | (1R,2R,4R)-1,7,7-Trimethylbicyclo[2.2.1]Heptan-2-Ol,Isoborneol,L-Isoborneol | GABRD |
| Astragalus mongholicus | MOL573 | (1R,2R,4R)-1,7,7-Trimethylbicyclo[2.2.1]Heptan-2-Ol,Isoborneol,L-Isoborneol | GABRE |
| Astragalus mongholicus | MOL574 | (1R,2R,4R)-1,7,7-Trimethylbicyclo[2.2.1]Heptan-2-Ol,Isoborneol,L-Isoborneol | GABRG1 |
| Astragalus mongholicus | MOL575 | (1R,2R,4R)-1,7,7-Trimethylbicyclo[2.2.1]Heptan-2-Ol,Isoborneol,L-Isoborneol | GABRG2 |
| Astragalus mongholicus | MOL576 | (1R,2R,4R)-1,7,7-Trimethylbicyclo[2.2.1]Heptan-2-Ol,Isoborneol,L-Isoborneol | GABRG3 |
| Astragalus mongholicus | MOL577 | (1R,2R,4R)-1,7,7-Trimethylbicyclo[2.2.1]Heptan-2-Ol,Isoborneol,L-Isoborneol | GABRP |
| Astragalus mongholicus | MOL578 | (1R,2R,4R)-1,7,7-Trimethylbicyclo[2.2.1]Heptan-2-Ol,Isoborneol,L-Isoborneol | GABRQ |
| Astragalus mongholicus | MOL579 | (1R,2R,4R)-1,7,7-Trimethylbicyclo[2.2.1]Heptan-2-Ol,Isoborneol,L-Isoborneol | HSD17B1 |
| Astragalus mongholicus | MOL580 | (1R,2R,4R)-1,7,7-Trimethylbicyclo[2.2.1]Heptan-2-Ol,Isoborneol,L-Isoborneol | HSD17B11 |
| Astragalus mongholicus | MOL581 | (1R,2R,4R)-1,7,7-Trimethylbicyclo[2.2.1]Heptan-2-Ol,Isoborneol,L-Isoborneol | IGHG2 |
| Astragalus mongholicus | MOL582 | (1R,2R,4R)-1,7,7-Trimethylbicyclo[2.2.1]Heptan-2-Ol,Isoborneol,L-Isoborneol | LSS |
| Astragalus mongholicus | MOL583 | (1R,2R,4R)-1,7,7-Trimethylbicyclo[2.2.1]Heptan-2-Ol,Isoborneol,L-Isoborneol | NR1I3 |
| Astragalus mongholicus | MOL584 | (1R,2R,4R)-1,7,7-Trimethylbicyclo[2.2.1]Heptan-2-Ol,Isoborneol,L-Isoborneol | NR3C2 |
| Astragalus mongholicus | MOL585 | (1R,2R,4R)-1,7,7-Trimethylbicyclo[2.2.1]Heptan-2-Ol,Isoborneol,L-Isoborneol | OPRK1 |
| Astragalus mongholicus | MOL586 | (1R,2R,4R)-1,7,7-Trimethylbicyclo[2.2.1]Heptan-2-Ol,Isoborneol,L-Isoborneol | SULT2A1 |
| Astragalus mongholicus | MOL587 | (1R,2R,4R)-1,7,7-Trimethylbicyclo[2.2.1]Heptan-2-Ol,Isoborneol,L-Isoborneol | TRPA1 |
| Astragalus mongholicus | MOL588 | (1R,2R,4R)-1,7,7-Trimethylbicyclo[2.2.1]Heptan-2-Ol,Isoborneol,L-Isoborneol | TRPM8 |
| Astragalus mongholicus | MOL589 | (1R,2R,4R)-1,7,7-Trimethylbicyclo[2.2.1]Heptan-2-Ol,Isoborneol,L-Isoborneol | TRPV3 |
| Astragalus mongholicus | MOL590 | (1R,2R,4R)-1,7,7-Trimethylbicyclo[2.2.1]Heptan-2-Ol,Isoborneol,L-Isoborneol | VDR |
| Astragalus mongholicus | MOL591 | 1,7,7-Trimethylbicyclo[2.2.1]Heptan-2-YlAcetate,BornylAcetate | AR |
| Astragalus mongholicus | MOL592 | 1,7,7-Trimethylbicyclo[2.2.1]Heptan-2-YlAcetate,BornylAcetate | ESR1 |
| Astragalus mongholicus | MOL593 | 1,7,7-Trimethylbicyclo[2.2.1]Heptan-2-YlAcetate,BornylAcetate | HSD17B1 |
| Astragalus mongholicus | MOL594 | 1,7,7-Trimethylbicyclo[2.2.1]Heptan-2-YlAcetate,BornylAcetate | NR3C2 |
| Astragalus mongholicus | MOL595 | 1,7,7-Trimethylbicyclo[2.2.1]Heptan-2-YlAcetate,BornylAcetate | TRPA1 |
| Astragalus mongholicus | MOL596 | 1,7,7-Trimethylbicyclo[2.2.1]Heptan-2-YlAcetate,BornylAcetate | TRPM8 |
| Astragalus mongholicus | MOL597 | 1,7,7-Trimethylbicyclo[2.2.1]Heptan-2-YlAcetate,BornylAcetate | TRPV1 |
| Astragalus mongholicus | MOL598 | 1,7,7-Trimethylbicyclo[2.2.1]Heptan-2-YlAcetate,BornylAcetate | TRPV3 |
| Astragalus mongholicus | MOL599 | (S)-5-Hydroxy-7-Methoxy-2-Phenylchroman-4-One,7-Hydroxy-5-Methoxyflavanone | AKR1C1 |
| Astragalus mongholicus | MOL600 | (S)-5-Hydroxy-7-Methoxy-2-Phenylchroman-4-One,7-Hydroxy-5-Methoxyflavanone | CYP19A1 |
| Astragalus mongholicus | MOL601 | (S)-5-Hydroxy-7-Methoxy-2-Phenylchroman-4-One,7-Hydroxy-5-Methoxyflavanone | CYP1B1 |
| Astragalus mongholicus | MOL602 | (S)-5-Hydroxy-7-Methoxy-2-Phenylchroman-4-One,7-Hydroxy-5-Methoxyflavanone | ESR1 |
| Astragalus mongholicus | MOL603 | (S)-5-Hydroxy-7-Methoxy-2-Phenylchroman-4-One,7-Hydroxy-5-Methoxyflavanone | ESR2 |
| Astragalus mongholicus | MOL604 | (S)-5-Hydroxy-7-Methoxy-2-Phenylchroman-4-One,7-Hydroxy-5-Methoxyflavanone | KANSL3 |
| Astragalus mongholicus | MOL605 | (S)-5-Hydroxy-7-Methoxy-2-Phenylchroman-4-One,7-Hydroxy-5-Methoxyflavanone | MTTP |
| Astragalus mongholicus | MOL606 | (S)-5-Hydroxy-7-Methoxy-2-Phenylchroman-4-One,7-Hydroxy-5-Methoxyflavanone | SHBG |
| Astragalus mongholicus | MOL607 | (S)-5-Hydroxy-7-Methoxy-2-Phenylchroman-4-One,7-Hydroxy-5-Methoxyflavanone | SOAT1 |
| Astragalus mongholicus | MOL608 | (S)-5-Hydroxy-7-Methoxy-2-Phenylchroman-4-One,7-Hydroxy-5-Methoxyflavanone | SOAT2 |
| Astragalus mongholicus | MOL609 | (S)-5,7-Dihydroxy-2-Phenylchroman-4-One,Pinocembrin | ACTB |
| Astragalus mongholicus | MOL610 | (S)-5,7-Dihydroxy-2-Phenylchroman-4-One,Pinocembrin | AHR |
| Astragalus mongholicus | MOL611 | (S)-5,7-Dihydroxy-2-Phenylchroman-4-One,Pinocembrin | AKR1C1 |
| Astragalus mongholicus | MOL612 | (S)-5,7-Dihydroxy-2-Phenylchroman-4-One,Pinocembrin | AKT1 |
| Astragalus mongholicus | MOL613 | (S)-5,7-Dihydroxy-2-Phenylchroman-4-One,Pinocembrin | ATP5A1 |
| Astragalus mongholicus | MOL614 | (S)-5,7-Dihydroxy-2-Phenylchroman-4-One,Pinocembrin | ATP5B |
| Astragalus mongholicus | MOL615 | (S)-5,7-Dihydroxy-2-Phenylchroman-4-One,Pinocembrin | ATP5C1 |
| Astragalus mongholicus | MOL616 | (S)-5,7-Dihydroxy-2-Phenylchroman-4-One,Pinocembrin | CBR1 |
| Astragalus mongholicus | MOL617 | (S)-5,7-Dihydroxy-2-Phenylchroman-4-One,Pinocembrin | CDK6 |
| Astragalus mongholicus | MOL618 | (S)-5,7-Dihydroxy-2-Phenylchroman-4-One,Pinocembrin | CEBPB |
| Astragalus mongholicus | MOL619 | (S)-5,7-Dihydroxy-2-Phenylchroman-4-One,Pinocembrin | CSNK2A1 |
| Astragalus mongholicus | MOL620 | (S)-5,7-Dihydroxy-2-Phenylchroman-4-One,Pinocembrin | CSNK2B |
| Astragalus mongholicus | MOL621 | (S)-5,7-Dihydroxy-2-Phenylchroman-4-One,Pinocembrin | CYP19A1 |
| Astragalus mongholicus | MOL622 | (S)-5,7-Dihydroxy-2-Phenylchroman-4-One,Pinocembrin | CYP1B1 |
| Astragalus mongholicus | MOL623 | (S)-5,7-Dihydroxy-2-Phenylchroman-4-One,Pinocembrin | EIF3F |
| Astragalus mongholicus | MOL624 | (S)-5,7-Dihydroxy-2-Phenylchroman-4-One,Pinocembrin | ESR1 |
| Astragalus mongholicus | MOL625 | (S)-5,7-Dihydroxy-2-Phenylchroman-4-One,Pinocembrin | ESR2 |
| Astragalus mongholicus | MOL626 | (S)-5,7-Dihydroxy-2-Phenylchroman-4-One,Pinocembrin | ESRRA |
| Astragalus mongholicus | MOL627 | (S)-5,7-Dihydroxy-2-Phenylchroman-4-One,Pinocembrin | ESRRB |
| Astragalus mongholicus | MOL628 | (S)-5,7-Dihydroxy-2-Phenylchroman-4-One,Pinocembrin | GPER1 |
| Astragalus mongholicus | MOL629 | (S)-5,7-Dihydroxy-2-Phenylchroman-4-One,Pinocembrin | HCK |
| Astragalus mongholicus | MOL630 | (S)-5,7-Dihydroxy-2-Phenylchroman-4-One,Pinocembrin | HIBCH |
| Astragalus mongholicus | MOL631 | (S)-5,7-Dihydroxy-2-Phenylchroman-4-One,Pinocembrin | HSP90AA1 |
| Astragalus mongholicus | MOL632 | (S)-5,7-Dihydroxy-2-Phenylchroman-4-One,Pinocembrin | HSPA2 |
| Astragalus mongholicus | MOL633 | (S)-5,7-Dihydroxy-2-Phenylchroman-4-One,Pinocembrin | IGHG1 |
| Astragalus mongholicus | MOL634 | (S)-5,7-Dihydroxy-2-Phenylchroman-4-One,Pinocembrin | JAK1 |
| Astragalus mongholicus | MOL635 | (S)-5,7-Dihydroxy-2-Phenylchroman-4-One,Pinocembrin | KANSL3 |
| Astragalus mongholicus | MOL636 | (S)-5,7-Dihydroxy-2-Phenylchroman-4-One,Pinocembrin | MTTP |
| Astragalus mongholicus | MOL637 | (S)-5,7-Dihydroxy-2-Phenylchroman-4-One,Pinocembrin | NCOA1 |
| Astragalus mongholicus | MOL638 | (S)-5,7-Dihydroxy-2-Phenylchroman-4-One,Pinocembrin | NCOA2 |
| Astragalus mongholicus | MOL639 | (S)-5,7-Dihydroxy-2-Phenylchroman-4-One,Pinocembrin | NQO2 |
| Astragalus mongholicus | MOL640 | (S)-5,7-Dihydroxy-2-Phenylchroman-4-One,Pinocembrin | NR1I2 |
| Astragalus mongholicus | MOL641 | (S)-5,7-Dihydroxy-2-Phenylchroman-4-One,Pinocembrin | PIK3CG |
| Astragalus mongholicus | MOL642 | (S)-5,7-Dihydroxy-2-Phenylchroman-4-One,Pinocembrin | PIM1 |
| Astragalus mongholicus | MOL643 | (S)-5,7-Dihydroxy-2-Phenylchroman-4-One,Pinocembrin | PTK2B |
| Astragalus mongholicus | MOL644 | (S)-5,7-Dihydroxy-2-Phenylchroman-4-One,Pinocembrin | RUVBL2 |
| Astragalus mongholicus | MOL645 | (S)-5,7-Dihydroxy-2-Phenylchroman-4-One,Pinocembrin | SF3B3 |
| Astragalus mongholicus | MOL646 | (S)-5,7-Dihydroxy-2-Phenylchroman-4-One,Pinocembrin | SHBG |
| Astragalus mongholicus | MOL647 | (S)-5,7-Dihydroxy-2-Phenylchroman-4-One,Pinocembrin | SOAT1 |
| Astragalus mongholicus | MOL648 | (S)-5,7-Dihydroxy-2-Phenylchroman-4-One,Pinocembrin | SOAT2 |
| Astragalus mongholicus | MOL649 | (S)-5,7-Dihydroxy-2-Phenylchroman-4-One,Pinocembrin | STK17B |
| Astragalus mongholicus | MOL650 | (S)-5,7-Dihydroxy-2-Phenylchroman-4-One,Pinocembrin | TOP2A |
| Astragalus mongholicus | MOL651 | (S)-5,7-Dihydroxy-2-Phenylchroman-4-One,Pinocembrin | UBA1 |
| Astragalus mongholicus | MOL652 | (S)-5,7-Dihydroxy-2-Phenylchroman-4-One,Pinocembrin | UGT3A1 |
| Astragalus mongholicus | MOL653 | 1-(2,4-Dihydroxy-6-Methoxyphenyl)-3-Phenylprop-2-En-1-One,Cardamonin | AR |
| Astragalus mongholicus | MOL654 | 1-(2,4-Dihydroxy-6-Methoxyphenyl)-3-Phenylprop-2-En-1-One,Cardamonin | CTRB1 |
| Astragalus mongholicus | MOL655 | 1-(2,4-Dihydroxy-6-Methoxyphenyl)-3-Phenylprop-2-En-1-One,Cardamonin | ESR1 |
| Astragalus mongholicus | MOL656 | 1-(2,4-Dihydroxy-6-Methoxyphenyl)-3-Phenylprop-2-En-1-One,Cardamonin | ESR2 |
| Astragalus mongholicus | MOL657 | 1-(2,4-Dihydroxy-6-Methoxyphenyl)-3-Phenylprop-2-En-1-One,Cardamonin | PGR |
| Astragalus mongholicus | MOL658 | 1-(2,4-Dihydroxy-6-Methoxyphenyl)-3-Phenylprop-2-En-1-One,Cardamonin | SHBG |
| Astragalus mongholicus | MOL659 | 1-(2,4-Dihydroxy-6-Methoxyphenyl)-3-Phenylprop-2-En-1-One,Cardamonin | Octanal |
| Astragalus mongholicus | MOL660 | 1-(2,4-Dihydroxy-6-Methoxyphenyl)-3-Phenylprop-2-En-1-One,Cardamonin | ACOT13 |
| Astragalus mongholicus | MOL661 | 1-(2,4-Dihydroxy-6-Methoxyphenyl)-3-Phenylprop-2-En-1-One,Cardamonin | DBI |
| Astragalus mongholicus | MOL662 | 1-(2,4-Dihydroxy-6-Methoxyphenyl)-3-Phenylprop-2-En-1-One,Cardamonin | RHO |
| Astragalus mongholicus | MOL663 | 1-(2,4-Dihydroxy-6-Methoxyphenyl)-3-Phenylprop-2-En-1-One,Cardamonin | Nonan-2-One |
| Astragalus mongholicus | MOL664 | 1-(2,4-Dihydroxy-6-Methoxyphenyl)-3-Phenylprop-2-En-1-One,Cardamonin | ACOT13 |
| Astragalus mongholicus | MOL665 | 1-(2,4-Dihydroxy-6-Methoxyphenyl)-3-Phenylprop-2-En-1-One,Cardamonin | DBI |
| Astragalus mongholicus | MOL666 | 1-(2,4-Dihydroxy-6-Methoxyphenyl)-3-Phenylprop-2-En-1-One,Cardamonin | RHO |
| Astragalus mongholicus | MOL667 | 1-(2,4-Dihydroxy-6-Methoxyphenyl)-3-Phenylprop-2-En-1-One,Cardamonin | Methyl-N-Nonylketone,Undecan-2-One |
| Astragalus mongholicus | MOL668 | 1-(2,4-Dihydroxy-6-Methoxyphenyl)-3-Phenylprop-2-En-1-One,Cardamonin | ACOT13 |
| Astragalus mongholicus | MOL669 | 1-(2,4-Dihydroxy-6-Methoxyphenyl)-3-Phenylprop-2-En-1-One,Cardamonin | DBI |
| Astragalus mongholicus | MOL670 | 1-(2,4-Dihydroxy-6-Methoxyphenyl)-3-Phenylprop-2-En-1-One,Cardamonin | RHO |
| Astragalus mongholicus | MOL671 | (-)-CaryophylleneOxide,(1R,4R,6R,10S)-4,12,12-Trimethyl-9-Methylidene-5-Oxatricyclo[8.2.0.0~4,6~]Dodecane | LCN9 |
| Astragalus mongholicus | MOL672 | (-)-CaryophylleneOxide,(1R,4R,6R,10S)-4,12,12-Trimethyl-9-Methylidene-5-Oxatricyclo[8.2.0.0~4,6~]Dodecane | Methanol |
| Astragalus mongholicus | MOL673 | (-)-CaryophylleneOxide,(1R,4R,6R,10S)-4,12,12-Trimethyl-9-Methylidene-5-Oxatricyclo[8.2.0.0~4,6~]Dodecane | MB |
| Astragalus mongholicus | MOL674 | (-)-CaryophylleneOxide,(1R,4R,6R,10S)-4,12,12-Trimethyl-9-Methylidene-5-Oxatricyclo[8.2.0.0~4,6~]Dodecane | Ethyl3-(4-Methoxyphenyl)Acrylate |
| Astragalus mongholicus | MOL675 | (-)-CaryophylleneOxide,(1R,4R,6R,10S)-4,12,12-Trimethyl-9-Methylidene-5-Oxatricyclo[8.2.0.0~4,6~]Dodecane | RARA |
| Astragalus mongholicus | MOL676 | (-)-CaryophylleneOxide,(1R,4R,6R,10S)-4,12,12-Trimethyl-9-Methylidene-5-Oxatricyclo[8.2.0.0~4,6~]Dodecane | RARB |
| Astragalus mongholicus | MOL677 | (-)-CaryophylleneOxide,(1R,4R,6R,10S)-4,12,12-Trimethyl-9-Methylidene-5-Oxatricyclo[8.2.0.0~4,6~]Dodecane | RARG |
| Astragalus mongholicus | MOL678 | (-)-CaryophylleneOxide,(1R,4R,6R,10S)-4,12,12-Trimethyl-9-Methylidene-5-Oxatricyclo[8.2.0.0~4,6~]Dodecane | RXRA |
| Astragalus mongholicus | MOL679 | (-)-CaryophylleneOxide,(1R,4R,6R,10S)-4,12,12-Trimethyl-9-Methylidene-5-Oxatricyclo[8.2.0.0~4,6~]Dodecane | RXRB |
| Astragalus mongholicus | MOL680 | (-)-CaryophylleneOxide,(1R,4R,6R,10S)-4,12,12-Trimethyl-9-Methylidene-5-Oxatricyclo[8.2.0.0~4,6~]Dodecane | RXRG |
| Astragalus mongholicus | MOL681 | (-)-CaryophylleneOxide,(1R,4R,6R,10S)-4,12,12-Trimethyl-9-Methylidene-5-Oxatricyclo[8.2.0.0~4,6~]Dodecane | Bicyclo[3.3.1]Nonan-2-Ol |
| Astragalus mongholicus | MOL682 | (-)-CaryophylleneOxide,(1R,4R,6R,10S)-4,12,12-Trimethyl-9-Methylidene-5-Oxatricyclo[8.2.0.0~4,6~]Dodecane | ADH1B |
| Astragalus mongholicus | MOL683 | (S)-2-Methyl-6-(4-Methylcyclohex-3-En-1-Yl)Hepta-2,6-Dien-1-Ol | NR1H4 |
| Astragalus mongholicus | MOL684 | (S)-2-Methyl-6-(4-Methylcyclohex-3-En-1-Yl)Hepta-2,6-Dien-1-Ol | MAOB |
| Astragalus mongholicus | MOL685 | (1R,2S,5S)-5-Isopropyl-2-Methylbicyclo[3.1.0]Hexan-2-Ol | NR1I3 |
| Astragalus mongholicus | MOL686 | (8S,8As)-8-Hydroxy-3,5,8A-Trimethyl-7,8,8A,9-Tetrahydronaphtho[2,3-B]Furan-4(6H)-One | AR |
| Astragalus mongholicus | MOL687 | (8S,8As)-8-Hydroxy-3,5,8A-Trimethyl-7,8,8A,9-Tetrahydronaphtho[2,3-B]Furan-4(6H)-One | ESR1 |
| Astragalus mongholicus | MOL688 | (8S,8As)-8-Hydroxy-3,5,8A-Trimethyl-7,8,8A,9-Tetrahydronaphtho[2,3-B]Furan-4(6H)-One | ESR2 |
| Astragalus mongholicus | MOL689 | (8S,8As)-8-Hydroxy-3,5,8A-Trimethyl-7,8,8A,9-Tetrahydronaphtho[2,3-B]Furan-4(6H)-One | GABRA1 |
| Astragalus mongholicus | MOL690 | (8S,8As)-8-Hydroxy-3,5,8A-Trimethyl-7,8,8A,9-Tetrahydronaphtho[2,3-B]Furan-4(6H)-One | GABRA2 |
| Astragalus mongholicus | MOL691 | (8S,8As)-8-Hydroxy-3,5,8A-Trimethyl-7,8,8A,9-Tetrahydronaphtho[2,3-B]Furan-4(6H)-One | GABRA3 |
| Astragalus mongholicus | MOL692 | (8S,8As)-8-Hydroxy-3,5,8A-Trimethyl-7,8,8A,9-Tetrahydronaphtho[2,3-B]Furan-4(6H)-One | GABRA4 |
| Astragalus mongholicus | MOL693 | (8S,8As)-8-Hydroxy-3,5,8A-Trimethyl-7,8,8A,9-Tetrahydronaphtho[2,3-B]Furan-4(6H)-One | GABRA5 |
| Astragalus mongholicus | MOL694 | (8S,8As)-8-Hydroxy-3,5,8A-Trimethyl-7,8,8A,9-Tetrahydronaphtho[2,3-B]Furan-4(6H)-One | GABRA6 |
| Astragalus mongholicus | MOL695 | (8S,8As)-8-Hydroxy-3,5,8A-Trimethyl-7,8,8A,9-Tetrahydronaphtho[2,3-B]Furan-4(6H)-One | GABRB1 |
| Astragalus mongholicus | MOL696 | (8S,8As)-8-Hydroxy-3,5,8A-Trimethyl-7,8,8A,9-Tetrahydronaphtho[2,3-B]Furan-4(6H)-One | GABRB2 |
| Astragalus mongholicus | MOL697 | (8S,8As)-8-Hydroxy-3,5,8A-Trimethyl-7,8,8A,9-Tetrahydronaphtho[2,3-B]Furan-4(6H)-One | GABRB3 |
| Astragalus mongholicus | MOL698 | (8S,8As)-8-Hydroxy-3,5,8A-Trimethyl-7,8,8A,9-Tetrahydronaphtho[2,3-B]Furan-4(6H)-One | GABRD |
| Astragalus mongholicus | MOL699 | (8S,8As)-8-Hydroxy-3,5,8A-Trimethyl-7,8,8A,9-Tetrahydronaphtho[2,3-B]Furan-4(6H)-One | GABRE |
| Astragalus mongholicus | MOL700 | (8S,8As)-8-Hydroxy-3,5,8A-Trimethyl-7,8,8A,9-Tetrahydronaphtho[2,3-B]Furan-4(6H)-One | GABRG1 |
| Astragalus mongholicus | MOL701 | (8S,8As)-8-Hydroxy-3,5,8A-Trimethyl-7,8,8A,9-Tetrahydronaphtho[2,3-B]Furan-4(6H)-One | GABRG2 |
| Astragalus mongholicus | MOL702 | (8S,8As)-8-Hydroxy-3,5,8A-Trimethyl-7,8,8A,9-Tetrahydronaphtho[2,3-B]Furan-4(6H)-One | GABRG3 |
| Astragalus mongholicus | MOL703 | (8S,8As)-8-Hydroxy-3,5,8A-Trimethyl-7,8,8A,9-Tetrahydronaphtho[2,3-B]Furan-4(6H)-One | GABRP |
| Astragalus mongholicus | MOL704 | (8S,8As)-8-Hydroxy-3,5,8A-Trimethyl-7,8,8A,9-Tetrahydronaphtho[2,3-B]Furan-4(6H)-One | GABRQ |
| Astragalus mongholicus | MOL705 | (8S,8As)-8-Hydroxy-3,5,8A-Trimethyl-7,8,8A,9-Tetrahydronaphtho[2,3-B]Furan-4(6H)-One | GRIN1 |
| Astragalus mongholicus | MOL706 | (8S,8As)-8-Hydroxy-3,5,8A-Trimethyl-7,8,8A,9-Tetrahydronaphtho[2,3-B]Furan-4(6H)-One | GRIN2A |
| Astragalus mongholicus | MOL707 | (8S,8As)-8-Hydroxy-3,5,8A-Trimethyl-7,8,8A,9-Tetrahydronaphtho[2,3-B]Furan-4(6H)-One | GRIN2B |
| Astragalus mongholicus | MOL708 | (8S,8As)-8-Hydroxy-3,5,8A-Trimethyl-7,8,8A,9-Tetrahydronaphtho[2,3-B]Furan-4(6H)-One | GRIN2C |
| Astragalus mongholicus | MOL709 | (8S,8As)-8-Hydroxy-3,5,8A-Trimethyl-7,8,8A,9-Tetrahydronaphtho[2,3-B]Furan-4(6H)-One | GRIN2D |
| Astragalus mongholicus | MOL710 | (8S,8As)-8-Hydroxy-3,5,8A-Trimethyl-7,8,8A,9-Tetrahydronaphtho[2,3-B]Furan-4(6H)-One | GRIN3A |
| Astragalus mongholicus | MOL711 | (8S,8As)-8-Hydroxy-3,5,8A-Trimethyl-7,8,8A,9-Tetrahydronaphtho[2,3-B]Furan-4(6H)-One | GRIN3B |
| Astragalus mongholicus | MOL712 | (8S,8As)-8-Hydroxy-3,5,8A-Trimethyl-7,8,8A,9-Tetrahydronaphtho[2,3-B]Furan-4(6H)-One | HSD11B2 |
| Astragalus mongholicus | MOL713 | (8S,8As)-8-Hydroxy-3,5,8A-Trimethyl-7,8,8A,9-Tetrahydronaphtho[2,3-B]Furan-4(6H)-One | HSD17B1 |
| Astragalus mongholicus | MOL714 | (8S,8As)-8-Hydroxy-3,5,8A-Trimethyl-7,8,8A,9-Tetrahydronaphtho[2,3-B]Furan-4(6H)-One | NPPB |
| Astragalus mongholicus | MOL715 | (8S,8As)-8-Hydroxy-3,5,8A-Trimethyl-7,8,8A,9-Tetrahydronaphtho[2,3-B]Furan-4(6H)-One | NR1I2 |
| Astragalus mongholicus | MOL716 | (8S,8As)-8-Hydroxy-3,5,8A-Trimethyl-7,8,8A,9-Tetrahydronaphtho[2,3-B]Furan-4(6H)-One | NR1I3 |
| Astragalus mongholicus | MOL717 | (8S,8As)-8-Hydroxy-3,5,8A-Trimethyl-7,8,8A,9-Tetrahydronaphtho[2,3-B]Furan-4(6H)-One | PPARA |
| Astragalus mongholicus | MOL718 | (8S,8As)-8-Hydroxy-3,5,8A-Trimethyl-7,8,8A,9-Tetrahydronaphtho[2,3-B]Furan-4(6H)-One | SIGMAR1 |
| Astragalus mongholicus | MOL719 | (8S,8As)-8-Hydroxy-3,5,8A-Trimethyl-7,8,8A,9-Tetrahydronaphtho[2,3-B]Furan-4(6H)-One | SULT2A1 |
| Astragalus mongholicus | MOL720 | (8S,8As)-8-Hydroxy-3,5,8A-Trimethyl-7,8,8A,9-Tetrahydronaphtho[2,3-B]Furan-4(6H)-One | SULT2B1 |
| Astragalus mongholicus | MOL721 | (1R,11R)-1,5,9,9-Tetramethyl-12-Oxabicyclo[9.1.0]Dodeca-4,7-Diene | LCN9 |
| Astragalus mongholicus | MOL722 | (1R,11R)-1,5,5,8-Tetramethyl-12-Oxabicyclo[9.1.0]Dodeca-3,7-Diene | LCN9 |
| Astragalus mongholicus | MOL723 | (3S,3As,8Ar,Z)-3-Hydroxy-3,8-Dimethyl-5-(Propan-2-Ylidene)-1,2,3,3A,4,5-Hexahydroazulen-6(8Ah)-One | AKR1C1 |
| Astragalus mongholicus | MOL724 | (3S,3As,8Ar,Z)-3-Hydroxy-3,8-Dimethyl-5-(Propan-2-Ylidene)-1,2,3,3A,4,5-Hexahydroazulen-6(8Ah)-One | AKR1C2 |
| Astragalus mongholicus | MOL725 | (3S,3As,8Ar,Z)-3-Hydroxy-3,8-Dimethyl-5-(Propan-2-Ylidene)-1,2,3,3A,4,5-Hexahydroazulen-6(8Ah)-One | AR |
| Astragalus mongholicus | MOL726 | (3S,3As,8Ar,Z)-3-Hydroxy-3,8-Dimethyl-5-(Propan-2-Ylidene)-1,2,3,3A,4,5-Hexahydroazulen-6(8Ah)-One | ESR1 |
| Astragalus mongholicus | MOL727 | (3S,3As,8Ar,Z)-3-Hydroxy-3,8-Dimethyl-5-(Propan-2-Ylidene)-1,2,3,3A,4,5-Hexahydroazulen-6(8Ah)-One | ESR2 |
| Astragalus mongholicus | MOL728 | (3S,3As,8Ar,Z)-3-Hydroxy-3,8-Dimethyl-5-(Propan-2-Ylidene)-1,2,3,3A,4,5-Hexahydroazulen-6(8Ah)-One | GABRA1 |
| Astragalus mongholicus | MOL729 | (3S,3As,8Ar,Z)-3-Hydroxy-3,8-Dimethyl-5-(Propan-2-Ylidene)-1,2,3,3A,4,5-Hexahydroazulen-6(8Ah)-One | GABRA2 |
| Astragalus mongholicus | MOL730 | (3S,3As,8Ar,Z)-3-Hydroxy-3,8-Dimethyl-5-(Propan-2-Ylidene)-1,2,3,3A,4,5-Hexahydroazulen-6(8Ah)-One | GABRA3 |
| Astragalus mongholicus | MOL731 | (3S,3As,8Ar,Z)-3-Hydroxy-3,8-Dimethyl-5-(Propan-2-Ylidene)-1,2,3,3A,4,5-Hexahydroazulen-6(8Ah)-One | GABRA4 |
| Astragalus mongholicus | MOL732 | (3S,3As,8Ar,Z)-3-Hydroxy-3,8-Dimethyl-5-(Propan-2-Ylidene)-1,2,3,3A,4,5-Hexahydroazulen-6(8Ah)-One | GABRA5 |
| Astragalus mongholicus | MOL733 | (3S,3As,8Ar,Z)-3-Hydroxy-3,8-Dimethyl-5-(Propan-2-Ylidene)-1,2,3,3A,4,5-Hexahydroazulen-6(8Ah)-One | GABRA6 |
| Astragalus mongholicus | MOL734 | (3S,3As,8Ar,Z)-3-Hydroxy-3,8-Dimethyl-5-(Propan-2-Ylidene)-1,2,3,3A,4,5-Hexahydroazulen-6(8Ah)-One | GABRB1 |
| Astragalus mongholicus | MOL735 | (3S,3As,8Ar,Z)-3-Hydroxy-3,8-Dimethyl-5-(Propan-2-Ylidene)-1,2,3,3A,4,5-Hexahydroazulen-6(8Ah)-One | GABRB2 |
| Astragalus mongholicus | MOL736 | (3S,3As,8Ar,Z)-3-Hydroxy-3,8-Dimethyl-5-(Propan-2-Ylidene)-1,2,3,3A,4,5-Hexahydroazulen-6(8Ah)-One | GABRB3 |
| Astragalus mongholicus | MOL737 | (3S,3As,8Ar,Z)-3-Hydroxy-3,8-Dimethyl-5-(Propan-2-Ylidene)-1,2,3,3A,4,5-Hexahydroazulen-6(8Ah)-One | GABRD |
| Astragalus mongholicus | MOL738 | (3S,3As,8Ar,Z)-3-Hydroxy-3,8-Dimethyl-5-(Propan-2-Ylidene)-1,2,3,3A,4,5-Hexahydroazulen-6(8Ah)-One | GABRE |
| Astragalus mongholicus | MOL739 | (3S,3As,8Ar,Z)-3-Hydroxy-3,8-Dimethyl-5-(Propan-2-Ylidene)-1,2,3,3A,4,5-Hexahydroazulen-6(8Ah)-One | GABRG1 |
| Astragalus mongholicus | MOL740 | (3S,3As,8Ar,Z)-3-Hydroxy-3,8-Dimethyl-5-(Propan-2-Ylidene)-1,2,3,3A,4,5-Hexahydroazulen-6(8Ah)-One | GABRG2 |
| Astragalus mongholicus | MOL741 | (3S,3As,8Ar,Z)-3-Hydroxy-3,8-Dimethyl-5-(Propan-2-Ylidene)-1,2,3,3A,4,5-Hexahydroazulen-6(8Ah)-One | GABRG3 |
| Astragalus mongholicus | MOL742 | (3S,3As,8Ar,Z)-3-Hydroxy-3,8-Dimethyl-5-(Propan-2-Ylidene)-1,2,3,3A,4,5-Hexahydroazulen-6(8Ah)-One | GABRP |
| Astragalus mongholicus | MOL743 | (3S,3As,8Ar,Z)-3-Hydroxy-3,8-Dimethyl-5-(Propan-2-Ylidene)-1,2,3,3A,4,5-Hexahydroazulen-6(8Ah)-One | GABRQ |
| Astragalus mongholicus | MOL744 | (3S,3As,8Ar,Z)-3-Hydroxy-3,8-Dimethyl-5-(Propan-2-Ylidene)-1,2,3,3A,4,5-Hexahydroazulen-6(8Ah)-One | GRIN1 |
| Astragalus mongholicus | MOL745 | (3S,3As,8Ar,Z)-3-Hydroxy-3,8-Dimethyl-5-(Propan-2-Ylidene)-1,2,3,3A,4,5-Hexahydroazulen-6(8Ah)-One | GRIN2A |
| Astragalus mongholicus | MOL746 | (3S,3As,8Ar,Z)-3-Hydroxy-3,8-Dimethyl-5-(Propan-2-Ylidene)-1,2,3,3A,4,5-Hexahydroazulen-6(8Ah)-One | GRIN2B |
| Astragalus mongholicus | MOL747 | (3S,3As,8Ar,Z)-3-Hydroxy-3,8-Dimethyl-5-(Propan-2-Ylidene)-1,2,3,3A,4,5-Hexahydroazulen-6(8Ah)-One | GRIN2C |
| Astragalus mongholicus | MOL748 | (3S,3As,8Ar,Z)-3-Hydroxy-3,8-Dimethyl-5-(Propan-2-Ylidene)-1,2,3,3A,4,5-Hexahydroazulen-6(8Ah)-One | GRIN2D |
| Astragalus mongholicus | MOL749 | (3S,3As,8Ar,Z)-3-Hydroxy-3,8-Dimethyl-5-(Propan-2-Ylidene)-1,2,3,3A,4,5-Hexahydroazulen-6(8Ah)-One | GRIN3A |
| Astragalus mongholicus | MOL750 | (3S,3As,8Ar,Z)-3-Hydroxy-3,8-Dimethyl-5-(Propan-2-Ylidene)-1,2,3,3A,4,5-Hexahydroazulen-6(8Ah)-One | GRIN3B |
| Astragalus mongholicus | MOL751 | (3S,3As,8Ar,Z)-3-Hydroxy-3,8-Dimethyl-5-(Propan-2-Ylidene)-1,2,3,3A,4,5-Hexahydroazulen-6(8Ah)-One | HSD17B1 |
| Astragalus mongholicus | MOL752 | (3S,3As,8Ar,Z)-3-Hydroxy-3,8-Dimethyl-5-(Propan-2-Ylidene)-1,2,3,3A,4,5-Hexahydroazulen-6(8Ah)-One | LSS |
| Astragalus mongholicus | MOL753 | (3S,3As,8Ar,Z)-3-Hydroxy-3,8-Dimethyl-5-(Propan-2-Ylidene)-1,2,3,3A,4,5-Hexahydroazulen-6(8Ah)-One | NCOA2 |
| Astragalus mongholicus | MOL754 | (3S,3As,8Ar,Z)-3-Hydroxy-3,8-Dimethyl-5-(Propan-2-Ylidene)-1,2,3,3A,4,5-Hexahydroazulen-6(8Ah)-One | NPPB |
| Astragalus mongholicus | MOL755 | (3S,3As,8Ar,Z)-3-Hydroxy-3,8-Dimethyl-5-(Propan-2-Ylidene)-1,2,3,3A,4,5-Hexahydroazulen-6(8Ah)-One | NR1I2 |
| Astragalus mongholicus | MOL756 | (3S,3As,8Ar,Z)-3-Hydroxy-3,8-Dimethyl-5-(Propan-2-Ylidene)-1,2,3,3A,4,5-Hexahydroazulen-6(8Ah)-One | NR1I3 |
| Astragalus mongholicus | MOL757 | (3S,3As,8Ar,Z)-3-Hydroxy-3,8-Dimethyl-5-(Propan-2-Ylidene)-1,2,3,3A,4,5-Hexahydroazulen-6(8Ah)-One | NR3C2 |
| Astragalus mongholicus | MOL758 | (3S,3As,8Ar,Z)-3-Hydroxy-3,8-Dimethyl-5-(Propan-2-Ylidene)-1,2,3,3A,4,5-Hexahydroazulen-6(8Ah)-One | PGR |
| Astragalus mongholicus | MOL759 | (3S,3As,8Ar,Z)-3-Hydroxy-3,8-Dimethyl-5-(Propan-2-Ylidene)-1,2,3,3A,4,5-Hexahydroazulen-6(8Ah)-One | PPARA |
| Astragalus mongholicus | MOL760 | (3S,3As,8Ar,Z)-3-Hydroxy-3,8-Dimethyl-5-(Propan-2-Ylidene)-1,2,3,3A,4,5-Hexahydroazulen-6(8Ah)-One | SIGMAR1 |
| Astragalus mongholicus | MOL761 | (3S,3As,8Ar,Z)-3-Hydroxy-3,8-Dimethyl-5-(Propan-2-Ylidene)-1,2,3,3A,4,5-Hexahydroazulen-6(8Ah)-One | SULT2A1 |
| Astragalus mongholicus | MOL762 | (3S,3As,8Ar,Z)-3-Hydroxy-3,8-Dimethyl-5-(Propan-2-Ylidene)-1,2,3,3A,4,5-Hexahydroazulen-6(8Ah)-One | SULT2B1 |
| Astragalus mongholicus | MOL763 | (3S,3As,8Ar,Z)-3-Hydroxy-3,8-Dimethyl-5-(Propan-2-Ylidene)-1,2,3,3A,4,5-Hexahydroazulen-6(8Ah)-One | VDR |
| Astragalus mongholicus | MOL764 | 1,7-Bis(4-Hydroxyphenyl)Hepta-1,6-Diene-3,5-Dione | PYGM |
| Astragalus mongholicus | MOL765 | 2,4-Dimethyloctan-4-Ol | LCN9 |
| Astragalus mongholicus | MOL766 | 2,3,3-Trimethyl-2-(3-Methylbuta-1,3-Dien-1-Yl)-6-Methylenecyclohexanone | AR |
| Astragalus mongholicus | MOL767 | 2,3,3-Trimethyl-2-(3-Methylbuta-1,3-Dien-1-Yl)-6-Methylenecyclohexanone | ESR1 |
| Astragalus mongholicus | MOL768 | 2,3,3-Trimethyl-2-(3-Methylbuta-1,3-Dien-1-Yl)-6-Methylenecyclohexanone | ESR2 |
| Astragalus mongholicus | MOL769 | 2,3,3-Trimethyl-2-(3-Methylbuta-1,3-Dien-1-Yl)-6-Methylenecyclohexanone | PGR |
| Astragalus mongholicus | MOL770 | (3Z,7Z)-3,7,10,10-Tetramethyl-12-Oxa-Bicyclo[9.1.0]Dodeca-3,7-Diene | LCN9 |
| Astragalus mongholicus | MOL771 | (3Z,7Z)-3,7,10,10-Tetramethyl-12-Oxa-Bicyclo[9.1.0]Dodeca-3,7-Diene | Decan-2-One |
| Astragalus mongholicus | MOL772 | (3Z,7Z)-3,7,10,10-Tetramethyl-12-Oxa-Bicyclo[9.1.0]Dodeca-3,7-Diene | ACOT13 |
| Astragalus mongholicus | MOL773 | (3Z,7Z)-3,7,10,10-Tetramethyl-12-Oxa-Bicyclo[9.1.0]Dodeca-3,7-Diene | DBI |
| Astragalus mongholicus | MOL774 | (3Z,7Z)-3,7,10,10-Tetramethyl-12-Oxa-Bicyclo[9.1.0]Dodeca-3,7-Diene | RHO |
| Astragalus mongholicus | MOL775 | (5S,8R,9S,10S,13S,14S)-3-Ethyl-3-Hydroxy-10,13-Dimethyl-Tetradecahydro-2H-Cyclopenta[A]Phenanthren-17(14H)-One | ADH1C |
| Astragalus mongholicus | MOL776 | (5S,8R,9S,10S,13S,14S)-3-Ethyl-3-Hydroxy-10,13-Dimethyl-Tetradecahydro-2H-Cyclopenta[A]Phenanthren-17(14H)-One | AKR1C1 |
| Astragalus mongholicus | MOL777 | (5S,8R,9S,10S,13S,14S)-3-Ethyl-3-Hydroxy-10,13-Dimethyl-Tetradecahydro-2H-Cyclopenta[A]Phenanthren-17(14H)-One | AKR1C2 |
| Astragalus mongholicus | MOL778 | (5S,8R,9S,10S,13S,14S)-3-Ethyl-3-Hydroxy-10,13-Dimethyl-Tetradecahydro-2H-Cyclopenta[A]Phenanthren-17(14H)-One | AKR1D1 |
| Astragalus mongholicus | MOL779 | (5S,8R,9S,10S,13S,14S)-3-Ethyl-3-Hydroxy-10,13-Dimethyl-Tetradecahydro-2H-Cyclopenta[A]Phenanthren-17(14H)-One | AR |
| Astragalus mongholicus | MOL780 | (5S,8R,9S,10S,13S,14S)-3-Ethyl-3-Hydroxy-10,13-Dimethyl-Tetradecahydro-2H-Cyclopenta[A]Phenanthren-17(14H)-One | BCL2 |
| Astragalus mongholicus | MOL781 | (5S,8R,9S,10S,13S,14S)-3-Ethyl-3-Hydroxy-10,13-Dimethyl-Tetradecahydro-2H-Cyclopenta[A]Phenanthren-17(14H)-One | CES1 |
| Astragalus mongholicus | MOL782 | (5S,8R,9S,10S,13S,14S)-3-Ethyl-3-Hydroxy-10,13-Dimethyl-Tetradecahydro-2H-Cyclopenta[A]Phenanthren-17(14H)-One | COX4I1 |
| Astragalus mongholicus | MOL783 | (5S,8R,9S,10S,13S,14S)-3-Ethyl-3-Hydroxy-10,13-Dimethyl-Tetradecahydro-2H-Cyclopenta[A]Phenanthren-17(14H)-One | COX5A |
| Astragalus mongholicus | MOL784 | (5S,8R,9S,10S,13S,14S)-3-Ethyl-3-Hydroxy-10,13-Dimethyl-Tetradecahydro-2H-Cyclopenta[A]Phenanthren-17(14H)-One | COX5B |
| Astragalus mongholicus | MOL785 | (5S,8R,9S,10S,13S,14S)-3-Ethyl-3-Hydroxy-10,13-Dimethyl-Tetradecahydro-2H-Cyclopenta[A]Phenanthren-17(14H)-One | COX6A2 |
| Astragalus mongholicus | MOL786 | (5S,8R,9S,10S,13S,14S)-3-Ethyl-3-Hydroxy-10,13-Dimethyl-Tetradecahydro-2H-Cyclopenta[A]Phenanthren-17(14H)-One | COX6B1 |
| Astragalus mongholicus | MOL787 | (5S,8R,9S,10S,13S,14S)-3-Ethyl-3-Hydroxy-10,13-Dimethyl-Tetradecahydro-2H-Cyclopenta[A]Phenanthren-17(14H)-One | COX6C |
| Astragalus mongholicus | MOL788 | (5S,8R,9S,10S,13S,14S)-3-Ethyl-3-Hydroxy-10,13-Dimethyl-Tetradecahydro-2H-Cyclopenta[A]Phenanthren-17(14H)-One | COX7A1 |
| Astragalus mongholicus | MOL789 | (5S,8R,9S,10S,13S,14S)-3-Ethyl-3-Hydroxy-10,13-Dimethyl-Tetradecahydro-2H-Cyclopenta[A]Phenanthren-17(14H)-One | COX7B |
| Astragalus mongholicus | MOL790 | (5S,8R,9S,10S,13S,14S)-3-Ethyl-3-Hydroxy-10,13-Dimethyl-Tetradecahydro-2H-Cyclopenta[A]Phenanthren-17(14H)-One | COX7C |
| Astragalus mongholicus | MOL791 | (5S,8R,9S,10S,13S,14S)-3-Ethyl-3-Hydroxy-10,13-Dimethyl-Tetradecahydro-2H-Cyclopenta[A]Phenanthren-17(14H)-One | COX8A |
| Astragalus mongholicus | MOL792 | (5S,8R,9S,10S,13S,14S)-3-Ethyl-3-Hydroxy-10,13-Dimethyl-Tetradecahydro-2H-Cyclopenta[A]Phenanthren-17(14H)-One | EFTUD1 |
| Astragalus mongholicus | MOL793 | (5S,8R,9S,10S,13S,14S)-3-Ethyl-3-Hydroxy-10,13-Dimethyl-Tetradecahydro-2H-Cyclopenta[A]Phenanthren-17(14H)-One | ESR1 |
| Astragalus mongholicus | MOL794 | (5S,8R,9S,10S,13S,14S)-3-Ethyl-3-Hydroxy-10,13-Dimethyl-Tetradecahydro-2H-Cyclopenta[A]Phenanthren-17(14H)-One | ESR2 |
| Astragalus mongholicus | MOL795 | (5S,8R,9S,10S,13S,14S)-3-Ethyl-3-Hydroxy-10,13-Dimethyl-Tetradecahydro-2H-Cyclopenta[A]Phenanthren-17(14H)-One | ESRRG |
| Astragalus mongholicus | MOL796 | (5S,8R,9S,10S,13S,14S)-3-Ethyl-3-Hydroxy-10,13-Dimethyl-Tetradecahydro-2H-Cyclopenta[A]Phenanthren-17(14H)-One | FABP6 |
| Astragalus mongholicus | MOL797 | (5S,8R,9S,10S,13S,14S)-3-Ethyl-3-Hydroxy-10,13-Dimethyl-Tetradecahydro-2H-Cyclopenta[A]Phenanthren-17(14H)-One | FECH |
| Astragalus mongholicus | MOL798 | (5S,8R,9S,10S,13S,14S)-3-Ethyl-3-Hydroxy-10,13-Dimethyl-Tetradecahydro-2H-Cyclopenta[A]Phenanthren-17(14H)-One | G6PD |
| Astragalus mongholicus | MOL799 | (5S,8R,9S,10S,13S,14S)-3-Ethyl-3-Hydroxy-10,13-Dimethyl-Tetradecahydro-2H-Cyclopenta[A]Phenanthren-17(14H)-One | GABRA1 |
| Astragalus mongholicus | MOL800 | (5S,8R,9S,10S,13S,14S)-3-Ethyl-3-Hydroxy-10,13-Dimethyl-Tetradecahydro-2H-Cyclopenta[A]Phenanthren-17(14H)-One | GABRA2 |
| Astragalus mongholicus | MOL801 | (5S,8R,9S,10S,13S,14S)-3-Ethyl-3-Hydroxy-10,13-Dimethyl-Tetradecahydro-2H-Cyclopenta[A]Phenanthren-17(14H)-One | GABRA3 |
| Astragalus mongholicus | MOL802 | (5S,8R,9S,10S,13S,14S)-3-Ethyl-3-Hydroxy-10,13-Dimethyl-Tetradecahydro-2H-Cyclopenta[A]Phenanthren-17(14H)-One | GABRA4 |
| Astragalus mongholicus | MOL803 | (5S,8R,9S,10S,13S,14S)-3-Ethyl-3-Hydroxy-10,13-Dimethyl-Tetradecahydro-2H-Cyclopenta[A]Phenanthren-17(14H)-One | GABRA5 |
| Astragalus mongholicus | MOL804 | (5S,8R,9S,10S,13S,14S)-3-Ethyl-3-Hydroxy-10,13-Dimethyl-Tetradecahydro-2H-Cyclopenta[A]Phenanthren-17(14H)-One | GABRA6 |
| Astragalus mongholicus | MOL805 | (5S,8R,9S,10S,13S,14S)-3-Ethyl-3-Hydroxy-10,13-Dimethyl-Tetradecahydro-2H-Cyclopenta[A]Phenanthren-17(14H)-One | GABRB1 |
| Astragalus mongholicus | MOL806 | (5S,8R,9S,10S,13S,14S)-3-Ethyl-3-Hydroxy-10,13-Dimethyl-Tetradecahydro-2H-Cyclopenta[A]Phenanthren-17(14H)-One | GABRB2 |
| Astragalus mongholicus | MOL807 | (5S,8R,9S,10S,13S,14S)-3-Ethyl-3-Hydroxy-10,13-Dimethyl-Tetradecahydro-2H-Cyclopenta[A]Phenanthren-17(14H)-One | GABRB3 |
| Astragalus mongholicus | MOL808 | (5S,8R,9S,10S,13S,14S)-3-Ethyl-3-Hydroxy-10,13-Dimethyl-Tetradecahydro-2H-Cyclopenta[A]Phenanthren-17(14H)-One | GABRD |
| Astragalus mongholicus | MOL809 | (5S,8R,9S,10S,13S,14S)-3-Ethyl-3-Hydroxy-10,13-Dimethyl-Tetradecahydro-2H-Cyclopenta[A]Phenanthren-17(14H)-One | GABRE |
| Astragalus mongholicus | MOL810 | (5S,8R,9S,10S,13S,14S)-3-Ethyl-3-Hydroxy-10,13-Dimethyl-Tetradecahydro-2H-Cyclopenta[A]Phenanthren-17(14H)-One | GABRG1 |
| Astragalus mongholicus | MOL811 | (5S,8R,9S,10S,13S,14S)-3-Ethyl-3-Hydroxy-10,13-Dimethyl-Tetradecahydro-2H-Cyclopenta[A]Phenanthren-17(14H)-One | GABRG2 |
| Astragalus mongholicus | MOL812 | (5S,8R,9S,10S,13S,14S)-3-Ethyl-3-Hydroxy-10,13-Dimethyl-Tetradecahydro-2H-Cyclopenta[A]Phenanthren-17(14H)-One | GABRG3 |
| Astragalus mongholicus | MOL813 | (5S,8R,9S,10S,13S,14S)-3-Ethyl-3-Hydroxy-10,13-Dimethyl-Tetradecahydro-2H-Cyclopenta[A]Phenanthren-17(14H)-One | GABRP |
| Astragalus mongholicus | MOL814 | (5S,8R,9S,10S,13S,14S)-3-Ethyl-3-Hydroxy-10,13-Dimethyl-Tetradecahydro-2H-Cyclopenta[A]Phenanthren-17(14H)-One | GABRQ |
| Astragalus mongholicus | MOL815 | (5S,8R,9S,10S,13S,14S)-3-Ethyl-3-Hydroxy-10,13-Dimethyl-Tetradecahydro-2H-Cyclopenta[A]Phenanthren-17(14H)-One | GPBAR1 |
| Astragalus mongholicus | MOL816 | (5S,8R,9S,10S,13S,14S)-3-Ethyl-3-Hydroxy-10,13-Dimethyl-Tetradecahydro-2H-Cyclopenta[A]Phenanthren-17(14H)-One | GSTP1 |
| Astragalus mongholicus | MOL817 | (5S,8R,9S,10S,13S,14S)-3-Ethyl-3-Hydroxy-10,13-Dimethyl-Tetradecahydro-2H-Cyclopenta[A]Phenanthren-17(14H)-One | HSD17B1 |
| Astragalus mongholicus | MOL818 | (5S,8R,9S,10S,13S,14S)-3-Ethyl-3-Hydroxy-10,13-Dimethyl-Tetradecahydro-2H-Cyclopenta[A]Phenanthren-17(14H)-One | HSD17B11 |
| Astragalus mongholicus | MOL819 | (5S,8R,9S,10S,13S,14S)-3-Ethyl-3-Hydroxy-10,13-Dimethyl-Tetradecahydro-2H-Cyclopenta[A]Phenanthren-17(14H)-One | IGHG2 |
| Astragalus mongholicus | MOL820 | (5S,8R,9S,10S,13S,14S)-3-Ethyl-3-Hydroxy-10,13-Dimethyl-Tetradecahydro-2H-Cyclopenta[A]Phenanthren-17(14H)-One | MT-CO1 |
| Astragalus mongholicus | MOL821 | (5S,8R,9S,10S,13S,14S)-3-Ethyl-3-Hydroxy-10,13-Dimethyl-Tetradecahydro-2H-Cyclopenta[A]Phenanthren-17(14H)-One | MT-CO2 |
| Astragalus mongholicus | MOL822 | (5S,8R,9S,10S,13S,14S)-3-Ethyl-3-Hydroxy-10,13-Dimethyl-Tetradecahydro-2H-Cyclopenta[A]Phenanthren-17(14H)-One | MT-CO3 |
| Astragalus mongholicus | MOL823 | (5S,8R,9S,10S,13S,14S)-3-Ethyl-3-Hydroxy-10,13-Dimethyl-Tetradecahydro-2H-Cyclopenta[A]Phenanthren-17(14H)-One | NCOA2 |
| Astragalus mongholicus | MOL824 | (5S,8R,9S,10S,13S,14S)-3-Ethyl-3-Hydroxy-10,13-Dimethyl-Tetradecahydro-2H-Cyclopenta[A]Phenanthren-17(14H)-One | NR1H4 |
| Astragalus mongholicus | MOL825 | (5S,8R,9S,10S,13S,14S)-3-Ethyl-3-Hydroxy-10,13-Dimethyl-Tetradecahydro-2H-Cyclopenta[A]Phenanthren-17(14H)-One | NR1I2 |
| Astragalus mongholicus | MOL826 | (5S,8R,9S,10S,13S,14S)-3-Ethyl-3-Hydroxy-10,13-Dimethyl-Tetradecahydro-2H-Cyclopenta[A]Phenanthren-17(14H)-One | NR3C1 |
| Astragalus mongholicus | MOL827 | (5S,8R,9S,10S,13S,14S)-3-Ethyl-3-Hydroxy-10,13-Dimethyl-Tetradecahydro-2H-Cyclopenta[A]Phenanthren-17(14H)-One | NR3C2 |
| Astragalus mongholicus | MOL828 | (5S,8R,9S,10S,13S,14S)-3-Ethyl-3-Hydroxy-10,13-Dimethyl-Tetradecahydro-2H-Cyclopenta[A]Phenanthren-17(14H)-One | PGR |
| Astragalus mongholicus | MOL829 | (5S,8R,9S,10S,13S,14S)-3-Ethyl-3-Hydroxy-10,13-Dimethyl-Tetradecahydro-2H-Cyclopenta[A]Phenanthren-17(14H)-One | PLA2G1B |
| Astragalus mongholicus | MOL830 | (5S,8R,9S,10S,13S,14S)-3-Ethyl-3-Hydroxy-10,13-Dimethyl-Tetradecahydro-2H-Cyclopenta[A]Phenanthren-17(14H)-One | SULT2A1 |
| Astragalus mongholicus | MOL831 | (5S,8R,9S,10S,13S,14S)-3-Ethyl-3-Hydroxy-10,13-Dimethyl-Tetradecahydro-2H-Cyclopenta[A]Phenanthren-17(14H)-One | SULT2B1 |
| Astragalus mongholicus | MOL832 | (1S,4S,4Ar,8Ar)-4-Isopropyl-1,6-Dimethyl-1,2,3,4,4A,7,8,8A-Octahydronaphthalen-1-Ol | AKR1C1 |
| Astragalus mongholicus | MOL833 | (1S,4S,4Ar,8Ar)-4-Isopropyl-1,6-Dimethyl-1,2,3,4,4A,7,8,8A-Octahydronaphthalen-1-Ol | AKR1C2 |
| Astragalus mongholicus | MOL834 | (1S,4S,4Ar,8Ar)-4-Isopropyl-1,6-Dimethyl-1,2,3,4,4A,7,8,8A-Octahydronaphthalen-1-Ol | AR |
| Astragalus mongholicus | MOL835 | (1S,4S,4Ar,8Ar)-4-Isopropyl-1,6-Dimethyl-1,2,3,4,4A,7,8,8A-Octahydronaphthalen-1-Ol | CLEC4E |
| Astragalus mongholicus | MOL836 | (1S,4S,4Ar,8Ar)-4-Isopropyl-1,6-Dimethyl-1,2,3,4,4A,7,8,8A-Octahydronaphthalen-1-Ol | ESR1 |
| Astragalus mongholicus | MOL837 | (1S,4S,4Ar,8Ar)-4-Isopropyl-1,6-Dimethyl-1,2,3,4,4A,7,8,8A-Octahydronaphthalen-1-Ol | LSS |
| Astragalus mongholicus | MOL838 | (1S,4S,4Ar,8Ar)-4-Isopropyl-1,6-Dimethyl-1,2,3,4,4A,7,8,8A-Octahydronaphthalen-1-Ol | NCOA2 |
| Astragalus mongholicus | MOL839 | (1S,4S,4Ar,8Ar)-4-Isopropyl-1,6-Dimethyl-1,2,3,4,4A,7,8,8A-Octahydronaphthalen-1-Ol | NR1I2 |
| Astragalus mongholicus | MOL840 | (1S,4S,4Ar,8Ar)-4-Isopropyl-1,6-Dimethyl-1,2,3,4,4A,7,8,8A-Octahydronaphthalen-1-Ol | NR1I3 |
| Astragalus mongholicus | MOL841 | (1S,4S,4Ar,8Ar)-4-Isopropyl-1,6-Dimethyl-1,2,3,4,4A,7,8,8A-Octahydronaphthalen-1-Ol | NR3C2 |
| Astragalus mongholicus | MOL842 | (1S,4S,4Ar,8Ar)-4-Isopropyl-1,6-Dimethyl-1,2,3,4,4A,7,8,8A-Octahydronaphthalen-1-Ol | PGR |
| Astragalus mongholicus | MOL843 | (1S,4S,4Ar,8Ar)-4-Isopropyl-1,6-Dimethyl-1,2,3,4,4A,7,8,8A-Octahydronaphthalen-1-Ol | RORA |
| Astragalus mongholicus | MOL844 | (1S,4S,4Ar,8Ar)-4-Isopropyl-1,6-Dimethyl-1,2,3,4,4A,7,8,8A-Octahydronaphthalen-1-Ol | SULT2B1 |
| Astragalus mongholicus | MOL845 | (1S,4S,4Ar,8Ar)-4-Isopropyl-1,6-Dimethyl-1,2,3,4,4A,7,8,8A-Octahydronaphthalen-1-Ol | VDR |
| Astragalus mongholicus | MOL846 | (1S,4R,4Ar,8Ar)-1-Isopropyl-4,7-Dimethyl-1,2,3,4,4A,5,6,8A-Octahydronaphthalen-4A-Ol | AKR1C1 |
| Astragalus mongholicus | MOL847 | (1S,4R,4Ar,8Ar)-1-Isopropyl-4,7-Dimethyl-1,2,3,4,4A,5,6,8A-Octahydronaphthalen-4A-Ol | AKR1C2 |
| Astragalus mongholicus | MOL848 | (1S,4R,4Ar,8Ar)-1-Isopropyl-4,7-Dimethyl-1,2,3,4,4A,5,6,8A-Octahydronaphthalen-4A-Ol | AR |
| Astragalus mongholicus | MOL849 | (1S,4R,4Ar,8Ar)-1-Isopropyl-4,7-Dimethyl-1,2,3,4,4A,5,6,8A-Octahydronaphthalen-4A-Ol | CLEC4E |
| Astragalus mongholicus | MOL850 | (1S,4R,4Ar,8Ar)-1-Isopropyl-4,7-Dimethyl-1,2,3,4,4A,5,6,8A-Octahydronaphthalen-4A-Ol | ESR1 |
| Astragalus mongholicus | MOL851 | (1S,4R,4Ar,8Ar)-1-Isopropyl-4,7-Dimethyl-1,2,3,4,4A,5,6,8A-Octahydronaphthalen-4A-Ol | ESR2 |
| Astragalus mongholicus | MOL852 | (1S,4R,4Ar,8Ar)-1-Isopropyl-4,7-Dimethyl-1,2,3,4,4A,5,6,8A-Octahydronaphthalen-4A-Ol | GABRA1 |
| Astragalus mongholicus | MOL853 | (1S,4R,4Ar,8Ar)-1-Isopropyl-4,7-Dimethyl-1,2,3,4,4A,5,6,8A-Octahydronaphthalen-4A-Ol | GABRA2 |
| Astragalus mongholicus | MOL854 | (1S,4R,4Ar,8Ar)-1-Isopropyl-4,7-Dimethyl-1,2,3,4,4A,5,6,8A-Octahydronaphthalen-4A-Ol | GABRA3 |
| Astragalus mongholicus | MOL855 | (1S,4R,4Ar,8Ar)-1-Isopropyl-4,7-Dimethyl-1,2,3,4,4A,5,6,8A-Octahydronaphthalen-4A-Ol | GABRA4 |
| Astragalus mongholicus | MOL856 | (1S,4R,4Ar,8Ar)-1-Isopropyl-4,7-Dimethyl-1,2,3,4,4A,5,6,8A-Octahydronaphthalen-4A-Ol | GABRA5 |
| Astragalus mongholicus | MOL857 | (1S,4R,4Ar,8Ar)-1-Isopropyl-4,7-Dimethyl-1,2,3,4,4A,5,6,8A-Octahydronaphthalen-4A-Ol | GABRA6 |
| Astragalus mongholicus | MOL858 | (1S,4R,4Ar,8Ar)-1-Isopropyl-4,7-Dimethyl-1,2,3,4,4A,5,6,8A-Octahydronaphthalen-4A-Ol | GABRB1 |
| Astragalus mongholicus | MOL859 | (1S,4R,4Ar,8Ar)-1-Isopropyl-4,7-Dimethyl-1,2,3,4,4A,5,6,8A-Octahydronaphthalen-4A-Ol | GABRB2 |
| Astragalus mongholicus | MOL860 | (1S,4R,4Ar,8Ar)-1-Isopropyl-4,7-Dimethyl-1,2,3,4,4A,5,6,8A-Octahydronaphthalen-4A-Ol | GABRB3 |
| Astragalus mongholicus | MOL861 | (1S,4R,4Ar,8Ar)-1-Isopropyl-4,7-Dimethyl-1,2,3,4,4A,5,6,8A-Octahydronaphthalen-4A-Ol | GABRD |
| Astragalus mongholicus | MOL862 | (1S,4R,4Ar,8Ar)-1-Isopropyl-4,7-Dimethyl-1,2,3,4,4A,5,6,8A-Octahydronaphthalen-4A-Ol | GABRE |
| Astragalus mongholicus | MOL863 | (1S,4R,4Ar,8Ar)-1-Isopropyl-4,7-Dimethyl-1,2,3,4,4A,5,6,8A-Octahydronaphthalen-4A-Ol | GABRG1 |
| Astragalus mongholicus | MOL864 | (1S,4R,4Ar,8Ar)-1-Isopropyl-4,7-Dimethyl-1,2,3,4,4A,5,6,8A-Octahydronaphthalen-4A-Ol | GABRG2 |
| Astragalus mongholicus | MOL865 | (1S,4R,4Ar,8Ar)-1-Isopropyl-4,7-Dimethyl-1,2,3,4,4A,5,6,8A-Octahydronaphthalen-4A-Ol | GABRG3 |
| Astragalus mongholicus | MOL866 | (1S,4R,4Ar,8Ar)-1-Isopropyl-4,7-Dimethyl-1,2,3,4,4A,5,6,8A-Octahydronaphthalen-4A-Ol | GABRP |
| Astragalus mongholicus | MOL867 | (1S,4R,4Ar,8Ar)-1-Isopropyl-4,7-Dimethyl-1,2,3,4,4A,5,6,8A-Octahydronaphthalen-4A-Ol | GABRQ |
| Astragalus mongholicus | MOL868 | (1S,4R,4Ar,8Ar)-1-Isopropyl-4,7-Dimethyl-1,2,3,4,4A,5,6,8A-Octahydronaphthalen-4A-Ol | GRIN1 |
| Astragalus mongholicus | MOL869 | (1S,4R,4Ar,8Ar)-1-Isopropyl-4,7-Dimethyl-1,2,3,4,4A,5,6,8A-Octahydronaphthalen-4A-Ol | GRIN2A |
| Astragalus mongholicus | MOL870 | (1S,4R,4Ar,8Ar)-1-Isopropyl-4,7-Dimethyl-1,2,3,4,4A,5,6,8A-Octahydronaphthalen-4A-Ol | GRIN2B |
| Astragalus mongholicus | MOL871 | (1S,4R,4Ar,8Ar)-1-Isopropyl-4,7-Dimethyl-1,2,3,4,4A,5,6,8A-Octahydronaphthalen-4A-Ol | GRIN2C |
| Astragalus mongholicus | MOL872 | (1S,4R,4Ar,8Ar)-1-Isopropyl-4,7-Dimethyl-1,2,3,4,4A,5,6,8A-Octahydronaphthalen-4A-Ol | GRIN2D |
| Astragalus mongholicus | MOL873 | (1S,4R,4Ar,8Ar)-1-Isopropyl-4,7-Dimethyl-1,2,3,4,4A,5,6,8A-Octahydronaphthalen-4A-Ol | GRIN3A |
| Astragalus mongholicus | MOL874 | (1S,4R,4Ar,8Ar)-1-Isopropyl-4,7-Dimethyl-1,2,3,4,4A,5,6,8A-Octahydronaphthalen-4A-Ol | GRIN3B |
| Astragalus mongholicus | MOL875 | (1S,4R,4Ar,8Ar)-1-Isopropyl-4,7-Dimethyl-1,2,3,4,4A,5,6,8A-Octahydronaphthalen-4A-Ol | HOXA10 |
| Astragalus mongholicus | MOL876 | (1S,4R,4Ar,8Ar)-1-Isopropyl-4,7-Dimethyl-1,2,3,4,4A,5,6,8A-Octahydronaphthalen-4A-Ol | HSD17B1 |
| Astragalus mongholicus | MOL877 | (1S,4R,4Ar,8Ar)-1-Isopropyl-4,7-Dimethyl-1,2,3,4,4A,5,6,8A-Octahydronaphthalen-4A-Ol | LSS |
| Astragalus mongholicus | MOL878 | (1S,4R,4Ar,8Ar)-1-Isopropyl-4,7-Dimethyl-1,2,3,4,4A,5,6,8A-Octahydronaphthalen-4A-Ol | NCOA2 |
| Astragalus mongholicus | MOL879 | (1S,4R,4Ar,8Ar)-1-Isopropyl-4,7-Dimethyl-1,2,3,4,4A,5,6,8A-Octahydronaphthalen-4A-Ol | NPPB |
| Astragalus mongholicus | MOL880 | (1S,4R,4Ar,8Ar)-1-Isopropyl-4,7-Dimethyl-1,2,3,4,4A,5,6,8A-Octahydronaphthalen-4A-Ol | NR1I2 |
| Astragalus mongholicus | MOL881 | (1S,4R,4Ar,8Ar)-1-Isopropyl-4,7-Dimethyl-1,2,3,4,4A,5,6,8A-Octahydronaphthalen-4A-Ol | NR1I3 |
| Astragalus mongholicus | MOL882 | (1S,4R,4Ar,8Ar)-1-Isopropyl-4,7-Dimethyl-1,2,3,4,4A,5,6,8A-Octahydronaphthalen-4A-Ol | NR3C2 |
| Astragalus mongholicus | MOL883 | (1S,4R,4Ar,8Ar)-1-Isopropyl-4,7-Dimethyl-1,2,3,4,4A,5,6,8A-Octahydronaphthalen-4A-Ol | PGR |
| Astragalus mongholicus | MOL884 | (1S,4R,4Ar,8Ar)-1-Isopropyl-4,7-Dimethyl-1,2,3,4,4A,5,6,8A-Octahydronaphthalen-4A-Ol | PPARA |
| Astragalus mongholicus | MOL885 | (1S,4R,4Ar,8Ar)-1-Isopropyl-4,7-Dimethyl-1,2,3,4,4A,5,6,8A-Octahydronaphthalen-4A-Ol | RORA |
| Astragalus mongholicus | MOL886 | (1S,4R,4Ar,8Ar)-1-Isopropyl-4,7-Dimethyl-1,2,3,4,4A,5,6,8A-Octahydronaphthalen-4A-Ol | SIGMAR1 |
| Astragalus mongholicus | MOL887 | (1S,4R,4Ar,8Ar)-1-Isopropyl-4,7-Dimethyl-1,2,3,4,4A,5,6,8A-Octahydronaphthalen-4A-Ol | SULT2A1 |
| Astragalus mongholicus | MOL888 | (1S,4R,4Ar,8Ar)-1-Isopropyl-4,7-Dimethyl-1,2,3,4,4A,5,6,8A-Octahydronaphthalen-4A-Ol | SULT2B1 |
| Astragalus mongholicus | MOL889 | (1S,4R,4Ar,8Ar)-1-Isopropyl-4,7-Dimethyl-1,2,3,4,4A,5,6,8A-Octahydronaphthalen-4A-Ol | VDR |
| Astragalus mongholicus | MOL890 | (1Ar,7S,7As,7Br,Z)-1,1,4,7-Tetramethyl-1A,2,3,5,6,7,7A,7B-Octahydro-1H-Cyclopropa[E]Azulen-7-Ol | CLEC4E |
| Astragalus mongholicus | MOL891 | (1Ar,7S,7As,7Br,Z)-1,1,4,7-Tetramethyl-1A,2,3,5,6,7,7A,7B-Octahydro-1H-Cyclopropa[E]Azulen-7-Ol | LSS |
| Astragalus mongholicus | MOL892 | (1Ar,7S,7As,7Br,Z)-1,1,4,7-Tetramethyl-1A,2,3,5,6,7,7A,7B-Octahydro-1H-Cyclopropa[E]Azulen-7-Ol | NR1I3 |
| Astragalus mongholicus | MOL893 | (1Ar,7S,7As,7Br,Z)-1,1,4,7-Tetramethyl-1A,2,3,5,6,7,7A,7B-Octahydro-1H-Cyclopropa[E]Azulen-7-Ol | RORA |
| Astragalus mongholicus | MOL894 | (1Ar,7S,7As,7Br,Z)-1,1,4,7-Tetramethyl-1A,2,3,5,6,7,7A,7B-Octahydro-1H-Cyclopropa[E]Azulen-7-Ol | VDR |
| Astragalus mongholicus | MOL895 | (3Z,7Z)-1,5,5,8-Tetramethyl-12-Oxa-Bicyclo[9.1.0]Dodeca-3,7-Diene | LCN9 |
| Astragalus mongholicus | MOL896 | (3S,3As,8R,8Ar)-3,8-Dihydroxy-3,8-Dimethyl-5-(Propan-2-Ylidene)-Hexahydroazulen-6(1H,2H,7H)-One | AR |
| Astragalus mongholicus | MOL897 | (3S,3As,8R,8Ar)-3,8-Dihydroxy-3,8-Dimethyl-5-(Propan-2-Ylidene)-Hexahydroazulen-6(1H,2H,7H)-One | ESR1 |
| Astragalus mongholicus | MOL898 | (3S,3As,8R,8Ar)-3,8-Dihydroxy-3,8-Dimethyl-5-(Propan-2-Ylidene)-Hexahydroazulen-6(1H,2H,7H)-One | HOXA10 |
| Astragalus mongholicus | MOL899 | (3S,3As,8R,8Ar)-3,8-Dihydroxy-3,8-Dimethyl-5-(Propan-2-Ylidene)-Hexahydroazulen-6(1H,2H,7H)-One | HSD11B2 |
| Astragalus mongholicus | MOL900 | (3S,3As,8R,8Ar)-3,8-Dihydroxy-3,8-Dimethyl-5-(Propan-2-Ylidene)-Hexahydroazulen-6(1H,2H,7H)-One | NCOA1 |
| Astragalus mongholicus | MOL901 | (3S,3As,8R,8Ar)-3,8-Dihydroxy-3,8-Dimethyl-5-(Propan-2-Ylidene)-Hexahydroazulen-6(1H,2H,7H)-One | NPPB |
| Astragalus mongholicus | MOL902 | (3S,3As,8R,8Ar)-3,8-Dihydroxy-3,8-Dimethyl-5-(Propan-2-Ylidene)-Hexahydroazulen-6(1H,2H,7H)-One | VDR |
| Astragalus mongholicus | MOL903 | (3S,3Ar,8Ar,Z)-3,8A-Dihydroxy-3,8-Dimethyl-5-(Propan-2-Ylidene)-1,2,3,3A,4,5-Hexahydroazulen-6(8Ah)-One | AR |
| Astragalus mongholicus | MOL904 | (3S,3Ar,8Ar,Z)-3,8A-Dihydroxy-3,8-Dimethyl-5-(Propan-2-Ylidene)-1,2,3,3A,4,5-Hexahydroazulen-6(8Ah)-One | CYP27B1 |
| Astragalus mongholicus | MOL905 | (3S,3Ar,8Ar,Z)-3,8A-Dihydroxy-3,8-Dimethyl-5-(Propan-2-Ylidene)-1,2,3,3A,4,5-Hexahydroazulen-6(8Ah)-One | HOXA10 |
| Astragalus mongholicus | MOL906 | (3S,3Ar,8Ar,Z)-3,8A-Dihydroxy-3,8-Dimethyl-5-(Propan-2-Ylidene)-1,2,3,3A,4,5-Hexahydroazulen-6(8Ah)-One | HSD11B2 |
| Astragalus mongholicus | MOL907 | (3S,3Ar,8Ar,Z)-3,8A-Dihydroxy-3,8-Dimethyl-5-(Propan-2-Ylidene)-1,2,3,3A,4,5-Hexahydroazulen-6(8Ah)-One | NCOA1 |
| Astragalus mongholicus | MOL908 | (3S,3Ar,8Ar,Z)-3,8A-Dihydroxy-3,8-Dimethyl-5-(Propan-2-Ylidene)-1,2,3,3A,4,5-Hexahydroazulen-6(8Ah)-One | NPPB |
| Astragalus mongholicus | MOL909 | (3S,3Ar,8Ar,Z)-3,8A-Dihydroxy-3,8-Dimethyl-5-(Propan-2-Ylidene)-1,2,3,3A,4,5-Hexahydroazulen-6(8Ah)-One | RXRA |
| Astragalus mongholicus | MOL910 | (3S,3Ar,8Ar,Z)-3,8A-Dihydroxy-3,8-Dimethyl-5-(Propan-2-Ylidene)-1,2,3,3A,4,5-Hexahydroazulen-6(8Ah)-One | VDR |
| Astragalus mongholicus | MOL911 | (3S,3As,5S,8As)-3A-Hydroxy-3,3',3',8-Tetramethyl-1,2,3,3A,4,8A-Hexahydro-6H-Spiro[Azulene-5,2'-Oxiran]-6-One | ESR1 |
| Astragalus mongholicus | MOL912 | (3S,3As,5S,8As)-3A-Hydroxy-3,3',3',8-Tetramethyl-1,2,3,3A,4,8A-Hexahydro-6H-Spiro[Azulene-5,2'-Oxiran]-6-One | ESR2 |
| Astragalus mongholicus | MOL913 | (3S,3As,5S,8As)-3A-Hydroxy-3,3',3',8-Tetramethyl-1,2,3,3A,4,8A-Hexahydro-6H-Spiro[Azulene-5,2'-Oxiran]-6-One | HSD3B1 |
| Astragalus mongholicus | MOL914 | (3S,3As,5S,8As)-3A-Hydroxy-3,3',3',8-Tetramethyl-1,2,3,3A,4,8A-Hexahydro-6H-Spiro[Azulene-5,2'-Oxiran]-6-One | HSD3B2 |
| Astragalus mongholicus | MOL915 | (4Ar,5R,5As,6Ar)-6A-Hydroxy-3,5A-Dimethyl-5-(3-Oxobutyl)-4,4A,5,5A,6,6A-Hexahydro-2H-Cyclopropa[F][1]Benzofuran-2-One | AR |
| Astragalus mongholicus | MOL916 | (4Ar,5R,5As,6Ar)-6A-Hydroxy-3,5A-Dimethyl-5-(3-Oxobutyl)-4,4A,5,5A,6,6A-Hexahydro-2H-Cyclopropa[F][1]Benzofuran-2-One | IGHG2 |
| Astragalus mongholicus | MOL917 | (4Ar,5R,5As,6Ar)-6A-Hydroxy-3,5A-Dimethyl-5-(3-Oxobutyl)-4,4A,5,5A,6,6A-Hexahydro-2H-Cyclopropa[F][1]Benzofuran-2-One | NR3C2 |
| Astragalus mongholicus | MOL918 | (4Ar,5R,5As,6Ar)-6A-Hydroxy-3,5A-Dimethyl-5-(3-Oxobutyl)-4,4A,5,5A,6,6A-Hexahydro-2H-Cyclopropa[F][1]Benzofuran-2-One | PGR |
| Astragalus mongholicus | MOL919 | (4Ar,5R,5As,6As)-3,5A-Dimethyl-5-(3-Oxobutyl)-4,4A,5,5A,6,6A-Hexahydro-2H-Cyclopropa[F][1]Benzofuran-2-One | AR |
| Astragalus mongholicus | MOL920 | (4Ar,5R,5As,6As)-3,5A-Dimethyl-5-(3-Oxobutyl)-4,4A,5,5A,6,6A-Hexahydro-2H-Cyclopropa[F][1]Benzofuran-2-One | ESR1 |
| Astragalus mongholicus | MOL921 | (4Ar,5R,5As,6As)-3,5A-Dimethyl-5-(3-Oxobutyl)-4,4A,5,5A,6,6A-Hexahydro-2H-Cyclopropa[F][1]Benzofuran-2-One | NR3C2 |
| Astragalus mongholicus | MOL922 | (4Ar,5R,5As,6As)-3,5A-Dimethyl-5-(3-Oxobutyl)-4,4A,5,5A,6,6A-Hexahydro-2H-Cyclopropa[F][1]Benzofuran-2-One | PGR |

**Table 4b. LA of the active ingredients from ETCM database.**

| Drug | MolId | MolName | Symbol |
| --- | --- | --- | --- |
| Astragalus mongholicus | MOL01 | Î‘-Curcumene | COMT |
| Astragalus mongholicus | MOL02 | Î‘-Curcumene | GABRA1 |
| Astragalus mongholicus | MOL03 | Î‘-Curcumene | GABRA2 |
| Astragalus mongholicus | MOL04 | Î‘-Curcumene | GABRA3 |
| Astragalus mongholicus | MOL05 | Î‘-Curcumene | GABRA4 |
| Astragalus mongholicus | MOL06 | Î‘-Curcumene | GABRA5 |
| Astragalus mongholicus | MOL07 | Î‘-Curcumene | GABRA6 |
| Astragalus mongholicus | MOL08 | Î‘-Curcumene | GABRG1 |
| Astragalus mongholicus | MOL09 | Î‘-Curcumene | GABRG2 |
| Astragalus mongholicus | MOL10 | Î‘-Curcumene | GABRG3 |
| Astragalus mongholicus | MOL11 | Î‘-Curcumene | IGHG1 |
| Astragalus mongholicus | MOL12 | Î‘-Curcumene | MTTP |
| Astragalus mongholicus | MOL13 | Î‘-Curcumene | SHBG |
| Astragalus mongholicus | MOL14 | Î‘-Curcumene | SOAT1 |
| Astragalus mongholicus | MOL15 | Î‘-Curcumene | SOAT2 |
| Astragalus mongholicus | MOL16 | Pavilion | NR1I3 |
| Astragalus mongholicus | MOL17 | Pavilion | VDR |
| Astragalus mongholicus | MOL18 | Cedrol | ADH1B |
| Astragalus mongholicus | MOL19 | Cedrol | AHSP |
| Astragalus mongholicus | MOL20 | Cedrol | ARF1 |
| Astragalus mongholicus | MOL21 | Cedrol | CP |
| Astragalus mongholicus | MOL22 | Cedrol | CYB5A |
| Astragalus mongholicus | MOL23 | Cedrol | EGLN1 |
| Astragalus mongholicus | MOL24 | Cedrol | FEN1 |
| Astragalus mongholicus | MOL25 | Cedrol | FTH1 |
| Astragalus mongholicus | MOL26 | Cedrol | FXN |
| Astragalus mongholicus | MOL27 | Cedrol | GSTP1 |
| Astragalus mongholicus | MOL28 | Cedrol | HBA1 |
| Astragalus mongholicus | MOL29 | Cedrol | HDAC8 |
| Astragalus mongholicus | MOL30 | Cedrol | HPGDS |
| Astragalus mongholicus | MOL31 | Cedrol | ISYNA1 |
| Astragalus mongholicus | MOL32 | Cedrol | ITPR1 |
| Astragalus mongholicus | MOL33 | Cedrol | NAGA |
| Astragalus mongholicus | MOL34 | Cedrol | NEIL1 |
| Astragalus mongholicus | MOL35 | Cedrol | NEIL2 |
| Astragalus mongholicus | MOL36 | Cedrol | PAEP |
| Astragalus mongholicus | MOL37 | Cedrol | PAPSS1 |
| Astragalus mongholicus | MOL38 | Cedrol | PLA2G2E |
| Astragalus mongholicus | MOL39 | Cedrol | POLB |
| Astragalus mongholicus | MOL40 | Cedrol | PPARD |
| Astragalus mongholicus | MOL41 | Cedrol | TF |
| Astragalus mongholicus | MOL42 | Cedrol | TFRC |
| Astragalus mongholicus | MOL43 | Cedrol | TGFBR2 |
| Astragalus mongholicus | MOL44 | Cedrol | TRDMT1 |
| Astragalus mongholicus | MOL45 | Cedrol | AADACL2 |
| Astragalus mongholicus | MOL46 | Cedrol | AKR1C1 |
| Astragalus mongholicus | MOL47 | Cedrol | AKR1C2 |
| Astragalus mongholicus | MOL48 | Cedrol | AR |
| Astragalus mongholicus | MOL49 | Cedrol | ESR1 |
| Astragalus mongholicus | MOL50 | Cedrol | ESR2 |
| Astragalus mongholicus | MOL51 | Cedrol | GABRA1 |
| Astragalus mongholicus | MOL52 | Cedrol | GABRA2 |
| Astragalus mongholicus | MOL53 | Cedrol | GABRA3 |
| Astragalus mongholicus | MOL54 | Cedrol | GABRA4 |
| Astragalus mongholicus | MOL55 | Cedrol | GABRA5 |
| Astragalus mongholicus | MOL56 | Cedrol | GABRA6 |
| Astragalus mongholicus | MOL57 | Cedrol | GABRB1 |
| Astragalus mongholicus | MOL58 | Fructose | GABRB2 |
| Astragalus mongholicus | MOL59 | Fructose | GABRB3 |
| Astragalus mongholicus | MOL60 | Fructose | GABRD |
| Astragalus mongholicus | MOL61 | Fructose | GABRE |
| Astragalus mongholicus | MOL62 | Fructose | GABRG1 |
| Astragalus mongholicus | MOL63 | Fructose | GABRG2 |
| Astragalus mongholicus | MOL64 | Fructose | GABRG3 |
| Astragalus mongholicus | MOL65 | Fructose | GABRP |
| Astragalus mongholicus | MOL66 | Fructose | GABRQ |
| Astragalus mongholicus | MOL67 | Fructose | GRIN1 |
| Astragalus mongholicus | MOL68 | Fructose | GRIN2A |
| Astragalus mongholicus | MOL69 | Fructose | GRIN2B |
| Astragalus mongholicus | MOL70 | Fructose | GRIN2C |
| Astragalus mongholicus | MOL71 | Fructose | GRIN2D |
| Astragalus mongholicus | MOL72 | Fructose | GRIN3A |
| Astragalus mongholicus | MOL73 | Fructose | GRIN3B |
| Astragalus mongholicus | MOL74 | Fructose | HSD11B2 |
| Astragalus mongholicus | MOL75 | Fructose | HSD17B1 |
| Astragalus mongholicus | MOL76 | Fructose | IL1B |
| Astragalus mongholicus | MOL77 | Fructose | IL6 |
| Astragalus mongholicus | MOL78 | Fructose | NCOA2 |
| Astragalus mongholicus | MOL79 | Fructose | NFKB1 |
| Astragalus mongholicus | MOL80 | Fructose | NFKB2 |
| Astragalus mongholicus | MOL81 | Fructose | NPPB(0.814) |
| Astragalus mongholicus | MOL82 | Fructose | NR1I2 |
| Astragalus mongholicus | MOL83 | Fructose | NR1I3 |
| Astragalus mongholicus | MOL84 | Fructose | NR3C2 |
| Astragalus mongholicus | MOL85 | Fructose | PGR |
| Astragalus mongholicus | MOL86 | Fructose | PPARA |
| Astragalus mongholicus | MOL87 | Fructose | SIGMAR1 |
| Astragalus mongholicus | MOL88 | Fructose | SULT2A1 |
| Astragalus mongholicus | MOL89 | Fructose | SULT2B1 |
| Astragalus mongholicus | MOL90 | Fructose | TNF(0.8) |
| Astragalus mongholicus | MOL91 | Î’-Elemol | COMT |
| Astragalus mongholicus | MOL92 | Î’-Elemol | GABRA1 |
| Astragalus mongholicus | MOL93 | Î’-Elemol | GABRA2 |
| Astragalus mongholicus | MOL94 | Î’-Elemol | GABRA3 |
| Astragalus mongholicus | MOL95 | Î’-Elemol | GABRA4 |
| Astragalus mongholicus | MOL96 | Î’-Elemol | GABRA5 |
| Astragalus mongholicus | MOL97 | Î’-Elemol | GABRA6 |
| Astragalus mongholicus | MOL98 | Î’-Elemol | GABRG1 |
| Astragalus mongholicus | MOL99 | Î’-Elemol | GABRG2 |
| Astragalus mongholicus | MOL100 | Î’-Elemol | GABRG3 |
| Astragalus mongholicus | MOL101 | Î’-Elemol | IGHG1 |
| Astragalus mongholicus | MOL102 | Î’-Elemol | MTTP |
| Astragalus mongholicus | MOL103 | Î’-Elemol | SHBG |
| Astragalus mongholicus | MOL104 | Î’-Elemol | SOAT1 |
| Astragalus mongholicus | MOL105 | Î’-Elemol | SOAT2 |
| Astragalus mongholicus | MOL106 | 6-Methoxy-7-Hydroxycoumarin | AR |
| Astragalus mongholicus | MOL107 | 6-Methoxy-7-Hydroxycoumarin | CYP19A1 |
| Astragalus mongholicus | MOL108 | 6-Methoxy-7-Hydroxycoumarin | NR3C2 |
| Astragalus mongholicus | MOL109 | 6-Methoxy-7-Hydroxycoumarin | PGR |
| Astragalus mongholicus | MOL110 | Atractylone | AR |
| Astragalus mongholicus | MOL111 | Atractylone | ESR1 |
| Astragalus mongholicus | MOL112 | Atractylone | NR3C2 |
| Astragalus mongholicus | MOL113 | 8Î’-EthoxyAtractylenolideIii | AR |
| Astragalus mongholicus | MOL114 | 8Î’-EthoxyAtractylenolideIii | CYP17A1 |
| Astragalus mongholicus | MOL115 | 8Î’-EthoxyAtractylenolideIii | CYP19A1 |
| Astragalus mongholicus | MOL116 | 8Î’-EthoxyAtractylenolideIii | ESR1 |
| Astragalus mongholicus | MOL117 | 8Î’-EthoxyAtractylenolideIii | ESR2 |
| Astragalus mongholicus | MOL118 | 8Î’-EthoxyAtractylenolideIii | NR3C1 |
| Astragalus mongholicus | MOL119 | 8Î’-EthoxyAtractylenolideIii | NR3C2 |
| Astragalus mongholicus | MOL120 | 8Î’-EthoxyAtractylenolideIii | OPRK1 |
| Astragalus mongholicus | MOL121 | 8Î’-EthoxyAtractylenolideIii | ORM1 |
| Astragalus mongholicus | MOL122 | 8Î’-EthoxyAtractylenolideIii | PGR |
| Astragalus mongholicus | MOL123 | 8Î’-EthoxyAtractylenolideIii | SHBG |
| Astragalus mongholicus | MOL124 | 8Î’-EthoxyAtractylenolideIii | VDR |
| Astragalus mongholicus | MOL125 | (+)-Eudesma-4(15) | AKR1C1 |
| Astragalus mongholicus | MOL126 | (+)-Eudesma-4(15) | AKR1C2 |
| Astragalus mongholicus | MOL127 | (+)-Eudesma-4(15) | AR |
| Astragalus mongholicus | MOL128 | (+)-Eudesma-4(15) | CLEC4E |
| Astragalus mongholicus | MOL129 | (+)-Eudesma-4(15) | ESR1 |
| Astragalus mongholicus | MOL130 | (+)-Eudesma-4(15) | LSS |
| Astragalus mongholicus | MOL131 | (+)-Eudesma-4(15) | NCOA2 |
| Astragalus mongholicus | MOL132 | (+)-Eudesma-4(15) | NR1I2 |
| Astragalus mongholicus | MOL133 | (+)-Eudesma-4(15) | NR1I3 |
| Astragalus mongholicus | MOL134 | (+)-Eudesma-4(15) | NR3C2 |
| Astragalus mongholicus | MOL135 | (+)-Eudesma-4(15) | PGR |
| Astragalus mongholicus | MOL136 | (+)-Eudesma-4(15) | RORA |
| Astragalus mongholicus | MOL137 | (+)-Eudesma-4(15) | SULT2B1 |
| Astragalus mongholicus | MOL138 | (+)-Eudesma-4(15) | VDR |
