# Supplementary material for: Mechanism of drug-pairs Astragalus Mongholicus–Largehead Atractylodes on treating knee osteoarthritis investigated by GEO gene chip with network pharmacology and molecular docking
Source: Medicine (Baltimore). 2024 Jul 5;103(27):e38699. doi: 10.1097/MD.0000000000038699 (PMC11224889; doi:10.1097/MD.0000000000038699)
Supplement: Supplementary file 5 [file medi-103-e38699-s005.doc]

# Appendix 5

**Core active components**

**Table S5. Drug pair of AM-LA and KOA of core active components**

| PubChem ID | NAME | OB | DL | Source | Structure |
| --- | --- | --- | --- | --- | --- |
| 5281654 | isorhamnetin | 49.60 | 0.31 | TCMSP | 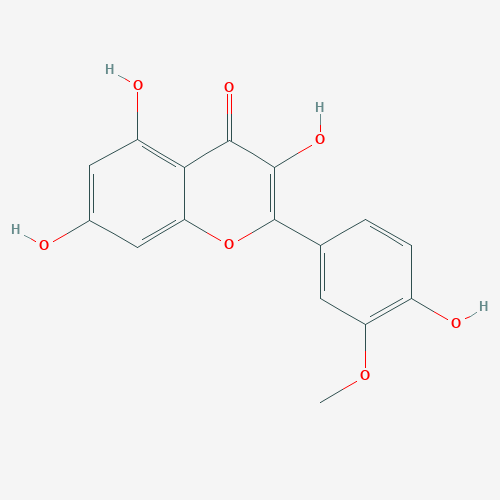 |
| 15689652 | 7-O-methylisomucronulatol | 74.69 | 0.30 | TCMSP | 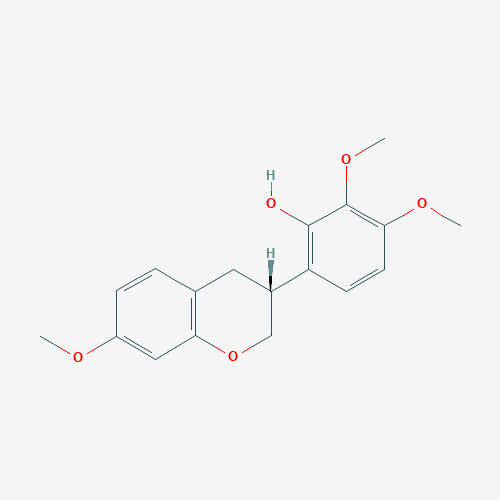 |
| 5280378 | formononetin | 69.67 | 0.21 | TCMSP | 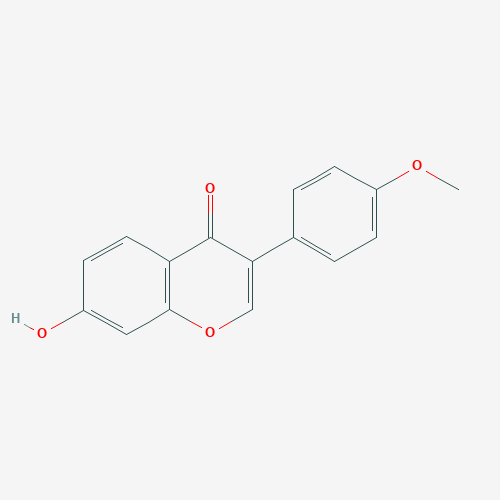 |
| 442811 | Mucronulatol | NA | NA | ETCM | 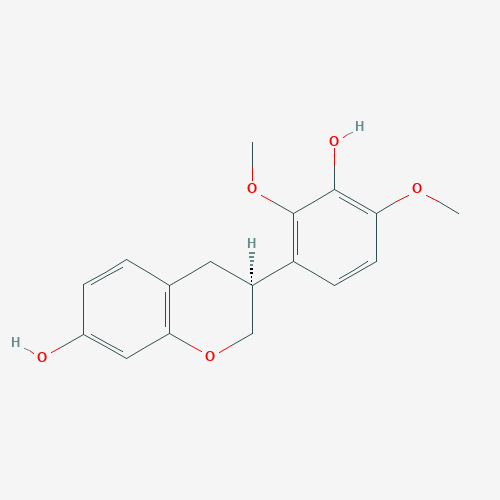 |
| 5280448 | Calycosin | 47.75 | 0.24 | TCMSP | 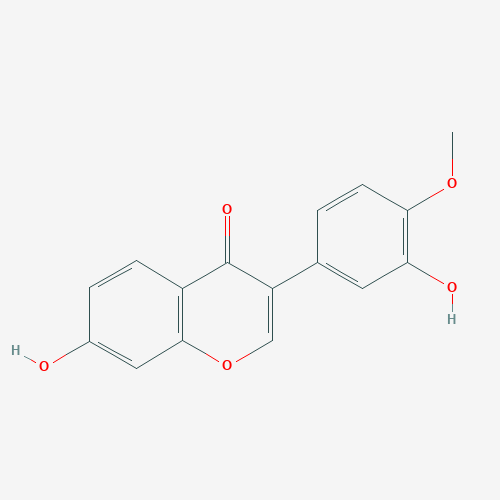 |
| 5280863 | kaempferol | 41.88 | 0.24 | TCMSP | 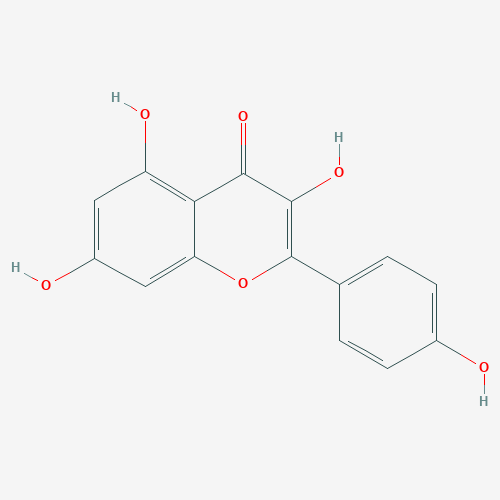 |
| 5280343 | quercetin | 46.43 | 0.28 | TCMSP | 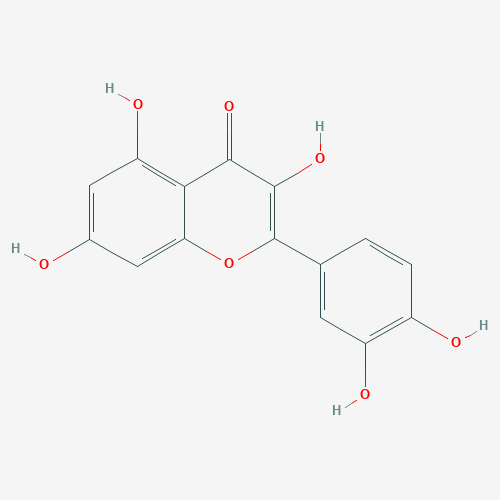 |
| 3917 | (S)-5,7-Dihydroxy-2-Phenylchroman-4-One,Pinocembrin | NA | NA | TCMID | 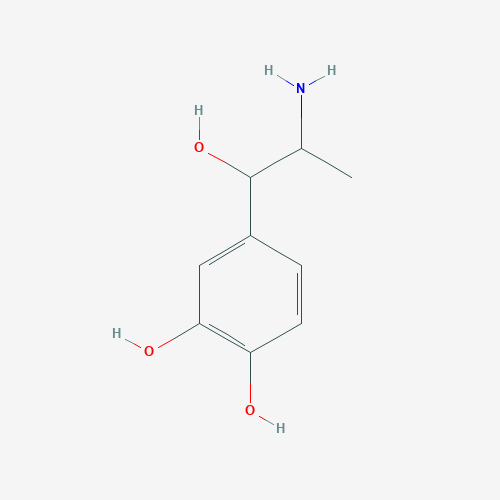 |
| 2723872 | Fructose | NA | NA | ETCM | 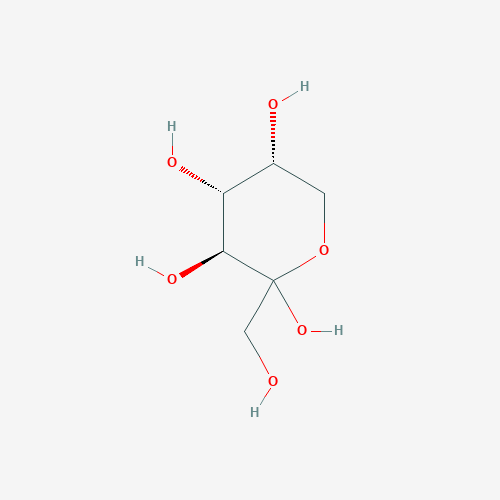 |
