# Supplementary material for: Mechanism of drug-pairs Astragalus Mongholicus–Largehead Atractylodes on treating knee osteoarthritis investigated by GEO gene chip with network pharmacology and molecular docking
Source: Medicine (Baltimore). 2024 Jul 5;103(27):e38699. doi: 10.1097/MD.0000000000038699 (PMC11224889; doi:10.1097/MD.0000000000038699)
Supplement: Supplementary file 6 [file medi-103-e38699-s006.doc]

# Appendix 6

**296 composite targets of drug pair of AM-LA and KOA**

**Table S6. 296 composite targets of drug pair of AM-LA and KOA.**

| SUID | Gene symbol | Betweenness | Closeness | Degree |
| --- | --- | --- | --- | --- |
| 4403 | MAPK1 | 5164.883391 | 0.032030402 | 51 |
| 4161 | HSP90AA1 | 6705.524991 | 0.032026924 | 49 |
| 4140 | TP53 | 8612.153488 | 0.032058248 | 48 |
| 4165 | AKT1 | 5063.980775 | 0.032013022 | 47 |
| 4169 | JUN | 4384.530704 | 0.0320478 | 43 |
| 4167 | RELA | 2392.677463 | 0.031929863 | 39 |
| 4367 | HDAC1 | 5956.748023 | 0.031929863 | 37 |
| 4266 | MAPK14 | 2805.108783 | 0.031881552 | 36 |
| 4334 | RXRA | 4894.468978 | 0.031912592 | 32 |
| 4200 | ESR1 | 3803.659632 | 0.031999132 | 29 |
| 4358 | EGFR | 3060.75442 | 0.031884998 | 29 |
| 4440 | FOS | 2313.016126 | 0.031916045 | 29 |
| 4550 | IL6 | 1572.791628 | 0.031652361 | 29 |
| 4146 | MAPK8 | 1468.399463 | 0.031836823 | 28 |
| 4342 | MYC | 2069.562541 | 0.031957534 | 28 |
| 4374 | NCOA1 | 2074.958931 | 0.031696572 | 26 |
| 4351 | CCND1 | 879.1606826 | 0.031823085 | 25 |
| 4593 | JAK2 | 1142.824746 | 0.031615047 | 25 |
| 4196 | STAT1 | 1492.395144 | 0.031878107 | 24 |
| 4198 | CYP1A1 | 5819.029085 | 0.031527199 | 24 |
| 4327 | CDKN1A | 539.9867076 | 0.03177852 | 23 |
| 4338 | HIF1A | 4155.284533 | 0.03196446 | 23 |
| 4142 | CYP3A4 | 2283.581778 | 0.031223539 | 22 |
| 4163 | PRKCA | 1782.66167 | 0.03177852 | 22 |
| 4262 | NFKB1 | 530.7930206 | 0.031679553 | 22 |
| 4276 | RB1 | 1028.528204 | 0.031884998 | 22 |
| 4302 | JAK1 | 939.824664 | 0.031621824 | 22 |
| 4149 | NR3C1 | 452.9966497 | 0.031874662 | 21 |
| 4144 | CAV1 | 2908.385451 | 0.031757993 | 20 |
| 4318 | RUNX2 | 2668.555128 | 0.031781944 | 20 |
| 4372 | NCOA2 | 650.8887295 | 0.031544055 | 20 |
| 4447 | CDK1 | 1802.129561 | 0.031764833 | 20 |
| 4611 | NFKBIA | 789.241677 | 0.031829953 | 20 |
| 4191 | AR | 1618.954196 | 0.031843696 | 19 |
| 4206 | AKR1C3 | 548.1290892 | 0.030974381 | 18 |
| 4856 | PPARA | 3548.243227 | 0.031867776 | 18 |
| 4944 | VEGFA | 785.9258397 | 0.031659154 | 18 |
| 4322 | RXRB | 470.4255202 | 0.031652361 | 17 |
| 4697 | PRKCB | 586.0561233 | 0.031550802 | 17 |
| 4304 | E2F1 | 1670.818631 | 0.031574441 | 16 |
| 4308 | RXRG | 371.5491763 | 0.031648965 | 16 |
| 4331 | JAK3 | 225.3447186 | 0.031577821 | 16 |
| 4512 | EGF | 1276.823985 | 0.031584582 | 16 |
| 4676 | PPARG | 609.656382 | 0.031689763 | 16 |
| 4955 | PTK2B | 2266.379077 | 0.031564306 | 16 |
| 4272 | RARA | 242.2597892 | 0.031665951 | 15 |
| 4423 | BIRC5 | 799.2355308 | 0.031527199 | 15 |
| 4430 | CCNA2 | 325.7292587 | 0.031446541 | 15 |
| 4498 | IL1B | 338.4472012 | 0.031496904 | 15 |
| 4218 | HSD3B1 | 142.5841971 | 0.030893287 | 14 |
| 4220 | CYP17A1 | 212.819607 | 0.031003678 | 14 |
| 4226 | HSD3B2 | 142.5841971 | 0.030893287 | 14 |
| 4248 | CYP19A1 | 2319.142645 | 0.031399681 | 14 |
| 4264 | IL2 | 80.43689223 | 0.031642175 | 14 |
| 4157 | CASP3 | 198.7432119 | 0.031557552 | 13 |
| 4325 | BCL2 | 197.78769 | 0.03164557 | 13 |
| 4355 | CDK6 | 125.0597075 | 0.031456601 | 13 |
| 4425 | CCNB1 | 183.3248148 | 0.031283139 | 13 |
| 4552 | CXCL8 | 705.6427524 | 0.031459955 | 13 |
| 4556 | CDK2 | 102.2568131 | 0.031544055 | 13 |
| 4148 | ACTB | 151.3347648 | 0.031557552 | 12 |
| 4159 | GSK3B | 716.4180362 | 0.031628605 | 12 |
| 4294 | NOS2 | 94.37193385 | 0.031727253 | 12 |
| 4298 | RARB | 162.8845912 | 0.031621824 | 12 |
| 4329 | RARG | 162.8845912 | 0.031621824 | 12 |
| 4363 | CASP8 | 668.0699025 | 0.031513727 | 12 |
| 4541 | IL1A | 104.5008842 | 0.031392998 | 12 |
| 5246 | IFNG | 97.90157569 | 0.031513727 | 12 |
| 4284 | BCL2L1 | 111.6895198 | 0.031486818 | 11 |
| 4311 | MCL1 | 109.1972498 | 0.031517094 | 11 |
| 4313 | CHUK | 93.19690059 | 0.031480098 | 11 |
| 4377 | COX5B | 1843.171429 | 0.029825094 | 11 |
| 4381 | COX5A | 1843.171429 | 0.029825094 | 11 |
| 4508 | HMOX1 | 2763.716983 | 0.031547428 | 11 |
| 4673 | CEBPB | 104.9592806 | 0.031699979 | 11 |
| 4920 | PTPN2 | 70.61322881 | 0.03125 | 11 |
| 4155 | NOS3 | 71.5462984 | 0.031652361 | 10 |
| 4212 | AKR1C1 | 54.70236812 | 0.030806182 | 10 |
| 4213 | AKR1D1 | 39.09696386 | 0.030738773 | 10 |
| 4240 | CYP1A2 | 129.4516719 | 0.030774045 | 10 |
| 4320 | PTPN1 | 325.9555091 | 0.031443189 | 10 |
| 4336 | IKBKB | 60.65573964 | 0.031470023 | 10 |
| 4347 | PTGS2 | 2147.179737 | 0.031557552 | 10 |
| 4510 | KDR | 168.5477973 | 0.031473381 | 10 |
| 4537 | CCL2 | 447.7368961 | 0.031372966 | 10 |
| 4872 | VDR | 1059.071087 | 0.031177341 | 10 |
| 4930 | ERBB2 | 77.45524979 | 0.031527199 | 10 |
| 4385 | COX4I1 | 5.1 | 0.029072632 | 9 |
| 4523 | IGFBP3 | 646.4215228 | 0.031618435 | 9 |
| 4547 | CXCL10 | 675.4405895 | 0.031141138 | 9 |
| 4560 | TYMS | 764.2599092 | 0.031042829 | 9 |
| 4564 | CHEK1 | 370.0304961 | 0.031466667 | 9 |
| 4705 | MMP2 | 317.5650053 | 0.03136963 | 9 |
| 4717 | COX6B1 | 151.647619 | 0.029072632 | 9 |
| 4726 | COX7C | 151.647619 | 0.029072632 | 9 |
| 4802 | HCK | 114.2585671 | 0.031376303 | 9 |
| 4816 | POR | 1234.44007 | 0.031483458 | 9 |
| 4915 | FGF2 | 113.9980783 | 0.031326325 | 9 |
| 5081 | GABRA1 | 44 | 0.003484321 | 9 |
| 4151 | IRF1 | 24.41043149 | 0.03153731 | 8 |
| 4188 | CACNA1C | 43.9 | 0.003496379 | 8 |
| 4224 | EPHX1 | 189.9502711 | 0.030974381 | 8 |
| 4270 | RAF1 | 215.6016654 | 0.031470023 | 8 |
| 4274 | ERBB3 | 35.2792737 | 0.031449893 | 8 |
| 4315 | CD40LG | 34.88396026 | 0.0314164 | 8 |
| 4349 | CYP2C19 | 683.6214969 | 0.031046096 | 8 |
| 4393 | COX6A2 | 226.5380952 | 0.029069767 | 8 |
| 4432 | RASA1 | 143.2157905 | 0.031426441 | 8 |
| 4434 | RORC | 40.9295329 | 0.031470023 | 8 |
| 4492 | PARP1 | 417.3047042 | 0.031332979 | 8 |
| 4529 | ESR2 | 62.67525855 | 0.031584582 | 8 |
| 4567 | E2F2 | 2.033802309 | 0.031296414 | 8 |
| 4700 | NFKB2 | 8.945233074 | 0.031236764 | 8 |
| 4728 | COX6C | 0.666666667 | 0.029069767 | 8 |
| 5039 | MMP1 | 67.94788255 | 0.031190527 | 8 |
| 5119 | GRIA2 | 2375.385131 | 0.031296414 | 8 |
| 5161 | IL6ST | 13.53190411 | 0.031356293 | 8 |
| 4190 | AHR | 997.4585123 | 0.031584582 | 7 |
| 4193 | CYP1B1 | 212.9075818 | 0.03105917 | 7 |
| 4215 | SRD5A1 | 1.850694998 | 0.030633437 | 7 |
| 4228 | SRD5A2 | 1.850694998 | 0.030633437 | 7 |
| 4230 | SULT2B1 | 4.78639183 | 0.030799749 | 7 |
| 4258 | HSD11B1 | 526.599218 | 0.031342966 | 7 |
| 4281 | NCF1 | 873.9773666 | 0.031403023 | 7 |
| 4340 | VAV1 | 26.61136663 | 0.031372966 | 7 |
| 4427 | RORA | 0 | 0.031356293 | 7 |
| 4437 | PIM1 | 0 | 0.031356293 | 7 |
| 4445 | TOP2A | 43.1268857 | 0.031026504 | 7 |
| 4451 | CACNA1S | 1.9 | 0.003496255 | 7 |
| 4453 | CACNA1F | 1.9 | 0.003496255 | 7 |
| 4459 | CACNA1D | 1.9 | 0.003496255 | 7 |
| 4722 | COX8A | 0 | 0.029066903 | 7 |
| 4724 | COX7B | 0 | 0.029066903 | 7 |
| 4808 | CYB5A | 771.3230349 | 0.031075529 | 7 |
| 4946 | MET | 26.11129985 | 0.031322999 | 7 |
| 4969 | ELK1 | 6.546980277 | 0.031463311 | 7 |
| 5041 | MMP3 | 160.3703812 | 0.031190527 | 7 |
| 5060 | NFE2L2 | 193.2165916 | 0.031456601 | 7 |
| 4210 | CBR1 | 403.6096814 | 0.030886818 | 6 |
| 4246 | HSD17B1 | 5.449178162 | 0.030851286 | 6 |
| 4291 | HSPA5 | 430.8509633 | 0.03136963 | 6 |
| 4296 | CASP9 | 48.46065127 | 0.03135296 | 6 |
| 4345 | PTGS1 | 473.6654193 | 0.030614363 | 6 |
| 4396 | BAX | 19.52952323 | 0.03140971 | 6 |
| 4461 | CACNB3 | 0.4 | 0.003496213 | 6 |
| 4495 | COL1A1 | 613.5866212 | 0.031197124 | 6 |
| 4543 | CXCL2 | 5.733566925 | 0.031144426 | 6 |
| 4587 | PSMD3 | 536.5149885 | 0.031279822 | 6 |
| 4660 | HDAC6 | 25.70502796 | 0.031493541 | 6 |
| 4768 | CSNK2A1 | 1101.879495 | 0.031560929 | 6 |
| 4841 | SULT2A1 | 63.38324721 | 0.031339637 | 6 |
| 4901 | GRIN1 | 530 | 0.030500414 | 6 |
| 5027 | NR1H4 | 1.571086691 | 0.031141138 | 6 |
| 5043 | MMP9 | 2.4998557 | 0.031013457 | 6 |
| 5126 | GRIN2A | 368.6658957 | 0.030799749 | 6 |
| 5339 | IL4R | 604.6495793 | 0.031157583 | 6 |
| 4208 | DHFR | 571.2794327 | 0.030958128 | 5 |
| 4232 | SULT1E1 | 0 | 0.03084806 | 5 |
| 4237 | HPGDS | 848.5809658 | 0.031326325 | 5 |
| 4268 | HSPB1 | 1.034422389 | 0.031571062 | 5 |
| 4286 | INSR | 103.048013 | 0.031316348 | 5 |
| 4376 | ATP5A1 | 170.5 | 0.029804001 | 5 |
| 4379 | ATP5B | 6096 | 0.030585796 | 5 |
| 4383 | ATP5C1 | 243.5571429 | 0.029807012 | 5 |
| 4442 | TK1 | 602.0316853 | 0.030925674 | 5 |
| 4457 | CACNB4 | 0 | 0.003496172 | 5 |
| 4463 | CACNB2 | 0 | 0.003496172 | 5 |
| 4487 | TOP1 | 160.3127708 | 0.031203723 | 5 |
| 4562 | FEN1 | 19.98154159 | 0.030945138 | 5 |
| 4982 | PGR | 16.6720463 | 0.031506996 | 5 |
| 5022 | FABP6 | 0.515018315 | 0.031137851 | 5 |
| 5082 | GABRA4 | 0 | 0.003484156 | 5 |
| 5084 | GABRA6 | 0 | 0.003484156 | 5 |
| 5092 | GABRA5 | 0 | 0.003484156 | 5 |
| 5094 | GABRA3 | 0 | 0.003484156 | 5 |
| 5096 | GABRA2 | 0 | 0.003484156 | 5 |
| 5120 | GRIN2D | 0 | 0.03049726 | 5 |
| 5122 | GRIN2C | 0 | 0.03049726 | 5 |
| 5128 | GRIN2B | 0 | 0.03049726 | 5 |
| 4139 | ABCB1 | 845.2230809 | 0.031560929 | 4 |
| 4153 | GJA1 | 352.833444 | 0.031309701 | 4 |
| 4171 | RUVBL2 | 13.59138675 | 0.031396339 | 4 |
| 4176 | DPP4 | 1586.073378 | 0.031276506 | 4 |
| 4181 | COMT | 1108.659963 | 0.03070358 | 4 |
| 4256 | HSD11B2 | 53.65876149 | 0.03069719 | 4 |
| 4288 | ITPR1 | 146.2314456 | 0.031154293 | 4 |
| 4388 | ESRRA | 6582.391628 | 0.03136963 | 4 |
| 4455 | CACNB1 | 0 | 0.003496131 | 4 |
| 4534 | PTGES | 138.7111796 | 0.030912711 | 4 |
| 4760 | CP | 664.1889834 | 0.030716368 | 4 |
| 4775 | CSNK2B | 110.6718185 | 0.031289775 | 4 |
| 4844 | GSTM1 | 664.3406151 | 0.03083194 | 4 |
| 4849 | GSTP1 | 351.1122204 | 0.031436488 | 4 |
| 5031 | POLB | 123.5066464 | 0.031180636 | 4 |
| 5148 | HSF1 | 12.50170593 | 0.031342966 | 4 |
| 5210 | HMGCR | 0 | 0.031216931 | 4 |
| 5283 | VCAM1 | 1058.880159 | 0.030777256 | 4 |
| 5296 | IGF2 | 10.27088896 | 0.030906234 | 4 |
| 5417 | LGALS4 | 8.943408345 | 0.031480098 | 4 |
| 5454 | RPS6KA5 | 0 | 0.031313024 | 4 |
| 4205 | AKR1B1 | 35.6956214 | 0.030459473 | 3 |
| 4306 | PIK3CG | 1.477446847 | 0.031216931 | 3 |
| 4344 | ALOX5 | 39.83875996 | 0.030819056 | 3 |
| 4501 | COL3A1 | 52.58337834 | 0.030732368 | 3 |
| 4576 | CHEK2 | 5.607299401 | 0.031197124 | 3 |
| 4619 | CDA | 120.1149225 | 0.03033419 | 3 |
| 4623 | TYMP | 15.25243271 | 0.030327953 | 3 |
| 4715 | MAOB | 72.5170952 | 0.030472059 | 3 |
| 4744 | COX7A1 | 0 | 0.028340859 | 3 |
| 4762 | MPO | 554.3631997 | 0.030077488 | 3 |
| 4764 | FXN | 0 | 0.030693996 | 3 |
| 4766 | FECH | 0 | 0.030693996 | 3 |
| 4780 | CTSD | 0 | 0.031236764 | 3 |
| 4866 | NR1I3 | 219.2847546 | 0.031216931 | 3 |
| 4884 | NR1I2 | 100.0422328 | 0.031263247 | 3 |
| 4935 | LGALS3 | 15.16189298 | 0.031177341 | 3 |
| 4949 | PLAU | 600.0897782 | 0.031036297 | 3 |
| 5051 | KDM6B | 1.299112364 | 0.031366295 | 3 |
| 5075 | G6PD | 562.534382 | 0.031174046 | 3 |
| 5090 | GABRB3 | 1 | 0.003484074 | 3 |
| 5098 | GABRG2 | 1 | 0.003484074 | 3 |
| 5154 | SPP1 | 269.557374 | 0.030932159 | 3 |
| 5166 | SERPINE1 | 605.0881081 | 0.031108299 | 3 |
| 5216 | NQO1 | 60.96946511 | 0.031269875 | 3 |
| 5229 | NR3C2 | 419.1854617 | 0.031356293 | 3 |
| 5521 | SLC2A4 | 0.597222222 | 0.031266561 | 3 |
| 5607 | TDP1 | 0.866666667 | 0.03049726 | 3 |
| 5626 | PPARD | 0 | 0.031029768 | 3 |
| 4173 | ADA | 530 | 0.030428056 | 2 |
| 4178 | ADH1B | 530 | 0.029885523 | 2 |
| 4184 | ADRA1B | 18 | 0.003495882 | 2 |
| 4186 | ADRB2 | 32 | 0.003496172 | 2 |
| 4279 | SOD1 | 0 | 0.031144426 | 2 |
| 4369 | SHBG | 530 | 0.030964627 | 2 |
| 4525 | TGFBR2 | 10.56561742 | 0.030948384 | 2 |
| 4545 | CCR1 | 0 | 0.030541464 | 2 |
| 4574 | PHF8 | 0 | 0.030890052 | 2 |
| 4621 | CES2 | 190.4606538 | 0.030415507 | 2 |
| 4707 | PCOLCE | 0 | 0.030349794 | 2 |
| 4770 | OGT | 530 | 0.03069719 | 2 |
| 4895 | FPGS | 0 | 0.030315487 | 2 |
| 4927 | F3 | 530 | 0.030719567 | 2 |
| 4966 | EGLN1 | 0 | 0.031296414 | 2 |
| 5072 | TFRC | 2 | 0.003401361 | 2 |
| 5076 | GCK | 36.87620403 | 0.031052632 | 2 |
| 5086 | GABRB2 | 0 | 0.003484033 | 2 |
| 5088 | GABRB1 | 0 | 0.003484033 | 2 |
| 5117 | SCN5A | 2 | 0.030573116 | 2 |
| 5182 | OPRM1 | 45.80630063 | 0.031118143 | 2 |
| 5252 | KCNH2 | 182.4883322 | 0.031141138 | 2 |
| 5275 | SIGMAR1 | 1.765873016 | 0.030531981 | 2 |
| 5280 | ICAM1 | 0 | 0.029952279 | 2 |
| 5281 | SELE | 0 | 0.029952279 | 2 |
| 5391 | PAPSS1 | 1.288034188 | 0.030799749 | 2 |
| 5447 | PLA2G1B | 0 | 0.03123015 | 2 |
| 5530 | NOX4 | 2.576923077 | 0.030687611 | 2 |
| 5612 | PLAT | 32.09200206 | 0.030268828 | 2 |
| 5613 | UBA1 | 29.25992775 | 0.030200655 | 2 |
| 5651 | TBXAS1 | 39.83875996 | 0.030709973 | 2 |
| 5703 | TRPA1 | 0 | 0.003401361 | 2 |
| 5704 | TRPM8 | 0 | 0.003401361 | 2 |
| 5706 | TRPV1 | 0 | 0.003401361 | 2 |
| 4174 | ADK | 0 | 0.029618474 | 1 |
| 4179 | ADH1C | 0 | 0.029104183 | 1 |
| 4183 | ADRA1A | 0 | 0.003495509 | 1 |
| 4203 | AHSA1 | 0 | 0.031131279 | 1 |
| 4449 | CA9 | 0 | 0.031072256 | 1 |
| 4518 | F2R | 0 | 0.03087712 | 1 |
| 4789 | CXCL11 | 0 | 0.030293695 | 1 |
| 4798 | TNFAIP6 | 0 | 0.030595312 | 1 |
| 4813 | MB | 0 | 0.030231605 | 1 |
| 4831 | HSD17B11 | 0 | 0.030538302 | 1 |
| 4871 | CYP27B1 | 0 | 0.030327953 | 1 |
| 4882 | UGT2B7 | 0 | 0.030371667 | 1 |
| 4898 | SLC5A2 | 0 | 0.03042178 | 1 |
| 4900 | DRD1 | 0 | 0.029687028 | 1 |
| 4912 | EFTUD1 | 0 | 0.003389831 | 1 |
| 4913 | EIF6 | 0 | 0.003389831 | 1 |
| 4937 | SLC5A1 | 0 | 0.030997163 | 1 |
| 5020 | F7 | 0 | 0.029894609 | 1 |
| 5034 | FGF1 | 0 | 0.031134565 | 1 |
| 5071 | FTH1 | 0 | 0.003401321 | 1 |
| 5079 | HK2 | 0 | 0.030324836 | 1 |
| 5113 | GC | 0 | 0.030327953 | 1 |
| 5145 | PPP3CA | 0 | 0.030754796 | 1 |
| 5152 | GSTM2 | 0 | 0.030001017 | 1 |
| 5185 | RUNX1T1 | 0 | 0.031039562 | 1 |
| 5187 | KDM5C | 0 | 0.031039562 | 1 |
| 5266 | HSPA2 | 0 | 0.031131279 | 1 |
| 5312 | IL10RB | 0 | 0.030748384 | 1 |
| 5341 | OPRD1 | 0 | 0.030309257 | 1 |
| 5428 | KANSL3 | 0 | 0.029873418 | 1 |
| 5515 | PON1 | 0 | 0.029286211 | 1 |
| 5602 | ODC1 | 0 | 0.030424917 | 1 |
| 5692 | SERPINA6 | 0 | 0.030126634 | 1 |
| 5696 | TF | 0 | 0.003401321 | 1 |
