# Supplementary material for: Mechanism of drug-pairs Astragalus Mongholicus–Largehead Atractylodes on treating knee osteoarthritis investigated by GEO gene chip with network pharmacology and molecular docking
Source: Medicine (Baltimore). 2024 Jul 5;103(27):e38699. doi: 10.1097/MD.0000000000038699 (PMC11224889; doi:10.1097/MD.0000000000038699)
Supplement: Supplementary file 7 [file medi-103-e38699-s007.doc]

# Appendix 7

**Filter once after the 71 core target gene of AM-LA and KOA**

**Table S7. Filter once after the 71 core target gene of AM-LA and KOA.**

| SUID | Gene symbol | Betweenness | Closeness | Degree |
| --- | --- | --- | --- | --- |
| 4334 | RXRA | 139.7745209 | 0.546875 | 21 |
| 4331 | JAK3 | 13.11498352 | 0.496453901 | 11 |
| 4329 | RARG | 20.00376337 | 0.507246377 | 12 |
| 4200 | ESR1 | 191.2519975 | 0.588235294 | 21 |
| 4327 | CDKN1A | 91.11026432 | 0.555555556 | 20 |
| 4198 | CYP1A1 | 13.09433704 | 0.4 | 5 |
| 4325 | BCL2 | 29.57693812 | 0.510948905 | 12 |
| 4196 | STAT1 | 68.21505048 | 0.551181102 | 17 |
| 4322 | RXRB | 24.93086434 | 0.503597122 | 14 |
| 4705 | MMP2 | 2.506747038 | 0.419161677 | 4 |
| 4447 | CDK1 | 52.73639709 | 0.510948905 | 15 |
| 4191 | AR | 86.42744844 | 0.56 | 17 |
| 4318 | RUNX2 | 41.03484072 | 0.526315789 | 14 |
| 4955 | PTK2B | 29.17732085 | 0.479452055 | 11 |
| 4697 | PRKCB | 9.959884851 | 0.479452055 | 9 |
| 4440 | FOS | 179.0306377 | 0.583333333 | 21 |
| 4311 | MCL1 | 5.60639635 | 0.479452055 | 8 |
| 4564 | CHEK1 | 5.833140281 | 0.440251572 | 5 |
| 4308 | RXRG | 24.93086434 | 0.503597122 | 14 |
| 4816 | POR | 8.902208902 | 0.414201183 | 3 |
| 4944 | VEGFA | 26.54334947 | 0.476190476 | 9 |
| 4304 | E2F1 | 10.38002713 | 0.472972973 | 12 |
| 4430 | CCNA2 | 11.01374813 | 0.45751634 | 11 |
| 4302 | JAK1 | 45.73467413 | 0.510948905 | 15 |
| 4556 | CDK2 | 16.28999896 | 0.479452055 | 12 |
| 4298 | RARB | 20.00376337 | 0.507246377 | 12 |
| 4425 | CCNB1 | 7.983087028 | 0.414201183 | 10 |
| 4169 | JUN | 368.8011083 | 0.642201835 | 31 |
| 4552 | CXCL8 | 12.45412496 | 0.466666667 | 10 |
| 4423 | BIRC5 | 12.05056617 | 0.451612903 | 8 |
| 4167 | RELA | 215.233 | 0.619469027 | 28 |
| 4550 | IL6 | 130.4049804 | 0.534351145 | 19 |
| 4165 | AKT1 | 277.4816913 | 0.625 | 29 |
| 4676 | PPARG | 36.7693401 | 0.514705882 | 13 |
| 4163 | PRKCA | 70.05956797 | 0.526315789 | 14 |
| 4802 | HCK | 9.856949994 | 0.432098765 | 7 |
| 4673 | CEBPB | 16.67398287 | 0.52238806 | 11 |
| 4161 | HSP90AA1 | 291.787243 | 0.583333333 | 25 |
| 4159 | GSK3B | 21.31989982 | 0.514705882 | 10 |
| 4541 | IL1A | 2.078131019 | 0.448717949 | 8 |
| 4157 | CASP3 | 14.05940397 | 0.496453901 | 10 |
| 4284 | BCL2L1 | 4.799701448 | 0.472972973 | 8 |
| 4537 | CCL2 | 1.023202614 | 0.445859873 | 7 |
| 4149 | NR3C1 | 65.86009929 | 0.578512397 | 19 |
| 4148 | ACTB | 8.964010184 | 0.496453901 | 8 |
| 4276 | RB1 | 72.60851339 | 0.569105691 | 19 |
| 4915 | FGF2 | 8.91610032 | 0.445859873 | 5 |
| 4403 | MAPK1 | 499.6476489 | 0.67961165 | 37 |
| 4146 | MAPK8 | 74.67982144 | 0.56 | 17 |
| 4144 | CAV1 | 5.807269952 | 0.479452055 | 7 |
| 4272 | RARA | 35.56789996 | 0.52238806 | 15 |
| 4140 | TP53 | 302.5023484 | 0.630630631 | 31 |
| 4266 | MAPK14 | 229.3990131 | 0.603448276 | 26 |
| 4262 | NFKB1 | 52.72229818 | 0.534351145 | 16 |
| 4512 | EGF | 28.80959096 | 0.476190476 | 10 |
| 4248 | CYP19A1 | 5.433104007 | 0.384615385 | 3 |
| 4374 | NCOA1 | 76.69170353 | 0.496453901 | 15 |
| 4372 | NCOA2 | 32.49356027 | 0.454545455 | 14 |
| 4498 | IL1B | 15.33482354 | 0.472972973 | 11 |
| 4367 | HDAC1 | 210.2854002 | 0.598290598 | 27 |
| 4363 | CASP8 | 13.25730359 | 0.492957746 | 8 |
| 4872 | VDR | 0.5 | 0.397727273 | 8 |
| 4358 | EGFR | 76.85873576 | 0.555555556 | 17 |
| 4355 | CDK6 | 20.39742739 | 0.486111111 | 12 |
| 4611 | NFKBIA | 22.97857388 | 0.538461538 | 13 |
| 4351 | CCND1 | 83.05899979 | 0.56 | 20 |
| 5246 | IFNG | 7.018483667 | 0.469798658 | 8 |
| 4856 | PPARA | 69.03394288 | 0.542635659 | 13 |
| 4342 | MYC | 80.87531645 | 0.588235294 | 21 |
| 4338 | HIF1A | 45.99731656 | 0.564516129 | 16 |
| 4593 | JAK2 | 64.28054623 | 0.534351145 | 15 |
