# Supplementary material for: Mechanism of drug-pairs Astragalus Mongholicus–Largehead Atractylodes on treating knee osteoarthritis investigated by GEO gene chip with network pharmacology and molecular docking
Source: Medicine (Baltimore). 2024 Jul 5;103(27):e38699. doi: 10.1097/MD.0000000000038699 (PMC11224889; doi:10.1097/MD.0000000000038699)
Supplement: Supplementary file 8 [file medi-103-e38699-s008.doc]

# Appendix 8

**Filter twice after the 21 core target genes**

**Table S8. Filter twice after the 21 core target genes.**

| SUID | Gene symbol | Betweenness | Closeness | Degree |
| --- | --- | --- | --- | --- |
| 4334 | RXRA | 4.322553264 | 0.606060606 | 7 |
| 4140 | TP53 | 16.42787539 | 0.833333333 | 16 |
| 4266 | MAPK14 | 8.677849928 | 0.714285714 | 12 |
| 4169 | JUN | 16.39710975 | 0.8 | 15 |
| 4200 | ESR1 | 5.73982684 | 0.740740741 | 13 |
| 4327 | CDKN1A | 5.105929038 | 0.666666667 | 10 |
| 4167 | RELA | 7.009362533 | 0.714285714 | 12 |
| 4550 | IL6 | 0.583333333 | 0.555555556 | 5 |
| 4165 | AKT1 | 13.62063492 | 0.714285714 | 12 |
| 4196 | STAT1 | 8.731049996 | 0.64516129 | 9 |
| 4161 | HSP90AA1 | 8.020286903 | 0.689655172 | 11 |
| 4447 | CDK1 | 3.352380952 | 0.588235294 | 7 |
| 4351 | CCND1 | 7.994211018 | 0.689655172 | 11 |
| 4440 | FOS | 6.991456583 | 0.689655172 | 11 |
| 4342 | MYC | 11.55887446 | 0.769230769 | 14 |
| 4149 | NR3C1 | 8.376262626 | 0.689655172 | 11 |
| 4276 | RB1 | 6.041787624 | 0.666666667 | 10 |
| 4403 | MAPK1 | 25.1888422 | 0.8 | 15 |
| 4338 | HIF1A | 5.284271284 | 0.714285714 | 12 |
| 4272 | RARA | 1.493795094 | 0.588235294 | 6 |
| 4367 | HDAC1 | 19.08230626 | 0.740740741 | 13 |
