# Supplementary material for: Mechanism of drug-pairs Astragalus Mongholicus–Largehead Atractylodes on treating knee osteoarthritis investigated by GEO gene chip with network pharmacology and molecular docking
Source: Medicine (Baltimore). 2024 Jul 5;103(27):e38699. doi: 10.1097/MD.0000000000038699 (PMC11224889; doi:10.1097/MD.0000000000038699)
Supplement: Supplementary file 9 [file medi-103-e38699-s009.doc]

# Appendix 9

**Filter thrice after the 5 core target genes**

**Table S9. Filter thrice after the 5 core target genes.**

| SUID | name | Protein name | Betweenness | Closeness | Degree |
| --- | --- | --- | --- | --- | --- |
| 4140 | TP53 | Cellular tumor antigen p53 | 16.42787539 | 0.833333333 | 16 |
| 4403 | MAPK1 | Mitogen-activated protein kinase 1 | 25.1888422 | 0.8 | 15 |
| 4169 | JUN | Transcription factor AP-1 | 16.39710975 | 0.8 | 15 |
| 4342 | MYC | Myc proto-oncogene protein | 11.55887446 | 0.769230769 | 14 |
| 4266 | MAPK14 | Mitogen-activated protein kinase 14 | 8.677849928 | 0.714285714 | 12 |
