# Supplementary material for: Mechanism of drug-pairs Astragalus Mongholicus–Largehead Atractylodes on treating knee osteoarthritis investigated by GEO gene chip with network pharmacology and molecular docking
Source: Medicine (Baltimore). 2024 Jul 5;103(27):e38699. doi: 10.1097/MD.0000000000038699 (PMC11224889; doi:10.1097/MD.0000000000038699)
Supplement: Supplementary file 10 [file medi-103-e38699-s010.doc]

# Appendix 10

**Molecular function(MF) of GO enrichment analysis**

**Table S10. Molecular function(MF) of GO enrichment analysis.**

| ID | Description | GeneRatio | pvalue | qvalue | geneID | Count |
| --- | --- | --- | --- | --- | --- | --- |
| GO:0015267 | channel activity | 49/416 | 4.05E-19 | 8.43E-18 | SCN5A/OPRM1/GABRA1/GRIA2/HTR3A/KCNH2/BCL2/BAX/GJA1/CLDN4/GLRA1/GLRA2/NMUR2/TRPA1/TRPM8/GABRA2/GABRA3/GABRA4/GABRA5/GABRA6/GABRG1/GABRG2/GABRG3/ITPR1/GABRB1/GABRB2/GABRB3/GABRD/GABRE/GABRP/GABRQ/GRIN1/GRIN2A/GRIN2B/GRIN2C/GRIN2D/GRIN3A/GRIN3B/CACNA1C/CACNA1D/CACNA1F/CACNA1S/CACNB1/CACNB2/CACNB3/CACNB4/TRPV3/TRPV1/PTK2B | 49 |
| GO:0022803 | passive transmembrane transporter activity | 49/416 | 4.45E-19 | 8.83E-18 | SCN5A/OPRM1/GABRA1/GRIA2/HTR3A/KCNH2/BCL2/BAX/GJA1/CLDN4/GLRA1/GLRA2/NMUR2/TRPA1/TRPM8/GABRA2/GABRA3/GABRA4/GABRA5/GABRA6/GABRG1/GABRG2/GABRG3/ITPR1/GABRB1/GABRB2/GABRB3/GABRD/GABRE/GABRP/GABRQ/GRIN1/GRIN2A/GRIN2B/GRIN2C/GRIN2D/GRIN3A/GRIN3B/CACNA1C/CACNA1D/CACNA1F/CACNA1S/CACNB1/CACNB2/CACNB3/CACNB4/TRPV3/TRPV1/PTK2B | 49 |
| GO:0005216 | ion channel activity | 46/416 | 1.72E-18 | 3.26E-17 | SCN5A/OPRM1/GABRA1/GRIA2/HTR3A/KCNH2/CLDN4/GLRA1/GLRA2/NMUR2/TRPA1/TRPM8/GABRA2/GABRA3/GABRA4/GABRA5/GABRA6/GABRG1/GABRG2/GABRG3/ITPR1/GABRB1/GABRB2/GABRB3/GABRD/GABRE/GABRP/GABRQ/GRIN1/GRIN2A/GRIN2B/GRIN2C/GRIN2D/GRIN3A/GRIN3B/CACNA1C/CACNA1D/CACNA1F/CACNA1S/CACNB1/CACNB2/CACNB3/CACNB4/TRPV3/TRPV1/PTK2B | 46 |
| GO:0022838 | substrate-specific channel activity | 46/416 | 5.32E-18 | 9.67E-17 | SCN5A/OPRM1/GABRA1/GRIA2/HTR3A/KCNH2/CLDN4/GLRA1/GLRA2/NMUR2/TRPA1/TRPM8/GABRA2/GABRA3/GABRA4/GABRA5/GABRA6/GABRG1/GABRG2/GABRG3/ITPR1/GABRB1/GABRB2/GABRB3/GABRD/GABRE/GABRP/GABRQ/GRIN1/GRIN2A/GRIN2B/GRIN2C/GRIN2D/GRIN3A/GRIN3B/CACNA1C/CACNA1D/CACNA1F/CACNA1S/CACNB1/CACNB2/CACNB3/CACNB4/TRPV3/TRPV1/PTK2B | 46 |
| GO:0022839 | ion gated channel activity | 43/416 | 7.34E-20 | 1.89E-18 | SCN5A/OPRM1/GABRA1/GRIA2/HTR3A/KCNH2/GLRA1/GLRA2/NMUR2/TRPA1/GABRA2/GABRA3/GABRA4/GABRA5/GABRA6/GABRG1/GABRG2/GABRG3/ITPR1/GABRB1/GABRB2/GABRB3/GABRD/GABRE/GABRP/GABRQ/GRIN1/GRIN2A/GRIN2B/GRIN2C/GRIN2D/GRIN3A/GRIN3B/CACNA1C/CACNA1D/CACNA1F/CACNA1S/CACNB1/CACNB2/CACNB3/CACNB4/TRPV1/PTK2B | 43 |
| GO:0022836 | gated channel activity | 43/416 | 2.06E-19 | 4.99E-18 | SCN5A/OPRM1/GABRA1/GRIA2/HTR3A/KCNH2/GLRA1/GLRA2/NMUR2/TRPA1/GABRA2/GABRA3/GABRA4/GABRA5/GABRA6/GABRG1/GABRG2/GABRG3/ITPR1/GABRB1/GABRB2/GABRB3/GABRD/GABRE/GABRP/GABRQ/GRIN1/GRIN2A/GRIN2B/GRIN2C/GRIN2D/GRIN3A/GRIN3B/CACNA1C/CACNA1D/CACNA1F/CACNA1S/CACNB1/CACNB2/CACNB3/CACNB4/TRPV1/PTK2B | 43 |
| GO:0030594 | neurotransmitter receptor activity | 34/416 | 6.28E-28 | 1.37E-25 | CHRM3/CHRM1/CHRM2/OPRM1/GABRA1/GRIA2/ADRB1/HTR3A/DRD1/CHRM5/CHRM4/GLRA1/GLRA2/GABRA2/GABRA3/GABRA4/GABRA5/GABRA6/GABRG1/GABRG2/GABRG3/GABRB1/GABRB2/GABRB3/GABRD/GABRE/GRIN1/GRIN2A/GRIN2B/GRIN2C/GRIN2D/GRIN3A/GRIN3B/PTK2B | 34 |
| GO:0015276 | ligand-gated ion channel activity | 32/416 | 5.46E-23 | 2.38E-21 | GABRA1/GRIA2/HTR3A/KCNH2/GLRA1/GLRA2/TRPA1/GABRA2/GABRA3/GABRA4/GABRA5/GABRA6/GABRG1/GABRG2/GABRG3/ITPR1/GABRB1/GABRB2/GABRB3/GABRD/GABRE/GABRP/GABRQ/GRIN1/GRIN2A/GRIN2B/GRIN2C/GRIN2D/GRIN3A/GRIN3B/TRPV1/PTK2B | 32 |
| GO:0022834 | ligand-gated channel activity | 32/416 | 5.46E-23 | 2.38E-21 | GABRA1/GRIA2/HTR3A/KCNH2/GLRA1/GLRA2/TRPA1/GABRA2/GABRA3/GABRA4/GABRA5/GABRA6/GABRG1/GABRG2/GABRG3/ITPR1/GABRB1/GABRB2/GABRB3/GABRD/GABRE/GABRP/GABRQ/GRIN1/GRIN2A/GRIN2B/GRIN2C/GRIN2D/GRIN3A/GRIN3B/TRPV1/PTK2B | 32 |
| GO:0033218 | amide binding | 31/416 | 3.89E-10 | 3.69E-09 | RXRA/ACHE/ADRB2/OPRM1/GRIA2/PPARG/OPRD1/GSTP1/INSR/PPP3CA/GSTM1/GSTM2/NFKBIA/NPEPPS/NQO2/TYMS/DHFR/FOLR1/FOLR2/SLC46A1/BACE1/NMUR2/SRD5A1/PTGES/SOAT1/SOAT2/GRIN1/GRIN2A/GRIN2B/OPRK1/DBI | 31 |
| GO:0046873 | metal ion transmembrane transporter activity | 31/416 | 5.31E-08 | 3.86E-07 | SCN5A/OPRM1/SLC6A2/KCNH2/SLC6A3/SLC6A4/ATP2A1/SLC5A2/SLC5A1/SLC28A3/TRPA1/TRPM8/ITPR1/TF/GRIN1/GRIN2A/GRIN2B/GRIN2C/GRIN2D/GRIN3A/GRIN3B/CACNA1C/CACNA1D/CACNA1F/CACNA1S/CACNB1/CACNB2/CACNB3/CACNB4/TRPV3/TRPV1 | 31 |
| GO:0001228 | DNA-binding transcription activator activity, RNA polymerase II-specific | 30/416 | 1.88E-07 | 1.24E-06 | PGR/AR/ESR1/RELA/RXRB/JUN/STAT1/NR1I2/NR1I3/FOS/TP53/ELK1/HIF1A/MYC/NFE2L2/PARP1/PPARA/RUNX2/E2F2/IRF1/NR3C1/NFKB1/NFKB2/RORA/NR1H4/CEBPB/ESRRA/ESRRB/ESRRG/HOXA10 | 30 |
| GO:0005230 | extracellular ligand-gated ion channel activity | 29/416 | 4.17E-28 | 1.37E-25 | GABRA1/GRIA2/HTR3A/GLRA1/GLRA2/GABRA2/GABRA3/GABRA4/GABRA5/GABRA6/GABRG1/GABRG2/GABRG3/GABRB1/GABRB2/GABRB3/GABRD/GABRE/GABRP/GABRQ/GRIN1/GRIN2A/GRIN2B/GRIN2C/GRIN2D/GRIN3A/GRIN3B/TRPV1/PTK2B | 29 |
| GO:0005496 | steroid binding | 27/416 | 4.03E-22 | 1.56E-20 | PGR/AR/ESR2/ESR1/CYP3A4/CAV1/SULT1E1/HSD11B1/NR3C1/SERPINA6/SHBG/GC/RORC/SOAT1/SOAT2/VDR/HSD11B2/HSD17B1/NR3C2/SULT2B1/RORA/NR1H4/AKR1D1/ESRRA/ESRRB/GPER1/ESRRG | 27 |
| GO:0043177 | organic acid binding | 27/416 | 3.95E-13 | 5.75E-12 | RXRA/NOS2/PPARG/PPARD/TNFAIP6/SELE/NOS3/PPARA/TYMS/DHFR/FOLR1/FOLR2/SLC46A1/GLRA1/GLRA2/VDR/EGLN1/HBA1/AKR1C1/AKR1C2/GRIN1/GRIN2B/GRIN3A/GRIN3B/NR1H4/RARA/PLA2G1B | 27 |
| GO:0022824 | transmitter-gated ion channel activity | 26/416 | 1.18E-26 | 8.58E-25 | GABRA1/GRIA2/HTR3A/GLRA1/GLRA2/GABRA2/GABRA3/GABRA4/GABRA5/GABRA6/GABRG1/GABRG2/GABRG3/GABRB1/GABRB2/GABRB3/GABRD/GABRE/GRIN1/GRIN2A/GRIN2B/GRIN2C/GRIN2D/GRIN3A/GRIN3B/PTK2B | 26 |
| GO:0022835 | transmitter-gated channel activity | 26/416 | 1.18E-26 | 8.58E-25 | GABRA1/GRIA2/HTR3A/GLRA1/GLRA2/GABRA2/GABRA3/GABRA4/GABRA5/GABRA6/GABRG1/GABRG2/GABRG3/GABRB1/GABRB2/GABRB3/GABRD/GABRE/GRIN1/GRIN2A/GRIN2B/GRIN2C/GRIN2D/GRIN3A/GRIN3B/PTK2B | 26 |
| GO:0031406 | carboxylic acid binding | 26/416 | 6.38E-13 | 8.71E-12 | RXRA/NOS2/PPARG/PPARD/TNFAIP6/SELE/NOS3/PPARA/TYMS/DHFR/FOLR1/FOLR2/SLC46A1/GLRA1/GLRA2/VDR/EGLN1/AKR1C1/AKR1C2/GRIN1/GRIN2B/GRIN3A/GRIN3B/NR1H4/RARA/PLA2G1B | 26 |
| GO:0005261 | cation channel activity | 26/416 | 4.07E-08 | 3.01E-07 | SCN5A/OPRM1/GRIA2/HTR3A/KCNH2/TRPA1/TRPM8/ITPR1/GRIN1/GRIN2A/GRIN2B/GRIN2C/GRIN2D/GRIN3A/GRIN3B/CACNA1C/CACNA1D/CACNA1F/CACNA1S/CACNB1/CACNB2/CACNB3/CACNB4/TRPV3/TRPV1/PTK2B | 26 |
| GO:0004674 | protein serine/threonine kinase activity | 26/416 | 1.65E-05 | 7.58E-05 | CDK2/CHEK1/MAPK14/GSK3B/IKBKB/AKT1/MAPK8/CDK1/EGFR/MAPK1/TOP1/RAF1/PRKCA/PRKCB/CHEK2/CHUK/RPS6KA5/NEK1/TGFBR2/CDK6/CSNK2A1/CSNK2B/PIK3CG/PIM1/PTK2B/STK17B | 26 |
| GO:0003707 | steroid hormone receptor activity | 25/416 | 2.66E-26 | 1.66E-24 | PGR/AR/RXRA/ESR2/ESR1/PPARG/PPARD/RXRB/NR1I2/NR1I3/PPARA/NR3C1/RORC/VDR/NR3C2/RORA/NR1H4/ESRRA/ESRRB/GPER1/RARA/RARB/RARG/RXRG/ESRRG | 25 |
| GO:0008509 | anion transmembrane transporter activity | 25/416 | 2.54E-07 | 1.66E-06 | GABRA1/SLC6A2/SLC6A3/SLC6A4/CLDN4/SLC46A1/GLRA1/GLRA2/SLC22A12/NMUR2/GABRA2/GABRA3/GABRA4/GABRA5/GABRA6/GABRG1/GABRG2/GABRG3/GABRB1/GABRB2/GABRB3/GABRD/GABRE/GABRP/GABRQ | 25 |
| GO:0004879 | nuclear receptor activity | 24/416 | 4.11E-27 | 4.49E-25 | PGR/AR/RXRA/ESR2/ESR1/PPARG/PPARD/RXRB/NR1I2/AHR/NR1I3/PPARA/NR3C1/RORC/VDR/RORA/NR1H4/ESRRA/ESRRB/RARA/RARB/RARG/RXRG/ESRRG | 24 |
| GO:0098531 | transcription factor activity, direct ligand regulated sequence-specific DNA binding | 24/416 | 4.11E-27 | 4.49E-25 | PGR/AR/RXRA/ESR2/ESR1/PPARG/PPARD/RXRB/NR1I2/AHR/NR1I3/PPARA/NR3C1/RORC/VDR/RORA/NR1H4/ESRRA/ESRRB/RARA/RARB/RARG/RXRG/ESRRG | 24 |
| GO:0044389 | ubiquitin-like protein ligase binding | 24/416 | 3.13E-07 | 2.01E-06 | SCN5A/GSK3B/RELA/KCNH2/JUN/BCL2/STAT1/EGFR/CDKN1A/RB1/TP53/NFKBIA/CASP8/HIF1A/HSPA5/CCNB1/CHEK2/ERBB3/JAK1/HSPA8/HDAC6/HSP90AA1/CEBPB/PTK2B | 24 |
| GO:0015108 | chloride transmembrane transporter activity | 23/416 | 8.90E-17 | 1.56E-15 | GABRA1/SLC6A2/SLC6A3/SLC6A4/CLDN4/GLRA1/GLRA2/NMUR2/GABRA2/GABRA3/GABRA4/GABRA5/GABRA6/GABRG1/GABRG2/GABRG3/GABRB1/GABRB2/GABRB3/GABRD/GABRE/GABRP/GABRQ | 23 |
| GO:0015103 | inorganic anion transmembrane transporter activity | 23/416 | 4.97E-13 | 7.00E-12 | GABRA1/SLC6A2/SLC6A3/SLC6A4/CLDN4/GLRA1/GLRA2/NMUR2/GABRA2/GABRA3/GABRA4/GABRA5/GABRA6/GABRG1/GABRG2/GABRG3/GABRB1/GABRB2/GABRB3/GABRD/GABRE/GABRP/GABRQ | 23 |
| GO:0016705 | oxidoreductase activity, acting on paired donors, with incorporation or reduction of molecular oxygen | 23/416 | 3.12E-12 | 3.79E-11 | PTGS2/NOS2/PTGS1/HMOX1/CYP3A4/CYP1A2/CYP1A1/CYP1B1/AKR1C3/POR/NOS3/CYP19A1/TYR/CYP17A1/KDM2A/PHF8/CYP2C19/TBXAS1/EGLN1/AKR1C1/AKR1C2/AKR1D1/CYP27B1 | 23 |
| GO:0015085 | calcium ion transmembrane transporter activity | 22/416 | 1.46E-12 | 1.82E-11 | OPRM1/ATP2A1/TRPA1/TRPM8/ITPR1/GRIN1/GRIN2A/GRIN2B/GRIN2C/GRIN2D/GRIN3A/GRIN3B/CACNA1C/CACNA1D/CACNA1F/CACNA1S/CACNB1/CACNB2/CACNB3/CACNB4/TRPV3/TRPV1 | 22 |
| GO:0015077 | monovalent inorganic cation transmembrane transporter activity | 22/416 | 0.000110416 | 0.000408767 | SCN5A/SLC6A2/KCNH2/SLC6A3/SLC6A4/ATP5F1B/ATP2A1/SLC5A2/SLC5A1/SLC46A1/SLC28A3/CYB5A/COX4I1/COX5A/COX5B/COX6A2/COX6B1/COX6C/COX7A1/COX7B/COX7C/COX8A | 22 |
| GO:0099529 | neurotransmitter receptor activity involved in regulation of postsynaptic membrane potential | 21/416 | 1.36E-21 | 4.25E-20 | CHRM1/GABRA1/ADRB1/GLRA1/GLRA2/GABRA2/GABRA3/GABRA4/GABRA5/GABRA6/GABRG1/GABRG2/GABRG3/GABRB1/GABRB2/GABRB3/GABRD/GABRE/GRIN1/GRIN2C/GRIN3A | 21 |
| GO:0098960 | postsynaptic neurotransmitter receptor activity | 21/416 | 3.72E-21 | 1.08E-19 | CHRM1/GABRA1/ADRB1/GLRA1/GLRA2/GABRA2/GABRA3/GABRA4/GABRA5/GABRA6/GABRG1/GABRG2/GABRG3/GABRB1/GABRB2/GABRB3/GABRD/GABRE/GRIN1/GRIN2C/GRIN3A | 21 |
| GO:0009055 | electron transfer activity | 21/416 | 2.19E-13 | 3.30E-12 | AKR1B1/MAOB/NCF1/CYP1A2/POR/NQO1/CYP19A1/NQO2/NOX4/SRD5A1/CYB5A/COX4I1/COX5A/COX5B/COX6A2/COX6B1/COX6C/COX7A1/COX7B/COX7C/COX8A | 21 |
| GO:0005262 | calcium channel activity | 21/416 | 1.04E-12 | 1.33E-11 | OPRM1/TRPA1/TRPM8/ITPR1/GRIN1/GRIN2A/GRIN2B/GRIN2C/GRIN2D/GRIN3A/GRIN3B/CACNA1C/CACNA1D/CACNA1F/CACNA1S/CACNB1/CACNB2/CACNB3/CACNB4/TRPV3/TRPV1 | 21 |
| GO:0020037 | heme binding | 21/416 | 6.69E-12 | 7.69E-11 | PTGS2/NOS2/PTGS1/HMOX1/CYP3A4/CYP1A2/CYP1A1/CYP1B1/DUOX2/NOS3/MPO/CYP19A1/JAK2/CYP17A1/NOX4/CYP2C19/TBXAS1/CYB5A/HBA1/MB/CYP27B1 | 21 |
| GO:0046906 | tetrapyrrole binding | 21/416 | 2.72E-11 | 2.77E-10 | PTGS2/NOS2/PTGS1/HMOX1/CYP3A4/CYP1A2/CYP1A1/CYP1B1/DUOX2/NOS3/MPO/CYP19A1/JAK2/CYP17A1/NOX4/CYP2C19/TBXAS1/CYB5A/HBA1/MB/CYP27B1 | 21 |
| GO:0031625 | ubiquitin protein ligase binding | 21/416 | 5.47E-06 | 2.72E-05 | SCN5A/GSK3B/RELA/KCNH2/JUN/BCL2/EGFR/CDKN1A/RB1/TP53/NFKBIA/CASP8/HIF1A/HSPA5/CHEK2/ERBB3/JAK1/HSPA8/HDAC6/HSP90AA1/PTK2B | 21 |
| GO:0042277 | peptide binding | 21/416 | 7.13E-06 | 3.50E-05 | RXRA/ACHE/ADRB2/OPRM1/GRIA2/PPARG/OPRD1/GSTP1/INSR/PPP3CA/GSTM1/GSTM2/NFKBIA/NPEPPS/BACE1/NMUR2/PTGES/GRIN1/GRIN2A/GRIN2B/OPRK1 | 21 |
| GO:0004175 | endopeptidase activity | 21/416 | 0.00124263 | 0.003435653 | DPP4/PRSS1/F7/CASP3/MMP1/MMP3/CASP9/PLAU/MMP2/MMP9/CASP8/F3/PLAT/CTSD/MMP8/BACE1/CTSK/CTSS/CTSC/CTSF/CTRB1 | 21 |
| GO:0005254 | chloride channel activity | 20/416 | 4.14E-16 | 6.95E-15 | GABRA1/CLDN4/GLRA1/GLRA2/NMUR2/GABRA2/GABRA3/GABRA4/GABRA5/GABRA6/GABRG1/GABRG2/GABRG3/GABRB1/GABRB2/GABRB3/GABRD/GABRE/GABRP/GABRQ | 20 |
| GO:0005253 | anion channel activity | 20/416 | 1.51E-14 | 2.36E-13 | GABRA1/CLDN4/GLRA1/GLRA2/NMUR2/GABRA2/GABRA3/GABRA4/GABRA5/GABRA6/GABRG1/GABRG2/GABRG3/GABRB1/GABRB2/GABRB3/GABRD/GABRE/GABRP/GABRQ | 20 |
| GO:0019902 | phosphatase binding | 20/416 | 1.50E-08 | 1.24E-07 | PPARG/MAPK14/SLC6A3/MET/AKT1/BCL2/STAT1/EGFR/MAPK1/TP53/SOD1/ERBB2/PPARA/PTPN1/JAK3/JAK1/HMGCR/LGALS3/CTSC/GRIN3A | 20 |
| GO:0003713 | transcription coactivator activity | 20/416 | 7.17E-05 | 0.000284696 | RXRA/NCOA2/NCOA1/ESR2/PPARG/PPARD/RXRB/JUN/NR1I2/NR1I3/RB1/PRKCB/PPARA/FGF2/VDR/NFKB2/NR1H4/RARA/RARB/RARG | 20 |
| GO:0048018 | receptor ligand activity | 20/416 | 0.010440247 | 0.020543871 | VEGFA/EGF/IL1B/CCL2/CXCL8/IL2/IFNG/IL1A/CXCL11/CXCL2/CXCL10/SPP1/IGF2/CD40LG/FGF1/FGF2/TYMP/LGALS3/IL6/NPPB | 20 |
| GO:1904315 | transmitter-gated ion channel activity involved in regulation of postsynaptic membrane potential | 19/416 | 2.86E-19 | 6.38E-18 | GABRA1/GLRA1/GLRA2/GABRA2/GABRA3/GABRA4/GABRA5/GABRA6/GABRG1/GABRG2/GABRG3/GABRB1/GABRB2/GABRB3/GABRD/GABRE/GRIN1/GRIN2C/GRIN3A | 19 |
| GO:0005126 | cytokine receptor binding | 19/416 | 5.01E-05 | 0.000214564 | CASP3/STAT1/VEGFA/IL6ST/CASP8/IL1B/CCL2/CXCL8/IL2/IFNG/IL1A/CXCL11/CXCL2/CXCL10/CD40LG/JAK1/JAK2/TGFBR2/IL6 | 19 |
| GO:0051427 | hormone receptor binding | 18/416 | 3.92E-07 | 2.48E-06 | RXRA/NCOA2/NCOA1/ESR1/PPARG/STAT1/RB1/HIF1A/PRKCB/PARP1/JAK1/JAK2/VDR/NR1H4/CEBPB/RARB/RARG/NPPB | 18 |
| GO:0005244 | voltage-gated ion channel activity | 18/416 | 9.91E-07 | 5.85E-06 | SCN5A/OPRM1/KCNH2/GRIN1/GRIN2A/GRIN2B/GRIN2C/GRIN2D/GRIN3A/CACNA1C/CACNA1D/CACNA1F/CACNA1S/CACNB1/CACNB2/CACNB3/CACNB4/PTK2B | 18 |
| GO:0022832 | voltage-gated channel activity | 18/416 | 9.91E-07 | 5.85E-06 | SCN5A/OPRM1/KCNH2/GRIN1/GRIN2A/GRIN2B/GRIN2C/GRIN2D/GRIN3A/CACNA1C/CACNA1D/CACNA1F/CACNA1S/CACNB1/CACNB2/CACNB3/CACNB4/PTK2B | 18 |
| GO:0050662 | coenzyme binding | 18/416 | 0.000192958 | 0.000663717 | NOS2/PYGM/MAOB/POR/NOS3/PARP1/NQO2/DHFR/HMGCR/NOX4/SRD5A1/SOAT1/SOAT2/EGLN1/HSD11B2/HSD17B1/DBI/G6PD | 18 |
| GO:0016616 | oxidoreductase activity, acting on the CH-OH group of donors, NAD or NADP as acceptor | 17/416 | 2.68E-09 | 2.39E-08 | ADH1B/ADH1C/AKR1B1/HSD3B2/HSD3B1/AKR1C3/HSD11B1/HMGCR/SRD5A2/AKR1C1/AKR1C2/HSD11B2/HSD17B1/AKR1D1/HSD17B11/CBR1/G6PD | 17 |
| GO:0016614 | oxidoreductase activity, acting on CH-OH group of donors | 17/416 | 8.34E-09 | 7.29E-08 | ADH1B/ADH1C/AKR1B1/HSD3B2/HSD3B1/AKR1C3/HSD11B1/HMGCR/SRD5A2/AKR1C1/AKR1C2/HSD11B2/HSD17B1/AKR1D1/HSD17B11/CBR1/G6PD | 17 |
| GO:0019903 | protein phosphatase binding | 17/416 | 3.27E-08 | 2.50E-07 | PPARG/MAPK14/SLC6A3/MET/AKT1/BCL2/STAT1/EGFR/TP53/SOD1/ERBB2/PTPN1/JAK3/JAK1/HMGCR/LGALS3/GRIN3A | 17 |
| GO:0022843 | voltage-gated cation channel activity | 17/416 | 4.04E-08 | 3.01E-07 | OPRM1/KCNH2/GRIN1/GRIN2A/GRIN2B/GRIN2C/GRIN2D/GRIN3A/CACNA1C/CACNA1D/CACNA1F/CACNA1S/CACNB1/CACNB2/CACNB3/CACNB4/PTK2B | 17 |
| GO:0005506 | iron ion binding | 17/416 | 1.11E-07 | 7.57E-07 | CYP3A4/CYP1A2/CYP1A1/CYP1B1/ALOX5/CYP19A1/CYP17A1/PHF8/CYP2C19/TBXAS1/EGLN1/FTH1/FXN/HBA1/TF/FECH/CYP27B1 | 17 |
| GO:0004890 | GABA-A receptor activity | 16/416 | 5.94E-24 | 3.24E-22 | GABRA1/GABRA2/GABRA3/GABRA4/GABRA5/GABRA6/GABRG1/GABRG2/GABRG3/GABRB1/GABRB2/GABRB3/GABRD/GABRE/GABRP/GABRQ | 16 |
| GO:0016917 | GABA receptor activity | 16/416 | 4.28E-22 | 1.56E-20 | GABRA1/GABRA2/GABRA3/GABRA4/GABRA5/GABRA6/GABRG1/GABRG2/GABRG3/GABRB1/GABRB2/GABRB3/GABRD/GABRE/GABRP/GABRQ | 16 |
| GO:0004497 | monooxygenase activity | 16/416 | 1.24E-09 | 1.13E-08 | NOS2/CYP3A4/CYP1A2/CYP1A1/CYP1B1/AKR1C3/NOS3/CYP19A1/TYR/CYP17A1/CYP2C19/TBXAS1/AKR1C1/AKR1C2/AKR1D1/CYP27B1 | 16 |
| GO:0031072 | heat shock protein binding | 16/416 | 1.92E-08 | 1.55E-07 | KDR/BAX/AHSA1/CDK1/CYP1A1/AHR/HIF1A/HSPA5/HSF1/NR3C1/HSPA8/HDAC6/HDAC8/FGF1/CSNK2A1/HSPA2 | 16 |
| GO:0099095 | ligand-gated anion channel activity | 15/416 | 4.01E-21 | 1.10E-19 | GABRA1/GLRA1/GLRA2/GABRA2/GABRA3/GABRA4/GABRA5/GABRA6/GABRG1/GABRG2/GABRG3/GABRB1/GABRB2/GABRB3/GABRE | 15 |
| GO:0035257 | nuclear hormone receptor binding | 15/416 | 3.06E-06 | 1.61E-05 | RXRA/NCOA2/NCOA1/ESR1/PPARG/STAT1/RB1/HIF1A/PRKCB/PARP1/VDR/NR1H4/CEBPB/RARB/RARG | 15 |
| GO:0001085 | RNA polymerase II transcription factor binding | 15/416 | 3.90E-06 | 2.03E-05 | AR/ESR1/PPARG/GSK3B/PPARD/JUN/AHR/FOS/RB1/TP53/ELK1/NFE2L2/PPARA/HDAC1/RUVBL2 | 15 |
| GO:1901681 | sulfur compound binding | 15/416 | 0.000883833 | 0.002563286 | GSTP1/GSTM1/GSTM2/VEGFA/MPO/CXCL11/CXCL10/PCOLCE/FGF1/FGF2/GLRA1/PTGES/SOAT1/SOAT2/DBI | 15 |
| GO:0030374 | nuclear receptor transcription coactivator activity | 14/416 | 3.99E-10 | 3.70E-09 | NCOA2/NCOA1/PPARG/PPARD/NR1I2/NR1I3/PRKCB/PPARA/FGF2/VDR/NR1H4/RARA/RARB/RARG | 14 |
| GO:0015078 | proton transmembrane transporter activity | 14/416 | 3.05E-06 | 1.61E-05 | ATP5F1B/ATP2A1/SLC46A1/CYB5A/COX4I1/COX5A/COX5B/COX6A2/COX6B1/COX6C/COX7A1/COX7B/COX7C/COX8A | 14 |
| GO:0017171 | serine hydrolase activity | 14/416 | 0.000133107 | 0.000480552 | ACHE/DPP4/PRSS1/F7/MMP1/MMP3/PLAU/MMP2/MMP9/F3/PLAT/MMP8/CTSC/CTRB1 | 14 |
| GO:0016829 | lyase activity | 14/416 | 0.000140863 | 0.000504384 | CYP1A1/ODC1/CA2/CA1/CA7/CA12/CA14/CA9/CYP17A1/CA4/NEIL1/NEIL2/POLB/FECH | 14 |
| GO:0019207 | kinase regulator activity | 14/416 | 0.000401979 | 0.001249608 | CCNA2/PKIA/CASP3/GSTP1/CCND1/CDKN1A/EGF/HSPB1/IL2/CCNB1/CXCL10/IGF2/ERBB3/CSNK2B | 14 |
| GO:0005125 | cytokine activity | 14/416 | 0.000736326 | 0.002173365 | VEGFA/IL1B/CCL2/CXCL8/IL2/IFNG/IL1A/CXCL11/CXCL2/CXCL10/SPP1/CD40LG/FGF2/IL6 | 14 |
| GO:0022851 | GABA-gated chloride ion channel activity | 13/416 | 5.56E-22 | 1.87E-20 | GABRA1/GABRA2/GABRA3/GABRA4/GABRA5/GABRA6/GABRG1/GABRG2/GABRG3/GABRB1/GABRB2/GABRB3/GABRE | 13 |
| GO:0005237 | inhibitory extracellular ligand-gated ion channel activity | 13/416 | 2.92E-19 | 6.38E-18 | GABRA1/GLRA1/GLRA2/GABRA2/GABRA3/GABRA4/GABRA5/GABRA6/GABRG1/GABRG2/GABRG3/GABRB2/GABRE | 13 |
| GO:0099094 | ligand-gated cation channel activity | 13/416 | 1.09E-06 | 6.36E-06 | GRIA2/HTR3A/KCNH2/TRPA1/ITPR1/GRIN1/GRIN2A/GRIN2B/GRIN2C/GRIN2D/GRIN3A/TRPV1/PTK2B | 13 |
| GO:0042826 | histone deacetylase binding | 13/416 | 2.07E-06 | 1.14E-05 | RELA/MAPK8/CCND1/TP53/HIF1A/TOP2A/PARP1/HDAC6/HDAC1/HSP90AA1/CEBPB/RARA/HOXA10 | 13 |
| GO:0005178 | integrin binding | 13/416 | 1.42E-05 | 6.60E-05 | KDR/ICAM1/VCAM1/EGFR/PRKCA/IL1B/COL3A1/SPP1/IGF2/FGF1/FGF2/LGALS8/PTPN2 | 13 |
| GO:0004252 | serine-type endopeptidase activity | 13/416 | 0.000106926 | 0.000399228 | DPP4/PRSS1/F7/MMP1/MMP3/PLAU/MMP2/MMP9/F3/PLAT/MMP8/CTSC/CTRB1 | 13 |
| GO:0008236 | serine-type peptidase activity | 13/416 | 0.000381804 | 0.00120861 | DPP4/PRSS1/F7/MMP1/MMP3/PLAU/MMP2/MMP9/F3/PLAT/MMP8/CTSC/CTRB1 | 13 |
| GO:0016675 | oxidoreductase activity, acting on a heme group of donors | 12/416 | 8.80E-13 | 1.16E-11 | POR/CYB5A/COX4I1/COX5A/COX5B/COX6A2/COX6B1/COX6C/COX7A1/COX7B/COX7C/COX8A | 12 |
| GO:0016229 | steroid dehydrogenase activity | 12/416 | 1.24E-11 | 1.39E-10 | HSD3B2/HSD3B1/AKR1C3/HSD11B1/SRD5A1/SRD5A2/AKR1C1/AKR1C2/HSD11B2/HSD17B1/AKR1D1/HSD17B11 | 12 |
| GO:0008227 | G protein-coupled amine receptor activity | 12/416 | 1.00E-08 | 8.59E-08 | CHRM3/CHRM1/ADRA1A/CHRM2/ADRB2/ADRA1B/ADRB1/ADRA2C/ADRA1D/CHRM5/CHRM4/ADRB3 | 12 |
| GO:0042562 | hormone binding | 12/416 | 4.87E-06 | 2.44E-05 | CHRM3/AR/ACHE/INSR/EGFR/NR3C1/SHBG/NQO2/VDR/HSD17B1/SULT2B1/GPER1 | 12 |
| GO:0016651 | oxidoreductase activity, acting on NAD(P)H | 12/416 | 8.04E-06 | 3.82E-05 | NOS2/NCF1/AKR1C3/POR/DUOX2/NOS3/NQO1/NQO2/NOX4/AKR1C1/AKR1C2/CBR1 | 12 |
| GO:0004713 | protein tyrosine kinase activity | 12/416 | 7.73E-05 | 0.000304209 | KDR/MET/INSR/EGFR/ERBB2/ERBB3/NEK1/JAK3/JAK1/JAK2/HCK/PTK2B | 12 |
| GO:0019887 | protein kinase regulator activity | 12/416 | 0.001160564 | 0.003229193 | CCNA2/PKIA/CASP3/CCND1/CDKN1A/EGF/HSPB1/CCNB1/CXCL10/IGF2/ERBB3/CSNK2B | 12 |
| GO:0004129 | cytochrome-c oxidase activity | 11/416 | 1.59E-11 | 1.66E-10 | CYB5A/COX4I1/COX5A/COX5B/COX6A2/COX6B1/COX6C/COX7A1/COX7B/COX7C/COX8A | 11 |
| GO:0015002 | heme-copper terminal oxidase activity | 11/416 | 1.59E-11 | 1.66E-10 | CYB5A/COX4I1/COX5A/COX5B/COX6A2/COX6B1/COX6C/COX7A1/COX7B/COX7C/COX8A | 11 |
| GO:0016676 | oxidoreductase activity, acting on a heme group of donors, oxygen as acceptor | 11/416 | 1.59E-11 | 1.66E-10 | CYB5A/COX4I1/COX5A/COX5B/COX6A2/COX6B1/COX6C/COX7A1/COX7B/COX7C/COX8A | 11 |
| GO:0072341 | modified amino acid binding | 11/416 | 7.47E-06 | 3.63E-05 | DPEP1/GSTP1/GSTM1/GSTM2/TYMS/DHFR/FOLR1/FOLR2/SLC46A1/NOX4/PTGES | 11 |
| GO:0035326 | enhancer binding | 11/416 | 0.000307155 | 0.001008859 | RELA/JUN/AHR/TP53/NFE2L2/HDAC1/NFKB1/NR1H4/ACTB/ESRRB/RUVBL2 | 11 |
| GO:0019838 | growth factor binding | 11/416 | 0.000396455 | 0.001245959 | SCN5A/KDR/INSR/EGFR/IL6ST/ERBB2/COL1A1/COL3A1/IGFBP3/ERBB3/TGFBR2 | 11 |
| GO:0005516 | calmodulin binding | 11/416 | 0.007863723 | 0.015903728 | SCN5A/NOS2/PCP4/AKT1/PPP3CA/EGFR/NOS3/GRIN1/CACNA1C/CACNA1S/TRPV1 | 11 |
| GO:0001227 | DNA-binding transcription repressor activity, RNA polymerase II-specific | 11/416 | 0.028433575 | 0.045332053 | PPARG/PPARD/RELA/MYC/PPARA/HSF1/E2F1/RORC/NFKB1/CEBPB/ESRRA | 11 |
| GO:0008503 | benzodiazepine receptor activity | 10/416 | 4.99E-16 | 8.07E-15 | GABRA1/GABRA2/GABRA3/GABRA4/GABRA5/GABRA6/GABRG1/GABRG2/GABRG3/GABRE | 10 |
| GO:0008066 | glutamate receptor activity | 10/416 | 2.73E-10 | 2.65E-09 | GRIA2/GRM4/GRIN1/GRIN2A/GRIN2B/GRIN2C/GRIN2D/GRIN3A/GRIN3B/PTK2B | 10 |
| GO:0051879 | Hsp90 protein binding | 10/416 | 2.70E-08 | 2.11E-07 | KDR/AHSA1/CYP1A1/AHR/HIF1A/HSF1/NR3C1/HDAC6/HDAC8/CSNK2A1 | 10 |
| GO:0042165 | neurotransmitter binding | 10/416 | 5.26E-07 | 3.28E-06 | CHRM3/ACHE/HTR3A/SLC6A4/GLRA1/GLRA2/GRIN1/GRIN2B/GRIN3A/GRIN3B | 10 |
| GO:0033293 | monocarboxylic acid binding | 10/416 | 2.26E-06 | 1.23E-05 | RXRA/PPARG/PPARD/PPARA/VDR/AKR1C1/AKR1C2/NR1H4/RARA/PLA2G1B | 10 |
| GO:0051213 | dioxygenase activity | 10/416 | 5.45E-05 | 0.000230989 | PTGS2/PTGS1/ALOX5/POR/KDM4E/KDM2A/KDM6B/PHF8/KDM5C/EGLN1 | 10 |
| GO:0000980 | RNA polymerase II distal enhancer sequence-specific DNA binding | 10/416 | 0.000111772 | 0.000410309 | RELA/JUN/TP53/NFE2L2/HDAC1/NFKB1/NR1H4/ACTB/ESRRB/RUVBL2 | 10 |
| GO:0051087 | chaperone binding | 10/416 | 0.00014358 | 0.000509932 | BAX/AHSA1/TP53/SOD1/HSPA5/BIRC5/HSPA8/CTSC/CP/HSPA2 | 10 |
| GO:0001158 | enhancer sequence-specific DNA binding | 10/416 | 0.000503972 | 0.001528864 | RELA/JUN/TP53/NFE2L2/HDAC1/NFKB1/NR1H4/ACTB/ESRRB/RUVBL2 | 10 |
| GO:0002020 | protease binding | 10/416 | 0.0008919 | 0.002563286 | DPP4/GSK3B/SLC6A3/BCL2/CASP3/TP53/F3/SERPINE1/COL1A1/COL3A1 | 10 |
| GO:0070851 | growth factor receptor binding | 10/416 | 0.001266501 | 0.00347963 | VEGFA/EGF/IL6ST/IL1B/IL2/IL1A/JAK2/FGF1/FGF2/IL6 | 10 |
| GO:0052689 | carboxylic ester hydrolase activity | 10/416 | 0.001416659 | 0.003750644 | ACHE/PON1/CA2/CA1/NOTUM/CES2/PLA2G2E/AADACL2/CES1/PLA2G1B | 10 |
| GO:0019842 | vitamin binding | 10/416 | 0.001580981 | 0.004160477 | PYGM/GC/TYMS/DHFR/FOLR1/FOLR2/SLC46A1/VDR/EGLN1/RHO | 10 |
| GO:0004970 | ionotropic glutamate receptor activity | 9/416 | 1.51E-10 | 1.50E-09 | GRIA2/GRIN1/GRIN2A/GRIN2B/GRIN2C/GRIN2D/GRIN3A/GRIN3B/PTK2B | 9 |
| GO:0033764 | steroid dehydrogenase activity, acting on the CH-OH group of donors, NAD or NADP as acceptor | 9/416 | 1.33E-08 | 1.12E-07 | HSD3B2/HSD3B1/AKR1C3/HSD11B1/SRD5A2/AKR1C1/HSD11B2/HSD17B1/HSD17B11 | 9 |
| GO:0019825 | oxygen binding | 9/416 | 1.08E-07 | 7.48E-07 | CYP3A4/CYP1A1/CYP1B1/CYP19A1/CYP17A1/NOX4/CYP2C19/HBA1/MB | 9 |
| GO:0005245 | voltage-gated calcium channel activity | 9/416 | 1.50E-06 | 8.60E-06 | OPRM1/CACNA1C/CACNA1D/CACNA1F/CACNA1S/CACNB1/CACNB2/CACNB3/CACNB4 | 9 |
| GO:0016835 | carbon-oxygen lyase activity | 9/416 | 6.40E-05 | 0.000261456 | CA2/CA1/CA7/CA12/CA14/CA9/CA4/NEIL1/NEIL2 | 9 |
| GO:0016209 | antioxidant activity | 9/416 | 0.000186852 | 0.000647817 | PTGS2/PTGS1/GSTP1/GSTM2/SOD1/DUOX2/MPO/NQO1/HBA1 | 9 |
| GO:0016298 | lipase activity | 9/416 | 0.003105783 | 0.007413862 | CHRM3/CHRM1/CHRM5/HMOX1/CCR1/NOTUM/PLA2G2E/CES1/PLA2G1B | 9 |
| GO:0016853 | isomerase activity | 9/416 | 0.012519662 | 0.023278106 | HSD3B2/HSD3B1/TOP1/TOP2A/TBXAS1/PTGES/HPGDS/ISYNA1/LSS | 9 |
| GO:0008331 | high voltage-gated calcium channel activity | 8/416 | 3.77E-12 | 4.45E-11 | CACNA1C/CACNA1D/CACNA1F/CACNA1S/CACNB1/CACNB2/CACNB3/CACNB4 | 8 |
| GO:0099604 | ligand-gated calcium channel activity | 8/416 | 1.31E-07 | 8.79E-07 | TRPA1/ITPR1/GRIN1/GRIN2A/GRIN2B/GRIN2C/GRIN2D/TRPV1 | 8 |
| GO:0051721 | protein phosphatase 2A binding | 8/416 | 5.59E-07 | 3.44E-06 | SLC6A3/AKT1/BCL2/STAT1/TP53/PTPN1/HMGCR/GRIN3A | 8 |
| GO:0016709 | oxidoreductase activity, acting on paired donors, with incorporation or reduction of molecular oxygen, NAD(P)H as one donor, and incorporation of one atom of oxygen | 8/416 | 2.83E-06 | 1.53E-05 | NOS2/CYP1A1/AKR1C3/NOS3/AKR1C1/AKR1C2/AKR1D1/CYP27B1 | 8 |
| GO:0050661 | NADP binding | 8/416 | 2.65E-05 | 0.000116984 | NOS2/POR/NOS3/DHFR/HMGCR/SRD5A1/HSD17B1/G6PD | 8 |
| GO:0001047 | core promoter binding | 8/416 | 4.03E-05 | 0.000176206 | STAT1/FOS/TP53/MYC/NR3C1/HDAC1/CEBPB/RUVBL2 | 8 |
| GO:0016597 | amino acid binding | 8/416 | 6.77E-05 | 0.000271261 | NOS2/NOS3/GLRA1/GLRA2/GRIN1/GRIN2B/GRIN3A/GRIN3B | 8 |
| GO:0097110 | scaffold protein binding | 8/416 | 6.77E-05 | 0.000271261 | SCN5A/KCNH2/IKBKB/CASP8/GJA1/NOS3/CHUK/HSP90AA1 | 8 |
| GO:0070491 | repressing transcription factor binding | 8/416 | 0.000254126 | 0.000847428 | PPARG/PPARD/RELA/STAT1/MYC/PPARA/RUNX2/HDAC1 | 8 |
| GO:0001540 | amyloid-beta binding | 8/416 | 0.000485599 | 0.001483426 | ACHE/ADRB2/GRIA2/INSR/BACE1/GRIN1/GRIN2A/GRIN2B | 8 |
| GO:0033613 | activating transcription factor binding | 8/416 | 0.000864438 | 0.002534383 | PPARG/RELA/JUN/FOS/RB1/MYC/NFE2L2/HDAC1 | 8 |
| GO:0004620 | phospholipase activity | 8/416 | 0.002791274 | 0.006889732 | CHRM3/CHRM1/CHRM5/HMOX1/CCR1/NOTUM/PLA2G2E/PLA2G1B | 8 |
| GO:0031490 | chromatin DNA binding | 8/416 | 0.007086945 | 0.014399424 | RXRA/RELA/HSF1/HDAC1/KDM6B/ACTB/RUVBL2/RARA | 8 |
| GO:0016247 | channel regulator activity | 8/416 | 0.020620269 | 0.036031207 | ADRB2/BCL2/CAV1/PRKCB/RASA1/ITPR1/CACNB3/TRPV1 | 8 |
| GO:0004089 | carbonate dehydratase activity | 7/416 | 2.06E-08 | 1.64E-07 | CA2/CA1/CA7/CA12/CA14/CA9/CA4 | 7 |
| GO:0004935 | adrenergic receptor activity | 7/416 | 5.98E-08 | 4.22E-07 | ADRA1A/ADRB2/ADRA1B/ADRB1/ADRA2C/ADRA1D/ADRB3 | 7 |
| GO:0035173 | histone kinase activity | 7/416 | 5.98E-08 | 4.22E-07 | CDK2/CHEK1/CDK1/PRKCA/PRKCB/CCNB1/JAK2 | 7 |
| GO:0016628 | oxidoreductase activity, acting on the CH-CH group of donors, NAD or NADP as acceptor | 7/416 | 1.69E-06 | 9.44E-06 | AKR1C3/SRD5A1/SRD5A2/TBXAS1/AKR1C1/AKR1C2/AKR1D1 | 7 |
| GO:0032451 | demethylase activity | 7/416 | 2.11E-05 | 9.60E-05 | CYP1A2/CYP1A1/KDM4E/KDM2A/KDM6B/PHF8/KDM5C | 7 |
| GO:0008395 | steroid hydroxylase activity | 7/416 | 2.53E-05 | 0.000112993 | CYP3A4/CYP1A2/CYP1A1/CYP1B1/CYP19A1/CYP17A1/CYP2C19 | 7 |
| GO:0099528 | G protein-coupled neurotransmitter receptor activity | 7/416 | 4.25E-05 | 0.000183802 | CHRM3/CHRM1/CHRM2/OPRM1/ADRB1/CHRM5/CHRM4 | 7 |
| GO:0001221 | transcription cofactor binding | 7/416 | 5.85E-05 | 0.000241072 | ESR1/RELA/AHR/NFE2L2/PPARA/RORC/RORA | 7 |
| GO:0004601 | peroxidase activity | 7/416 | 0.00020251 | 0.000691133 | PTGS2/PTGS1/GSTP1/GSTM2/DUOX2/MPO/HBA1 | 7 |
| GO:0016684 | oxidoreductase activity, acting on peroxide as acceptor | 7/416 | 0.000323879 | 0.00104803 | PTGS2/PTGS1/GSTP1/GSTM2/DUOX2/MPO/HBA1 | 7 |
| GO:0016836 | hydro-lyase activity | 7/416 | 0.000323879 | 0.00104803 | CA2/CA1/CA7/CA12/CA14/CA9/CA4 | 7 |
| GO:0016627 | oxidoreductase activity, acting on the CH-CH group of donors | 7/416 | 0.000403337 | 0.001249608 | AKR1C3/SRD5A1/SRD5A2/TBXAS1/AKR1C1/AKR1C2/AKR1D1 | 7 |
| GO:0042379 | chemokine receptor binding | 7/416 | 0.000889629 | 0.002563286 | STAT1/CCL2/CXCL8/CXCL11/CXCL2/CXCL10/JAK1 | 7 |
| GO:0019199 | transmembrane receptor protein kinase activity | 7/416 | 0.002552956 | 0.006372792 | KDR/MET/INSR/EGFR/ERBB2/ERBB3/TGFBR2 | 7 |
| GO:0008013 | beta-catenin binding | 7/416 | 0.003153767 | 0.00748749 | AR/ESR1/GSK3B/HDAC6/KDM6B/RORA/RUVBL2 | 7 |
| GO:0051219 | phosphoprotein binding | 7/416 | 0.003376282 | 0.007929582 | MAPK1/RB1/PLAT/RASA1/VAV1/TRPV1/HCK | 7 |
| GO:0035258 | steroid hormone receptor binding | 7/416 | 0.005956348 | 0.012630989 | NCOA1/ESR1/PPARG/RB1/PRKCB/PARP1/CEBPB | 7 |
| GO:0008081 | phosphoric diester hydrolase activity | 7/416 | 0.006688617 | 0.013980238 | CHRM3/CHRM1/CHRM5/HMOX1/CCR1/TDP1/NOTUM | 7 |
| GO:0047485 | protein N-terminus binding | 7/416 | 0.014473515 | 0.026235023 | NCOA1/RELA/SLC6A3/TP53/PARP1/HDAC1/CSNK2A1 | 7 |
| GO:0004197 | cysteine-type endopeptidase activity | 7/416 | 0.019734683 | 0.034902593 | CASP3/CASP9/CASP8/CTSK/CTSS/CTSC/CTSF | 7 |
| GO:0016594 | glycine binding | 6/416 | 6.81E-07 | 4.13E-06 | GLRA1/GLRA2/GRIN1/GRIN2B/GRIN3A/GRIN3B | 6 |
| GO:0046965 | retinoid X receptor binding | 6/416 | 1.62E-06 | 9.17E-06 | NCOA1/PPARG/VDR/NR1H4/RARB/RARG | 6 |
| GO:1901338 | catecholamine binding | 6/416 | 4.77E-06 | 2.44E-05 | ADRB2/ADRB1/ADRA2C/DRD1/SLC6A3/ADRB3 | 6 |
| GO:0001223 | transcription coactivator binding | 6/416 | 1.17E-05 | 5.50E-05 | ESR1/RELA/AHR/PPARA/RORC/RORA | 6 |
| GO:0042974 | retinoic acid receptor binding | 6/416 | 2.51E-05 | 0.000112993 | NCOA1/PPARG/VDR/NR1H4/RARB/RARG | 6 |
| GO:0016712 | oxidoreductase activity, acting on paired donors, with incorporation or reduction of molecular oxygen, reduced flavin or flavoprotein as one donor, and incorporation of one atom of oxygen | 6/416 | 8.78E-05 | 0.000336498 | CYP3A4/CYP1A2/CYP1A1/CYP1B1/CYP19A1/CYP2C19 | 6 |
| GO:0097718 | disordered domain specific binding | 6/416 | 0.000105218 | 0.000396238 | HSP90AA2P/RB1/TP53/GJA1/HSP90AA1/HSPA2 | 6 |
| GO:0001046 | core promoter sequence-specific DNA binding | 6/416 | 0.000610128 | 0.001825545 | STAT1/FOS/TP53/HDAC1/CEBPB/RUVBL2 | 6 |
| GO:0042805 | actinin binding | 6/416 | 0.00068793 | 0.002044332 | PPARG/RELA/NFKB1/CACNA1C/CACNA1D/RARA | 6 |
| GO:0032934 | sterol binding | 6/416 | 0.001958331 | 0.004973728 | CAV1/RORC/SOAT1/SOAT2/SULT2B1/RORA | 6 |
| GO:0016655 | oxidoreductase activity, acting on NAD(P)H, quinone or similar compound as acceptor | 6/416 | 0.002791587 | 0.006889732 | AKR1C3/NQO1/NQO2/AKR1C1/AKR1C2/CBR1 | 6 |
| GO:0004714 | transmembrane receptor protein tyrosine kinase activity | 6/416 | 0.00329608 | 0.007783063 | KDR/MET/INSR/EGFR/ERBB2/ERBB3 | 6 |
| GO:0001098 | basal transcription machinery binding | 6/416 | 0.006890093 | 0.014064873 | AR/ESR1/AHR/TP53/ESRRB/RUVBL2 | 6 |
| GO:0001099 | basal RNA polymerase II transcription machinery binding | 6/416 | 0.006890093 | 0.014064873 | AR/ESR1/AHR/TP53/ESRRB/RUVBL2 | 6 |
| GO:0015370 | solute:sodium symporter activity | 6/416 | 0.006890093 | 0.014064873 | SLC6A2/SLC6A3/SLC6A4/SLC5A2/SLC5A1/SLC28A3 | 6 |
| GO:0070405 | ammonium ion binding | 6/416 | 0.008375918 | 0.016861537 | CHRM3/ACHE/HTR3A/DRD1/SLC6A3/SLC6A4 | 6 |
| GO:0050660 | flavin adenine dinucleotide binding | 6/416 | 0.010697304 | 0.020923835 | NOS2/MAOB/POR/NOS3/NQO2/NOX4 | 6 |
| GO:0019209 | kinase activator activity | 6/416 | 0.015797966 | 0.028517424 | CDKN1A/EGF/IL2/CCNB1/IGF2/ERBB3 | 6 |
| GO:1990782 | protein tyrosine kinase binding | 6/416 | 0.022374532 | 0.03730369 | TP53/GJA1/PTPN1/HSP90AA1/NOX4/PTPN2 | 6 |
| GO:0005542 | folic acid binding | 5/416 | 4.84E-06 | 2.44E-05 | TYMS/DHFR/FOLR1/FOLR2/SLC46A1 | 5 |
| GO:0004032 | alditol:NADP+ 1-oxidoreductase activity | 5/416 | 7.72E-06 | 3.71E-05 | AKR1B1/AKR1C3/AKR1C1/AKR1C2/AKR1D1 | 5 |
| GO:0001091 | RNA polymerase II basal transcription factor binding | 5/416 | 8.12E-05 | 0.000316527 | AR/ESR1/AHR/TP53/RUVBL2 | 5 |
| GO:0008106 | alcohol dehydrogenase (NADP+) activity | 5/416 | 0.000104468 | 0.000396238 | AKR1B1/AKR1C3/AKR1C1/AKR1C2/AKR1D1 | 5 |
| GO:0015464 | acetylcholine receptor activity | 5/416 | 0.000166181 | 0.000585443 | CHRM3/CHRM1/CHRM2/CHRM5/CHRM4 | 5 |
| GO:0008198 | ferrous iron binding | 5/416 | 0.000205889 | 0.000697216 | EGLN1/FTH1/FXN/TF/FECH | 5 |
| GO:0004033 | aldo-keto reductase (NADP) activity | 5/416 | 0.000306547 | 0.001008859 | AKR1B1/AKR1C3/AKR1C1/AKR1C2/AKR1D1 | 5 |
| GO:0004364 | glutathione transferase activity | 5/416 | 0.000369016 | 0.001176654 | GSTP1/GSTM1/GSTM2/PTGES/HPGDS | 5 |
| GO:0032452 | histone demethylase activity | 5/416 | 0.000369016 | 0.001176654 | KDM4E/KDM2A/KDM6B/PHF8/KDM5C | 5 |
| GO:0004629 | phospholipase C activity | 5/416 | 0.000440643 | 0.001355574 | CHRM3/CHRM1/CHRM5/CCR1/NOTUM | 5 |
| GO:0051059 | NF-kappaB binding | 5/416 | 0.000522265 | 0.00157343 | GSK3B/PPARD/RELA/NFKBIA/HDAC1 | 5 |
| GO:0000979 | RNA polymerase II core promoter sequence-specific DNA binding | 5/416 | 0.001111328 | 0.003112018 | STAT1/FOS/HDAC1/CEBPB/RUVBL2 | 5 |
| GO:0004993 | G protein-coupled serotonin receptor activity | 5/416 | 0.001111328 | 0.003112018 | CHRM3/CHRM1/CHRM2/CHRM5/CHRM4 | 5 |
| GO:0099589 | serotonin receptor activity | 5/416 | 0.001111328 | 0.003112018 | CHRM3/CHRM1/CHRM2/CHRM5/CHRM4 | 5 |
| GO:0030544 | Hsp70 protein binding | 5/416 | 0.002913893 | 0.007151187 | BAX/CDK1/CYP1A1/HDAC8/FGF1 | 5 |
| GO:0019213 | deacetylase activity | 5/416 | 0.003580363 | 0.008319431 | HDAC6/HDAC8/HDAC1/CES2/CES1 | 5 |
| GO:0048156 | tau protein binding | 5/416 | 0.003951642 | 0.009037924 | GSK3B/HDAC6/HSP90AA1/ACTB/HSPA2 | 5 |
| GO:0004715 | non-membrane spanning protein tyrosine kinase activity | 5/416 | 0.004349656 | 0.00989642 | JAK3/JAK1/JAK2/HCK/PTK2B | 5 |
| GO:0016860 | intramolecular oxidoreductase activity | 5/416 | 0.004775425 | 0.010589374 | HSD3B2/HSD3B1/TBXAS1/PTGES/HPGDS | 5 |
| GO:0008009 | chemokine activity | 5/416 | 0.005714277 | 0.012253635 | CCL2/CXCL8/CXCL11/CXCL2/CXCL10 | 5 |
| GO:0016538 | cyclin-dependent protein serine/threonine kinase regulator activity | 5/416 | 0.005714277 | 0.012253635 | CCNA2/CASP3/CCND1/CDKN1A/CCNB1 | 5 |
| GO:0070888 | E-box binding | 5/416 | 0.006229359 | 0.013146118 | PPARG/AHR/HIF1A/MYC/HDAC1 | 5 |
| GO:0043621 | protein self-association | 5/416 | 0.01001994 | 0.019806026 | ACHE/PPARG/TP53/HSF1/CTSC | 5 |
| GO:0050840 | extracellular matrix binding | 5/416 | 0.010777035 | 0.020923835 | ACHE/VEGFA/SPP1/CTSS/LGALS3 | 5 |
| GO:0016765 | transferase activity, transferring alkyl or aryl (other than methyl) groups | 5/416 | 0.011572299 | 0.022075403 | GSTP1/GSTM1/GSTM2/PTGES/HPGDS | 5 |
| GO:0005507 | copper ion binding | 5/416 | 0.012406568 | 0.023278106 | TP53/SOD1/IL1A/TYR/CP | 5 |
| GO:0005518 | collagen binding | 5/416 | 0.02057746 | 0.036031207 | ACHE/MMP9/PCOLCE/CTSK/CTSS | 5 |
| GO:0001618 | virus receptor activity | 5/416 | 0.030093952 | 0.047119374 | DPP4/CDK1/ICAM1/EGFR/TFRC | 5 |
| GO:0104005 | hijacked molecular function | 5/416 | 0.030093952 | 0.047119374 | DPP4/CDK1/ICAM1/EGFR/TFRC | 5 |
| GO:0051400 | BH domain binding | 4/416 | 5.65E-05 | 0.000235011 | BCL2/BAX/BCL2L1/MCL1 | 4 |
| GO:0070513 | death domain binding | 4/416 | 5.65E-05 | 0.000235011 | BCL2/BAX/BCL2L1/MCL1 | 4 |
| GO:0043295 | glutathione binding | 4/416 | 8.71E-05 | 0.000336498 | GSTP1/GSTM1/GSTM2/PTGES | 4 |
| GO:1900750 | oligopeptide binding | 4/416 | 0.000128258 | 0.000466905 | GSTP1/GSTM1/GSTM2/PTGES | 4 |
| GO:0016653 | oxidoreductase activity, acting on NAD(P)H, heme protein as acceptor | 4/416 | 0.000181832 | 0.000635454 | NOS2/POR/NOS3/NQO1 | 4 |
| GO:0050998 | nitric-oxide synthase binding | 4/416 | 0.000249854 | 0.000839591 | SCN5A/SLC6A4/CAV1/ACTB | 4 |
| GO:0016922 | nuclear receptor binding | 4/416 | 0.001081481 | 0.003087819 | RXRA/NCOA2/NCOA1/NR1H4 | 4 |
| GO:0044183 | protein binding involved in protein folding | 4/416 | 0.001869553 | 0.00477602 | HSPA5/HSPB1/HSPA8/HSPA2 | 4 |
| GO:0042923 | neuropeptide binding | 4/416 | 0.002202286 | 0.005560989 | OPRM1/OPRD1/NMUR2/OPRK1 | 4 |
| GO:0004435 | phosphatidylinositol phospholipase C activity | 4/416 | 0.002985894 | 0.007246468 | CHRM3/CHRM1/CHRM5/CCR1 | 4 |
| GO:0051787 | misfolded protein binding | 4/416 | 0.002985894 | 0.007246468 | HSPA5/HSPA8/HDAC6/HSPA2 | 4 |
| GO:0001968 | fibronectin binding | 4/416 | 0.003441003 | 0.008038369 | VEGFA/IGFBP3/CTSK/CTSS | 4 |
| GO:1901567 | fatty acid derivative binding | 4/416 | 0.003941063 | 0.009037924 | PPARG/SOAT1/SOAT2/DBI | 4 |
| GO:0035035 | histone acetyltransferase binding | 4/416 | 0.004488071 | 0.010158437 | STAT1/TP53/HIF1A/CEBPB | 4 |
| GO:0030332 | cyclin binding | 4/416 | 0.005083967 | 0.011160256 | CDK2/CDK1/CDKN1A/CDK6 | 4 |
| GO:0097472 | cyclin-dependent protein kinase activity | 4/416 | 0.005083967 | 0.011160256 | CDK2/CCNA2/CDK1/CDK6 | 4 |
| GO:0030291 | protein serine/threonine kinase inhibitor activity | 4/416 | 0.006429889 | 0.013504069 | PKIA/CASP3/CDKN1A/HSPB1 | 4 |
| GO:0001103 | RNA polymerase II repressing transcription factor binding | 4/416 | 0.008860365 | 0.017754957 | PPARG/PPARD/PPARA/HDAC1 | 4 |
| GO:0051393 | alpha-actinin binding | 4/416 | 0.010773885 | 0.020923835 | PPARG/CACNA1C/CACNA1D/RARA | 4 |
| GO:0016248 | channel inhibitor activity | 4/416 | 0.011823002 | 0.022358378 | BCL2/CAV1/RASA1/ITPR1 | 4 |
| GO:0042056 | chemoattractant activity | 4/416 | 0.011823002 | 0.022358378 | VEGFA/CXCL10/FGF2/LGALS3 | 4 |
| GO:0001784 | phosphotyrosine residue binding | 4/416 | 0.014112523 | 0.025939899 | MAPK1/RASA1/VAV1/HCK | 4 |
| GO:0030331 | estrogen receptor binding | 4/416 | 0.016664945 | 0.029835859 | NCOA1/ESR1/PPARG/PARP1 | 4 |
| GO:0032813 | tumor necrosis factor receptor superfamily binding | 4/416 | 0.022590567 | 0.037380723 | CASP3/STAT1/CASP8/CD40LG | 4 |
| GO:1990841 | promoter-specific chromatin binding | 4/416 | 0.025976683 | 0.042631233 | STAT1/TP53/HSF1/HDAC1 | 4 |
| GO:0015485 | cholesterol binding | 4/416 | 0.027777785 | 0.044448741 | CAV1/SOAT1/SOAT2/SULT2B1 | 4 |
| GO:0005326 | neurotransmitter transporter activity | 4/416 | 0.031598402 | 0.048095862 | SLC6A2/SLC6A3/SLC6A4/GABRQ | 4 |
| GO:0045309 | protein phosphorylated amino acid binding | 4/416 | 0.031598402 | 0.048095862 | MAPK1/RASA1/VAV1/HCK | 4 |
| GO:0004322 | ferroxidase activity | 3/416 | 0.001368341 | 0.00364481 | CP/FTH1/FXN | 3 |
| GO:0015378 | sodium:chloride symporter activity | 3/416 | 0.001368341 | 0.00364481 | SLC6A2/SLC6A3/SLC6A4 | 3 |
| GO:0016724 | oxidoreductase activity, oxidizing metal ions, oxygen as acceptor | 3/416 | 0.001368341 | 0.00364481 | CP/FTH1/FXN | 3 |
| GO:0031078 | histone deacetylase activity (H3-K14 specific) | 3/416 | 0.001368341 | 0.00364481 | HDAC6/HDAC8/HDAC1 | 3 |
| GO:0032041 | NAD-dependent histone deacetylase activity (H3-K14 specific) | 3/416 | 0.001368341 | 0.00364481 | HDAC6/HDAC8/HDAC1 | 3 |
| GO:0001094 | TFIID-class transcription factor complex binding | 3/416 | 0.001848799 | 0.004750783 | AHR/TP53/RUVBL2 | 3 |
| GO:0005536 | glucose binding | 3/416 | 0.001848799 | 0.004750783 | HK2/GCK/G6PD | 3 |
| GO:0008199 | ferric iron binding | 3/416 | 0.001848799 | 0.004750783 | FTH1/FXN/TF | 3 |
| GO:0045236 | CXCR chemokine receptor binding | 3/416 | 0.001848799 | 0.004750783 | CXCL8/CXCL11/CXCL10 | 3 |
| GO:0016175 | superoxide-generating NADPH oxidase activity | 3/416 | 0.002422312 | 0.006081425 | NCF1/DUOX2/NOX4 | 3 |
| GO:0003906 | DNA-(apurinic or apyrimidinic site) endonuclease activity | 3/416 | 0.003094456 | 0.007413862 | NEIL1/NEIL2/POLB | 3 |
| GO:0015373 | anion:sodium symporter activity | 3/416 | 0.003094456 | 0.007413862 | SLC6A2/SLC6A3/SLC6A4 | 3 |
| GO:0004707 | MAP kinase activity | 3/416 | 0.003870257 | 0.008945456 | MAPK14/MAPK8/MAPK1 | 3 |
| GO:0000900 | translation repressor activity, mRNA regulatory element binding | 3/416 | 0.004754218 | 0.010589374 | TYMS/DHFR/RARA | 3 |
| GO:0017136 | NAD-dependent histone deacetylase activity | 3/416 | 0.004754218 | 0.010589374 | HDAC6/HDAC8/HDAC1 | 3 |
| GO:0097153 | cysteine-type endopeptidase activity involved in apoptotic process | 3/416 | 0.004754218 | 0.010589374 | CASP3/CASP9/CASP8 | 3 |
| GO:0010181 | FMN binding | 3/416 | 0.00575035 | 0.012253635 | NOS2/POR/NOS3 | 3 |
| GO:0015377 | cation:chloride symporter activity | 3/416 | 0.00575035 | 0.012253635 | SLC6A2/SLC6A3/SLC6A4 | 3 |
| GO:0034979 | NAD-dependent protein deacetylase activity | 3/416 | 0.00575035 | 0.012253635 | HDAC6/HDAC8/HDAC1 | 3 |
| GO:0070402 | NADPH binding | 3/416 | 0.00575035 | 0.012253635 | DHFR/HMGCR/SRD5A1 | 3 |
| GO:0050664 | oxidoreductase activity, acting on NAD(P)H, oxygen as acceptor | 3/416 | 0.006862195 | 0.014064873 | NCF1/DUOX2/NOX4 | 3 |
| GO:0050811 | GABA receptor binding | 3/416 | 0.006862195 | 0.014064873 | GABRA5/GABRG1/GABRB1 | 3 |
| GO:0015296 | anion:cation symporter activity | 3/416 | 0.009444995 | 0.018754415 | SLC6A2/SLC6A3/SLC6A4 | 3 |
| GO:0016722 | oxidoreductase activity, oxidizing metal ions | 3/416 | 0.009444995 | 0.018754415 | CP/FTH1/FXN | 3 |
| GO:0004745 | retinol dehydrogenase activity | 3/416 | 0.010920909 | 0.020924179 | ADH1B/ADH1C/AKR1C3 | 3 |
| GO:0005355 | glucose transmembrane transporter activity | 3/416 | 0.010920909 | 0.020924179 | SLC2A4/SLC5A2/SLC5A1 | 3 |
| GO:0015149 | hexose transmembrane transporter activity | 3/416 | 0.010920909 | 0.020924179 | SLC2A4/SLC5A2/SLC5A1 | 3 |
| GO:0015278 | calcium-release channel activity | 3/416 | 0.012522499 | 0.023278106 | TRPA1/ITPR1/TRPV1 | 3 |
| GO:0051861 | glycolipid binding | 3/416 | 0.012522499 | 0.023278106 | DPEP1/IL2/HSPA2 | 3 |
| GO:0000062 | fatty-acyl-CoA binding | 3/416 | 0.014251318 | 0.025939899 | SOAT1/SOAT2/DBI | 3 |
| GO:0015145 | monosaccharide transmembrane transporter activity | 3/416 | 0.014251318 | 0.025939899 | SLC2A4/SLC5A2/SLC5A1 | 3 |
| GO:0017025 | TBP-class protein binding | 3/416 | 0.014251318 | 0.025939899 | ESR1/AHR/RUVBL2 | 3 |
| GO:0051537 | 2 iron, 2 sulfur cluster binding | 3/416 | 0.014251318 | 0.025939899 | CISD1/FXN/FECH | 3 |
| GO:0070412 | R-SMAD binding | 3/416 | 0.016108583 | 0.028958466 | JUN/FOS/PARP1 | 3 |
| GO:0001965 | G-protein alpha-subunit binding | 3/416 | 0.018095197 | 0.032133105 | OPRM1/DRD1/F2R | 3 |
| GO:0051119 | sugar transmembrane transporter activity | 3/416 | 0.018095197 | 0.032133105 | SLC2A4/SLC5A2/SLC5A1 | 3 |
| GO:0004190 | aspartic-type endopeptidase activity | 3/416 | 0.020211766 | 0.03560222 | CASP3/CTSD/BACE1 | 3 |
| GO:0005328 | neurotransmitter:sodium symporter activity | 3/416 | 0.022458619 | 0.03730369 | SLC6A2/SLC6A3/SLC6A4 | 3 |
| GO:0030371 | translation repressor activity | 3/416 | 0.022458619 | 0.03730369 | TYMS/DHFR/RARA | 3 |
| GO:0070001 | aspartic-type peptidase activity | 3/416 | 0.022458619 | 0.03730369 | CASP3/CTSD/BACE1 | 3 |
| GO:0004407 | histone deacetylase activity | 3/416 | 0.027343192 | 0.043914183 | HDAC6/HDAC8/HDAC1 | 3 |
| GO:0090079 | translation regulator activity, nucleic acid binding | 3/416 | 0.027343192 | 0.043914183 | TYMS/DHFR/RARA | 3 |
| GO:0004693 | cyclin-dependent protein serine/threonine kinase activity | 3/416 | 0.029980322 | 0.047119374 | CDK2/CDK1/CDK6 | 3 |
| GO:0033558 | protein deacetylase activity | 3/416 | 0.029980322 | 0.047119374 | HDAC6/HDAC8/HDAC1 | 3 |
| GO:0043236 | laminin binding | 3/416 | 0.029980322 | 0.047119374 | ACHE/CTSS/LGALS3 | 3 |
| GO:0008519 | ammonium transmembrane transporter activity | 3/416 | 0.032746583 | 0.049670439 | SLC6A2/SLC6A3/SLC6A4 | 3 |
| GO:0004955 | prostaglandin receptor activity | 2/416 | 0.021894568 | 0.036928453 | PPARG/PTGER3 | 2 |
| GO:0016595 | glutamate binding | 2/416 | 0.021894568 | 0.036928453 | GRIN1/GRIN2B | 2 |
| GO:0035620 | ceramide transporter activity | 2/416 | 0.021894568 | 0.036928453 | ABCB1/MTTP | 2 |
| GO:0043176 | amine binding | 2/416 | 0.021894568 | 0.036928453 | HTR3A/SLC6A4 | 2 |
| GO:0043995 | histone acetyltransferase activity (H4-K5 specific) | 2/416 | 0.021894568 | 0.036928453 | OGT/KANSL3 | 2 |
| GO:0043996 | histone acetyltransferase activity (H4-K8 specific) | 2/416 | 0.021894568 | 0.036928453 | OGT/KANSL3 | 2 |
| GO:0046972 | histone acetyltransferase activity (H4-K16 specific) | 2/416 | 0.021894568 | 0.036928453 | OGT/KANSL3 | 2 |
| GO:0051378 | serotonin binding | 2/416 | 0.021894568 | 0.036928453 | HTR3A/SLC6A4 | 2 |
| GO:0140078 | class I DNA-(apurinic or apyrimidinic site) endonuclease activity | 2/416 | 0.021894568 | 0.036928453 | NEIL1/NEIL2 | 2 |
| GO:0004303 | estradiol 17-beta-dehydrogenase activity | 2/416 | 0.026349184 | 0.042631233 | HSD17B1/HSD17B11 | 2 |
| GO:0004954 | prostanoid receptor activity | 2/416 | 0.026349184 | 0.042631233 | PPARG/PTGER3 | 2 |
| GO:0043560 | insulin receptor substrate binding | 2/416 | 0.026349184 | 0.042631233 | INSR/JAK2 | 2 |
| GO:0048407 | platelet-derived growth factor binding | 2/416 | 0.026349184 | 0.042631233 | COL1A1/COL3A1 | 2 |
| GO:0052650 | NADP-retinol dehydrogenase activity | 2/416 | 0.026349184 | 0.042631233 | AKR1B1/AKR1C3 | 2 |
| GO:0004861 | cyclin-dependent protein serine/threonine kinase inhibitor activity | 2/416 | 0.031134558 | 0.047722407 | CASP3/CDKN1A | 2 |
| GO:0008239 | dipeptidyl-peptidase activity | 2/416 | 0.031134558 | 0.047722407 | DPP4/DPEP1 | 2 |
| GO:0016004 | phospholipase activator activity | 2/416 | 0.031134558 | 0.047722407 | CASP3/ARF1 | 2 |
| GO:0031994 | insulin-like growth factor I binding | 2/416 | 0.031134558 | 0.047722407 | INSR/IGFBP3 | 2 |
| GO:0046624 | sphingolipid transporter activity | 2/416 | 0.031134558 | 0.047722407 | ABCB1/MTTP | 2 |
