# Supplementary material for: Mechanism of drug-pairs Astragalus Mongholicus–Largehead Atractylodes on treating knee osteoarthritis investigated by GEO gene chip with network pharmacology and molecular docking
Source: Medicine (Baltimore). 2024 Jul 5;103(27):e38699. doi: 10.1097/MD.0000000000038699 (PMC11224889; doi:10.1097/MD.0000000000038699)
Supplement: Supplementary file 11 [file medi-103-e38699-s011.doc]

# Appendix 11

**Cellular composition(CC) of GO enrichment analysis**

**Table S11. Cellular composition(CC) of GO enrichment analysis.**

| ID | Description | GeneRatio | pvalue | qvalue | geneID | Count |
| --- | --- | --- | --- | --- | --- | --- |
| GO:0045211 | postsynaptic membrane | 46/416 | 6.15E-25 | 1.65E-22 | CHRM3/CHRM1/ADRA1A/CHRM2/OPRM1/GABRA1/GRIA2/HTR3A/ADRA2C/DRD1/CHRM5/CHRM4/OPRD1/SLC6A3/SLC6A4/F2R/GLRA1/GLRA2/COMT/GABRA2/GABRA3/GABRA4/GABRA5/GABRA6/GABRG1/GABRG2/GABRG3/ARF1/GABRB1/GABRB2/GABRB3/GABRD/GABRE/GABRP/GABRQ/GRIN1/GRIN2A/GRIN2B/GRIN2C/GRIN2D/GRIN3A/GRIN3B/SIGMAR1/OPRK1/CACNA1C/TRPV1 | 46 |
| GO:1902711 | GABA-A receptor complex | 16/416 | 1.06E-24 | 1.65E-22 | GABRA1/GABRA2/GABRA3/GABRA4/GABRA5/GABRA6/GABRG1/GABRG2/GABRG3/GABRB1/GABRB2/GABRB3/GABRD/GABRE/GABRP/GABRQ | 16 |
| GO:1902710 | GABA receptor complex | 16/416 | 5.20E-24 | 5.40E-22 | GABRA1/GABRA2/GABRA3/GABRA4/GABRA5/GABRA6/GABRG1/GABRG2/GABRG3/GABRB1/GABRB2/GABRB3/GABRD/GABRE/GABRP/GABRQ | 16 |
| GO:0097060 | synaptic membrane | 50/416 | 6.87E-23 | 5.35E-21 | CHRM3/CHRM1/ADRA1A/CHRM2/OPRM1/GABRA1/GRIA2/SLC6A2/HTR3A/ADRA2C/DRD1/CHRM5/CHRM4/OPRD1/SLC6A3/SLC6A4/F2R/GLRA1/GLRA2/GRM4/COMT/GABRA2/GABRA3/GABRA4/GABRA5/GABRA6/GABRG1/GABRG2/GABRG3/ARF1/GABRB1/GABRB2/GABRB3/GABRD/GABRE/GABRP/GABRQ/GRIN1/GRIN2A/GRIN2B/GRIN2C/GRIN2D/GRIN3A/GRIN3B/SIGMAR1/OPRK1/CACNA1C/CACNA1D/TRPV1/GPER1 | 50 |
| GO:0034702 | ion channel complex | 41/416 | 1.42E-21 | 8.86E-20 | SCN5A/GABRA1/GRIA2/HTR3A/KCNH2/CLDN4/ATP2A1/GLRA1/GLRA2/GABRA2/GABRA3/GABRA4/GABRA5/GABRA6/GABRG1/GABRG2/GABRG3/GABRB1/GABRB2/GABRB3/GABRD/GABRE/GABRP/GABRQ/GRIN1/GRIN2A/GRIN2B/GRIN2C/GRIN2D/GRIN3A/GRIN3B/CACNA1C/CACNA1D/CACNA1F/CACNA1S/CACNB1/CACNB2/CACNB3/CACNB4/HSPA2/PTK2B | 41 |
| GO:1902495 | transmembrane transporter complex | 41/416 | 2.32E-20 | 1.21E-18 | SCN5A/GABRA1/GRIA2/HTR3A/KCNH2/CLDN4/ATP2A1/GLRA1/GLRA2/GABRA2/GABRA3/GABRA4/GABRA5/GABRA6/GABRG1/GABRG2/GABRG3/GABRB1/GABRB2/GABRB3/GABRD/GABRE/GABRP/GABRQ/GRIN1/GRIN2A/GRIN2B/GRIN2C/GRIN2D/GRIN3A/GRIN3B/CACNA1C/CACNA1D/CACNA1F/CACNA1S/CACNB1/CACNB2/CACNB3/CACNB4/HSPA2/PTK2B | 41 |
| GO:1990351 | transporter complex | 41/416 | 5.80E-20 | 2.58E-18 | SCN5A/GABRA1/GRIA2/HTR3A/KCNH2/CLDN4/ATP2A1/GLRA1/GLRA2/GABRA2/GABRA3/GABRA4/GABRA5/GABRA6/GABRG1/GABRG2/GABRG3/GABRB1/GABRB2/GABRB3/GABRD/GABRE/GABRP/GABRQ/GRIN1/GRIN2A/GRIN2B/GRIN2C/GRIN2D/GRIN3A/GRIN3B/CACNA1C/CACNA1D/CACNA1F/CACNA1S/CACNB1/CACNB2/CACNB3/CACNB4/HSPA2/PTK2B | 41 |
| GO:0034707 | chloride channel complex | 19/416 | 1.61E-19 | 6.26E-18 | GABRA1/CLDN4/GLRA1/GLRA2/GABRA2/GABRA3/GABRA4/GABRA5/GABRA6/GABRG1/GABRG2/GABRG3/GABRB1/GABRB2/GABRB3/GABRD/GABRE/GABRP/GABRQ | 19 |
| GO:0045121 | membrane raft | 36/416 | 1.47E-16 | 5.06E-15 | PTGS2/SCN5A/ADRA1A/OPRM1/DPP4/ADRA1B/SLC6A2/OLR1/OPRD1/SLC6A3/SLC6A4/KDR/IKBKB/CASP3/HMOX1/ICAM1/SELE/HAS2/SLC2A4/INSR/EGFR/MAPK1/CASP8/CAV1/GJA1/NOS3/CTSD/JAK2/F2R/HDAC6/HPSE/BACE1/TRPM8/TGFBR2/HCK/PTK2B | 36 |
| GO:0098857 | membrane microdomain | 36/416 | 1.62E-16 | 5.06E-15 | PTGS2/SCN5A/ADRA1A/OPRM1/DPP4/ADRA1B/SLC6A2/OLR1/OPRD1/SLC6A3/SLC6A4/KDR/IKBKB/CASP3/HMOX1/ICAM1/SELE/HAS2/SLC2A4/INSR/EGFR/MAPK1/CASP8/CAV1/GJA1/NOS3/CTSD/JAK2/F2R/HDAC6/HPSE/BACE1/TRPM8/TGFBR2/HCK/PTK2B | 36 |
| GO:0098589 | membrane region | 36/416 | 5.34E-16 | 1.51E-14 | PTGS2/SCN5A/ADRA1A/OPRM1/DPP4/ADRA1B/SLC6A2/OLR1/OPRD1/SLC6A3/SLC6A4/KDR/IKBKB/CASP3/HMOX1/ICAM1/SELE/HAS2/SLC2A4/INSR/EGFR/MAPK1/CASP8/CAV1/GJA1/NOS3/CTSD/JAK2/F2R/HDAC6/HPSE/BACE1/TRPM8/TGFBR2/HCK/PTK2B | 36 |
| GO:0032590 | dendrite membrane | 15/416 | 1.41E-15 | 3.67E-14 | OPRM1/GABRA1/OPRD1/INSR/GABRA2/GABRA3/GABRA4/GABRA5/GABRA6/GABRG1/GABRG2/GABRG3/GABRE/TRPV1/GPER1 | 15 |
| GO:0099055 | integral component of postsynaptic membrane | 21/416 | 4.78E-14 | 1.14E-12 | CHRM3/CHRM1/ADRA1A/CHRM2/OPRM1/GABRA1/HTR3A/ADRA2C/DRD1/OPRD1/SLC6A3/SLC6A4/GLRA1/GABRA3/GABRA4/GABRA5/GABRB2/GABRD/GRIN1/GRIN2A/OPRK1 | 21 |
| GO:0098936 | intrinsic component of postsynaptic membrane | 21/416 | 1.13E-13 | 2.52E-12 | CHRM3/CHRM1/ADRA1A/CHRM2/OPRM1/GABRA1/HTR3A/ADRA2C/DRD1/OPRD1/SLC6A3/SLC6A4/GLRA1/GABRA3/GABRA4/GABRA5/GABRB2/GABRD/GRIN1/GRIN2A/OPRK1 | 21 |
| GO:0099699 | integral component of synaptic membrane | 23/416 | 1.32E-13 | 2.74E-12 | CHRM3/CHRM1/ADRA1A/CHRM2/OPRM1/GABRA1/HTR3A/ADRA2C/DRD1/OPRD1/SLC6A3/SLC6A4/GLRA1/GABRA3/GABRA4/GABRA5/GABRG3/GABRB2/GABRD/GRIN1/GRIN2A/OPRK1/CACNA1D | 23 |
| GO:0032589 | neuron projection membrane | 15/416 | 5.59E-13 | 1.09E-11 | OPRM1/GABRA1/OPRD1/INSR/GABRA2/GABRA3/GABRA4/GABRA5/GABRA6/GABRG1/GABRG2/GABRG3/GABRE/TRPV1/GPER1 | 15 |
| GO:0099240 | intrinsic component of synaptic membrane | 23/416 | 6.87E-13 | 1.26E-11 | CHRM3/CHRM1/ADRA1A/CHRM2/OPRM1/GABRA1/HTR3A/ADRA2C/DRD1/OPRD1/SLC6A3/SLC6A4/GLRA1/GABRA3/GABRA4/GABRA5/GABRG3/GABRB2/GABRD/GRIN1/GRIN2A/OPRK1/CACNA1D | 23 |
| GO:0017146 | NMDA selective glutamate receptor complex | 8/416 | 5.73E-12 | 9.93E-11 | GRIN1/GRIN2A/GRIN2B/GRIN2C/GRIN2D/GRIN3A/GRIN3B/PTK2B | 8 |
| GO:0005901 | caveola | 16/416 | 9.09E-12 | 1.49E-10 | PTGS2/SCN5A/ADRA1A/ADRA1B/SLC6A3/HMOX1/SELE/INSR/MAPK1/CAV1/NOS3/JAK2/F2R/HDAC6/TGFBR2/HCK | 16 |
| GO:0090575 | RNA polymerase II transcription factor complex | 21/416 | 3.64E-11 | 5.68E-10 | RXRA/PPARG/PPARD/RXRB/JUN/NR1I2/NR1I3/FOS/RB1/TP53/HIF1A/PPARA/E2F1/E2F2/VDR/NR1H4/CEBPB/RARA/RARB/RARG/RXRG | 21 |
| GO:0044853 | plasma membrane raft | 17/416 | 1.30E-10 | 1.93E-09 | PTGS2/SCN5A/ADRA1A/ADRA1B/SLC6A3/HMOX1/SELE/HAS2/INSR/MAPK1/CAV1/NOS3/JAK2/F2R/HDAC6/TGFBR2/HCK | 17 |
| GO:0099056 | integral component of presynaptic membrane | 14/416 | 3.32E-10 | 4.70E-09 | CHRM3/CHRM1/ADRA1A/CHRM2/OPRM1/HTR3A/DRD1/OPRD1/SLC6A3/SLC6A4/GLRA1/GABRA5/OPRK1/CACNA1D | 14 |
| GO:0098889 | intrinsic component of presynaptic membrane | 14/416 | 1.66E-09 | 2.25E-08 | CHRM3/CHRM1/ADRA1A/CHRM2/OPRM1/HTR3A/DRD1/OPRD1/SLC6A3/SLC6A4/GLRA1/GABRA5/OPRK1/CACNA1D | 14 |
| GO:0034703 | cation channel complex | 22/416 | 1.74E-09 | 2.26E-08 | SCN5A/GRIA2/HTR3A/KCNH2/ATP2A1/GRIN1/GRIN2A/GRIN2B/GRIN2C/GRIN2D/GRIN3A/GRIN3B/CACNA1C/CACNA1D/CACNA1F/CACNA1S/CACNB1/CACNB2/CACNB3/CACNB4/HSPA2/PTK2B | 22 |
| GO:0044798 | nuclear transcription factor complex | 21/416 | 1.85E-09 | 2.31E-08 | RXRA/PPARG/PPARD/RXRB/JUN/NR1I2/NR1I3/FOS/RB1/TP53/HIF1A/PPARA/E2F1/E2F2/VDR/NR1H4/CEBPB/RARA/RARB/RARG/RXRG | 21 |
| GO:0099634 | postsynaptic specialization membrane | 15/416 | 3.24E-09 | 3.88E-08 | CHRM3/CHRM1/GABRA1/ADRA2C/OPRD1/GLRA1/GABRA3/GABRA4/GABRA5/GABRB2/GRIN1/GRIN2A/GRIN2B/GRIN2C/GRIN2D | 15 |
| GO:0005667 | transcription factor complex | 28/416 | 4.57E-09 | 5.28E-08 | RXRA/CDK2/PPARG/PPARD/RELA/RXRB/JUN/NR1I2/AHR/NR1I3/FOS/RB1/TP53/HIF1A/PARP1/PPARA/RUNX2/E2F1/E2F2/HDAC1/VDR/NR1H4/CEBPB/RARA/RARB/RARG/RXRG/HOXA10 | 28 |
| GO:0042734 | presynaptic membrane | 18/416 | 9.26E-09 | 1.03E-07 | CHRM3/CHRM1/ADRA1A/CHRM2/OPRM1/SLC6A2/HTR3A/DRD1/OPRD1/SLC6A3/SLC6A4/GLRA1/GRM4/GABRA5/GRIN2A/OPRK1/CACNA1D/GPER1 | 18 |
| GO:0031983 | vesicle lumen | 26/416 | 1.65E-08 | 1.78E-07 | MAPK14/ALOX5/GSTP1/PSMD3/SLPI/EGFR/VEGFA/MAPK1/EGF/SERPINE1/MPO/CTSD/IGF2/MMP8/HSPA8/ADA/HPSE/HSP90AA1/BACE1/CDA/CTSC/HBA1/TF/NFKB1/ORM1/CSNK2B | 26 |
| GO:0031253 | cell projection membrane | 26/416 | 2.37E-08 | 2.46E-07 | OPRM1/GABRA1/DPP4/DRD1/OPRD1/DPEP1/INSR/NPC1L1/SLC5A1/CA9/FOLR1/SLC46A1/SLC22A12/CA4/GABRA2/GABRA3/GABRA4/GABRA5/GABRA6/GABRG1/GABRG2/GABRG3/GABRE/TRPV1/GPER1/RHO | 26 |
| GO:0043025 | neuronal cell body | 32/416 | 2.48E-08 | 2.49E-07 | CHRM2/OPRM1/HSP90AA2P/SLC6A2/NCF1/HTR3A/ADRA2C/SLC6A3/CASP3/INSR/MAPK1/IL6ST/ELK1/TOP1/SOD1/NQO1/ADA/HDAC6/HDAC1/CYP17A1/GLRA1/HSP90AA1/BACE1/SRD5A1/GABRA5/GRIN3A/GRIN3B/OPRK1/CACNA1C/CACNA1F/TRPV1/PTK2B | 32 |
| GO:0098982 | GABA-ergic synapse | 12/416 | 2.77E-08 | 2.69E-07 | ADRA1A/GABRA1/DRD1/GABRA3/GABRA4/GABRA5/GABRG2/GABRG3/GABRB1/GABRB2/GABRB3/GABRD | 12 |
| GO:0099060 | integral component of postsynaptic specialization membrane | 12/416 | 4.48E-08 | 4.23E-07 | CHRM3/CHRM1/GABRA1/ADRA2C/OPRD1/GLRA1/GABRA3/GABRA4/GABRA5/GABRB2/GRIN1/GRIN2A | 12 |
| GO:0060205 | cytoplasmic vesicle lumen | 25/416 | 6.37E-08 | 5.84E-07 | MAPK14/ALOX5/GSTP1/PSMD3/SLPI/VEGFA/MAPK1/EGF/SERPINE1/MPO/CTSD/IGF2/MMP8/HSPA8/ADA/HPSE/HSP90AA1/BACE1/CDA/CTSC/HBA1/TF/NFKB1/ORM1/CSNK2B | 25 |
| GO:0098948 | intrinsic component of postsynaptic specialization membrane | 12/416 | 7.08E-08 | 6.30E-07 | CHRM3/CHRM1/GABRA1/ADRA2C/OPRD1/GLRA1/GABRA3/GABRA4/GABRA5/GABRB2/GRIN1/GRIN2A | 12 |
| GO:0098878 | neurotransmitter receptor complex | 10/416 | 1.36E-07 | 1.18E-06 | GRIA2/HTR3A/GRIN1/GRIN2A/GRIN2B/GRIN2C/GRIN2D/GRIN3A/GRIN3B/PTK2B | 10 |
| GO:0098793 | presynapse | 30/416 | 2.14E-07 | 1.80E-06 | CHRM3/CHRM1/ADRA1A/CHRM2/OPRM1/SLC6A2/HTR3A/ADRA2C/DRD1/OPRD1/SLC6A3/SLC6A4/SLC2A4/BCL2L1/ELK1/PRKCB/SLC29A1/HSPA8/GLRA1/BACE1/GRM4/GABRA2/GABRA5/GRIN1/GRIN2A/OPRK1/CACNA1D/CACNB2/ACTB/GPER1 | 30 |
| GO:0005891 | voltage-gated calcium channel complex | 9/416 | 2.27E-07 | 1.86E-06 | CACNA1C/CACNA1D/CACNA1F/CACNA1S/CACNB1/CACNB2/CACNB3/CACNB4/HSPA2 | 9 |
| GO:1904813 | ficolin-1-rich granule lumen | 14/416 | 3.68E-07 | 2.94E-06 | MAPK14/TNFAIP6/ALOX5/GSTP1/PSMD3/MMP9/MAPK1/CTSD/HSPA8/HSP90AA1/CTSS/CDA/FTH1/CSNK2B | 14 |
| GO:0099572 | postsynaptic specialization | 24/416 | 4.24E-07 | 3.30E-06 | CHRM3/CHRM1/GABRA1/GRIA2/ADRA2C/OPRD1/MAPK1/GLRA1/GABRA3/GABRA4/GABRA5/ARF1/ITPR1/GABRB2/GRIN1/GRIN2A/GRIN2B/GRIN2C/GRIN2D/GRIN3A/SIGMAR1/CACNA1C/GPER1/PTK2B | 24 |
| GO:0031256 | leading edge membrane | 16/416 | 6.71E-07 | 5.10E-06 | OPRM1/GABRA1/DPP4/OPRD1/INSR/GABRA2/GABRA3/GABRA4/GABRA5/GABRA6/GABRG1/GABRG2/GABRG3/GABRE/TRPV1/GPER1 | 16 |
| GO:0008328 | ionotropic glutamate receptor complex | 9/416 | 1.06E-06 | 7.83E-06 | GRIA2/GRIN1/GRIN2A/GRIN2B/GRIN2C/GRIN2D/GRIN3A/GRIN3B/PTK2B | 9 |
| GO:0034704 | calcium channel complex | 10/416 | 1.15E-06 | 8.36E-06 | ATP2A1/CACNA1C/CACNA1D/CACNA1F/CACNA1S/CACNB1/CACNB2/CACNB3/CACNB4/HSPA2 | 10 |
| GO:0043679 | axon terminus | 13/416 | 1.40E-06 | 9.90E-06 | CHRM3/CHRM1/CHRM2/ADRA2C/OPRD1/ELK1/PRKCB/HSPA8/GLRA1/GRIN1/OPRK1/ACTB/GPER1 | 13 |
| GO:0034774 | secretory granule lumen | 22/416 | 1.43E-06 | 9.90E-06 | MAPK14/ALOX5/GSTP1/PSMD3/SLPI/VEGFA/MAPK1/EGF/SERPINE1/MPO/CTSD/IGF2/MMP8/HSPA8/HPSE/HSP90AA1/CDA/CTSC/TF/NFKB1/ORM1/CSNK2B | 22 |
| GO:0000307 | cyclin-dependent protein kinase holoenzyme complex | 8/416 | 2.31E-06 | 1.57E-05 | CDK2/CCNA2/CDK1/CCND1/CDKN1A/RB1/CCNB1/CDK6 | 8 |
| GO:0045277 | respiratory chain complex IV | 6/416 | 2.57E-06 | 1.70E-05 | COX4I1/COX5A/COX6A2/COX6B1/COX7B/COX8A | 6 |
| GO:1990454 | L-type voltage-gated calcium channel complex | 5/416 | 2.86E-06 | 1.86E-05 | CACNA1C/CACNA1D/CACNA1S/CACNB2/CACNB3 | 5 |
| GO:0000790 | nuclear chromatin | 23/416 | 5.86E-06 | 3.73E-05 | AR/RXRA/NCOA1/ESR1/PPARD/RELA/JUN/STAT1/RB1/TP53/HIF1A/MYC/RUNX2/E2F1/IRF1/HDAC1/NR1H4/ACTB/CEBPB/CSNK2A1/RUVBL2/RARA/RARG | 23 |
| GO:0042383 | sarcolemma | 13/416 | 6.30E-06 | 3.92E-05 | SCN5A/ADRA1A/OPRM1/VCAM1/SLC2A4/PPP3CA/CAV1/CACNA1C/CACNA1D/CACNA1S/CACNB1/CACNB2/CACNB3 | 13 |
| GO:0044306 | neuron projection terminus | 13/416 | 7.39E-06 | 4.52E-05 | CHRM3/CHRM1/CHRM2/ADRA2C/OPRD1/ELK1/PRKCB/HSPA8/GLRA1/GRIN1/OPRK1/ACTB/GPER1 | 13 |
| GO:0045177 | apical part of cell | 23/416 | 7.92E-06 | 4.74E-05 | ADRB2/DPP4/DPEP1/VCAM1/EGFR/ERBB2/GJA1/DUOX2/PLAT/CLDN4/ERBB3/NPC1L1/ABCB1/SLC5A1/SLC29A1/CA2/FOLR1/SLC46A1/NOX4/SLC22A12/CA4/TF/CACNB3 | 23 |
| GO:0101002 | ficolin-1-rich granule | 15/416 | 9.45E-06 | 5.56E-05 | MAPK14/TNFAIP6/ALOX5/GSTP1/PSMD3/MMP9/MAPK1/CTSD/HSPA8/HSP90AA1/CTSS/LGALS3/CDA/FTH1/CSNK2B | 15 |
| GO:0030315 | T-tubule | 8/416 | 1.23E-05 | 7.07E-05 | SCN5A/ADRA1A/SLC2A4/CACNA1C/CACNA1D/CACNA1S/CACNB2/CACNB3 | 8 |
| GO:0072562 | blood microparticle | 13/416 | 1.47E-05 | 8.33E-05 | PRSS1/IGHG1/PON1/HSPA8/GC/CP/HBA1/TF/TFRC/ORM1/IGHG2/ACTB/HSPA2 | 13 |
| GO:0016324 | apical plasma membrane | 20/416 | 1.53E-05 | 8.52E-05 | ADRB2/DPP4/DPEP1/EGFR/ERBB2/GJA1/DUOX2/CLDN4/ERBB3/NPC1L1/ABCB1/SLC5A1/SLC29A1/FOLR1/SLC46A1/NOX4/SLC22A12/CA4/TF/CACNB3 | 20 |
| GO:1902554 | serine/threonine protein kinase complex | 10/416 | 1.63E-05 | 8.93E-05 | CDK2/CCNA2/IKBKB/CDK1/CCND1/CDKN1A/RB1/CCNB1/CHUK/CDK6 | 10 |
| GO:0098978 | glutamatergic synapse | 21/416 | 1.83E-05 | 9.83E-05 | CHRM3/CHRM1/ADRA1A/CHRM2/MAPK14/GSK3B/RELA/HTR3A/ADRA2C/DRD1/PPP3CA/PLAT/JAK2/ARF1/GABRD/GRIN1/GRIN2A/GRIN2C/GRIN3A/ACTB/PTK2B | 21 |
| GO:1904724 | tertiary granule lumen | 8/416 | 1.88E-05 | 9.91E-05 | TNFAIP6/MMP9/CTSD/MMP8/CTSS/CDA/FTH1/ORM1 | 8 |
| GO:1902911 | protein kinase complex | 11/416 | 1.94E-05 | 0.000100924 | CDK2/CCNA2/IKBKB/CDK1/INSR/CCND1/CDKN1A/RB1/CCNB1/CHUK/CDK6 | 11 |
| GO:0098839 | postsynaptic density membrane | 9/416 | 2.50E-05 | 0.000127647 | CHRM3/CHRM1/ADRA2C/OPRD1/GRIN1/GRIN2A/GRIN2B/GRIN2C/GRIN2D | 9 |
| GO:0009897 | external side of plasma membrane | 22/416 | 3.51E-05 | 0.000176164 | GRIA2/IGHG1/ICAM1/VCAM1/SLC2A4/INSR/IL6ST/F3/CXCL10/CD40LG/ADA/CCR1/FOLR1/FOLR2/GLRA1/TRPM8/CA4/TF/TFRC/TGFBR2/IGHG2/TRPV1 | 22 |
| GO:0031252 | cell leading edge | 22/416 | 5.10E-05 | 0.000252254 | OPRM1/GABRA1/DPP4/OPRD1/INSR/DUOX2/RASA1/HDAC6/GABRA2/GABRA3/GABRA4/GABRA5/GABRA6/GABRG1/GABRG2/GABRG3/ARF1/GABRE/TRPV1/CDK6/GPER1/PTK2B | 22 |
| GO:0098984 | neuron to neuron synapse | 20/416 | 5.96E-05 | 0.000288395 | CHRM3/CHRM1/CHRM2/GRIA2/ADRA2C/OPRD1/MAPK1/BACE1/ARF1/ITPR1/GRIN1/GRIN2A/GRIN2B/GRIN2C/GRIN2D/GRIN3A/SIGMAR1/CACNA1C/GPER1/PTK2B | 20 |
| GO:0098802 | plasma membrane receptor complex | 18/416 | 6.02E-05 | 0.000288395 | GRIA2/HTR3A/IKBKB/INSR/IL10RB/IL6ST/CHUK/TF/TFRC/GRIN1/GRIN2A/GRIN2B/GRIN2C/GRIN2D/GRIN3A/GRIN3B/IL6/PTK2B | 18 |
| GO:0009925 | basal plasma membrane | 6/416 | 6.94E-05 | 0.00032253 | MET/EGFR/ERBB2/CLDN4/ERBB3/TF | 6 |
| GO:0070069 | cytochrome complex | 6/416 | 6.94E-05 | 0.00032253 | COX4I1/COX5A/COX6A2/COX6B1/COX7B/COX8A | 6 |
| GO:0043209 | myelin sheath | 7/416 | 7.08E-05 | 0.00032421 | HSP90AA2P/AKR1B1/BCL2/ERBB2/CA2/HSP90AA1/SRD5A1 | 7 |
| GO:0032279 | asymmetric synapse | 19/416 | 7.58E-05 | 0.000342244 | CHRM3/CHRM1/CHRM2/GRIA2/ADRA2C/OPRD1/MAPK1/ARF1/ITPR1/GRIN1/GRIN2A/GRIN2B/GRIN2C/GRIN2D/GRIN3A/SIGMAR1/CACNA1C/GPER1/PTK2B | 19 |
| GO:0031968 | organelle outer membrane | 14/416 | 9.89E-05 | 0.000440086 | PGR/MAOB/BCL2/BAX/BCL2L1/CASP8/RAF1/GJA1/HK2/MCL1/CISD1/CYB5A/SIGMAR1/CYP27B1 | 14 |
| GO:0005741 | mitochondrial outer membrane | 13/416 | 0.000108295 | 0.000475245 | PGR/MAOB/BCL2/BAX/BCL2L1/CASP8/RAF1/GJA1/HK2/MCL1/CISD1/CYB5A/CYP27B1 | 13 |
| GO:0019867 | outer membrane | 14/416 | 0.000109848 | 0.000475365 | PGR/MAOB/BCL2/BAX/BCL2L1/CASP8/RAF1/GJA1/HK2/MCL1/CISD1/CYB5A/SIGMAR1/CYP27B1 | 14 |
| GO:0043202 | lysosomal lumen | 9/416 | 0.000180065 | 0.000768556 | CTSD/HSPA8/GBA/GC/HPSE/HSP90AA1/CTSK/CTSS/CTSF | 9 |
| GO:0014069 | postsynaptic density | 18/416 | 0.000195782 | 0.000824345 | CHRM3/CHRM1/GRIA2/ADRA2C/OPRD1/MAPK1/ARF1/ITPR1/GRIN1/GRIN2A/GRIN2B/GRIN2C/GRIN2D/GRIN3A/SIGMAR1/CACNA1C/GPER1/PTK2B | 18 |
| GO:0005775 | vacuolar lumen | 12/416 | 0.000301924 | 0.001254309 | MAPK1/MPO/CTSD/HSPA8/GBA/GC/HPSE/HSP90AA1/CTSK/CTSS/CTSC/CTSF | 12 |
| GO:0005788 | endoplasmic reticulum lumen | 17/416 | 0.000327654 | 0.00134329 | PTGS2/F7/HSPA5/COL1A1/COL3A1/SPP1/IGFBP3/BACE1/NOTUM/CTSC/CES2/MTTP/CP/TF/IL6/DBI/CES1 | 17 |
| GO:0150034 | distal axon | 16/416 | 0.00039012 | 0.001578612 | CHRM3/CHRM1/CHRM2/ADRA2C/OPRD1/ELK1/PRKCB/HSPA8/GLRA1/HSP90AA1/GRIN1/SIGMAR1/OPRK1/ACTB/GPER1/PTK2B | 16 |
| GO:0062023 | collagen-containing extracellular matrix | 20/416 | 0.000426938 | 0.001705445 | ACHE/PRSS1/F7/ICAM1/SLPI/MMP2/MMP9/F3/SERPINE1/COL1A1/COL3A1/CTSD/PCOLCE/MMP8/CTSS/LGALS4/LGALS3/CTSC/CTSF/ORM1 | 20 |
| GO:0033267 | axon part | 19/416 | 0.000525196 | 0.002071393 | CHRM3/CHRM1/CHRM2/ADRA2C/OPRD1/ELK1/SOD1/HIF1A/PRKCB/HSPB1/HSPA8/GLRA1/HSP90AA1/GRIN1/SIGMAR1/OPRK1/ACTB/GPER1/PTK2B | 19 |
| GO:0099061 | integral component of postsynaptic density membrane | 6/416 | 0.000616776 | 0.002402179 | CHRM3/CHRM1/ADRA2C/OPRD1/GRIN1/GRIN2A | 6 |
| GO:0045178 | basal part of cell | 6/416 | 0.00068675 | 0.002641688 | MET/EGFR/ERBB2/CLDN4/ERBB3/TF | 6 |
| GO:0016323 | basolateral plasma membrane | 13/416 | 0.000739388 | 0.002783425 | CHRM3/MET/EGFR/ERBB2/CLDN4/ERBB3/SLC29A1/CA2/CA9/FOLR1/MTTP/TF/TFRC | 13 |
| GO:0070820 | tertiary granule | 11/416 | 0.000741463 | 0.002783425 | OLR1/TNFAIP6/PLAU/MMP9/CTSD/MMP8/CTSS/LGALS3/CDA/FTH1/ORM1 | 11 |
| GO:0031526 | brush border membrane | 6/416 | 0.000845003 | 0.003097472 | NPC1L1/SLC5A1/FOLR1/SLC46A1/SLC22A12/CA4 | 6 |
| GO:0099146 | intrinsic component of postsynaptic density membrane | 6/416 | 0.000845003 | 0.003097472 | CHRM3/CHRM1/ADRA2C/OPRD1/GRIN1/GRIN2A | 6 |
| GO:0043197 | dendritic spine | 11/416 | 0.000949603 | 0.003440423 | OPRM1/GRIA2/DRD1/OPRD1/PPP3CA/COMT/GRIN1/GRIN2A/TRPV1/GPER1/PTK2B | 11 |
| GO:0044309 | neuron spine | 11/416 | 0.001045375 | 0.003732105 | OPRM1/GRIA2/DRD1/OPRD1/PPP3CA/COMT/GRIN1/GRIN2A/TRPV1/GPER1/PTK2B | 11 |
| GO:0005925 | focal adhesion | 19/416 | 0.001054067 | 0.003732105 | OPRM1/DPP4/SLC6A4/ICAM1/EGFR/PLAU/MAPK1/HSPA5/CAV1/GJA1/HSPB1/JAK1/JAK2/HSPA8/NOX4/ARF1/ACTB/HCK/PTK2B | 19 |
| GO:0005924 | cell-substrate adherens junction | 19/416 | 0.001148749 | 0.00402164 | OPRM1/DPP4/SLC6A4/ICAM1/EGFR/PLAU/MAPK1/HSPA5/CAV1/GJA1/HSPB1/JAK1/JAK2/HSPA8/NOX4/ARF1/ACTB/HCK/PTK2B | 19 |
| GO:0030055 | cell-substrate junction | 19/416 | 0.001286224 | 0.004452892 | OPRM1/DPP4/SLC6A4/ICAM1/EGFR/PLAU/MAPK1/HSPA5/CAV1/GJA1/HSPB1/JAK1/JAK2/HSPA8/NOX4/ARF1/ACTB/HCK/PTK2B | 19 |
| GO:0031094 | platelet dense tubular network | 3/416 | 0.001356271 | 0.004643796 | ATP2A1/F2R/ITPR1 | 3 |
| GO:0031233 | intrinsic component of external side of plasma membrane | 4/416 | 0.001485023 | 0.005029369 | F3/FOLR1/FOLR2/CA4 | 4 |
| GO:0005751 | mitochondrial respiratory chain complex IV | 3/416 | 0.001780184 | 0.005964169 | COX4I1/COX5A/COX6A2 | 3 |
| GO:0035580 | specific granule lumen | 6/416 | 0.001930415 | 0.006398689 | SLPI/CTSD/MMP8/HPSE/NFKB1/ORM1 | 6 |
| GO:0043020 | NADPH oxidase complex | 3/416 | 0.003512663 | 0.011520757 | NCF1/DUOX2/NOX4 | 3 |
| GO:0061695 | transferase complex, transferring phosphorus-containing groups | 13/416 | 0.003557705 | 0.011546938 | CDK2/CCNA2/IKBKB/CDK1/INSR/CCND1/CDKN1A/RB1/TP53/CCNB1/CHUK/CDK6/PIK3CG | 13 |
| GO:0005771 | multivesicular body | 5/416 | 0.004321482 | 0.013881266 | SLC2A4/EGFR/GJA1/HDAC6/BACE1 | 5 |
| GO:0032839 | dendrite cytoplasm | 4/416 | 0.004401186 | 0.013993028 | OPRM1/MAPK1/SOD1/ADA | 4 |
| GO:0005903 | brush border | 7/416 | 0.004992382 | 0.015712334 | NPC1L1/SLC5A1/FOLR1/SLC46A1/SLC22A12/CA4/SOAT2 | 7 |
| GO:0070469 | respiratory chain | 7/416 | 0.005273363 | 0.016430689 | COX4I1/COX5A/COX6A2/COX6B1/COX7A1/COX7B/COX8A | 7 |
| GO:0005743 | mitochondrial inner membrane | 19/416 | 0.005846144 | 0.018035004 | ATP5F1B/HSD3B2/HSD3B1/CYP1A1/BCL2L1/TYMS/FPGS/LGALS3/COX4I1/COX5A/COX5B/COX6A2/COX6B1/COX6C/COX7A1/COX7B/COX7C/COX8A/FECH | 19 |
| GO:0034399 | nuclear periphery | 8/416 | 0.006678949 | 0.020402156 | ALOX5/EIF6/TP53/RUNX1T1/JAK2/CEBPB/CSNK2B/RUVBL2 | 8 |
| GO:0031362 | anchored component of external side of plasma membrane | 3/416 | 0.00702797 | 0.021259879 | FOLR1/FOLR2/CA4 | 3 |
| GO:0005819 | spindle | 15/416 | 0.007194718 | 0.021555027 | MAPK14/AKT1/CDK1/MAPK1/RB1/BIRC5/HSPB1/CCNB1/HSF1/RASSF1/NR3C1/NEIL2/POLB/HSPA2/ACOT13 | 15 |
| GO:0043204 | perikaryon | 8/416 | 0.007622048 | 0.022617807 | OPRM1/MAPK1/TOP1/HDAC6/GLRA1/OPRK1/CACNA1C/CACNA1F | 8 |
| GO:0098685 | Schaffer collateral - CA1 synapse | 6/416 | 0.00774287 | 0.022759579 | CHRM1/ADRB1/PPP3CA/PLAT/ITPR1/ACTB | 6 |
| GO:0071682 | endocytic vesicle lumen | 3/416 | 0.008140478 | 0.023607953 | MPO/HSP90AA1/HBA1 | 3 |
| GO:0000791 | euchromatin | 4/416 | 0.008183027 | 0.023607953 | JUN/HSF1/NR1H4/RUVBL2 | 4 |
| GO:0016363 | nuclear matrix | 7/416 | 0.008364248 | 0.023909392 | ALOX5/TP53/RUNX1T1/JAK2/CEBPB/CSNK2B/RUVBL2 | 7 |
| GO:0120111 | neuron projection cytoplasm | 6/416 | 0.008679962 | 0.024586303 | OPRM1/MAPK1/SOD1/HIF1A/HSPB1/ADA | 6 |
| GO:0098803 | respiratory chain complex | 6/416 | 0.009177763 | 0.025762143 | COX4I1/COX5A/COX6A2/COX6B1/COX7B/COX8A | 6 |
| GO:0031970 | organelle envelope lumen | 6/416 | 0.010793232 | 0.030026286 | HSD3B2/HSD3B1/ALOX5/SOD1/PTGES/COX6B1 | 6 |
| GO:0030665 | clathrin-coated vesicle membrane | 7/416 | 0.011057689 | 0.030489762 | CHRM2/ADRB2/EGFR/EGF/HSPA8/TF/TFRC | 7 |
| GO:0098562 | cytoplasmic side of membrane | 9/416 | 0.013498998 | 0.036483555 | IKBKB/PPP3CA/CHUK/PTPN1/CISD1/CACNB4/HCK/PTK2B/G6PD | 9 |
| GO:0031093 | platelet alpha granule lumen | 5/416 | 0.013526806 | 0.036483555 | VEGFA/EGF/SERPINE1/IGF2/ORM1 | 5 |
| GO:0044305 | calyx of Held | 3/416 | 0.013582729 | 0.036483555 | PRKCB/GLRA1/ACTB | 3 |
| GO:0098862 | cluster of actin-based cell projections | 8/416 | 0.014429084 | 0.03842563 | NPC1L1/SLC5A1/FOLR1/SLC46A1/SLC22A12/TRPA1/CA4/SOAT2 | 8 |
| GO:0016529 | sarcoplasmic reticulum | 5/416 | 0.017041423 | 0.044942082 | SLC2A4/GSTM2/HK2/ATP2A1/ITPR1 | 5 |
| GO:0005769 | early endosome | 14/416 | 0.017164535 | 0.044942082 | ADRB2/ADRB1/KDR/VCAM1/EGFR/MAPK1/CAV1/GJA1/PTPN1/F2R/BACE1/TF/TFRC/GPER1 | 14 |
| GO:0097470 | ribbon synapse | 2/416 | 0.017865414 | 0.046004025 | CACNA1D/CACNB2 | 2 |
| GO:0098981 | cholinergic synapse | 2/416 | 0.017865414 | 0.046004025 | CHRM1/CHRM2 | 2 |
| GO:0030136 | clathrin-coated vesicle | 9/416 | 0.018630792 | 0.047469991 | CHRM2/ADRB2/SLC2A4/EGFR/EGF/HSPA8/FOLR1/TF/TFRC | 9 |
| GO:0032809 | neuronal cell body membrane | 3/416 | 0.018739421 | 0.047469991 | SLC6A2/INSR/GABRA5 | 3 |
