# Supplementary material for: Mechanism of drug-pairs Astragalus Mongholicus–Largehead Atractylodes on treating knee osteoarthritis investigated by GEO gene chip with network pharmacology and molecular docking
Source: Medicine (Baltimore). 2024 Jul 5;103(27):e38699. doi: 10.1097/MD.0000000000038699 (PMC11224889; doi:10.1097/MD.0000000000038699)
Supplement: Supplementary file 12 [file medi-103-e38699-s012.doc]

# Appendix 12

**Biological process(BP) of GO enrichment analysis**

**Table S12. Biological process(BP) of GO enrichment analysis.**

| ID | Description | GeneRatio | pvalue | qvalue | geneID | Count |
| --- | --- | --- | --- | --- | --- | --- |
| GO:0031667 | response to nutrient levels | 63/412 | 7.44E-30 | 4.71E-27 | PTGS2/ADRB2/OPRM1/NCOA1/PPARG/PPARD/F7/RELA/ADRB1/SLC6A4/JUN/AKT1/BCL2/MAPK8/STAT1/HMOX1/CYP1A1/ICAM1/VCAM1/GSTP1/AKR1C3/EGFR/CCND1/CDKN1A/MAPK1/TP53/POR/SOD1/HSPA5/IL1B/COL1A1/MPO/NFE2L2/NQO1/PPARA/HSF1/CXCL10/SPP1/PON1/TYR/HSPA8/ADA/GBA/TYMS/FOLR1/FOLR2/HMGCR/NMUR2/OGT/SRD5A1/COMT/VDR/TGFBR2/GRIN1/HSD11B2/NR1H4/CACNB3/TRPV1/PIM1/RARA/COX4I1/G6PD/CYP27B1 | 63 |
| GO:0048545 | response to steroid hormone | 59/412 | 1.27E-32 | 1.61E-29 | PTGS2/PGR/AR/RXRA/NCOA2/NCOA1/ESR2/ESR1/PPARG/PPARD/MAOB/RELA/RXRB/HSD3B2/HSD3B1/BCL2/CASP3/ICAM1/NR1I2/GSTP1/NR1I3/AKR1C3/EGFR/CCND1/FOS/CDKN1A/CASP9/RB1/CAV1/COL1A1/PARP1/CLDN4/PPARA/SPP1/JAK2/NR3C1/CA2/GBA/TYMS/HDAC6/HDAC1/RORC/SRD5A1/VDR/TGFBR2/GABRB1/HSD11B2/IL6/NR3C2/RORA/NR1H4/ESRRA/ESRRB/GPER1/RARA/RARB/RARG/RXRG/ESRRG | 59 |
| GO:0046677 | response to antibiotic | 58/412 | 1.16E-35 | 2.93E-32 | OPRM1/MAOB/RELA/HTR3A/SLC6A3/MET/JUN/BCL2/CASP3/STAT1/CDK1/HMOX1/CYP1A1/ICAM1/VCAM1/CYP1B1/GSTP1/AHR/SLC2A4/CCND1/BCL2L1/CASP9/TP53/CASP8/SOD1/HSPA5/IL2/COL1A1/NFE2L2/NQO1/HSF1/CHUK/JAK1/JAK2/ADA/TYMS/HDAC6/HMGCR/GLRA1/GLRA2/HSP90AA1/KDM6B/SRD5A1/TRPA1/FXN/HBA1/POLB/GRIN1/GRIN2A/GRIN2B/GRIN3A/IL6/SIGMAR1/OPRK1/PTK2B/MB/RARA/G6PD | 58 |
| GO:0042391 | regulation of membrane potential | 55/412 | 3.55E-26 | 8.16E-24 | CHRM1/SCN5A/ADRA1A/ADRB2/OPRM1/GABRA1/GSK3B/ADRB1/HTR3A/DRD1/KCNH2/OPRD1/KDR/JUN/AKT1/BCL2/BAX/PPP3CA/BCL2L1/SOD1/CAV1/GJA1/PARP1/GBA/GLRA1/GLRA2/GABRA2/GABRA3/GABRA4/GABRA5/GABRA6/GABRG1/GABRG2/GABRG3/GABRB1/GABRB2/GABRB3/GABRD/GABRE/GABRP/GABRQ/GRIN1/GRIN2A/GRIN2B/GRIN2C/GRIN2D/GRIN3A/CACNA1C/CACNA1D/CACNB2/CACNB3/CACNB4/TRPV1/GPER1/PTK2B | 55 |
| GO:0006979 | response to oxidative stress | 55/412 | 2.43E-25 | 5.01E-23 | PTGS2/PTGS1/CDK2/CCNA2/RELA/NCF1/MET/DPEP1/JUN/AKT1/BCL2/CASP3/MAPK8/STAT1/CDK1/HMOX1/CYP1B1/GSTP1/AKR1C3/MMP3/EGFR/FOS/MMP2/MMP9/MAPK1/TP53/SOD1/HIF1A/DUOX2/NOS3/HSPB1/COL1A1/MPO/NFE2L2/NQO1/PARP1/HSF1/CHUK/JAK2/ADA/DHFR/HDAC6/KDM6B/NOX4/MCL1/TRPA1/EGLN1/FXN/HBA1/NEIL1/IL6/SIGMAR1/PTK2B/MB/G6PD | 55 |
| GO:0070838 | divalent metal ion transport | 55/412 | 7.19E-24 | 1.07E-21 | PTGS2/ADRA1A/OPRM1/DRD1/OPRD1/BCL2/BAX/ICAM1/PPP3CA/GSTM2/EGF/CAV1/GJA1/CCL2/PRKCB/NOS3/IFNG/CXCL11/CXCL10/ATP2A1/CCR1/F2R/FFAR1/FGF2/NMUR2/LGALS3/TRPA1/GCK/TRPM8/VDR/ITPR1/GRIN1/GRIN2A/GRIN2B/GRIN2C/GRIN2D/GRIN3A/GRIN3B/CACNA1C/CACNA1D/CACNA1F/CACNA1S/CACNB1/CACNB2/CACNB3/CACNB4/TRPV3/TRPV1/GPER1/HSPA2/PIK3CG/PTK2B/G6PD/PLA2G1B/CYP27B1 | 55 |
| GO:0072511 | divalent inorganic cation transport | 55/412 | 1.31E-23 | 1.77E-21 | PTGS2/ADRA1A/OPRM1/DRD1/OPRD1/BCL2/BAX/ICAM1/PPP3CA/GSTM2/EGF/CAV1/GJA1/CCL2/PRKCB/NOS3/IFNG/CXCL11/CXCL10/ATP2A1/CCR1/F2R/FFAR1/FGF2/NMUR2/LGALS3/TRPA1/GCK/TRPM8/VDR/ITPR1/GRIN1/GRIN2A/GRIN2B/GRIN2C/GRIN2D/GRIN3A/GRIN3B/CACNA1C/CACNA1D/CACNA1F/CACNA1S/CACNB1/CACNB2/CACNB3/CACNB4/TRPV3/TRPV1/GPER1/HSPA2/PIK3CG/PTK2B/G6PD/PLA2G1B/CYP27B1 | 55 |
| GO:0006816 | calcium ion transport | 54/412 | 2.57E-25 | 5.01E-23 | PTGS2/ADRA1A/OPRM1/DRD1/OPRD1/BCL2/BAX/ICAM1/PPP3CA/GSTM2/EGF/CAV1/GJA1/CCL2/PRKCB/NOS3/CXCL11/CXCL10/ATP2A1/CCR1/F2R/FFAR1/FGF2/NMUR2/LGALS3/TRPA1/GCK/TRPM8/VDR/ITPR1/GRIN1/GRIN2A/GRIN2B/GRIN2C/GRIN2D/GRIN3A/GRIN3B/CACNA1C/CACNA1D/CACNA1F/CACNA1S/CACNB1/CACNB2/CACNB3/CACNB4/TRPV3/TRPV1/GPER1/HSPA2/PIK3CG/PTK2B/G6PD/PLA2G1B/CYP27B1 | 54 |
| GO:0008202 | steroid metabolic process | 53/412 | 2.62E-30 | 2.21E-27 | RXRA/ESR1/PPARD/AKR1B1/HSD3B2/HSD3B1/CYP3A4/CYP1A2/CYP1A1/NR1I2/CYP1B1/AKR1C3/POR/SOD1/ACACA/IL1B/SULT1E1/IFNG/SPP1/PON1/CYP19A1/NPC1L1/HSD11B1/SERPINA6/GBA/GC/HMGCR/CYP17A1/RORC/FGF1/CYP2C19/SRD5A1/SRD5A2/UGT2B7/COMT/SOAT1/SOAT2/VDR/AKR1C1/AKR1C2/HSD11B2/HSD17B1/NFKB1/SULT2A1/SULT2B1/LSS/RORA/NR1H4/AKR1D1/HSD17B11/CES1/G6PD/CYP27B1 | 53 |
| GO:0035690 | cellular response to drug | 53/412 | 6.42E-28 | 2.71E-25 | PTGS2/CHRM3/CHRM1/CHRM2/OPRM1/NCOA1/NOS2/CDK2/CCNA2/RELA/DRD1/KCNH2/CHRM5/CHRM4/KDR/MET/DPEP1/CDK1/HMOX1/ICAM1/CYP1B1/AHR/PPP3CA/GSTM2/MMP3/EGFR/CASP9/MAPK1/TP53/SOD1/HSPA5/MYC/IL1B/NFE2L2/NQO1/CHEK2/HSF1/NR3C1/FOLR1/FOLR2/HDAC6/GLRA1/GLRA2/KDM6B/SRD5A1/FXN/TFRC/IL6/NFKB1/SIGMAR1/CACNA1S/TRPV1/ACTB | 53 |
| GO:0032496 | response to lipopolysaccharide | 50/412 | 1.81E-27 | 6.55E-25 | PTGS2/OPRM1/NOS2/MAPK14/PPARD/MAOB/RELA/JUN/AKT1/CASP3/MAPK8/CYP1A2/CYP1A1/ICAM1/SELE/VCAM1/GSTP1/SLPI/FOS/CASP9/MAPK1/NFKBIA/CASP8/PRKCA/GJA1/IL1B/CCL2/CXCL8/NOS3/THBD/SERPINE1/MPO/CXCL11/CXCL2/HSF1/CXCL10/CHUK/JAK2/F2R/PTGES/COMT/IL6/NFKB1/NFKB2/OPRK1/NR1H4/CEBPB/HCK/RARA/CYP27B1 | 50 |
| GO:0002237 | response to molecule of bacterial origin | 50/412 | 1.13E-26 | 3.19E-24 | PTGS2/OPRM1/NOS2/MAPK14/PPARD/MAOB/RELA/JUN/AKT1/CASP3/MAPK8/CYP1A2/CYP1A1/ICAM1/SELE/VCAM1/GSTP1/SLPI/FOS/CASP9/MAPK1/NFKBIA/CASP8/PRKCA/GJA1/IL1B/CCL2/CXCL8/NOS3/THBD/SERPINE1/MPO/CXCL11/CXCL2/HSF1/CXCL10/CHUK/JAK2/F2R/PTGES/COMT/IL6/NFKB1/NFKB2/OPRK1/NR1H4/CEBPB/HCK/RARA/CYP27B1 | 50 |
| GO:0009410 | response to xenobiotic stimulus | 49/412 | 4.99E-29 | 2.53E-26 | OPRM1/PTGS1/PPARG/CCNA2/F7/RELA/HTR3A/DRD1/SLC6A3/DPEP1/CYP3A4/CYP1A2/CYP1A1/ICAM1/NR1I2/CYP1B1/GSTP1/AHR/PPP3CA/GSTM1/GSTM2/EGFR/CASP9/RB1/POR/SOD1/HSPA5/NQO1/CHEK2/HSF1/E2F1/NR3C1/NQO2/ADA/GBA/EPHX1/RORC/CYP2C19/SRD5A1/CES2/TRDMT1/AKR1C1/GRIN1/GRIN2A/OPRK1/RORA/CACNA1S/PTK2B/CES1 | 49 |
| GO:0070482 | response to oxygen levels | 48/412 | 3.59E-22 | 4.11E-20 | PTGS2/DPP4/NOS2/PPARG/CCNA2/PPARD/F7/OPRD1/SLC6A4/AKT1/BCL2/CASP3/HMOX1/CYP1A1/ICAM1/VCAM1/PSMD3/SLC2A4/VEGFA/CDKN1A/PLAU/MMP2/TP53/RAF1/HIF1A/CAV1/MYC/CCNB1/PLAT/COL1A1/NFE2L2/PPARA/HSF1/E2F1/NPEPPS/HK2/CA9/ADA/NOX4/EGLN1/ITPR1/POLB/TGFBR2/HSD11B2/RORA/CACNB3/PTK2B/MB | 48 |
| GO:0048871 | multicellular organismal homeostasis | 48/412 | 2.30E-18 | 1.49E-16 | PTGS2/ACHE/ADRB2/AKR1B1/ADRB1/DRD1/MET/IL4R/BCL2/BAX/HAS2/EGFR/VEGFA/RB1/SOD1/PRKCA/ACACA/CAV1/GJA1/IL1B/PTGER3/NOS3/HSPB1/IL1A/CLDN4/HSF1/SPP1/JAK2/ADRB3/CA2/GBA/F2R/KDM6B/NOX4/CTSK/OGT/TRPM8/TF/TFRC/IL6/TRPV1/ACTB/CEBPB/ESRRB/PTK2B/ACOT13/RHO/ESRRG | 48 |
| GO:0001101 | response to acid chemical | 47/412 | 6.32E-24 | 9.99E-22 | PTGS2/RXRA/NCOA1/PPARG/AKR1B1/F7/RELA/SLC6A4/RXRB/KDR/AKT1/CASP3/ICAM1/GSTP1/AKR1C3/EGFR/VEGFA/BCL2L1/MMP2/ACACA/GJA1/CCNB1/COL1A1/NQO1/COL3A1/HSF1/CHUK/E2F1/PON1/TYMS/DHFR/FOLR1/FOLR2/FFAR1/GLRA1/GLRA2/PTGES/AKR1C1/AKR1C2/GRIN1/NR1H4/CEBPB/PTK2B/RARA/RARB/RARG/RXRG | 47 |
| GO:0010038 | response to metal ion | 47/412 | 8.33E-23 | 1.06E-20 | PTGS2/SCN5A/MAOB/NCF1/SLC6A3/DPEP1/JUN/AKT1/BCL2/CASP3/MAPK8/CDK1/HMOX1/CYP1A2/CYP1A1/ICAM1/VCAM1/PPP3CA/AKR1C3/EGFR/CCND1/FOS/CASP9/MMP9/MAPK1/CASP8/SOD1/HIF1A/HSPA5/CAV1/CCNB1/IL1A/NFE2L2/NQO1/PARP1/HSF1/CHUK/CA2/GLRA1/GLRA2/BACE1/PTGES/CYB5A/FXN/TF/PTK2B/G6PD | 47 |
| GO:0062012 | regulation of small molecule metabolic process | 47/412 | 1.35E-18 | 9.79E-17 | PTGS2/NCOA2/NOS2/PPARG/GSK3B/AKT1/CDK1/HAS2/PSMD3/INSR/AKR1C3/EIF6/EGF/TP53/POR/ODC1/SOD1/HIF1A/ACACA/CAV1/IL1B/NOS3/CCNB1/IFNG/NQO1/PARP1/PPARA/IGFBP3/IGF2/HMGCR/RORC/FGF1/PTPN2/CDA/OGT/GCK/COMT/VDR/NFKB1/LSS/RORA/NR1H4/ESRRB/GPER1/PTK2B/COX7A1/CYP27B1 | 47 |
| GO:0072593 | reactive oxygen species metabolic process | 46/412 | 1.33E-26 | 3.36E-24 | PTGS2/NOS2/MAPK14/MAOB/NCF1/AKT1/BCL2/CYP1A2/CYP1A1/ICAM1/CYP1B1/GSTP1/INSR/AKR1C3/MMP3/EGFR/CDKN1A/EIF6/TP53/POR/SOD1/HIF1A/CAV1/IL1B/DUOX2/NOS3/IFNG/MPO/NFE2L2/NQO1/HK2/JAK2/VAV1/NQO2/MMP8/DHFR/HDAC6/HSP90AA1/NOX4/HBA1/TGFBR2/GRIN1/RORA/TRPV1/PTK2B/G6PD | 46 |
| GO:0036293 | response to decreased oxygen levels | 44/412 | 5.64E-20 | 5.06E-18 | PTGS2/DPP4/NOS2/CCNA2/PPARD/F7/OPRD1/SLC6A4/AKT1/BCL2/CASP3/HMOX1/CYP1A1/ICAM1/VCAM1/PSMD3/SLC2A4/VEGFA/PLAU/MMP2/TP53/RAF1/HIF1A/CAV1/MYC/CCNB1/PLAT/NFE2L2/PPARA/HSF1/E2F1/NPEPPS/HK2/CA9/ADA/NOX4/EGLN1/ITPR1/TGFBR2/HSD11B2/RORA/CACNB3/PTK2B/MB | 44 |
| GO:0009314 | response to radiation | 44/412 | 8.80E-17 | 4.55E-15 | PTGS2/OPRM1/CHEK1/MAPK14/RELA/DRD1/JUN/AKT1/BCL2/BAX/CASP3/MAPK8/ICAM1/VCAM1/EGFR/CCND1/BCL2L1/FOS/CDKN1A/CASP9/TP53/ELK1/HIF1A/HSPA5/MYC/THBD/PARP1/COL3A1/CHEK2/HSF1/CXCL10/TYR/HMGCR/BACE1/NOX4/FEN1/POLB/GRIN1/GRIN2A/OPRK1/CACNA1F/RUVBL2/RHO/FECH | 44 |
| GO:0000302 | response to reactive oxygen species | 43/412 | 2.17E-27 | 6.85E-25 | CDK2/CCNA2/RELA/NCF1/MET/DPEP1/JUN/AKT1/BCL2/CASP3/MAPK8/STAT1/CDK1/HMOX1/CYP1B1/GSTP1/AKR1C3/MMP3/EGFR/FOS/MMP2/MMP9/MAPK1/SOD1/NOS3/COL1A1/MPO/NFE2L2/NQO1/HSF1/CHUK/ADA/DHFR/HDAC6/KDM6B/TRPA1/EGLN1/FXN/HBA1/IL6/SIGMAR1/PTK2B/MB | 43 |
| GO:0001666 | response to hypoxia | 43/412 | 1.16E-19 | 9.80E-18 | PTGS2/DPP4/NOS2/CCNA2/PPARD/F7/OPRD1/SLC6A4/AKT1/BCL2/CASP3/HMOX1/CYP1A1/ICAM1/VCAM1/PSMD3/SLC2A4/VEGFA/PLAU/MMP2/TP53/RAF1/HIF1A/CAV1/MYC/CCNB1/PLAT/NFE2L2/PPARA/HSF1/E2F1/NPEPPS/HK2/CA9/ADA/NOX4/EGLN1/ITPR1/TGFBR2/HSD11B2/RORA/PTK2B/MB | 43 |
| GO:0010959 | regulation of metal ion transport | 43/412 | 4.04E-18 | 2.49E-16 | PTGS2/SCN5A/ADRB2/DRD1/KCNH2/OPRD1/AKT1/BCL2/BAX/ICAM1/GSTM2/EGF/CAV1/GJA1/CCL2/NOS3/IFNG/CXCL11/CXCL10/ATP2A1/CCR1/F2R/FFAR1/LGALS3/GCK/ARF1/TF/GRIN1/GRIN3B/OPRK1/CACNA1C/CACNA1D/CACNB1/CACNB2/CACNB3/CACNB4/TRPV3/GPER1/HSPA2/PIK3CG/PTK2B/G6PD/PLA2G1B | 43 |
| GO:0034599 | cellular response to oxidative stress | 42/412 | 1.02E-21 | 1.03E-19 | CDK2/CCNA2/RELA/NCF1/MET/DPEP1/JUN/AKT1/BCL2/MAPK8/CDK1/HMOX1/CYP1B1/GSTP1/AKR1C3/MMP3/EGFR/FOS/MMP2/MMP9/MAPK1/TP53/SOD1/HIF1A/NOS3/HSPB1/MPO/NFE2L2/NQO1/PARP1/HSF1/CHUK/JAK2/DHFR/HDAC6/KDM6B/NOX4/MCL1/FXN/IL6/SIGMAR1/G6PD | 42 |
| GO:0070997 | neuron death | 42/412 | 2.38E-19 | 1.83E-17 | GSK3B/JUN/AKT1/BCL2/BAX/CASP3/HMOX1/BCL2L1/FOS/CASP9/RB1/TP53/ELK1/CASP8/SOD1/HIF1A/HSPA5/CCL2/IFNG/NQO1/PARP1/PPARA/HSF1/ERBB3/RASA1/JAK2/NQO2/GBA/F2R/BACE1/MCL1/GRM4/GABRA5/POLB/GABRB2/GABRB3/GRIN1/GRIN2B/SIGMAR1/CEBPB/PTK2B/G6PD | 42 |
| GO:0048732 | gland development | 42/412 | 7.81E-16 | 3.66E-14 | PGR/AR/RXRA/NCOA1/ESR1/RELA/SLC6A3/MET/JUN/AKT1/BCL2/BAX/HMOX1/CYP1A1/INSR/EGFR/VEGFA/CCND1/MAPK1/EGF/RAF1/SOD1/HIF1A/CAV1/GJA1/IGF2/HK2/CYP19A1/JAK2/TYR/ADA/TYMS/FPGS/SRD5A1/VDR/POLB/TGFBR2/CEBPB/CSNK2B/RARA/RARB/RARG | 42 |
| GO:0097305 | response to alcohol | 41/412 | 3.07E-25 | 5.55E-23 | OPRM1/PPARG/F7/MAOB/HTR3A/SLC6A3/HSD3B2/HSD3B1/AKT1/CDK1/ICAM1/VCAM1/GSTP1/AHR/SLC2A4/AKR1C3/CCND1/BCL2L1/FOS/CDKN1A/CASP8/SOD1/ACACA/IL2/NQO1/PARP1/TYMS/HMGCR/GLRA1/GLRA2/POLB/TGFBR2/AKR1C2/GRIN1/GRIN2A/GRIN2B/GRIN3A/OPRK1/PTK2B/RARA/G6PD | 41 |
| GO:0043434 | response to peptide hormone | 41/412 | 4.74E-15 | 1.96E-13 | PTGS2/PPARG/GSK3B/CCNA2/F7/RELA/AKT1/STAT1/ICAM1/GSTP1/SLC2A4/INSR/EIF6/POR/CAV1/GJA1/IL1B/PRKCB/COL1A1/NFE2L2/PARP1/PPARA/HSF1/CHUK/IGF2/PTPN1/JAK3/JAK2/CA2/PTPN2/OGT/SRD5A1/GCK/HSD11B2/NFKB1/OPRK1/NR1H4/TRPV1/ESRRA/GPER1/PLA2G1B | 41 |
| GO:0071496 | cellular response to external stimulus | 40/412 | 4.02E-18 | 2.49E-16 | PTGS2/NCOA1/CHEK1/PPARG/JUN/AKT1/BCL2/MAPK8/HMOX1/ICAM1/VCAM1/GSTP1/AKR1C3/EGFR/FOS/CDKN1A/MAPK1/TP53/CASP8/SOD1/HSPA5/GJA1/IL1B/COL1A1/NFE2L2/HSF1/IRF1/HSPA8/GBA/FOLR1/FOLR2/SRD5A1/COMT/VDR/NFKB1/NR1H4/CACNB3/TRPV1/PIM1/CYP27B1 | 40 |
| GO:0001505 | regulation of neurotransmitter levels | 40/412 | 1.87E-17 | 1.08E-15 | PTGS2/ACHE/ADRA1A/CHRM2/NOS2/SLC6A2/GSK3B/MAOB/DRD1/SLC6A3/SLC6A4/AKT1/ICAM1/CYP1B1/INSR/POR/CAV1/IL1B/PRKCB/NOS3/IFNG/NQO1/JAK2/SLC29A1/MMP8/HSPA8/HSP90AA1/BACE1/SRD5A1/GRM4/COMT/GABRA2/GRIN2A/GRIN3A/RORA/CACNA1D/CACNB2/TRPV1/GPER1/PTK2B | 40 |
| GO:0006066 | alcohol metabolic process | 40/412 | 5.00E-17 | 2.65E-15 | RXRA/ADH1B/ADH1C/PPARD/AKR1B1/CYP3A4/CYP1A2/CYP1A1/CYP1B1/AKR1C3/POR/SOD1/ACACA/IL1B/SULT1E1/IFNG/PON1/NPC1L1/GBA/DHFR/HMGCR/FGF1/FGF2/SOAT1/SOAT2/VDR/ISYNA1/AKR1C1/AKR1C2/NFKB1/SULT2A1/SULT2B1/LSS/NR1H4/AKR1D1/GPER1/PTK2B/CES1/G6PD/CYP27B1 | 40 |
| GO:0048608 | reproductive structure development | 40/412 | 1.62E-14 | 5.84E-13 | PTGS2/PGR/AR/RXRA/NCOA1/ESR1/PPARG/MAPK14/PPARD/AKT1/BCL2/BAX/CASP3/ICAM1/INSR/AKR1C3/EGFR/VEGFA/CCND1/BCL2L1/MAPK1/CASP8/SOD1/HIF1A/HSPA5/GJA1/NOS3/HSF1/SPP1/IGF2/CYP19A1/ADA/SRD5A1/SRD5A2/VDR/CEBPB/RARA/RARG/HOXA10/CYP27B1 | 40 |
| GO:0061458 | reproductive system development | 40/412 | 2.04E-14 | 7.06E-13 | PTGS2/PGR/AR/RXRA/NCOA1/ESR1/PPARG/MAPK14/PPARD/AKT1/BCL2/BAX/CASP3/ICAM1/INSR/AKR1C3/EGFR/VEGFA/CCND1/BCL2L1/MAPK1/CASP8/SOD1/HIF1A/HSPA5/GJA1/NOS3/HSF1/SPP1/IGF2/CYP19A1/ADA/SRD5A1/SRD5A2/VDR/CEBPB/RARA/RARG/HOXA10/CYP27B1 | 40 |
| GO:0007584 | response to nutrient | 39/412 | 2.98E-24 | 5.03E-22 | PTGS2/NCOA1/PPARG/PPARD/F7/RELA/SLC6A4/STAT1/HMOX1/CYP1A1/VCAM1/GSTP1/AKR1C3/EGFR/CCND1/POR/SOD1/IL1B/COL1A1/NQO1/HSF1/CXCL10/SPP1/TYR/ADA/TYMS/FOLR1/FOLR2/HMGCR/OGT/VDR/TGFBR2/GRIN1/NR1H4/TRPV1/PIM1/RARA/COX4I1/CYP27B1 | 39 |
| GO:1901214 | regulation of neuron death | 39/412 | 1.59E-18 | 1.09E-16 | GSK3B/JUN/AKT1/BCL2/BAX/CASP3/HMOX1/BCL2L1/FOS/CASP9/TP53/ELK1/CASP8/SOD1/HIF1A/CCL2/IFNG/NQO1/PARP1/PPARA/HSF1/ERBB3/RASA1/JAK2/NQO2/GBA/F2R/BACE1/MCL1/GRM4/GABRA5/GABRB2/GABRB3/GRIN1/GRIN2B/SIGMAR1/CEBPB/PTK2B/G6PD | 39 |
| GO:0019932 | second-messenger-mediated signaling | 39/412 | 1.44E-13 | 4.00E-12 | ADRA1A/ADRB2/OPRM1/NOS2/ADRA1B/GSK3B/ADRB1/ADRA2C/ADRA1D/DRD1/KDR/SELE/VCAM1/AHR/PPP3CA/GSTM2/EGFR/VEGFA/PRKCA/PTGER3/CXCL8/NOS3/CXCL11/CXCL10/ERBB3/ADRB3/ADA/CCR1/NMUR2/ITPR1/GRIN1/GRIN2A/GRIN2B/GRIN2C/GRIN2D/CACNA1C/GPER1/PTK2B/NPPB | 39 |
| GO:0042110 | T cell activation | 39/412 | 8.31E-13 | 1.88E-11 | DPP4/IL4R/AKT1/BCL2/BAX/CASP3/ICAM1/VCAM1/PPP3CA/IL6ST/TP53/CASP8/SOD1/ERBB2/CAV1/GJA1/IL1B/CCL2/IL2/IFNG/RUNX2/IGF2/CD40LG/IRF1/JAK3/VAV1/ADA/RORC/LGALS3/PTPN2/TFRC/TGFBR2/IL6/CLEC4E/RORA/CDK6/CEBPB/PIK3CG/RARA | 39 |
| GO:0055074 | calcium ion homeostasis | 39/412 | 1.33E-12 | 2.93E-11 | ADRA1A/ADRA1B/ESR1/DRD1/BCL2/BAX/GSTM2/CAV1/GJA1/PTGER3/PRKCB/IL2/CXCL11/CXCL10/JAK2/ATP2A1/CCR1/F2R/FGF2/NMUR2/TRPA1/TRPM8/VDR/ITPR1/GRIN1/GRIN2A/GRIN2B/GRIN2C/GRIN2D/CACNA1C/CACNA1D/CACNB2/CACNB3/TRPV1/GPER1/PIK3CG/PTK2B/PLA2G1B/CYP27B1 | 39 |
| GO:0034765 | regulation of ion transmembrane transport | 39/412 | 2.90E-12 | 6.18E-11 | SCN5A/ADRB2/OPRM1/GRIA2/HTR3A/DRD1/KCNH2/AKT1/BAX/GSTM2/MMP9/CAV1/GJA1/CCL2/IFNG/CXCL11/CXCL10/ATP2A1/ABCB1/F2R/CTSS/ARF1/GRIN1/GRIN2A/GRIN2B/OPRK1/CACNA1C/CACNA1D/CACNA1F/CACNA1S/CACNB1/CACNB2/CACNB3/CACNB4/GPER1/HSPA2/PIK3CG/PTK2B/G6PD | 39 |
| GO:0050727 | regulation of inflammatory response | 39/412 | 3.30E-12 | 6.85E-11 | PTGS2/NOS2/IGHG1/ESR1/PPARG/MAPK14/PPARD/RELA/TNFAIP6/SELE/GSTP1/MMP3/EGFR/MMP9/RB1/IL6ST/NFKBIA/SOD1/IL1B/PTGER3/IL2/SERPINE1/PPARA/CYP19A1/JAK2/MMP8/ADA/GBA/PTPN2/CTSC/IL6/NFKB1/RORA/NR1H4/IGHG2/CEBPB/GPER1/HCK/PIK3CG | 39 |
| GO:0042445 | hormone metabolic process | 38/412 | 2.63E-22 | 3.18E-20 | ACHE/ADH1B/ADH1C/ESR1/AKR1B1/HSD3B2/HSD3B1/CYP3A4/CYP1A2/CYP1A1/CYP1B1/DIO1/AKR1C3/POR/HIF1A/IL1B/DUOX2/SULT1E1/IFNG/SPP1/CYP19A1/HSD11B1/SERPINA6/CYP17A1/SRD5A1/SRD5A2/UGT2B7/COMT/VDR/AKR1C1/AKR1C2/HSD11B2/HSD17B1/NFKB1/AKR1D1/HSD17B11/CES1/CYP27B1 | 38 |
| GO:0048511 | rhythmic process | 38/412 | 1.42E-18 | 9.97E-17 | PGR/OPRM1/NCOA2/NCOA1/NOS2/ESR1/PPARG/GSK3B/F7/SLC6A4/JUN/CASP3/MAPK8/CDK1/HAS2/AHR/EGFR/TP53/TOP1/HSPA5/NOS3/SERPINE1/TOP2A/CLDN4/PPARA/ADA/TYMS/HDAC1/RORC/KDM2A/KDM5C/OGT/SRD5A1/GABRB1/NFKB2/OPRK1/RORA/CSNK2A1 | 38 |
| GO:2001233 | regulation of apoptotic signaling pathway | 38/412 | 5.82E-14 | 1.77E-12 | PTGS2/AR/GSK3B/RELA/AKT1/BCL2/BAX/MAPK8/HMOX1/ICAM1/GSTP1/BCL2L1/MMP9/RB1/TP53/CASP8/RAF1/SOD1/HIF1A/CAV1/IL1B/NOS3/HSPB1/SERPINE1/IL1A/NFE2L2/PARP1/E2F1/PTPN1/JAK2/HDAC1/MCL1/LGALS3/PTPN2/CTSC/FXN/CSNK2A1/GPER1 | 38 |
| GO:0006874 | cellular calcium ion homeostasis | 38/412 | 2.47E-12 | 5.29E-11 | ADRA1A/ADRA1B/ESR1/DRD1/BCL2/BAX/GSTM2/CAV1/GJA1/PTGER3/PRKCB/IL2/CXCL11/CXCL10/JAK2/ATP2A1/CCR1/F2R/FGF2/NMUR2/TRPA1/TRPM8/VDR/ITPR1/GRIN1/GRIN2A/GRIN2B/GRIN2C/GRIN2D/CACNA1C/CACNA1D/CACNB2/CACNB3/TRPV1/GPER1/PIK3CG/PTK2B/PLA2G1B | 38 |
| GO:0003012 | muscle system process | 38/412 | 3.91E-12 | 8.04E-11 | PTGS2/CHRM3/SCN5A/ADRA1A/CHRM2/ADRB2/ADRA1B/ADRA2C/DRD1/KCNH2/HMOX1/PPP3CA/GSTM2/IL6ST/SOD1/PRKCA/CAV1/GJA1/IL1B/PTGER3/NOS3/PARP1/PPARA/CHUK/ATP2A1/ADA/F2R/GLRA1/NMUR2/CACNA1C/CACNA1D/CACNA1S/CACNB2/TRPV1/GPER1/PIK3CG/MB/G6PD | 38 |
| GO:0072503 | cellular divalent inorganic cation homeostasis | 38/412 | 2.25E-11 | 4.04E-10 | ADRA1A/ADRA1B/ESR1/DRD1/BCL2/BAX/GSTM2/CAV1/GJA1/PTGER3/PRKCB/IL2/CXCL11/CXCL10/JAK2/ATP2A1/CCR1/F2R/FGF2/NMUR2/TRPA1/TRPM8/VDR/ITPR1/GRIN1/GRIN2A/GRIN2B/GRIN2C/GRIN2D/CACNA1C/CACNA1D/CACNB2/CACNB3/TRPV1/GPER1/PIK3CG/PTK2B/PLA2G1B | 38 |
| GO:0071383 | cellular response to steroid hormone stimulus | 37/412 | 3.47E-20 | 3.37E-18 | PGR/AR/RXRA/NCOA1/ESR2/ESR1/PPARG/PPARD/RXRB/ICAM1/NR1I2/GSTP1/NR1I3/AKR1C3/EGFR/CASP9/RB1/PARP1/PPARA/JAK2/NR3C1/HDAC6/HDAC1/RORC/SRD5A1/VDR/NR3C2/RORA/NR1H4/ESRRA/ESRRB/GPER1/RARA/RARB/RARG/RXRG/ESRRG | 37 |
| GO:0030522 | intracellular receptor signaling pathway | 37/412 | 1.73E-18 | 1.15E-16 | PGR/AR/RXRA/NCOA1/ESR2/ESR1/PPARG/PPARD/RELA/RXRB/NR1I2/AHR/NR1I3/AKR1C3/RB1/NFKBIA/CASP8/PARP1/PPARA/JAK2/NR3C1/HDAC6/HDAC1/RORC/VDR/RORA/NR1H4/ESRRA/ESRRB/GPER1/PIM1/RARA/RARB/RARG/RXRG/ESRRG/CYP27B1 | 37 |
| GO:1901653 | cellular response to peptide | 37/412 | 5.59E-14 | 1.73E-12 | ADRB2/PPARG/GSK3B/CCNA2/AKR1B1/RELA/AKT1/STAT1/ICAM1/VCAM1/GSTP1/SLC2A4/INSR/TP53/POR/CAV1/IL1B/PRKCB/NFE2L2/PARP1/HSF1/IGF2/PTPN1/JAK3/JAK2/CA2/BACE1/PTPN2/OGT/SRD5A1/GCK/NFKB1/NR1H4/CACNB1/ESRRA/GPER1/PLA2G1B | 37 |
| GO:0030099 | myeloid cell differentiation | 37/412 | 6.05E-13 | 1.43E-11 | PPARG/MAPK14/JUN/CASP3/STAT1/VEGFA/FOS/EIF6/CASP9/MMP9/RB1/NFKBIA/CASP8/PRKCA/HIF1A/MYC/PRKCB/IFNG/PARP1/JAK3/JAK2/CA2/CCR1/HDAC1/LGALS3/PTPN2/AHSP/TFRC/TGFBR2/CDK6/CEBPB/ESRRA/PTK2B/MB/RARA/RARG/G6PD | 37 |
| GO:1904951 | positive regulation of establishment of protein localization | 37/412 | 9.39E-12 | 1.76E-10 | PTGS2/CHRM1/ACHE/MAPK14/GSK3B/PPARD/IL4R/BCL2/MAPK8/EGFR/MAPK1/TP53/CASP8/HIF1A/ERBB2/GJA1/IL1B/IL2/IFNG/IL1A/CHUK/E2F1/NPEPPS/JAK2/MMP8/F2R/FFAR1/GCK/ARF1/PAEP/IL6/ORM1/CLEC4E/NR1H4/CACNB3/GPER1/PLA2G1B | 37 |
| GO:0023061 | signal release | 36/412 | 5.73E-11 | 9.36E-10 | ADRA1A/CHRM2/OPRM1/DPP4/NOS2/GSK3B/PPARD/ADRA2C/DRD1/PPP3CA/EGFR/RAF1/PRKCA/HIF1A/GJA1/IL1B/PRKCB/IFNG/SPP1/CYP19A1/JAK2/HSPA8/HMGCR/FFAR1/BACE1/GRM4/GCK/ITPR1/GRIN3A/IL6/OPRK1/NR1H4/CACNA1C/CACNA1D/CACNB2/GPER1 | 36 |
| GO:0050708 | regulation of protein secretion | 36/412 | 1.04E-10 | 1.60E-09 | ACHE/OPRM1/DPP4/NOS2/MAPK14/PPARD/ADRA2C/IL4R/PPP3CA/EGFR/PRKCA/HIF1A/GJA1/IL1B/IL2/IFNG/IL1A/CHUK/CD40LG/JAK2/MMP8/F2R/HMGCR/FFAR1/GCK/ARF1/ITPR1/PAEP/IL6/ORM1/CLEC4E/NR1H4/CACNA1C/CACNA1D/GPER1/PLA2G1B | 36 |
| GO:0002791 | regulation of peptide secretion | 36/412 | 5.12E-10 | 6.65E-09 | ACHE/OPRM1/DPP4/NOS2/MAPK14/PPARD/ADRA2C/IL4R/PPP3CA/EGFR/PRKCA/HIF1A/GJA1/IL1B/IL2/IFNG/IL1A/CHUK/CD40LG/JAK2/MMP8/F2R/HMGCR/FFAR1/GCK/ARF1/ITPR1/PAEP/IL6/ORM1/CLEC4E/NR1H4/CACNA1C/CACNA1D/GPER1/PLA2G1B | 36 |
| GO:1901654 | response to ketone | 35/412 | 3.86E-22 | 4.11E-20 | AR/NCOA2/NCOA1/PPARG/AKR1B1/F7/MAOB/RELA/HSD3B2/HSD3B1/AKT1/ICAM1/AHR/AKR1C3/EGFR/CCND1/BCL2L1/FOS/CDKN1A/CASP9/ELK1/ACACA/CAV1/PARP1/CLDN4/HSF1/SPP1/NR3C1/CA9/GBA/TYMS/SRD5A1/AKR1C2/GABRB1/CSNK2B | 35 |
| GO:0043270 | positive regulation of ion transport | 35/412 | 5.03E-17 | 2.65E-15 | CHRM1/SCN5A/ADRB2/HTR3A/DRD1/KCNH2/SLC6A4/AKT1/BAX/GSTM2/CAV1/IL1B/CCL2/IFNG/CXCL11/CXCL10/ATP2A1/ABCB1/CCR1/F2R/FFAR1/CTSS/LGALS3/ARF1/GRIN1/OPRK1/CACNA1D/CACNB2/CACNB3/TRPV3/CEBPB/GPER1/HSPA2/G6PD/PLA2G1B | 35 |
| GO:0051090 | regulation of DNA-binding transcription factor activity | 35/412 | 3.67E-11 | 6.31E-10 | AR/ESR2/ESR1/PPARG/MAPK14/RELA/OPRD1/JUN/IKBKB/AKT1/MAPK8/HMOX1/ICAM1/CYP1B1/PPP3CA/VEGFA/FOS/MAPK1/RB1/NFKBIA/CAV1/IL1B/PRKCB/CHUK/CD40LG/RPS6KA5/JAK2/EGLN1/IL6/NFKB1/NFKB2/NR1H4/HCK/PIM1/PLA2G1B | 35 |
| GO:1903706 | regulation of hemopoiesis | 35/412 | 4.87E-10 | 6.39E-09 | MAPK14/JUN/IL4R/STAT1/PSMD3/FOS/EIF6/RB1/NFKBIA/CASP8/SOD1/PRKCA/HIF1A/ERBB2/MYC/PRKCB/IL2/IFNG/NFE2L2/IRF1/JAK3/CA2/ADA/CCR1/HDAC1/LGALS3/PTPN2/TGFBR2/CDK6/CEBPB/ESRRA/PIM1/PTK2B/RARA/RARG | 35 |
| GO:0071466 | cellular response to xenobiotic stimulus | 34/412 | 3.89E-22 | 4.11E-20 | OPRM1/PTGS1/CCNA2/DPEP1/CYP3A4/CYP1A2/CYP1A1/ICAM1/NR1I2/CYP1B1/GSTP1/AHR/GSTM1/GSTM2/EGFR/CASP9/RB1/POR/NQO1/CHEK2/HSF1/E2F1/NR3C1/NQO2/EPHX1/RORC/CYP2C19/SRD5A1/CES2/AKR1C1/GRIN1/RORA/CACNA1S/CES1 | 34 |
| GO:0051924 | regulation of calcium ion transport | 34/412 | 2.98E-17 | 1.67E-15 | PTGS2/DRD1/OPRD1/BCL2/BAX/ICAM1/GSTM2/EGF/CAV1/GJA1/CCL2/NOS3/CXCL11/CXCL10/ATP2A1/CCR1/F2R/FFAR1/LGALS3/GRIN1/GRIN3B/CACNA1C/CACNA1D/CACNB1/CACNB2/CACNB3/CACNB4/TRPV3/GPER1/HSPA2/PIK3CG/PTK2B/G6PD/PLA2G1B | 34 |
| GO:0070588 | calcium ion transmembrane transport | 34/412 | 2.10E-14 | 7.19E-13 | OPRM1/DRD1/BAX/GSTM2/CXCL11/CXCL10/ATP2A1/F2R/FGF2/TRPA1/TRPM8/ITPR1/GRIN1/GRIN2A/GRIN2B/GRIN2C/GRIN2D/GRIN3A/GRIN3B/CACNA1C/CACNA1D/CACNA1F/CACNA1S/CACNB1/CACNB2/CACNB3/CACNB4/TRPV3/TRPV1/GPER1/HSPA2/PIK3CG/PTK2B/G6PD | 34 |
| GO:0007204 | positive regulation of cytosolic calcium ion concentration | 34/412 | 3.05E-14 | 1.00E-12 | ADRA1A/ADRA1B/ESR1/DRD1/BCL2/BAX/GSTM2/CAV1/GJA1/PTGER3/IL2/CXCL11/CXCL10/JAK2/CCR1/F2R/FGF2/NMUR2/TRPA1/ITPR1/GRIN1/GRIN2A/GRIN2B/GRIN2C/GRIN2D/CACNA1C/CACNA1D/CACNB2/CACNB3/TRPV1/GPER1/PIK3CG/PTK2B/PLA2G1B | 34 |
| GO:0007568 | aging | 34/412 | 3.67E-14 | 1.19E-12 | PTGS2/ADRA1A/CHEK1/MAPK14/RELA/SLC6A3/JUN/AKT1/BCL2/CDK1/CYP1A1/ICAM1/VCAM1/PPP3CA/FOS/CDKN1A/CASP9/MAPK1/TP53/SOD1/SERPINE1/MPO/NFE2L2/NQO1/CHEK2/ADA/TYMS/HMGCR/NOX4/CTSC/POLB/TGFBR2/NFKB2/CDK6 | 34 |
| GO:0051480 | regulation of cytosolic calcium ion concentration | 34/412 | 7.97E-13 | 1.82E-11 | ADRA1A/ADRA1B/ESR1/DRD1/BCL2/BAX/GSTM2/CAV1/GJA1/PTGER3/IL2/CXCL11/CXCL10/JAK2/CCR1/F2R/FGF2/NMUR2/TRPA1/ITPR1/GRIN1/GRIN2A/GRIN2B/GRIN2C/GRIN2D/CACNA1C/CACNA1D/CACNB2/CACNB3/TRPV1/GPER1/PIK3CG/PTK2B/PLA2G1B | 34 |
| GO:0019216 | regulation of lipid metabolic process | 34/412 | 3.77E-11 | 6.44E-10 | PTGS2/RXRA/NCOA2/NCOA1/PPARG/PPARD/CHRM5/AKT1/CYP1A1/AKR1C3/EIF6/RB1/POR/SOD1/ACACA/CAV1/IL1B/IFNG/PPARA/HMGCR/RORC/FGF1/FGF2/VDR/ARF1/NFKB1/SULT2A1/LSS/RORA/NR1H4/GPER1/PIK3CG/PTK2B/CYP27B1 | 34 |
| GO:0050804 | modulation of chemical synaptic transmission | 34/412 | 1.97E-10 | 2.93E-09 | PTGS2/ACHE/ADRA1A/CHRM2/ADRB2/GSK3B/DRD1/SLC6A4/PPP3CA/EGFR/MAPK1/IL1B/CCL2/PRKCB/PLAT/JAK2/CA2/CA7/F2R/BACE1/GRM4/ARF1/GRIN1/GRIN2A/GRIN2B/GRIN2C/GRIN2D/GRIN3A/CACNA1D/CACNB2/CACNB3/GPER1/PTK2B/RARA | 34 |
| GO:0060249 | anatomical structure homeostasis | 34/412 | 2.09E-10 | 3.06E-09 | ADRB2/SLC6A2/AKR1B1/BCL2/BAX/EGFR/VEGFA/MAPK1/RB1/SOD1/PRKCA/HIF1A/ACACA/MYC/NOS3/HSPB1/PARP1/SPP1/CA2/F2R/HDAC8/HSP90AA1/NOX4/CTSK/FEN1/TF/TFRC/IL6/CACNA1D/CACNB2/ACTB/ESRRB/PTK2B/RHO | 34 |
| GO:0099177 | regulation of trans-synaptic signaling | 34/412 | 2.09E-10 | 3.06E-09 | PTGS2/ACHE/ADRA1A/CHRM2/ADRB2/GSK3B/DRD1/SLC6A4/PPP3CA/EGFR/MAPK1/IL1B/CCL2/PRKCB/PLAT/JAK2/CA2/CA7/F2R/BACE1/GRM4/ARF1/GRIN1/GRIN2A/GRIN2B/GRIN2C/GRIN2D/GRIN3A/CACNA1D/CACNB2/CACNB3/GPER1/PTK2B/RARA | 34 |
| GO:2000377 | regulation of reactive oxygen species metabolic process | 33/412 | 5.80E-20 | 5.06E-18 | PTGS2/MAPK14/AKT1/BCL2/ICAM1/CYP1B1/GSTP1/INSR/AKR1C3/MMP3/EGFR/CDKN1A/EIF6/TP53/SOD1/HIF1A/CAV1/IL1B/IFNG/NFE2L2/HK2/JAK2/NQO2/MMP8/DHFR/HDAC6/HSP90AA1/NOX4/TGFBR2/GRIN1/TRPV1/PTK2B/G6PD | 33 |
| GO:0006352 | DNA-templated transcription, initiation | 33/412 | 1.20E-16 | 6.08E-15 | PGR/AR/RXRA/ESR2/ESR1/PPARG/PPARD/RXRB/JUN/BAX/CDK1/NR1I2/NR1I3/CCND1/CDKN1A/TP53/CCNB1/PPARA/RUNX2/E2F2/NR3C1/RORC/VDR/NR3C2/RORA/NR1H4/ESRRA/ESRRB/RARA/RARB/RARG/RXRG/ESRRG | 33 |
| GO:1904062 | regulation of cation transmembrane transport | 33/412 | 1.22E-12 | 2.74E-11 | SCN5A/ADRB2/OPRM1/GRIA2/DRD1/KCNH2/BAX/GSTM2/MMP9/CAV1/CCL2/IFNG/CXCL11/CXCL10/ATP2A1/F2R/CTSS/ARF1/GRIN1/GRIN2A/GRIN2B/OPRK1/CACNA1C/CACNA1D/CACNB1/CACNB2/CACNB3/CACNB4/GPER1/HSPA2/PIK3CG/PTK2B/G6PD | 33 |
| GO:0032102 | negative regulation of response to external stimulus | 33/412 | 7.31E-12 | 1.38E-10 | PPARG/PPARD/DRD1/SLC6A3/TNFAIP6/GSTP1/CDKN1A/PLAU/RB1/SOD1/GJA1/CCL2/NOS3/IL2/PLAT/THBD/SERPINE1/PPARA/SPP1/CYP19A1/ADA/GBA/HMGCR/FGF2/BACE1/PTPN2/GRIN1/GRIN3A/NFKB1/RORA/NR1H4/CACNB3/GPER1 | 33 |
| GO:0022407 | regulation of cell-cell adhesion | 33/412 | 1.03E-10 | 1.59E-09 | DPP4/MAPK14/RELA/IL4R/AKT1/CASP3/ICAM1/VCAM1/HAS2/VEGFA/IL6ST/PRKCA/ERBB2/CAV1/IL1B/CCL2/IL2/IFNG/PPARA/IGF2/CD40LG/IRF1/JAK3/JAK2/VAV1/ADA/LGALS3/PTPN2/TFRC/TGFBR2/IL6/CEBPB/RARA | 33 |
| GO:0051222 | positive regulation of protein transport | 33/412 | 2.68E-10 | 3.74E-09 | PTGS2/CHRM1/ACHE/MAPK14/GSK3B/PPARD/IL4R/EGFR/MAPK1/TP53/HIF1A/ERBB2/GJA1/IL1B/IL2/IFNG/IL1A/CHUK/NPEPPS/JAK2/MMP8/F2R/FFAR1/GCK/ARF1/PAEP/IL6/ORM1/CLEC4E/NR1H4/CACNB3/GPER1/PLA2G1B | 33 |
| GO:0051047 | positive regulation of secretion | 33/412 | 4.92E-10 | 6.42E-09 | ACHE/MAPK14/PPARD/SLC6A4/IL4R/EGFR/HIF1A/GJA1/IL1B/IL2/IFNG/IL1A/CHUK/SPP1/CYP19A1/JAK2/MMP8/F2R/FFAR1/GCK/ARF1/PAEP/IL6/OPRK1/ORM1/CLEC4E/NR1H4/CACNA1D/CACNB2/TRPV1/GPER1/NPPB/PLA2G1B | 33 |
| GO:0001819 | positive regulation of cytokine production | 33/412 | 3.78E-09 | 3.68E-08 | PTGS2/MAPK14/RELA/IL4R/STAT1/HMOX1/CYP1B1/IL6ST/CASP8/SOD1/HIF1A/IL1B/HSPB1/IL2/SERPINE1/IFNG/IL1A/CHUK/CD40LG/IRF1/JAK2/MMP8/F2R/HPSE/PAEP/IL6/NFKB1/NFKB2/ORM1/CLEC4E/RORA/CEBPB/RARA | 33 |
| GO:0060078 | regulation of postsynaptic membrane potential | 32/412 | 1.33E-23 | 1.77E-21 | CHRM1/ADRB2/OPRM1/GABRA1/GSK3B/ADRB1/AKT1/PPP3CA/GLRA1/GLRA2/GABRA2/GABRA3/GABRA4/GABRA5/GABRA6/GABRG1/GABRG2/GABRG3/GABRB1/GABRB2/GABRB3/GABRD/GABRE/GRIN1/GRIN2A/GRIN2B/GRIN2C/GRIN2D/GRIN3A/CACNB3/TRPV1/PTK2B | 32 |
| GO:0006367 | transcription initiation from RNA polymerase II promoter | 32/412 | 1.80E-19 | 1.47E-17 | PGR/AR/RXRA/ESR2/ESR1/PPARG/PPARD/RXRB/BAX/CDK1/NR1I2/NR1I3/CCND1/CDKN1A/TP53/CCNB1/PPARA/RUNX2/E2F2/NR3C1/RORC/VDR/NR3C2/RORA/NR1H4/ESRRA/ESRRB/RARA/RARB/RARG/RXRG/ESRRG | 32 |
| GO:0009755 | hormone-mediated signaling pathway | 32/412 | 1.59E-16 | 7.91E-15 | PGR/AR/RXRA/NCOA1/ESR2/ESR1/PPARG/PPARD/RXRB/NR1I2/NR1I3/RB1/PARP1/PPARA/JAK2/NR3C1/HDAC6/HDAC1/RORC/VDR/NR3C2/RORA/NR1H4/CSNK2B/ESRRA/ESRRB/GPER1/RARA/RARB/RARG/RXRG/ESRRG | 32 |
| GO:0051402 | neuron apoptotic process | 32/412 | 2.62E-16 | 1.28E-14 | JUN/BCL2/BAX/CASP3/HMOX1/BCL2L1/CASP9/RB1/TP53/SOD1/HIF1A/HSPA5/CCL2/NQO1/PARP1/ERBB3/RASA1/JAK2/NQO2/F2R/BACE1/MCL1/GRM4/GABRA5/POLB/GABRB2/GABRB3/GRIN1/SIGMAR1/CEBPB/PTK2B/G6PD | 32 |
| GO:0031668 | cellular response to extracellular stimulus | 32/412 | 7.30E-15 | 2.89E-13 | PTGS2/NCOA1/PPARG/JUN/BCL2/MAPK8/HMOX1/ICAM1/VCAM1/GSTP1/AKR1C3/FOS/CDKN1A/MAPK1/TP53/SOD1/HSPA5/COL1A1/NFE2L2/HSF1/HSPA8/GBA/FOLR1/FOLR2/SRD5A1/COMT/VDR/NR1H4/CACNB3/TRPV1/PIM1/CYP27B1 | 32 |
| GO:1903522 | regulation of blood circulation | 32/412 | 1.33E-13 | 3.73E-12 | PTGS2/CHRM3/SCN5A/ADRA1A/CHRM2/ADRA1B/ADRB1/ADRA2C/KCNH2/AKT1/ICAM1/GSTM2/EGFR/CAV1/GJA1/NOS3/IL2/JAK2/ATP2A1/ADA/F2R/ITPR1/CACNA1C/CACNA1D/CACNA1F/CACNA1S/CACNB1/CACNB2/CACNB3/CACNB4/TRPV1/PIK3CG | 32 |
| GO:0034612 | response to tumor necrosis factor | 32/412 | 5.17E-13 | 1.25E-11 | PTGS2/MAPK14/RELA/IKBKB/AKT1/CASP3/STAT1/ICAM1/SELE/VCAM1/HAS2/GSTP1/PSMD3/SLC2A4/MAPK1/NFKBIA/CASP8/CCL2/CXCL8/COL1A1/NFE2L2/CHUK/CD40LG/JAK2/GBA/PTPN2/NFKB1/RORA/NR1H4/TRPV1/GPER1/PTK2B | 32 |
| GO:0051098 | regulation of binding | 32/412 | 6.06E-11 | 9.83E-10 | ADRB2/PPARG/GSK3B/MET/JUN/AKT1/BCL2/BAX/MAPK8/HMOX1/PPP3CA/SLPI/MMP9/EGF/RB1/NFKBIA/HSPA5/CAV1/IFNG/PARP1/PPARA/HSF1/E2F1/PON1/JAK2/MMP8/HDAC8/LGALS3/ACTB/CSNK2B/RUVBL2/RARA | 32 |
| GO:0010876 | lipid localization | 32/412 | 3.61E-10 | 4.91E-09 | RXRA/NCOA2/NCOA1/NOS2/PPARG/PPARD/AKT1/EGF/NFKBIA/ACACA/CAV1/IL1B/PPARA/SPP1/PON1/CYP19A1/NPC1L1/ABCB1/NMUR2/PTPN2/MTTP/SOAT1/SOAT2/PLA2G2E/AKR1C1/IL6/NFKB1/SIGMAR1/NR1H4/CES1/FABP6/PLA2G1B | 32 |
| GO:0050673 | epithelial cell proliferation | 32/412 | 2.74E-09 | 2.80E-08 | PGR/AR/SCN5A/ESR1/PPARG/PPARD/KDR/JUN/AKT1/BAX/STAT1/HMOX1/HAS2/EGFR/VEGFA/CCND1/MAPK1/RB1/PRKCA/HIF1A/ERBB2/CAV1/MYC/F3/GJA1/CCL2/IGFBP3/FGF1/FGF2/VDR/CDK6/CEBPB | 32 |
| GO:0034614 | cellular response to reactive oxygen species | 31/412 | 5.74E-20 | 5.06E-18 | CDK2/CCNA2/RELA/NCF1/MET/DPEP1/JUN/AKT1/MAPK8/CDK1/CYP1B1/AKR1C3/MMP3/EGFR/FOS/MMP2/MMP9/MAPK1/SOD1/NOS3/MPO/NFE2L2/NQO1/HSF1/CHUK/DHFR/HDAC6/KDM6B/FXN/IL6/SIGMAR1 | 31 |
| GO:0043401 | steroid hormone mediated signaling pathway | 31/412 | 9.18E-19 | 6.84E-17 | PGR/AR/RXRA/NCOA1/ESR2/ESR1/PPARG/PPARD/RXRB/NR1I2/NR1I3/RB1/PARP1/PPARA/JAK2/NR3C1/HDAC6/HDAC1/RORC/VDR/NR3C2/RORA/NR1H4/ESRRA/ESRRB/GPER1/RARA/RARB/RARG/RXRG/ESRRG | 31 |
| GO:0097237 | cellular response to toxic substance | 31/412 | 4.87E-15 | 1.96E-13 | PTGS2/PTGS1/RELA/OPRD1/KDR/MET/CDK1/HMOX1/CYP1B1/GSTP1/GSTM1/GSTM2/SOD1/DUOX2/NOS3/MPO/ABCG2/NFE2L2/NQO1/HSF1/DHFR/HDAC6/GLRA1/GLRA2/KDM6B/FXN/HBA1/IL6/NFKB1/SIGMAR1/ACTB | 31 |
| GO:0018209 | peptidyl-serine modification | 31/412 | 6.40E-12 | 1.22E-10 | PTGS2/CDK2/MAPK14/GSK3B/DRD1/OPRD1/IKBKB/AKT1/BCL2/BAX/MAPK8/CDK1/EGFR/VEGFA/MAPK1/TOP1/RAF1/PRKCA/CAV1/PRKCB/CCNB1/IFNG/PARP1/CHEK2/CHUK/RPS6KA5/HDAC6/HSP90AA1/TGFBR2/IL6/CSNK2A1 | 31 |
| GO:0006631 | fatty acid metabolic process | 31/412 | 5.17E-10 | 6.68E-09 | PTGS2/PTGS1/PPARG/MAPK14/PPARD/AKT1/CYP3A4/CYP1A2/CYP1A1/CYP1B1/ALOX5/GSTP1/GSTM2/AKR1C3/EIF6/POR/ACACA/CAV1/IL1B/PPARA/PON1/CYP2C19/TBXAS1/PTGES/CES2/HPGDS/AKR1C2/CBR1/HIBCH/CES1/PLA2G1B | 31 |
| GO:1903532 | positive regulation of secretion by cell | 31/412 | 1.40E-09 | 1.60E-08 | ACHE/MAPK14/PPARD/SLC6A4/IL4R/EGFR/HIF1A/GJA1/IL1B/IL2/IFNG/IL1A/CHUK/SPP1/CYP19A1/JAK2/MMP8/F2R/FFAR1/GCK/ARF1/PAEP/IL6/OPRK1/ORM1/CLEC4E/NR1H4/CACNA1D/CACNB2/GPER1/PLA2G1B | 31 |
| GO:0045785 | positive regulation of cell adhesion | 31/412 | 1.78E-09 | 1.94E-08 | DPP4/GSK3B/RELA/KDR/IL4R/AKT1/ICAM1/VCAM1/HAS2/PPP3CA/VEGFA/IL6ST/PRKCA/ERBB2/CAV1/IL1B/CCL2/IL2/IFNG/IGF2/CD40LG/JAK3/JAK2/VAV1/ADA/TFRC/TGFBR2/IL6/CDK6/PTK2B/RARA | 31 |
| GO:1901342 | regulation of vasculature development | 31/412 | 5.35E-09 | 5.05E-08 | PTGS2/PPARG/KDR/STAT1/HMOX1/CYP1B1/VEGFA/PRKCA/HIF1A/ERBB2/F3/IL1B/CXCL8/PRKCB/NOS3/HSPB1/SERPINE1/IL1A/NFE2L2/CXCL10/E2F2/HK2/JAK1/FGF1/FGF2/EGLN1/TGFBR2/IL6/GPER1/PTK2B/NPPB | 31 |
| GO:0046394 | carboxylic acid biosynthetic process | 31/412 | 4.36E-08 | 3.33E-07 | PTGS2/PTGS1/CYP3A4/CYP1A2/CYP1A1/ALOX5/HAS2/INSR/AKR1C3/EIF6/EGF/TP53/HIF1A/ACACA/IL1B/IFNG/PPARA/HK2/DHFR/FPGS/OGT/GCK/TBXAS1/PTGES/HPGDS/NFKB1/NR1H4/AKR1D1/CBR1/ESRRB/PLA2G1B | 31 |
| GO:0016053 | organic acid biosynthetic process | 31/412 | 4.58E-08 | 3.45E-07 | PTGS2/PTGS1/CYP3A4/CYP1A2/CYP1A1/ALOX5/HAS2/INSR/AKR1C3/EIF6/EGF/TP53/HIF1A/ACACA/IL1B/IFNG/PPARA/HK2/DHFR/FPGS/OGT/GCK/TBXAS1/PTGES/HPGDS/NFKB1/NR1H4/AKR1D1/CBR1/ESRRB/PLA2G1B | 31 |
| GO:0015711 | organic anion transport | 31/412 | 1.13E-07 | 7.52E-07 | RXRA/NCOA2/NCOA1/NOS2/PPARG/PPARD/AKT1/ACACA/GJA1/IL1B/PPARA/ABCB1/CA2/CA1/CA7/CA12/CA14/CA9/FOLR1/FOLR2/SLC46A1/SLC22A12/NMUR2/CA4/MTTP/HBA1/PLA2G2E/AKR1C1/NR1H4/TRPV1/PLA2G1B | 31 |
| GO:0051249 | regulation of lymphocyte activation | 31/412 | 1.30E-07 | 8.50E-07 | DPP4/IGHG1/IL4R/AKT1/BCL2/CASP3/VCAM1/AHR/CDKN1A/IL6ST/SOD1/ERBB2/CAV1/IL1B/CCL2/IL2/IFNG/IGF2/CD40LG/IRF1/JAK3/VAV1/ADA/LGALS3/PTPN2/TFRC/TGFBR2/IL6/IGHG2/CEBPB/RARA | 31 |
| GO:0071241 | cellular response to inorganic substance | 30/412 | 9.52E-16 | 4.23E-14 | PTGS2/SCN5A/CDK2/CCNA2/NCF1/DPEP1/JUN/AKT1/MAPK8/HMOX1/CYP1A2/CYP1A1/AKR1C3/MMP3/EGFR/FOS/MMP9/MAPK1/SOD1/HSPA5/CCNB1/NFE2L2/NQO1/PARP1/HSF1/CHUK/GLRA1/GLRA2/BACE1/TF | 30 |
| GO:0071216 | cellular response to biotic stimulus | 30/412 | 9.61E-15 | 3.74E-13 | NOS2/MAPK14/GSK3B/PPARD/RELA/AKT1/MAPK8/ICAM1/GSTP1/MAPK1/TP53/NFKBIA/PRKCA/HSPA5/IL1B/CCL2/CXCL8/NOS3/SERPINE1/CXCL11/CXCL2/HSF1/CXCL10/IL6/NFKB1/OPRK1/NR1H4/CEBPB/HCK/RARA | 30 |
| GO:0033002 | muscle cell proliferation | 30/412 | 1.36E-14 | 5.05E-13 | PTGS2/RXRA/PPARG/MAPK14/PPARD/AKR1B1/JUN/AKT1/STAT1/CDK1/HMOX1/GSTP1/EGFR/CDKN1A/MMP2/MMP9/MAPK1/GJA1/CCNB1/IFNG/IGFBP3/JAK2/NQO2/HMGCR/FGF2/COMT/TGFBR2/IL6/GPER1/PIM1 | 30 |
| GO:1901617 | organic hydroxy compound biosynthetic process | 30/412 | 3.89E-13 | 9.57E-12 | AKR1B1/SLC6A3/CYP3A4/AKR1C3/TP53/POR/SOD1/ACACA/IL1B/IFNG/CYP19A1/NPC1L1/TYR/GBA/DHFR/HMGCR/FGF1/FGF2/SRD5A2/VDR/ISYNA1/NFKB1/LSS/NR1H4/AKR1D1/GPER1/PTK2B/CES1/G6PD/CYP27B1 | 30 |
| GO:0097193 | intrinsic apoptotic signaling pathway | 30/412 | 2.08E-12 | 4.49E-11 | PTGS2/AKT1/BCL2/BAX/CASP3/HMOX1/CYP1B1/BCL2L1/CDKN1A/CASP9/MMP9/TP53/SOD1/HIF1A/CAV1/HSPB1/NFE2L2/PARP1/CHEK2/E2F1/E2F2/PTPN1/JAK2/ATP2A1/HDAC1/MCL1/PTPN2/ITPR1/POLB/CEBPB | 30 |
| GO:0018105 | peptidyl-serine phosphorylation | 30/412 | 4.97E-12 | 9.83E-11 | PTGS2/CDK2/MAPK14/GSK3B/DRD1/OPRD1/IKBKB/AKT1/BCL2/BAX/MAPK8/CDK1/EGFR/VEGFA/MAPK1/TOP1/RAF1/PRKCA/CAV1/PRKCB/CCNB1/IFNG/CHEK2/CHUK/RPS6KA5/HDAC6/HSP90AA1/TGFBR2/IL6/CSNK2A1 | 30 |
| GO:0071375 | cellular response to peptide hormone stimulus | 30/412 | 2.98E-11 | 5.25E-10 | PPARG/GSK3B/CCNA2/RELA/AKT1/STAT1/GSTP1/SLC2A4/INSR/POR/CAV1/IL1B/PRKCB/NFE2L2/PARP1/HSF1/IGF2/PTPN1/JAK3/JAK2/CA2/PTPN2/OGT/SRD5A1/GCK/NFKB1/NR1H4/ESRRA/GPER1/PLA2G1B | 30 |
| GO:0032103 | positive regulation of response to external stimulus | 30/412 | 3.48E-11 | 6.08E-10 | PTGS2/OPRM1/MAPK14/F7/KDR/MET/EGFR/VEGFA/IL6ST/NFKBIA/PRKCA/F3/IL1B/PTGER3/CXCL8/HSPB1/IL2/THBD/SERPINE1/CXCL10/JAK2/MMP8/CCR1/FGF2/CTSC/IL6/CEBPB/PIK3CG/PTK2B/CYP27B1 | 30 |
| GO:0071214 | cellular response to abiotic stimulus | 30/412 | 6.40E-11 | 1.02E-09 | PTGS2/CHEK1/MAPK14/AKR1B1/AKT1/BAX/CASP3/MAPK8/SLC2A4/EGFR/BCL2L1/CDKN1A/CASP9/TP53/ELK1/CASP8/HSPA5/MYC/GJA1/IL1B/COL1A1/PARP1/CHEK2/HSF1/IRF1/NOX4/NFKB1/TRPV1/RUVBL2/RHO | 30 |
| GO:0104004 | cellular response to environmental stimulus | 30/412 | 6.40E-11 | 1.02E-09 | PTGS2/CHEK1/MAPK14/AKR1B1/AKT1/BAX/CASP3/MAPK8/SLC2A4/EGFR/BCL2L1/CDKN1A/CASP9/TP53/ELK1/CASP8/HSPA5/MYC/GJA1/IL1B/COL1A1/PARP1/CHEK2/HSF1/IRF1/NOX4/NFKB1/TRPV1/RUVBL2/RHO | 30 |
| GO:0007159 | leukocyte cell-cell adhesion | 30/412 | 9.96E-11 | 1.55E-09 | DPP4/RELA/IL4R/AKT1/CASP3/ICAM1/SELE/VCAM1/HAS2/IL6ST/ERBB2/CAV1/IL1B/CCL2/IL2/IFNG/PPARA/IGF2/CD40LG/IRF1/JAK3/VAV1/ADA/LGALS3/PTPN2/TFRC/TGFBR2/IL6/CEBPB/RARA | 30 |
| GO:0006869 | lipid transport | 30/412 | 6.89E-10 | 8.63E-09 | RXRA/NCOA2/NCOA1/NOS2/PPARG/PPARD/AKT1/EGF/NFKBIA/ACACA/CAV1/IL1B/PPARA/SPP1/PON1/CYP19A1/NPC1L1/ABCB1/NMUR2/MTTP/SOAT1/SOAT2/PLA2G2E/AKR1C1/NFKB1/SIGMAR1/NR1H4/CES1/FABP6/PLA2G1B | 30 |
| GO:0009123 | nucleoside monophosphate metabolic process | 30/412 | 1.31E-09 | 1.50E-08 | ATP5F1B/CDK1/INSR/EIF6/TP53/HIF1A/CCNB1/IFNG/PARP1/PPARA/HK2/ADK/HSPA8/ADA/TYMS/TK1/OGT/GCK/FXN/ESRRB/COX4I1/COX5A/COX5B/COX6A2/COX6B1/COX6C/COX7A1/COX7B/COX7C/COX8A | 30 |
| GO:0045765 | regulation of angiogenesis | 30/412 | 2.15E-09 | 2.26E-08 | PTGS2/PPARG/KDR/STAT1/HMOX1/CYP1B1/VEGFA/PRKCA/HIF1A/ERBB2/F3/IL1B/CXCL8/PRKCB/NOS3/HSPB1/SERPINE1/IL1A/NFE2L2/CXCL10/E2F2/HK2/JAK1/FGF1/FGF2/EGLN1/TGFBR2/IL6/PTK2B/NPPB | 30 |
| GO:0052547 | regulation of peptidase activity | 30/412 | 9.21E-08 | 6.27E-07 | PTGS2/PPARG/MAPK14/DPEP1/AKT1/BAX/SLPI/VEGFA/CASP9/MMP9/POR/CASP8/RAF1/CAV1/MYC/F3/BIRC5/SERPINE1/HSF1/CTSD/PCOLCE/JAK2/SERPINA6/F2R/HDAC1/GRIN1/GRIN2A/GRIN2B/CSNK2A1/GPER1 | 30 |
| GO:0050900 | leukocyte migration | 30/412 | 7.61E-07 | 4.13E-06 | MAPK14/F7/OLR1/AKT1/MMP1/HMOX1/ICAM1/SELE/VCAM1/VEGFA/CAV1/IL1B/CCL2/CXCL8/THBD/SERPINE1/COL1A1/CXCL11/CXCL2/CXCL10/CYP19A1/VAV1/ADA/CCR1/LGALS3/IL6/HCK/PIK3CG/PTK2B/PLA2G1B | 30 |
| GO:0006694 | steroid biosynthetic process | 29/412 | 4.61E-16 | 2.20E-14 | AKR1B1/HSD3B2/HSD3B1/CYP3A4/AKR1C3/POR/SOD1/ACACA/IL1B/IFNG/CYP19A1/NPC1L1/HSD11B1/HMGCR/CYP17A1/FGF1/SRD5A1/SRD5A2/VDR/HSD11B2/HSD17B1/NFKB1/LSS/NR1H4/AKR1D1/HSD17B11/CES1/G6PD/CYP27B1 | 29 |
| GO:0043523 | regulation of neuron apoptotic process | 29/412 | 3.00E-15 | 1.26E-13 | JUN/BCL2/BAX/CASP3/HMOX1/BCL2L1/CASP9/TP53/SOD1/HIF1A/CCL2/NQO1/PARP1/ERBB3/RASA1/JAK2/NQO2/F2R/BACE1/MCL1/GRM4/GABRA5/GABRB2/GABRB3/GRIN1/SIGMAR1/CEBPB/PTK2B/G6PD | 29 |
| GO:0044706 | multi-multicellular organism process | 29/412 | 1.32E-14 | 5.05E-13 | PTGS2/PGR/AR/RXRA/ESR1/PPARD/AKR1B1/ADRA2C/SLC6A4/AKT1/BCL2/CYP1A1/FOS/MMP2/MMP9/MAPK1/SOD1/GJA1/IL1B/THBD/CLDN4/HSF1/SPP1/COMT/VDR/TGFBR2/HSD11B2/RARA/CYP27B1 | 29 |
| GO:0031669 | cellular response to nutrient levels | 29/412 | 7.38E-14 | 2.22E-12 | PTGS2/NCOA1/PPARG/JUN/BCL2/MAPK8/HMOX1/ICAM1/AKR1C3/CDKN1A/MAPK1/TP53/SOD1/HSPA5/COL1A1/NFE2L2/HSF1/HSPA8/GBA/FOLR1/FOLR2/SRD5A1/COMT/VDR/NR1H4/CACNB3/TRPV1/PIM1/CYP27B1 | 29 |
| GO:0071356 | cellular response to tumor necrosis factor | 29/412 | 1.35E-11 | 2.47E-10 | MAPK14/RELA/IKBKB/AKT1/STAT1/ICAM1/VCAM1/HAS2/GSTP1/PSMD3/SLC2A4/MAPK1/NFKBIA/CASP8/CCL2/CXCL8/COL1A1/NFE2L2/CHUK/CD40LG/JAK2/GBA/PTPN2/NFKB1/RORA/NR1H4/TRPV1/GPER1/PTK2B | 29 |
| GO:1903037 | regulation of leukocyte cell-cell adhesion | 29/412 | 3.92E-11 | 6.66E-10 | DPP4/RELA/IL4R/AKT1/CASP3/ICAM1/VCAM1/HAS2/IL6ST/ERBB2/CAV1/IL1B/CCL2/IL2/IFNG/PPARA/IGF2/CD40LG/IRF1/JAK3/VAV1/ADA/LGALS3/PTPN2/TFRC/TGFBR2/IL6/CEBPB/RARA | 29 |
| GO:0006936 | muscle contraction | 29/412 | 2.12E-09 | 2.24E-08 | PTGS2/CHRM3/SCN5A/ADRA1A/CHRM2/ADRB2/ADRA1B/ADRA2C/DRD1/KCNH2/GSTM2/SOD1/CAV1/GJA1/PTGER3/CHUK/ATP2A1/ADA/F2R/GLRA1/NMUR2/CACNA1C/CACNA1D/CACNA1S/CACNB2/TRPV1/GPER1/PIK3CG/MB | 29 |
| GO:0009141 | nucleoside triphosphate metabolic process | 29/412 | 2.41E-09 | 2.51E-08 | ATP5F1B/CDK1/INSR/EIF6/TP53/HIF1A/CCNB1/IFNG/PARP1/PPARA/HK2/ADK/HSPA8/ADA/TYMS/OGT/GCK/FXN/ESRRB/COX4I1/COX5A/COX5B/COX6A2/COX6B1/COX6C/COX7A1/COX7B/COX7C/COX8A | 29 |
| GO:0043062 | extracellular structure organization | 29/412 | 7.23E-08 | 5.10E-07 | DPP4/PRSS1/KDR/MMP1/ICAM1/VCAM1/CYP1B1/HAS2/MMP3/MMP2/MMP9/RB1/SERPINE1/COL1A1/MPO/COL3A1/SPP1/MMP8/FGF2/CTSK/CTSS/MTTP/SOAT1/SOAT2/ARF1/PLA2G2E/IL6/NFKB2/CTRB1 | 29 |
| GO:0016049 | cell growth | 29/412 | 1.25E-06 | 6.37E-06 | ADRA1A/ESR2/PPARG/GSK3B/PPARD/AKT1/BCL2/EGFR/VEGFA/CDKN1A/RB1/TP53/ERBB2/GJA1/IL2/PPARA/SPP1/IGFBP3/HDAC6/HSP90AA1/CDA/FXN/TGFBR2/CSNK2A1/PTK2B/RARG/NPPB/G6PD/CYP27B1 | 29 |
| GO:0009612 | response to mechanical stimulus | 28/412 | 2.26E-14 | 7.63E-13 | PTGS2/CHEK1/PPARG/MAPK14/RELA/JUN/AKT1/MAPK8/STAT1/EGFR/FOS/NFKBIA/CASP8/RAF1/GJA1/IL1B/CCNB1/COL1A1/MPO/COL3A1/CXCL10/IRF1/BACE1/TRPA1/TGFBR2/NFKB1/CACNB3/PTK2B | 28 |
| GO:0015980 | energy derivation by oxidation of organic compounds | 28/412 | 4.30E-11 | 7.25E-10 | NOS2/GSK3B/PYGM/AKT1/CDK1/CYP1A2/INSR/IL6ST/TP53/HIF1A/MYC/CCNB1/IFNG/IGF2/ADRB3/CISD1/GCK/FXN/ESRRB/COX4I1/COX5A/COX5B/COX6A2/COX6B1/COX6C/COX7B/COX7C/COX8A | 28 |
| GO:0009416 | response to light stimulus | 28/412 | 4.11E-10 | 5.48E-09 | PTGS2/CHEK1/RELA/DRD1/AKT1/BCL2/BAX/CASP3/MAPK8/EGFR/CCND1/FOS/CDKN1A/CASP9/TP53/ELK1/HIF1A/MYC/PARP1/TYR/HMGCR/FEN1/GRIN1/GRIN2A/CACNA1F/RUVBL2/RHO/FECH | 28 |
| GO:0051235 | maintenance of location | 28/412 | 1.27E-09 | 1.46E-08 | PPARG/PPARD/DRD1/AKT1/BAX/GSTM2/NFKBIA/HSPA5/CAV1/IL1B/CXCL11/PPARA/CXCL10/HK2/ATP2A1/F2R/FGF2/PTPN2/TRPA1/SOAT1/FTH1/ITPR1/IL6/NFKB1/CACNA1C/TRPV1/GPER1/PTK2B | 28 |
| GO:0071902 | positive regulation of protein serine/threonine kinase activity | 28/412 | 1.67E-09 | 1.84E-08 | ADRB2/MAPK14/AKT1/CDK1/INSR/EGFR/VEGFA/CCND1/MAPK1/EGF/RAF1/SOD1/ERBB2/IL1B/CCNB1/IFNG/IGF2/CD40LG/PTPN1/JAK2/F2R/FGF1/FGF2/NOX4/GRM4/PIK3CG/PTK2B/PLA2G1B | 28 |
| GO:0007596 | blood coagulation | 28/412 | 1.91E-09 | 2.04E-08 | F7/ADRA2C/PLAU/MAPK1/RAF1/PRKCA/CAV1/F3/PRKCB/NOS3/HSPB1/PLAT/THBD/SERPINE1/COL1A1/NFE2L2/COL3A1/CD40LG/IRF1/JAK2/VAV1/F2R/HDAC1/HPSE/ITPR1/IL6/ACTB/PIK3CG | 28 |
| GO:0009167 | purine ribonucleoside monophosphate metabolic process | 28/412 | 2.48E-09 | 2.57E-08 | ATP5F1B/CDK1/INSR/EIF6/TP53/HIF1A/CCNB1/IFNG/PARP1/PPARA/HK2/ADK/HSPA8/ADA/OGT/GCK/FXN/ESRRB/COX4I1/COX5A/COX5B/COX6A2/COX6B1/COX6C/COX7A1/COX7B/COX7C/COX8A | 28 |
| GO:0007599 | hemostasis | 28/412 | 2.65E-09 | 2.72E-08 | F7/ADRA2C/PLAU/MAPK1/RAF1/PRKCA/CAV1/F3/PRKCB/NOS3/HSPB1/PLAT/THBD/SERPINE1/COL1A1/NFE2L2/COL3A1/CD40LG/IRF1/JAK2/VAV1/F2R/HDAC1/HPSE/ITPR1/IL6/ACTB/PIK3CG | 28 |
| GO:0009126 | purine nucleoside monophosphate metabolic process | 28/412 | 2.65E-09 | 2.72E-08 | ATP5F1B/CDK1/INSR/EIF6/TP53/HIF1A/CCNB1/IFNG/PARP1/PPARA/HK2/ADK/HSPA8/ADA/OGT/GCK/FXN/ESRRB/COX4I1/COX5A/COX5B/COX6A2/COX6B1/COX6C/COX7A1/COX7B/COX7C/COX8A | 28 |
| GO:0009144 | purine nucleoside triphosphate metabolic process | 28/412 | 2.83E-09 | 2.87E-08 | ATP5F1B/CDK1/INSR/EIF6/TP53/HIF1A/CCNB1/IFNG/PARP1/PPARA/HK2/ADK/HSPA8/ADA/OGT/GCK/FXN/ESRRB/COX4I1/COX5A/COX5B/COX6A2/COX6B1/COX6C/COX7A1/COX7B/COX7C/COX8A | 28 |
| GO:0050817 | coagulation | 28/412 | 2.83E-09 | 2.87E-08 | F7/ADRA2C/PLAU/MAPK1/RAF1/PRKCA/CAV1/F3/PRKCB/NOS3/HSPB1/PLAT/THBD/SERPINE1/COL1A1/NFE2L2/COL3A1/CD40LG/IRF1/JAK2/VAV1/F2R/HDAC1/HPSE/ITPR1/IL6/ACTB/PIK3CG | 28 |
| GO:0048638 | regulation of developmental growth | 28/412 | 3.90E-09 | 3.79E-08 | AR/ADRB2/MAPK14/GSK3B/PPARD/ADRB1/SLC6A3/SLC6A4/AKT1/BCL2/CDK1/INSR/VEGFA/CDKN1A/MAPK1/POR/SOD1/GJA1/CCNB1/PPARA/HSF1/SPP1/IGF2/FGF2/FXN/TGFBR2/PIM1/G6PD | 28 |
| GO:0009161 | ribonucleoside monophosphate metabolic process | 28/412 | 6.07E-09 | 5.65E-08 | ATP5F1B/CDK1/INSR/EIF6/TP53/HIF1A/CCNB1/IFNG/PARP1/PPARA/HK2/ADK/HSPA8/ADA/OGT/GCK/FXN/ESRRB/COX4I1/COX5A/COX5B/COX6A2/COX6B1/COX6C/COX7A1/COX7B/COX7C/COX8A | 28 |
| GO:0018108 | peptidyl-tyrosine phosphorylation | 28/412 | 1.05E-08 | 9.36E-08 | ADRA1A/NCF1/KDR/MET/ICAM1/INSR/EGFR/VEGFA/EGF/IL6ST/TP53/ERBB2/CAV1/IL2/IFNG/HSF1/IGF2/ERBB3/PTPN1/NEK1/JAK3/JAK1/JAK2/NOX4/PTPN2/IL6/HCK/PTK2B | 28 |
| GO:0018212 | peptidyl-tyrosine modification | 28/412 | 1.26E-08 | 1.11E-07 | ADRA1A/NCF1/KDR/MET/ICAM1/INSR/EGFR/VEGFA/EGF/IL6ST/TP53/ERBB2/CAV1/IL2/IFNG/HSF1/IGF2/ERBB3/PTPN1/NEK1/JAK3/JAK1/JAK2/NOX4/PTPN2/IL6/HCK/PTK2B | 28 |
| GO:0050678 | regulation of epithelial cell proliferation | 28/412 | 2.52E-08 | 2.07E-07 | PGR/AR/SCN5A/PPARG/PPARD/KDR/JUN/AKT1/BAX/STAT1/HMOX1/HAS2/EGFR/VEGFA/CCND1/RB1/PRKCA/HIF1A/ERBB2/CAV1/MYC/F3/GJA1/CCL2/FGF1/FGF2/VDR/CDK6 | 28 |
| GO:0016570 | histone modification | 28/412 | 1.09E-06 | 5.70E-06 | NCOA1/CDK2/CHEK1/CCNA2/DRD1/MAPK8/CDK1/VEGFA/TP53/PRKCA/IL1B/PRKCB/CCNB1/IGF2/RPS6KA5/JAK2/HDAC6/HDAC8/HDAC1/KDM4E/KDM2A/KDM6B/PHF8/KDM5C/OGT/NR1H4/KANSL3/RUVBL2 | 28 |
| GO:0001667 | ameboidal-type cell migration | 28/412 | 1.47E-06 | 7.27E-06 | PTGS2/DPP4/PPARG/PPARD/KDR/MET/JUN/ATP5F1B/AKT1/HMOX1/CYP1B1/HAS2/VEGFA/MMP9/PRKCA/HIF1A/NOS3/HSPB1/IFNG/NFE2L2/FOLR1/HDAC6/FGF1/FGF2/LGALS8/TGFBR2/CSNK2B/PTK2B | 28 |
| GO:0016569 | covalent chromatin modification | 28/412 | 2.52E-06 | 1.16E-05 | NCOA1/CDK2/CHEK1/CCNA2/DRD1/MAPK8/CDK1/VEGFA/TP53/PRKCA/IL1B/PRKCB/CCNB1/IGF2/RPS6KA5/JAK2/HDAC6/HDAC8/HDAC1/KDM4E/KDM2A/KDM6B/PHF8/KDM5C/OGT/NR1H4/KANSL3/RUVBL2 | 28 |
| GO:0045471 | response to ethanol | 27/412 | 2.08E-19 | 1.64E-17 | OPRM1/MAOB/HTR3A/SLC6A3/CDK1/ICAM1/VCAM1/GSTP1/SLC2A4/CCND1/CASP8/SOD1/IL2/NQO1/TYMS/HMGCR/GLRA1/GLRA2/POLB/GRIN1/GRIN2A/GRIN2B/GRIN3A/OPRK1/PTK2B/RARA/G6PD | 27 |
| GO:0071248 | cellular response to metal ion | 27/412 | 1.35E-14 | 5.05E-13 | PTGS2/SCN5A/NCF1/DPEP1/JUN/AKT1/MAPK8/HMOX1/CYP1A2/CYP1A1/AKR1C3/EGFR/FOS/MMP9/MAPK1/SOD1/HSPA5/CCNB1/NFE2L2/NQO1/PARP1/HSF1/CHUK/GLRA1/GLRA2/BACE1/TF | 27 |
| GO:0007565 | female pregnancy | 27/412 | 1.76E-14 | 6.28E-13 | PTGS2/PGR/AR/RXRA/ESR1/PPARD/AKR1B1/ADRA2C/AKT1/BCL2/FOS/MMP2/MMP9/MAPK1/SOD1/GJA1/IL1B/THBD/CLDN4/HSF1/SPP1/COMT/VDR/TGFBR2/HSD11B2/RARA/CYP27B1 | 27 |
| GO:0071222 | cellular response to lipopolysaccharide | 27/412 | 9.03E-14 | 2.60E-12 | NOS2/MAPK14/PPARD/RELA/AKT1/MAPK8/ICAM1/GSTP1/MAPK1/NFKBIA/PRKCA/IL1B/CCL2/CXCL8/NOS3/SERPINE1/CXCL11/CXCL2/HSF1/CXCL10/IL6/NFKB1/OPRK1/NR1H4/CEBPB/HCK/RARA | 27 |
| GO:0071229 | cellular response to acid chemical | 27/412 | 1.45E-13 | 4.00E-12 | PPARG/SLC6A4/KDR/AKT1/AKR1C3/EGFR/VEGFA/BCL2L1/MMP2/ACACA/CCNB1/COL1A1/COL3A1/HSF1/E2F1/FOLR1/FOLR2/GLRA1/GLRA2/AKR1C1/AKR1C2/NR1H4/CEBPB/PTK2B/RARA/RARB/RARG | 27 |
| GO:0071219 | cellular response to molecule of bacterial origin | 27/412 | 2.06E-13 | 5.39E-12 | NOS2/MAPK14/PPARD/RELA/AKT1/MAPK8/ICAM1/GSTP1/MAPK1/NFKBIA/PRKCA/IL1B/CCL2/CXCL8/NOS3/SERPINE1/CXCL11/CXCL2/HSF1/CXCL10/IL6/NFKB1/OPRK1/NR1H4/CEBPB/HCK/RARA | 27 |
| GO:2001234 | negative regulation of apoptotic signaling pathway | 27/412 | 1.49E-12 | 3.26E-11 | PTGS2/AR/RELA/AKT1/BCL2/BAX/HMOX1/ICAM1/GSTP1/BCL2L1/MMP9/RB1/CASP8/RAF1/HIF1A/IL1B/NOS3/HSPB1/SERPINE1/IL1A/NFE2L2/PTPN1/HDAC1/MCL1/LGALS3/FXN/CSNK2A1 | 27 |
| GO:0009266 | response to temperature stimulus | 27/412 | 5.53E-12 | 1.07E-10 | PTGS2/ADRB2/HSP90AA2P/PPARG/GSK3B/ADRB1/AKT1/HMOX1/FOS/CDKN1A/MAPK1/NFKBIA/CASP8/SOD1/HSPA5/NOS3/IL1A/HSF1/CXCL10/HSPA8/FGF1/HSP90AA1/TRPA1/TRPM8/TRPV3/TRPV1/HSPA2 | 27 |
| GO:0032409 | regulation of transporter activity | 27/412 | 1.88E-10 | 2.81E-09 | ADRB2/OPRM1/GRIA2/PPARG/HTR3A/BCL2/GSTM2/MMP9/CAV1/GJA1/CCL2/IFNG/PON1/ATP2A1/ABCB1/CTSS/GRIN1/GRIN2A/GRIN2B/CACNA1D/CACNB1/CACNB2/CACNB3/CACNB4/ACTB/HSPA2/PTK2B | 27 |
| GO:0003015 | heart process | 27/412 | 3.25E-10 | 4.50E-09 | SCN5A/ADRA1A/CHRM2/ADRA1B/ADRB1/KCNH2/GSTM2/SOD1/CAV1/GJA1/NOS3/IL2/JAK2/ATP2A1/ADA/NOX4/ITPR1/CACNA1C/CACNA1D/CACNA1F/CACNA1S/CACNB1/CACNB2/CACNB3/CACNB4/TRPV1/PIK3CG | 27 |
| GO:0010631 | epithelial cell migration | 27/412 | 2.05E-08 | 1.74E-07 | PTGS2/DPP4/PPARG/PPARD/KDR/MET/JUN/ATP5F1B/AKT1/HMOX1/CYP1B1/HAS2/VEGFA/MMP9/PRKCA/HIF1A/NOS3/HSPB1/IFNG/NFE2L2/HDAC6/FGF1/FGF2/LGALS8/TGFBR2/CSNK2B/PTK2B | 27 |
| GO:0090132 | epithelium migration | 27/412 | 2.45E-08 | 2.03E-07 | PTGS2/DPP4/PPARG/PPARD/KDR/MET/JUN/ATP5F1B/AKT1/HMOX1/CYP1B1/HAS2/VEGFA/MMP9/PRKCA/HIF1A/NOS3/HSPB1/IFNG/NFE2L2/HDAC6/FGF1/FGF2/LGALS8/TGFBR2/CSNK2B/PTK2B | 27 |
| GO:0090130 | tissue migration | 27/412 | 3.48E-08 | 2.77E-07 | PTGS2/DPP4/PPARG/PPARD/KDR/MET/JUN/ATP5F1B/AKT1/HMOX1/CYP1B1/HAS2/VEGFA/MMP9/PRKCA/HIF1A/NOS3/HSPB1/IFNG/NFE2L2/HDAC6/FGF1/FGF2/LGALS8/TGFBR2/CSNK2B/PTK2B | 27 |
| GO:0045862 | positive regulation of proteolysis | 27/412 | 4.13E-08 | 3.17E-07 | PPARG/MAPK14/GSK3B/AKT1/BAX/CASP9/EGF/CASP8/CAV1/MYC/F3/IL1B/IFNG/NFE2L2/HSF1/CTSD/PCOLCE/JAK2/GBA/F2R/OGT/CTSC/GRIN1/GRIN2A/GRIN2B/GPER1/PTK2B | 27 |
| GO:0052548 | regulation of endopeptidase activity | 27/412 | 9.64E-07 | 5.08E-06 | PTGS2/PPARG/DPEP1/AKT1/BAX/SLPI/VEGFA/CASP9/MMP9/POR/CASP8/RAF1/MYC/F3/BIRC5/SERPINE1/HSF1/CTSD/JAK2/SERPINA6/F2R/HDAC1/GRIN1/GRIN2A/GRIN2B/CSNK2A1/GPER1 | 27 |
| GO:0034764 | positive regulation of transmembrane transport | 26/412 | 5.72E-13 | 1.37E-11 | ADRB2/MAPK14/HTR3A/DRD1/KCNH2/AKT1/BAX/INSR/GSTM2/CCL2/IFNG/NFE2L2/CXCL11/CXCL10/ATP2A1/ABCB1/CA2/F2R/CTSS/ARF1/OPRK1/CACNB2/CACNB3/GPER1/HSPA2/G6PD | 26 |
| GO:0097191 | extrinsic apoptotic signaling pathway | 26/412 | 5.07E-12 | 9.96E-11 | AR/GSK3B/RELA/AKT1/BCL2/BAX/CASP3/HMOX1/ICAM1/GSTP1/BCL2L1/CASP9/CASP8/RAF1/CAV1/IL1B/NOS3/IL2/SERPINE1/IFNG/IL1A/ERBB3/JAK2/MCL1/LGALS3/GPER1 | 26 |
| GO:0015850 | organic hydroxy compound transport | 26/412 | 1.74E-10 | 2.62E-09 | RXRA/NCOA2/NCOA1/SLC6A2/PPARG/MAOB/ADRA2C/DRD1/CHRM5/SLC6A3/SLC6A4/EGF/NFKBIA/CAV1/SPP1/PON1/CYP19A1/NPC1L1/SOAT1/SOAT2/AKR1C1/NFKB1/OPRK1/NR1H4/ACTB/CES1 | 26 |
| GO:0060047 | heart contraction | 26/412 | 7.39E-10 | 9.06E-09 | SCN5A/ADRA1A/CHRM2/ADRA1B/ADRB1/KCNH2/GSTM2/SOD1/CAV1/GJA1/NOS3/IL2/JAK2/ATP2A1/ADA/ITPR1/CACNA1C/CACNA1D/CACNA1F/CACNA1S/CACNB1/CACNB2/CACNB3/CACNB4/TRPV1/PIK3CG | 26 |
| GO:0046034 | ATP metabolic process | 26/412 | 4.55E-09 | 4.37E-08 | ATP5F1B/CDK1/INSR/EIF6/TP53/HIF1A/CCNB1/IFNG/PARP1/PPARA/HK2/HSPA8/OGT/GCK/FXN/ESRRB/COX4I1/COX5A/COX5B/COX6A2/COX6B1/COX6C/COX7A1/COX7B/COX7C/COX8A | 26 |
| GO:0050863 | regulation of T cell activation | 26/412 | 8.35E-09 | 7.58E-08 | DPP4/IL4R/AKT1/CASP3/VCAM1/IL6ST/SOD1/ERBB2/CAV1/IL1B/CCL2/IL2/IFNG/IGF2/CD40LG/IRF1/JAK3/VAV1/ADA/LGALS3/PTPN2/TFRC/TGFBR2/IL6/CEBPB/RARA | 26 |
| GO:0009205 | purine ribonucleoside triphosphate metabolic process | 26/412 | 3.15E-08 | 2.52E-07 | ATP5F1B/CDK1/INSR/EIF6/TP53/HIF1A/CCNB1/IFNG/PARP1/PPARA/HK2/HSPA8/OGT/GCK/FXN/ESRRB/COX4I1/COX5A/COX5B/COX6A2/COX6B1/COX6C/COX7A1/COX7B/COX7C/COX8A | 26 |
| GO:0043405 | regulation of MAP kinase activity | 26/412 | 3.55E-08 | 2.82E-07 | MAPK14/CDK1/GSTP1/INSR/EGFR/VEGFA/MAPK1/EGF/RAF1/SOD1/ERBB2/CAV1/IL1B/CD40LG/PTPN1/JAK2/GBA/F2R/HMGCR/FGF1/FGF2/NOX4/GRM4/PIK3CG/PTK2B/PLA2G1B | 26 |
| GO:0009199 | ribonucleoside triphosphate metabolic process | 26/412 | 4.50E-08 | 3.41E-07 | ATP5F1B/CDK1/INSR/EIF6/TP53/HIF1A/CCNB1/IFNG/PARP1/PPARA/HK2/HSPA8/OGT/GCK/FXN/ESRRB/COX4I1/COX5A/COX5B/COX6A2/COX6B1/COX6C/COX7A1/COX7B/COX7C/COX8A | 26 |
| GO:0072330 | monocarboxylic acid biosynthetic process | 26/412 | 5.06E-08 | 3.74E-07 | PTGS2/PTGS1/CYP3A4/CYP1A2/CYP1A1/ALOX5/INSR/AKR1C3/EIF6/TP53/HIF1A/ACACA/IL1B/IFNG/PPARA/HK2/OGT/GCK/TBXAS1/PTGES/HPGDS/NR1H4/AKR1D1/CBR1/ESRRB/PLA2G1B | 26 |
| GO:0045787 | positive regulation of cell cycle | 26/412 | 5.92E-07 | 3.30E-06 | CDK2/CHEK1/SLC6A4/AKT1/BAX/CDK1/CYP1A1/INSR/EGFR/CCND1/CDKN1A/EGF/RB1/TP53/PRKCA/IL1B/CCNB1/IL1A/CHEK2/HSF1/E2F1/IGF2/FEN1/GPER1/HSPA2/RARA | 26 |
| GO:0050867 | positive regulation of cell activation | 26/412 | 7.54E-07 | 4.10E-06 | DPP4/IGHG1/IL4R/AKT1/BCL2/VCAM1/CDKN1A/IL6ST/CAV1/IL1B/CCL2/IL2/IFNG/IGF2/CD40LG/JAK3/JAK2/VAV1/MMP8/ADA/CTSC/TFRC/TGFBR2/IL6/IGHG2/RARA | 26 |
| GO:0001503 | ossification | 26/412 | 9.12E-07 | 4.87E-06 | PTGS2/ACHE/ADRB2/MAPK14/ATP5F1B/AKT1/BCL2/EGFR/MMP2/MAPK1/IL6ST/HIF1A/GJA1/COL1A1/SPP1/RUNX2/IGFBP3/IGF2/CCR1/CTSK/IL6/CDK6/CEBPB/ESRRA/PTK2B/CYP27B1 | 26 |
| GO:0009896 | positive regulation of catabolic process | 26/412 | 2.82E-06 | 1.27E-05 | ADRB2/GSK3B/KDR/AKT1/BAX/HMOX1/INSR/EGF/HIF1A/CAV1/GJA1/IL1B/IFNG/NFE2L2/PPARA/HSF1/HK2/PTPN1/GBA/HDAC6/HSP90AA1/CTSC/IL6/CSNK2A1/ESRRB/PTK2B | 26 |
| GO:0032386 | regulation of intracellular transport | 26/412 | 2.82E-06 | 1.27E-05 | PTGS2/CHRM1/ADRA1A/CHRM2/MAPK14/GSK3B/DRD1/PKIA/IL4R/HMOX1/SLC2A4/MAPK1/TP53/ERBB2/IL1B/PRKCB/IFNG/NPEPPS/PTPN1/ATP2A1/BACE1/ARF1/GRIN3A/CACNA1D/CACNB2/CACNB3 | 26 |
| GO:0051052 | regulation of DNA metabolic process | 26/412 | 3.64E-06 | 1.57E-05 | CDK2/CHEK1/PPARG/CCNA2/JUN/AKT1/BAX/CDK1/EGFR/CDKN1A/MAPK1/TP53/MYC/GJA1/IL2/PARP1/CHEK2/HSF1/HDAC8/FGF2/HSP90AA1/NOX4/TFRC/IL6/GPER1/PTK2B | 26 |
| GO:0002446 | neutrophil mediated immunity | 26/412 | 5.02E-05 | 0.000146089 | MAPK14/OLR1/TNFAIP6/ALOX5/GSTP1/PSMD3/SLPI/PLAU/MMP9/MAPK1/MPO/CTSD/MMP8/HSPA8/HPSE/HSP90AA1/CTSS/LGALS3/CDA/CTSC/FTH1/IL6/NFKB1/ORM1/CSNK2B/PLA2G1B | 26 |
| GO:1903409 | reactive oxygen species biosynthetic process | 25/412 | 1.77E-17 | 1.04E-15 | PTGS2/NOS2/MAOB/AKT1/CYP1A2/CYP1A1/ICAM1/CYP1B1/INSR/SOD1/CAV1/IL1B/DUOX2/NOS3/IFNG/MPO/NQO1/JAK2/MMP8/HSP90AA1/NOX4/GRIN1/RORA/TRPV1/PTK2B | 25 |
| GO:0042133 | neurotransmitter metabolic process | 25/412 | 4.80E-15 | 1.96E-13 | PTGS2/ACHE/NOS2/MAOB/SLC6A3/SLC6A4/AKT1/ICAM1/CYP1B1/INSR/POR/CAV1/IL1B/NOS3/IFNG/NQO1/JAK2/MMP8/HSP90AA1/SRD5A1/COMT/GRIN2A/RORA/TRPV1/PTK2B | 25 |
| GO:0007623 | circadian rhythm | 25/412 | 6.05E-12 | 1.16E-10 | NCOA2/NOS2/PPARG/GSK3B/F7/SLC6A4/JUN/MAPK8/CDK1/AHR/EGFR/TP53/TOP1/SERPINE1/TOP2A/CLDN4/PPARA/ADA/TYMS/HDAC1/RORC/KDM2A/OGT/SRD5A1/RORA | 25 |
| GO:0048872 | homeostasis of number of cells | 25/412 | 2.37E-10 | 3.38E-09 | MAPK14/AKT1/BCL2/BAX/CASP3/STAT1/HMOX1/VEGFA/RB1/SOD1/HIF1A/NOS3/IL2/JAK3/JAK2/ADA/F2R/PTPN2/AHSP/POLB/IL6/CACNA1F/CDK6/MB/G6PD | 25 |
| GO:0008016 | regulation of heart contraction | 25/412 | 3.63E-10 | 4.91E-09 | SCN5A/ADRA1A/CHRM2/ADRA1B/ADRB1/KCNH2/GSTM2/CAV1/GJA1/NOS3/IL2/JAK2/ATP2A1/ADA/ITPR1/CACNA1C/CACNA1D/CACNA1F/CACNA1S/CACNB1/CACNB2/CACNB3/CACNB4/TRPV1/PIK3CG | 25 |
| GO:0007187 | G protein-coupled receptor signaling pathway, coupled to cyclic nucleotide second messenger | 25/412 | 5.51E-10 | 7.04E-09 | CHRM3/CHRM1/ADRA1A/CHRM2/ADRB2/OPRM1/ADRA1B/ADRB1/ADRA2C/ADRA1D/DRD1/CHRM5/CHRM4/OPRD1/PRKCA/CCL2/PTGER3/CXCL11/CXCL10/ADRB3/CCR1/GRM4/OPRK1/CACNA1D/GPER1 | 25 |
| GO:0090257 | regulation of muscle system process | 25/412 | 7.04E-10 | 8.73E-09 | PTGS2/CHRM3/SCN5A/ADRA1A/CHRM2/ADRB2/ADRA1B/ADRA2C/PPP3CA/GSTM2/IL6ST/SOD1/PRKCA/CAV1/NOS3/PARP1/PPARA/ATP2A1/ADA/F2R/NMUR2/CACNA1C/GPER1/PIK3CG/G6PD | 25 |
| GO:0022898 | regulation of transmembrane transporter activity | 25/412 | 1.44E-09 | 1.62E-08 | ADRB2/OPRM1/GRIA2/HTR3A/BCL2/GSTM2/MMP9/CAV1/GJA1/CCL2/IFNG/ATP2A1/ABCB1/CTSS/GRIN1/GRIN2A/GRIN2B/CACNA1D/CACNB1/CACNB2/CACNB3/CACNB4/ACTB/HSPA2/PTK2B | 25 |
| GO:0050714 | positive regulation of protein secretion | 25/412 | 1.44E-09 | 1.62E-08 | ACHE/MAPK14/PPARD/IL4R/EGFR/HIF1A/GJA1/IL1B/IL2/IFNG/IL1A/CHUK/JAK2/MMP8/F2R/FFAR1/GCK/ARF1/PAEP/IL6/ORM1/CLEC4E/NR1H4/GPER1/PLA2G1B | 25 |
| GO:0032868 | response to insulin | 25/412 | 1.95E-09 | 2.08E-08 | PPARG/GSK3B/RELA/AKT1/STAT1/ICAM1/GSTP1/SLC2A4/INSR/EIF6/IL1B/PRKCB/PARP1/PPARA/IGF2/PTPN1/PTPN2/OGT/SRD5A1/GCK/HSD11B2/OPRK1/NR1H4/ESRRA/PLA2G1B | 25 |
| GO:0002793 | positive regulation of peptide secretion | 25/412 | 6.29E-09 | 5.81E-08 | ACHE/MAPK14/PPARD/IL4R/EGFR/HIF1A/GJA1/IL1B/IL2/IFNG/IL1A/CHUK/JAK2/MMP8/F2R/FFAR1/GCK/ARF1/PAEP/IL6/ORM1/CLEC4E/NR1H4/GPER1/PLA2G1B | 25 |
| GO:0098656 | anion transmembrane transport | 25/412 | 6.29E-09 | 5.81E-08 | GABRA1/AKT1/ACACA/CLDN4/ABCB1/FOLR1/FOLR2/SLC46A1/GLRA1/GLRA2/GABRA2/GABRA3/GABRA4/GABRA5/GABRA6/GABRG1/GABRG2/GABRG3/GABRB1/GABRB2/GABRB3/GABRD/GABRE/GABRP/GABRQ | 25 |
| GO:0060326 | cell chemotaxis | 25/412 | 1.86E-08 | 1.59E-07 | MAPK14/F7/KDR/MET/VCAM1/GSTP1/VEGFA/IL1B/CCL2/CXCL8/HSPB1/SERPINE1/CXCL11/CXCL2/CXCL10/CYP19A1/VAV1/CCR1/FGF1/FGF2/LGALS3/IL6/PIK3CG/PTK2B/PLA2G1B | 25 |
| GO:0046879 | hormone secretion | 25/412 | 3.12E-08 | 2.50E-07 | DPP4/NOS2/PPARD/ADRA2C/PPP3CA/EGFR/RAF1/PRKCA/HIF1A/GJA1/IL1B/IFNG/SPP1/CYP19A1/JAK2/HMGCR/FFAR1/GCK/ITPR1/IL6/OPRK1/NR1H4/CACNA1C/CACNA1D/GPER1 | 25 |
| GO:0009914 | hormone transport | 25/412 | 5.78E-08 | 4.19E-07 | DPP4/NOS2/PPARD/ADRA2C/PPP3CA/EGFR/RAF1/PRKCA/HIF1A/GJA1/IL1B/IFNG/SPP1/CYP19A1/JAK2/HMGCR/FFAR1/GCK/ITPR1/IL6/OPRK1/NR1H4/CACNA1C/CACNA1D/GPER1 | 25 |
| GO:0002696 | positive regulation of leukocyte activation | 25/412 | 1.30E-06 | 6.62E-06 | DPP4/IGHG1/IL4R/AKT1/BCL2/VCAM1/CDKN1A/IL6ST/CAV1/IL1B/CCL2/IL2/IFNG/IGF2/CD40LG/JAK3/VAV1/MMP8/ADA/CTSC/TFRC/TGFBR2/IL6/IGHG2/RARA | 25 |
| GO:0060537 | muscle tissue development | 25/412 | 4.63E-06 | 1.91E-05 | RXRA/ADRA1A/MAPK14/BCL2/CDK1/PPP3CA/VEGFA/FOS/MAPK1/RB1/CAV1/GJA1/CCNB1/COL3A1/PPARA/ERBB3/HMGCR/FGF2/KDM6B/NOX4/TGFBR2/PIM1/RARA/RARB/G6PD | 25 |
| GO:0045088 | regulation of innate immune response | 25/412 | 2.65E-05 | 8.57E-05 | ESR1/PPARG/RELA/IKBKB/STAT1/PSMD3/NFKBIA/CASP8/RAF1/CAV1/IFNG/CHUK/IRF1/PTPN1/RPS6KA5/JAK1/JAK2/VAV1/CTSK/CTSS/PTPN2/NFKB1/CLEC4E/NR1H4/HCK | 25 |
| GO:0006914 | autophagy | 25/412 | 0.000118092 | 0.00031174 | ADRA1A/ADRB2/GSK3B/KDR/MET/AKT1/BCL2/CASP3/MAPK8/HMOX1/TP53/HIF1A/HSPB1/IFNG/HK2/HSPA8/GBA/HDAC6/HSP90AA1/MCL1/CTSK/LGALS8/ITPR1/CSNK2A1/CSNK2B | 25 |
| GO:0061919 | process utilizing autophagic mechanism | 25/412 | 0.000118092 | 0.00031174 | ADRA1A/ADRB2/GSK3B/KDR/MET/AKT1/BCL2/CASP3/MAPK8/HMOX1/TP53/HIF1A/HSPB1/IFNG/HK2/HSPA8/GBA/HDAC6/HSP90AA1/MCL1/CTSK/LGALS8/ITPR1/CSNK2A1/CSNK2B | 25 |
| GO:0042119 | neutrophil activation | 25/412 | 0.000125733 | 0.000329506 | MAPK14/OLR1/TNFAIP6/ALOX5/GSTP1/PSMD3/SLPI/PLAU/MMP9/MAPK1/CXCL8/MPO/CTSD/MMP8/HSPA8/HPSE/HSP90AA1/CTSS/LGALS3/CDA/CTSC/FTH1/NFKB1/ORM1/CSNK2B | 25 |
| GO:0034754 | cellular hormone metabolic process | 24/412 | 8.11E-16 | 3.73E-14 | ADH1B/ADH1C/ESR1/AKR1B1/HSD3B2/HSD3B1/CYP3A4/CYP1A2/CYP1A1/CYP1B1/AKR1C3/SULT1E1/SPP1/CYP19A1/CYP17A1/SRD5A1/SRD5A2/UGT2B7/COMT/AKR1C1/AKR1C2/HSD17B1/AKR1D1/HSD17B11 | 24 |
| GO:0042542 | response to hydrogen peroxide | 24/412 | 1.49E-14 | 5.46E-13 | RELA/MET/JUN/BCL2/CASP3/STAT1/CDK1/HMOX1/CYP1B1/SOD1/COL1A1/NFE2L2/NQO1/HSF1/ADA/HDAC6/KDM6B/TRPA1/FXN/HBA1/IL6/SIGMAR1/PTK2B/MB | 24 |
| GO:0062013 | positive regulation of small molecule metabolic process | 24/412 | 4.38E-14 | 1.39E-12 | PTGS2/NOS2/PPARG/AKT1/CDK1/HAS2/INSR/EGF/POR/HIF1A/IL1B/NOS3/CCNB1/IFNG/PPARA/IGF2/FGF1/PTPN2/GCK/COMT/NFKB1/NR1H4/ESRRB/GPER1 | 24 |
| GO:0003018 | vascular process in circulatory system | 24/412 | 7.02E-13 | 1.65E-11 | PTGS2/CHRM3/ADRA1A/ADRB2/ADRA1B/PPARD/ADRB1/ADRA2C/DRD1/SLC6A4/AKT1/HMOX1/ICAM1/EGFR/VEGFA/SOD1/CAV1/GJA1/NOS3/ADRB3/F2R/HMGCR/GPER1/NPPB | 24 |
| GO:0031099 | regeneration | 24/412 | 1.35E-11 | 2.47E-10 | PPARG/CCNA2/PPARD/F7/JUN/BCL2/CDK1/HMOX1/GSTP1/PPP3CA/EGFR/CCND1/CDKN1A/GJA1/CCNB1/SPP1/JAK2/TYMS/DHFR/FOLR1/FPGS/TGFBR2/CEBPB/CSNK2B | 24 |
| GO:0035265 | organ growth | 24/412 | 2.55E-11 | 4.52E-10 | AR/RXRA/ADRA1A/ESR1/MAPK14/SLC6A4/AKT1/BCL2/CDK1/MAPK1/POR/SOD1/GJA1/CCNB1/PPARA/CYP19A1/FGF2/FXN/TGFBR2/PIM1/RARA/RARB/RARG/G6PD | 24 |
| GO:0001894 | tissue homeostasis | 24/412 | 2.41E-10 | 3.43E-09 | ADRB2/AKR1B1/BCL2/BAX/EGFR/VEGFA/RB1/SOD1/PRKCA/ACACA/NOS3/HSPB1/SPP1/CA2/F2R/NOX4/CTSK/TF/TFRC/IL6/ACTB/ESRRB/PTK2B/RHO | 24 |
| GO:0071453 | cellular response to oxygen levels | 24/412 | 4.52E-10 | 5.96E-09 | PTGS2/PPARG/CCNA2/PPARD/OPRD1/AKT1/BCL2/HMOX1/ICAM1/PSMD3/SLC2A4/VEGFA/TP53/HIF1A/CAV1/MYC/CCNB1/NFE2L2/E2F1/NPEPPS/CA9/EGLN1/RORA/CACNB3 | 24 |
| GO:0045637 | regulation of myeloid cell differentiation | 24/412 | 1.88E-09 | 2.03E-08 | MAPK14/JUN/STAT1/FOS/EIF6/RB1/NFKBIA/CASP8/PRKCA/HIF1A/MYC/PRKCB/IFNG/CA2/CCR1/HDAC1/LGALS3/PTPN2/CDK6/CEBPB/ESRRA/PTK2B/RARA/RARG | 24 |
| GO:0043491 | protein kinase B signaling | 24/412 | 7.44E-09 | 6.82E-08 | ESR1/F7/KDR/MET/AKT1/INSR/AKR1C3/EGFR/EGF/ERBB2/F3/IL1B/CCL2/IGF2/ERBB3/VAV1/FGF1/FGF2/HPSE/HSP90AA1/NOX4/AKR1C2/GPER1/PIK3CG | 24 |
| GO:0007548 | sex differentiation | 24/412 | 8.00E-09 | 7.28E-08 | PGR/AR/NCOA1/ESR1/BCL2/BAX/CASP3/ICAM1/INSR/AKR1C3/VEGFA/CCND1/BCL2L1/SOD1/HSPA5/GJA1/NOS3/CYP19A1/CYP17A1/SRD5A1/SRD5A2/CEBPB/RARA/HOXA10 | 24 |
| GO:0070661 | leukocyte proliferation | 24/412 | 5.36E-08 | 3.92E-07 | BCL2/BAX/CASP3/VCAM1/GSTP1/AHR/CDKN1A/IL6ST/TP53/ERBB2/GJA1/IL1B/IL2/IGF2/CD40LG/IRF1/JAK3/ADA/LGALS3/TFRC/TGFBR2/IL6/CEBPB/PIK3CG | 24 |
| GO:0070371 | ERK1 and ERK2 cascade | 24/412 | 1.71E-07 | 1.07E-06 | ADRA1A/OPRM1/KDR/JUN/ICAM1/GSTP1/EGFR/MAPK1/EGF/PRKCA/ERBB2/MYC/IL1B/CCL2/PTPN1/NQO2/CCR1/F2R/HMGCR/FGF2/NOX4/PTPN2/GPER1/PTK2B | 24 |
| GO:0016042 | lipid catabolic process | 24/412 | 4.21E-07 | 2.41E-06 | PPARD/AKT1/CYP3A4/CYP1A2/CYP1B1/AKR1C3/IL1B/SULT1E1/PPARA/SPP1/CYP19A1/GBA/SRD5A1/NAGA/PLA2G2E/SULT2A1/AKR1D1/HSD17B11/HIBCH/PIK3CG/CES1/FABP6/PLA2G1B/CYP27B1 | 24 |
| GO:0032147 | activation of protein kinase activity | 24/412 | 4.21E-07 | 2.41E-06 | ADRB2/MAPK14/ADRA2C/AKT1/CDK1/INSR/EGFR/VEGFA/MAPK1/EGF/RAF1/SOD1/IL1B/CD40LG/PTPN1/NEK1/JAK2/F2R/FGF1/FGF2/GRM4/TGFBR2/PTK2B/PLA2G1B | 24 |
| GO:0030098 | lymphocyte differentiation | 24/412 | 1.20E-06 | 6.15E-06 | IL4R/BCL2/BAX/VCAM1/TP53/SOD1/ERBB2/IL2/IFNG/RUNX2/CD40LG/IRF1/JAK3/VAV1/ADA/RORC/PTPN2/TGFBR2/IL6/CLEC4E/RORA/CDK6/PTK2B/RARA | 24 |
| GO:0031331 | positive regulation of cellular catabolic process | 24/412 | 1.77E-06 | 8.58E-06 | ADRB2/GSK3B/KDR/AKT1/BAX/HMOX1/INSR/EGF/HIF1A/CAV1/IL1B/IFNG/NFE2L2/PPARA/HSF1/HK2/PTPN1/GBA/HDAC6/HSP90AA1/CTSC/IL6/ESRRB/PTK2B | 24 |
| GO:0042176 | regulation of protein catabolic process | 24/412 | 4.50E-06 | 1.88E-05 | NOS2/GSK3B/RELA/AKT1/PSMD3/EGFR/EGF/ODC1/CAV1/GJA1/IL1B/IFNG/NFE2L2/CHEK2/GBA/HMGCR/HSP90AA1/OGT/CTSC/GRIN2A/GRIN2C/CSNK2A1/PTK2B/SF3B3 | 24 |
| GO:0014706 | striated muscle tissue development | 24/412 | 6.69E-06 | 2.62E-05 | RXRA/ADRA1A/MAPK14/BCL2/CDK1/PPP3CA/VEGFA/FOS/MAPK1/RB1/CAV1/GJA1/CCNB1/PPARA/ERBB3/HMGCR/FGF2/KDM6B/NOX4/TGFBR2/PIM1/RARA/RARB/G6PD | 24 |
| GO:1901293 | nucleoside phosphate biosynthetic process | 24/412 | 6.69E-06 | 2.62E-05 | PTGS2/NOS2/ATP5F1B/INSR/EIF6/MAPK1/TP53/HIF1A/ACACA/NOS3/IFNG/PARP1/PPARA/HK2/ADK/ADA/TYMS/TK1/OGT/GCK/PAPSS1/ESRRB/NPPB/COX5B | 24 |
| GO:0006732 | coenzyme metabolic process | 24/412 | 1.16E-05 | 4.23E-05 | PTGS2/INSR/EIF6/TP53/HIF1A/ACACA/IFNG/PPARA/HK2/AHCYL2/TYMS/DHFR/FOLR1/FOLR2/SLC46A1/FPGS/HMGCR/OGT/GCK/CYB5A/ESRRB/ACOT13/DBI/G6PD | 24 |
| GO:0001558 | regulation of cell growth | 24/412 | 1.95E-05 | 6.57E-05 | ESR2/PPARG/GSK3B/PPARD/AKT1/BCL2/EGFR/VEGFA/CDKN1A/RB1/TP53/ERBB2/GJA1/IL2/PPARA/SPP1/IGFBP3/CDA/FXN/CSNK2A1/PTK2B/NPPB/G6PD/CYP27B1 | 24 |
| GO:0043312 | neutrophil degranulation | 24/412 | 0.000211914 | 0.000518836 | MAPK14/OLR1/TNFAIP6/ALOX5/GSTP1/PSMD3/SLPI/PLAU/MMP9/MAPK1/MPO/CTSD/MMP8/HSPA8/HPSE/HSP90AA1/CTSS/LGALS3/CDA/CTSC/FTH1/NFKB1/ORM1/CSNK2B | 24 |
| GO:0002283 | neutrophil activation involved in immune response | 24/412 | 0.000232115 | 0.000558572 | MAPK14/OLR1/TNFAIP6/ALOX5/GSTP1/PSMD3/SLPI/PLAU/MMP9/MAPK1/MPO/CTSD/MMP8/HSPA8/HPSE/HSP90AA1/CTSS/LGALS3/CDA/CTSC/FTH1/NFKB1/ORM1/CSNK2B | 24 |
| GO:2000379 | positive regulation of reactive oxygen species metabolic process | 23/412 | 3.68E-17 | 2.03E-15 | PTGS2/MAPK14/AKT1/ICAM1/GSTP1/INSR/AKR1C3/EGFR/CDKN1A/TP53/SOD1/IL1B/IFNG/NFE2L2/JAK2/NQO2/MMP8/HSP90AA1/NOX4/TGFBR2/GRIN1/TRPV1/PTK2B | 23 |
| GO:0060359 | response to ammonium ion | 23/412 | 2.40E-14 | 7.98E-13 | CHRM3/CHRM1/CHRM2/OPRM1/GABRA1/CCNA2/RELA/HTR3A/DRD1/CHRM5/CHRM4/SLC6A3/CASP3/MAPK1/HSPA5/ADA/GABRG2/GABRB1/GABRB2/GABRB3/GRIN1/OPRK1/PTK2B | 23 |
| GO:0035296 | regulation of tube diameter | 23/412 | 8.62E-14 | 2.51E-12 | PTGS2/CHRM3/ADRA1A/ADRB2/ADRA1B/PPARD/ADRB1/ADRA2C/DRD1/SLC6A4/AKT1/HMOX1/ICAM1/EGFR/SOD1/CAV1/GJA1/NOS3/ADRB3/F2R/HMGCR/GPER1/NPPB | 23 |
| GO:0050880 | regulation of blood vessel size | 23/412 | 8.62E-14 | 2.51E-12 | PTGS2/CHRM3/ADRA1A/ADRB2/ADRA1B/PPARD/ADRB1/ADRA2C/DRD1/SLC6A4/AKT1/HMOX1/ICAM1/EGFR/SOD1/CAV1/GJA1/NOS3/ADRB3/F2R/HMGCR/GPER1/NPPB | 23 |
| GO:0097746 | regulation of blood vessel diameter | 23/412 | 8.62E-14 | 2.51E-12 | PTGS2/CHRM3/ADRA1A/ADRB2/ADRA1B/PPARD/ADRB1/ADRA2C/DRD1/SLC6A4/AKT1/HMOX1/ICAM1/EGFR/SOD1/CAV1/GJA1/NOS3/ADRB3/F2R/HMGCR/GPER1/NPPB | 23 |
| GO:0035150 | regulation of tube size | 23/412 | 1.00E-13 | 2.86E-12 | PTGS2/CHRM3/ADRA1A/ADRB2/ADRA1B/PPARD/ADRB1/ADRA2C/DRD1/SLC6A4/AKT1/HMOX1/ICAM1/EGFR/SOD1/CAV1/GJA1/NOS3/ADRB3/F2R/HMGCR/GPER1/NPPB | 23 |
| GO:0014074 | response to purine-containing compound | 23/412 | 2.13E-13 | 5.49E-12 | PTGS2/PPARG/RELA/SLC6A3/SLC6A4/JUN/STAT1/AHR/GSTM2/FOS/SOD1/HSPA5/IL1B/DUOX2/THBD/COL1A1/TYR/NOX4/SRD5A1/CACNA1S/TRPV1/PIK3CG/PTK2B | 23 |
| GO:0060402 | calcium ion transport into cytosol | 23/412 | 7.60E-13 | 1.75E-11 | ADRA1A/DRD1/BCL2/BAX/GSTM2/CAV1/CXCL11/CXCL10/F2R/FGF2/TRPA1/ITPR1/GRIN1/GRIN2A/GRIN2B/GRIN2C/GRIN2D/CACNA1C/CACNB3/TRPV1/GPER1/PTK2B/PLA2G1B | 23 |
| GO:0031960 | response to corticosteroid | 23/412 | 1.30E-12 | 2.89E-11 | PTGS2/MAOB/HSD3B2/HSD3B1/BCL2/CASP3/ICAM1/GSTP1/AKR1C3/EGFR/CCND1/FOS/CDKN1A/CASP9/COL1A1/PARP1/NR3C1/GBA/TYMS/SRD5A1/HSD11B2/IL6/GPER1 | 23 |
| GO:0015698 | inorganic anion transport | 23/412 | 3.21E-12 | 6.72E-11 | GABRA1/CLDN4/ABCB1/CA2/CA7/GLRA1/GLRA2/GABRA2/GABRA3/GABRA4/GABRA5/GABRA6/GABRG1/GABRG2/GABRG3/GABRB1/GABRB2/GABRB3/GABRD/GABRE/GABRP/GABRQ/CEBPB | 23 |
| GO:0048660 | regulation of smooth muscle cell proliferation | 23/412 | 3.21E-12 | 6.72E-11 | PTGS2/PPARG/PPARD/AKR1B1/JUN/AKT1/STAT1/HMOX1/GSTP1/EGFR/CDKN1A/MMP2/MMP9/IFNG/IGFBP3/JAK2/NQO2/HMGCR/FGF2/COMT/TGFBR2/IL6/GPER1 | 23 |
| GO:0048659 | smooth muscle cell proliferation | 23/412 | 4.12E-12 | 8.21E-11 | PTGS2/PPARG/PPARD/AKR1B1/JUN/AKT1/STAT1/HMOX1/GSTP1/EGFR/CDKN1A/MMP2/MMP9/IFNG/IGFBP3/JAK2/NQO2/HMGCR/FGF2/COMT/TGFBR2/IL6/GPER1 | 23 |
| GO:0060401 | cytosolic calcium ion transport | 23/412 | 4.12E-12 | 8.21E-11 | ADRA1A/DRD1/BCL2/BAX/GSTM2/CAV1/CXCL11/CXCL10/F2R/FGF2/TRPA1/ITPR1/GRIN1/GRIN2A/GRIN2B/GRIN2C/GRIN2D/CACNA1C/CACNB3/TRPV1/GPER1/PTK2B/PLA2G1B | 23 |
| GO:0001659 | temperature homeostasis | 23/412 | 5.27E-12 | 1.03E-10 | PTGS2/ACHE/ADRB2/ADRB1/DRD1/IL4R/VEGFA/RB1/CAV1/GJA1/IL1B/PTGER3/IL1A/HSF1/JAK2/ADRB3/KDM6B/OGT/TRPM8/TRPV1/CEBPB/ACOT13/ESRRG | 23 |
| GO:0022900 | electron transport chain | 23/412 | 2.40E-11 | 4.27E-10 | AKR1B1/MAOB/NCF1/CDK1/CYP1A2/POR/CCNB1/NQO1/CYP19A1/NQO2/NOX4/SRD5A1/CYB5A/COX4I1/COX5A/COX5B/COX6A2/COX6B1/COX6C/COX7A1/COX7B/COX7C/COX8A | 23 |
| GO:1901215 | negative regulation of neuron death | 23/412 | 2.35E-10 | 3.38E-09 | GSK3B/JUN/AKT1/BCL2/BAX/HMOX1/BCL2L1/SOD1/HIF1A/CCL2/PPARA/HSF1/ERBB3/RASA1/JAK2/GBA/F2R/GABRA5/GABRB2/GABRB3/GRIN1/CEBPB/PTK2B | 23 |
| GO:0008406 | gonad development | 23/412 | 5.48E-10 | 7.04E-09 | PGR/AR/NCOA1/ESR1/BCL2/BAX/CASP3/ICAM1/INSR/AKR1C3/VEGFA/CCND1/BCL2L1/SOD1/HSPA5/GJA1/NOS3/CYP19A1/SRD5A1/SRD5A2/CEBPB/RARA/HOXA10 | 23 |
| GO:0007188 | adenylate cyclase-modulating G protein-coupled receptor signaling pathway | 23/412 | 7.86E-10 | 9.48E-09 | CHRM3/CHRM1/ADRA1A/CHRM2/ADRB2/OPRM1/ADRA1B/ADRB1/ADRA2C/ADRA1D/DRD1/CHRM5/CHRM4/OPRD1/PRKCA/PTGER3/CXCL11/CXCL10/ADRB3/GRM4/OPRK1/CACNA1D/GPER1 | 23 |
| GO:0045137 | development of primary sexual characteristics | 23/412 | 9.39E-10 | 1.12E-08 | PGR/AR/NCOA1/ESR1/BCL2/BAX/CASP3/ICAM1/INSR/AKR1C3/VEGFA/CCND1/BCL2L1/SOD1/HSPA5/GJA1/NOS3/CYP19A1/SRD5A1/SRD5A2/CEBPB/RARA/HOXA10 | 23 |
| GO:1904018 | positive regulation of vasculature development | 23/412 | 1.72E-09 | 1.89E-08 | PTGS2/KDR/HMOX1/CYP1B1/VEGFA/PRKCA/HIF1A/F3/IL1B/CXCL8/PRKCB/NOS3/HSPB1/SERPINE1/IL1A/NFE2L2/HK2/JAK1/FGF1/FGF2/TGFBR2/GPER1/PTK2B | 23 |
| GO:2000116 | regulation of cysteine-type endopeptidase activity | 23/412 | 3.63E-09 | 3.57E-08 | PTGS2/PPARG/DPEP1/AKT1/BAX/VEGFA/CASP9/MMP9/POR/CASP8/RAF1/MYC/F3/BIRC5/HSF1/CTSD/JAK2/F2R/GRIN1/GRIN2A/GRIN2B/CSNK2A1/GPER1 | 23 |
| GO:0046883 | regulation of hormone secretion | 23/412 | 2.76E-08 | 2.24E-07 | DPP4/NOS2/PPARD/ADRA2C/PPP3CA/EGFR/PRKCA/HIF1A/GJA1/IL1B/IFNG/SPP1/CYP19A1/JAK2/HMGCR/FFAR1/GCK/ITPR1/OPRK1/NR1H4/CACNA1C/CACNA1D/GPER1 | 23 |
| GO:0046651 | lymphocyte proliferation | 23/412 | 4.18E-08 | 3.19E-07 | BCL2/BAX/CASP3/VCAM1/AHR/CDKN1A/IL6ST/TP53/ERBB2/GJA1/IL1B/IL2/IGF2/CD40LG/IRF1/JAK3/ADA/LGALS3/TFRC/TGFBR2/IL6/CEBPB/PIK3CG | 23 |
| GO:0032943 | mononuclear cell proliferation | 23/412 | 4.78E-08 | 3.56E-07 | BCL2/BAX/CASP3/VCAM1/AHR/CDKN1A/IL6ST/TP53/ERBB2/GJA1/IL1B/IL2/IGF2/CD40LG/IRF1/JAK3/ADA/LGALS3/TFRC/TGFBR2/IL6/CEBPB/PIK3CG | 23 |
| GO:0010632 | regulation of epithelial cell migration | 23/412 | 1.43E-07 | 9.28E-07 | PTGS2/PPARG/KDR/MET/JUN/ATP5F1B/AKT1/HMOX1/HAS2/VEGFA/MMP9/PRKCA/HIF1A/NOS3/HSPB1/IFNG/NFE2L2/HDAC6/FGF1/FGF2/TGFBR2/CSNK2B/PTK2B | 23 |
| GO:0044843 | cell cycle G1/S phase transition | 23/412 | 2.20E-07 | 1.35E-06 | CDK2/CCNA2/AKT1/BCL2/BAX/CDK1/CYP1A1/PPP3CA/EGFR/CCND1/CDKN1A/RB1/TP53/MYC/CCL2/CCNB1/CHEK2/E2F1/TYMS/DHFR/PHF8/CDK6/ESRRB | 23 |
| GO:0051251 | positive regulation of lymphocyte activation | 23/412 | 1.61E-06 | 7.87E-06 | DPP4/IGHG1/IL4R/AKT1/BCL2/VCAM1/CDKN1A/IL6ST/CAV1/IL1B/CCL2/IL2/IFNG/IGF2/CD40LG/JAK3/VAV1/ADA/TFRC/TGFBR2/IL6/IGHG2/RARA | 23 |
| GO:0030198 | extracellular matrix organization | 23/412 | 8.09E-06 | 3.09E-05 | DPP4/PRSS1/KDR/MMP1/ICAM1/VCAM1/CYP1B1/HAS2/MMP3/MMP2/MMP9/RB1/SERPINE1/COL1A1/COL3A1/SPP1/MMP8/FGF2/CTSK/CTSS/IL6/NFKB2/CTRB1 | 23 |
| GO:0042692 | muscle cell differentiation | 23/412 | 1.67E-05 | 5.79E-05 | RXRA/ADRA1A/MAPK14/IL4R/AKT1/BCL2/CASP3/CDK1/PPP3CA/VEGFA/RB1/CCNB1/PPARA/CXCL10/CHUK/IGF2/KDM6B/NOX4/RORA/GPER1/RARA/RARB/G6PD | 23 |
| GO:0009165 | nucleotide biosynthetic process | 23/412 | 1.74E-05 | 6.01E-05 | PTGS2/NOS2/ATP5F1B/INSR/EIF6/MAPK1/TP53/HIF1A/ACACA/NOS3/IFNG/PARP1/PPARA/HK2/ADK/ADA/TYMS/OGT/GCK/PAPSS1/ESRRB/NPPB/COX5B | 23 |
| GO:0002683 | negative regulation of immune system process | 23/412 | 0.000270615 | 0.000638378 | PPARG/IL4R/AKT1/CASP3/HMOX1/NFKBIA/ERBB2/MYC/CCL2/IL2/NFE2L2/COL3A1/IRF1/CYP19A1/JAK3/ADA/LGALS3/PTPN2/CDK6/CEBPB/GPER1/PTK2B/RARA | 23 |
| GO:0043254 | regulation of protein complex assembly | 23/412 | 0.000305738 | 0.000708792 | ESR1/GSK3B/OPRD1/MET/JUN/BAX/MMP1/ICAM1/MMP3/VEGFA/RB1/TP53/RAF1/IFNG/PARP1/RASA1/HSPA8/GBA/HDAC6/HSP90AA1/ARF1/HCK/PTK2B | 23 |
| GO:0002429 | immune response-activating cell surface receptor signaling pathway | 23/412 | 0.000365945 | 0.000824951 | IGHG1/RELA/IKBKB/BCL2/BAX/PSMD3/MAPK1/RAF1/PRKCB/CHUK/RPS6KA5/VAV1/ADA/HSP90AA1/LGALS3/PTPN2/NFKB1/CLEC4E/CACNA1F/CACNB3/IGHG2/ACTB/HCK | 23 |
| GO:0050769 | positive regulation of neurogenesis | 23/412 | 0.000376932 | 0.000847455 | OPRM1/NCOA1/PCP4/PPARG/RELA/ADRA2C/BCL2/VEGFA/IL6ST/HIF1A/HSPA5/IL1B/IL2/IFNG/NFE2L2/E2F1/HDAC1/ARF1/IL6/GPER1/PTK2B/RARA/RARB | 23 |
| GO:0042446 | hormone biosynthetic process | 22/412 | 9.74E-18 | 5.87E-16 | AKR1B1/HSD3B2/HSD3B1/CYP3A4/DIO1/AKR1C3/POR/HIF1A/IL1B/DUOX2/IFNG/CYP19A1/HSD11B1/CYP17A1/SRD5A1/SRD5A2/VDR/HSD11B2/HSD17B1/NFKB1/HSD17B11/CYP27B1 | 22 |
| GO:0006821 | chloride transport | 22/412 | 1.79E-15 | 7.67E-14 | GABRA1/CLDN4/ABCB1/CA2/CA7/GLRA1/GLRA2/GABRA2/GABRA3/GABRA4/GABRA5/GABRA6/GABRG1/GABRG2/GABRG3/GABRB1/GABRB2/GABRB3/GABRD/GABRE/GABRP/GABRQ | 22 |
| GO:0006805 | xenobiotic metabolic process | 22/412 | 4.40E-14 | 1.39E-12 | PTGS1/DPEP1/CYP3A4/CYP1A2/CYP1A1/NR1I2/CYP1B1/GSTP1/AHR/GSTM1/GSTM2/POR/NQO1/NQO2/EPHX1/RORC/CYP2C19/CES2/AKR1C1/GRIN1/RORA/CES1 | 22 |
| GO:0032355 | response to estradiol | 22/412 | 1.95E-13 | 5.14E-12 | PTGS2/NCOA1/ESR1/CCNA2/F7/SLC6A4/CASP3/CYP1A2/GSTP1/EGFR/CCND1/CASP9/CASP8/GJA1/COL1A1/NQO1/HSF1/SRD5A1/ESRRA/GPER1/RUVBL2/RARA | 22 |
| GO:0046683 | response to organophosphorus | 22/412 | 1.95E-13 | 5.14E-12 | PTGS2/RELA/SLC6A3/SLC6A4/JUN/STAT1/AHR/FOS/SOD1/HSPA5/IL1B/DUOX2/THBD/COL1A1/TYR/TYMS/NOX4/SRD5A1/AKR1C1/TRPV1/PIK3CG/PTK2B | 22 |
| GO:0046165 | alcohol biosynthetic process | 22/412 | 4.72E-11 | 7.81E-10 | AKR1B1/CYP3A4/POR/SOD1/ACACA/IL1B/IFNG/NPC1L1/GBA/DHFR/HMGCR/FGF1/FGF2/VDR/ISYNA1/NFKB1/LSS/GPER1/PTK2B/CES1/G6PD/CYP27B1 | 22 |
| GO:0048771 | tissue remodeling | 22/412 | 7.40E-11 | 1.17E-09 | ADRB2/BAX/PPP3CA/EGFR/MMP2/TP53/PRKCA/HIF1A/CAV1/GJA1/NOS3/IL2/IL1A/SPP1/CA2/F2R/NOX4/CTSK/VDR/TFRC/IL6/PTK2B | 22 |
| GO:0006575 | cellular modified amino acid metabolic process | 22/412 | 7.78E-10 | 9.45E-09 | DPEP1/GSTP1/DIO1/GSTM1/GSTM2/POR/SOD1/DUOX2/NFE2L2/AHCYL2/TYMS/DHFR/FOLR1/FOLR2/SLC46A1/FPGS/PTGES/EGLN1/HPGDS/PLA2G2E/G6PD/PLA2G1B | 22 |
| GO:0045766 | positive regulation of angiogenesis | 22/412 | 9.39E-10 | 1.12E-08 | PTGS2/KDR/HMOX1/CYP1B1/VEGFA/PRKCA/HIF1A/F3/IL1B/CXCL8/PRKCB/NOS3/HSPB1/SERPINE1/IL1A/NFE2L2/HK2/JAK1/FGF1/FGF2/TGFBR2/PTK2B | 22 |
| GO:0006109 | regulation of carbohydrate metabolic process | 22/412 | 1.13E-09 | 1.31E-08 | NCOA2/GSK3B/AKT1/HAS2/INSR/EIF6/EGF/TP53/HIF1A/IFNG/PPARA/IGFBP3/IGF2/RORC/PTPN2/OGT/GCK/NFKB1/RORA/ESRRB/GPER1/PTK2B | 22 |
| GO:0036294 | cellular response to decreased oxygen levels | 22/412 | 3.02E-09 | 3.04E-08 | PTGS2/CCNA2/PPARD/OPRD1/AKT1/BCL2/HMOX1/ICAM1/PSMD3/SLC2A4/VEGFA/TP53/HIF1A/MYC/CCNB1/NFE2L2/E2F1/NPEPPS/CA9/EGLN1/RORA/CACNB3 | 22 |
| GO:1903039 | positive regulation of leukocyte cell-cell adhesion | 22/412 | 3.29E-09 | 3.27E-08 | DPP4/RELA/IL4R/AKT1/ICAM1/VCAM1/HAS2/IL6ST/CAV1/IL1B/CCL2/IL2/IFNG/IGF2/CD40LG/JAK3/VAV1/ADA/TFRC/TGFBR2/IL6/RARA | 22 |
| GO:0030072 | peptide hormone secretion | 22/412 | 4.06E-08 | 3.14E-07 | DPP4/NOS2/PPARD/ADRA2C/PPP3CA/EGFR/RAF1/PRKCA/HIF1A/GJA1/IL1B/IFNG/JAK2/HMGCR/FFAR1/GCK/ITPR1/IL6/NR1H4/CACNA1C/CACNA1D/GPER1 | 22 |
| GO:0022409 | positive regulation of cell-cell adhesion | 22/412 | 5.78E-08 | 4.19E-07 | DPP4/RELA/IL4R/AKT1/ICAM1/VCAM1/HAS2/IL6ST/CAV1/IL1B/CCL2/IL2/IFNG/IGF2/CD40LG/JAK3/VAV1/ADA/TFRC/TGFBR2/IL6/RARA | 22 |
| GO:0043406 | positive regulation of MAP kinase activity | 22/412 | 7.11E-08 | 5.03E-07 | MAPK14/CDK1/INSR/EGFR/VEGFA/MAPK1/EGF/RAF1/SOD1/ERBB2/IL1B/CD40LG/PTPN1/JAK2/F2R/FGF1/FGF2/NOX4/GRM4/PIK3CG/PTK2B/PLA2G1B | 22 |
| GO:0032412 | regulation of ion transmembrane transporter activity | 22/412 | 8.16E-08 | 5.66E-07 | ADRB2/OPRM1/GRIA2/HTR3A/GSTM2/MMP9/CAV1/CCL2/IFNG/ATP2A1/ABCB1/CTSS/GRIN1/GRIN2A/GRIN2B/CACNA1D/CACNB1/CACNB2/CACNB3/CACNB4/HSPA2/PTK2B | 22 |
| GO:0051091 | positive regulation of DNA-binding transcription factor activity | 22/412 | 8.73E-08 | 5.97E-07 | AR/ESR2/ESR1/PPARG/RELA/OPRD1/IKBKB/AKT1/ICAM1/PPP3CA/VEGFA/CAV1/IL1B/PRKCB/CHUK/CD40LG/RPS6KA5/JAK2/IL6/NFKB1/NFKB2/PLA2G1B | 22 |
| GO:1902105 | regulation of leukocyte differentiation | 22/412 | 1.80E-07 | 1.12E-06 | JUN/IL4R/FOS/RB1/CASP8/SOD1/PRKCA/ERBB2/MYC/IL2/IFNG/IRF1/JAK3/CA2/ADA/CCR1/PTPN2/TGFBR2/CDK6/CEBPB/ESRRA/RARA | 22 |
| GO:0000082 | G1/S transition of mitotic cell cycle | 22/412 | 2.79E-07 | 1.70E-06 | CDK2/AKT1/BCL2/BAX/CDK1/CYP1A1/PPP3CA/EGFR/CCND1/CDKN1A/RB1/TP53/MYC/CCL2/CCNB1/CHEK2/E2F1/TYMS/DHFR/PHF8/CDK6/ESRRB | 22 |
| GO:0044262 | cellular carbohydrate metabolic process | 22/412 | 4.27E-07 | 2.45E-06 | NCOA2/GSK3B/PYGM/AKR1B1/AKT1/HAS2/INSR/IL6ST/TP53/PPARA/IGFBP3/IGF2/HK2/RORC/PTPN2/OGT/GCK/ISYNA1/RORA/ESRRB/GPER1/PTK2B | 22 |
| GO:1903829 | positive regulation of cellular protein localization | 22/412 | 3.41E-06 | 1.49E-05 | PTGS2/CHRM1/MAPK14/GSK3B/AKT1/BCL2/MAPK8/CDK1/EGFR/MAPK1/EGF/TP53/CASP8/ERBB2/IL1B/IFNG/PARP1/E2F1/NPEPPS/LGALS3/CACNB3/GPER1 | 22 |
| GO:0009615 | response to virus | 22/412 | 3.76E-06 | 1.60E-05 | CHRM2/MAPK14/RELA/IKBKB/BCL2/STAT1/CYP1A1/BCL2L1/IL10RB/ODC1/IL1B/DUOX2/HSPB1/IFNG/CXCL10/CHUK/IRF1/LGALS8/ARF1/IL6/OPRK1/CDK6 | 22 |
| GO:0051188 | cofactor biosynthetic process | 22/412 | 3.76E-06 | 1.60E-05 | PTGS2/MAOB/CYP1A2/CYP1A1/INSR/EIF6/TP53/SOD1/HIF1A/ACACA/DUOX2/IFNG/NFE2L2/PPARA/HK2/DHFR/FPGS/OGT/GCK/FXN/ESRRB/FECH | 22 |
| GO:0048568 | embryonic organ development | 22/412 | 0.00022857 | 0.000552988 | NCOA1/KDR/AKT1/EGFR/VEGFA/MAPK1/TP53/CASP8/SOD1/HIF1A/GJA1/CXCL8/HSF1/RUNX2/IGF2/ADA/FOLR1/TGFBR2/CEBPB/RARA/RARB/RARG | 22 |
| GO:0042326 | negative regulation of phosphorylation | 22/412 | 0.000764874 | 0.001552798 | PKIA/JUN/AKT1/BAX/CASP3/GSTP1/CDKN1A/RB1/CAV1/MYC/IL1B/HSPB1/IL2/CCNB1/IFNG/PPARA/IGFBP3/PTPN1/GBA/HMGCR/PTPN2/GPER1 | 22 |
| GO:0033273 | response to vitamin | 21/412 | 8.59E-16 | 3.88E-14 | PTGS2/PPARG/PPARD/F7/RELA/CYP1A1/GSTP1/EGFR/CCND1/COL1A1/CXCL10/SPP1/TYR/ADA/TYMS/FOLR1/FOLR2/VDR/PIM1/RARA/CYP27B1 | 21 |
| GO:0051928 | positive regulation of calcium ion transport | 21/412 | 3.13E-13 | 7.93E-12 | DRD1/BAX/GSTM2/CAV1/CCL2/CXCL11/CXCL10/ATP2A1/CCR1/F2R/FFAR1/LGALS3/GRIN1/CACNA1D/CACNB2/CACNB3/TRPV3/GPER1/HSPA2/G6PD/PLA2G1B | 21 |
| GO:0034767 | positive regulation of ion transmembrane transport | 21/412 | 3.53E-11 | 6.13E-10 | ADRB2/HTR3A/DRD1/KCNH2/BAX/GSTM2/CCL2/IFNG/CXCL11/CXCL10/ATP2A1/ABCB1/F2R/CTSS/ARF1/OPRK1/CACNB2/CACNB3/GPER1/HSPA2/G6PD | 21 |
| GO:0010634 | positive regulation of epithelial cell migration | 21/412 | 2.04E-10 | 3.02E-09 | PTGS2/KDR/MET/JUN/ATP5F1B/AKT1/HMOX1/HAS2/VEGFA/MMP9/PRKCA/HIF1A/NOS3/HSPB1/IFNG/NFE2L2/HDAC6/FGF1/FGF2/TGFBR2/PTK2B | 21 |
| GO:0002573 | myeloid leukocyte differentiation | 21/412 | 5.28E-09 | 5.00E-08 | PPARG/MAPK14/JUN/VEGFA/FOS/MMP9/RB1/CASP8/PRKCA/MYC/IFNG/PARP1/CA2/CCR1/PTPN2/TFRC/TGFBR2/CDK6/CEBPB/ESRRA/RARA | 21 |
| GO:0035637 | multicellular organismal signaling | 21/412 | 5.28E-09 | 5.00E-08 | SCN5A/DRD1/KCNH2/CHRM5/SOD1/CAV1/GJA1/ATP2A1/GBA/GLRA1/TYMP/ITPR1/CACNA1C/CACNA1D/CACNA1F/CACNA1S/CACNB1/CACNB2/CACNB3/CACNB4/GPER1 | 21 |
| GO:0071456 | cellular response to hypoxia | 21/412 | 6.86E-09 | 6.31E-08 | PTGS2/CCNA2/PPARD/OPRD1/AKT1/BCL2/HMOX1/ICAM1/PSMD3/SLC2A4/VEGFA/TP53/HIF1A/MYC/CCNB1/NFE2L2/E2F1/NPEPPS/CA9/EGLN1/RORA | 21 |
| GO:0051896 | regulation of protein kinase B signaling | 21/412 | 1.21E-07 | 7.99E-07 | ESR1/F7/MET/AKT1/INSR/AKR1C3/EGFR/EGF/ERBB2/F3/IGF2/ERBB3/VAV1/FGF1/FGF2/HPSE/HSP90AA1/NOX4/AKR1C2/GPER1/PIK3CG | 21 |
| GO:0042180 | cellular ketone metabolic process | 21/412 | 1.59E-07 | 1.01E-06 | PTGS2/PPARG/AKR1B1/AKT1/PSMD3/AKR1C3/EIF6/ODC1/CAV1/IL1B/NQO1/PPARA/CYP19A1/HMGCR/CYP17A1/SRD5A1/SRD5A2/COMT/AKR1C1/AKR1C2/NR1H4 | 21 |
| GO:0045927 | positive regulation of growth | 21/412 | 6.54E-07 | 3.61E-06 | MAPK14/PPARD/SLC6A3/AKT1/BCL2/CDK1/INSR/EGFR/VEGFA/MAPK1/ERBB2/IL2/CCNB1/HSF1/IGF2/FGF2/FXN/TGFBR2/CSNK2A1/PIM1/PTK2B | 21 |
| GO:0042063 | gliogenesis | 21/412 | 2.07E-06 | 9.83E-06 | CHRM1/PPARG/RELA/DRD1/AKT1/CDK1/GSTP1/EGFR/MAPK1/IL6ST/SOD1/ERBB2/IL1B/CCL2/IFNG/E2F1/ERBB3/HDAC1/IL6/CDK6/PTK2B | 21 |
| GO:0005996 | monosaccharide metabolic process | 21/412 | 2.31E-06 | 1.09E-05 | NCOA2/MAPK14/GSK3B/PPARD/AKR1B1/AKT1/INSR/TP53/PPARA/IGFBP3/IGF2/HK2/RORC/PTPN2/OGT/GCK/UGT2B7/CYB5A/RORA/ESRRB/G6PD | 21 |
| GO:0051146 | striated muscle cell differentiation | 21/412 | 2.44E-06 | 1.13E-05 | RXRA/ADRA1A/MAPK14/IL4R/AKT1/BCL2/CASP3/CDK1/PPP3CA/VEGFA/RB1/CCNB1/PPARA/CXCL10/CHUK/IGF2/KDM6B/NOX4/RARA/RARB/G6PD | 21 |
| GO:0050890 | cognition | 21/412 | 2.87E-06 | 1.29E-05 | PTGS2/CHRM1/DRD1/SLC6A4/JUN/CASP3/INSR/EGFR/FOS/MAPK1/HIF1A/NQO2/HMGCR/COMT/GABRA5/FEN1/GRIN1/GRIN2A/GRIN2B/OPRK1/CEBPB | 21 |
| GO:0070372 | regulation of ERK1 and ERK2 cascade | 21/412 | 3.54E-06 | 1.54E-05 | ADRA1A/OPRM1/KDR/JUN/ICAM1/GSTP1/EGFR/PRKCA/ERBB2/IL1B/CCL2/PTPN1/NQO2/CCR1/F2R/HMGCR/FGF2/NOX4/PTPN2/GPER1/PTK2B | 21 |
| GO:0009895 | negative regulation of catabolic process | 21/412 | 5.33E-06 | 2.16E-05 | ADRA1A/NOS2/MAPK14/RELA/MET/AKT1/BCL2/HMOX1/EGFR/TP53/IL1B/PPARA/E2F1/HMGCR/MCL1/OGT/GRIN2A/GRIN2C/CSNK2A1/PIK3CG/SF3B3 | 21 |
| GO:0031098 | stress-activated protein kinase signaling cascade | 21/412 | 7.54E-06 | 2.91E-05 | MAPK14/AKR1B1/NCF1/IKBKB/AKT1/MAPK8/GSTP1/EGFR/VEGFA/MAPK1/MYC/IL1B/CHUK/CD40LG/PTPN1/NEK1/MMP8/HMGCR/NFKB1/OPRK1/PTK2B | 21 |
| GO:0001655 | urogenital system development | 21/412 | 1.53E-05 | 5.34E-05 | AR/RXRA/ESR1/AKR1B1/BCL2/BAX/STAT1/HAS2/VEGFA/CASP9/MMP9/ODC1/MYC/CYP19A1/CA2/FGF1/FGF2/SRD5A1/RARA/RARB/RARG | 21 |
| GO:0007517 | muscle organ development | 21/412 | 0.000330895 | 0.000758775 | RXRA/ACHE/MAPK14/BCL2/CDK1/PPP3CA/FOS/MAPK1/RB1/CAV1/GJA1/CCNB1/COL3A1/PPARA/CXCL10/ERBB3/HMGCR/FGF2/TGFBR2/PIM1/G6PD | 21 |
| GO:0001933 | negative regulation of protein phosphorylation | 21/412 | 0.000598514 | 0.001260554 | PKIA/JUN/AKT1/BAX/CASP3/GSTP1/CDKN1A/RB1/CAV1/MYC/IL1B/HSPB1/IL2/CCNB1/IFNG/IGFBP3/PTPN1/GBA/HMGCR/PTPN2/GPER1 | 21 |
| GO:1901987 | regulation of cell cycle phase transition | 21/412 | 0.002399818 | 0.004023397 | CDK2/CHEK1/PKIA/AKT1/BCL2/BAX/CDK1/CYP1A1/PSMD3/EGFR/CCND1/CDKN1A/RB1/TP53/CCL2/CCNB1/CHEK2/E2F1/HSP90AA1/CDK6/HSPA2 | 21 |
| GO:0042136 | neurotransmitter biosynthetic process | 20/412 | 1.62E-13 | 4.41E-12 | PTGS2/ACHE/NOS2/SLC6A3/SLC6A4/AKT1/ICAM1/CYP1B1/INSR/CAV1/IL1B/NOS3/IFNG/NQO1/JAK2/MMP8/HSP90AA1/RORA/TRPV1/PTK2B | 20 |
| GO:0006766 | vitamin metabolic process | 20/412 | 1.52E-11 | 2.77E-10 | RXRA/PRSS1/PPARD/CYP3A4/CYP1A1/AKR1C3/IL1B/IFNG/GC/DHFR/FOLR1/FOLR2/SLC46A1/FPGS/VDR/CYB5A/NFKB1/CBR1/CTRB1/CYP27B1 | 20 |
| GO:0030879 | mammary gland development | 20/412 | 5.14E-11 | 8.45E-10 | PGR/AR/NCOA1/ESR1/SLC6A3/AKT1/BAX/VEGFA/CCND1/MAPK1/EGF/HIF1A/CAV1/GJA1/HK2/CYP19A1/JAK2/VDR/TGFBR2/CEBPB | 20 |
| GO:0010212 | response to ionizing radiation | 20/412 | 8.57E-11 | 1.35E-09 | MAPK14/BCL2/BAX/CASP3/ICAM1/VCAM1/CCND1/BCL2L1/CDKN1A/TP53/ELK1/HSPA5/MYC/THBD/PARP1/CHEK2/HSF1/CXCL10/NOX4/POLB | 20 |
| GO:0001890 | placenta development | 20/412 | 1.58E-10 | 2.40E-09 | PTGS2/RXRA/NCOA1/PPARG/MAPK14/PPARD/AKT1/EGFR/MAPK1/CASP8/SOD1/HIF1A/GJA1/HSF1/SPP1/IGF2/ADA/VDR/CEBPB/CYP27B1 | 20 |
| GO:1902652 | secondary alcohol metabolic process | 20/412 | 2.26E-10 | 3.27E-09 | RXRA/PPARD/CYP3A4/POR/SOD1/ACACA/PON1/NPC1L1/GBA/HMGCR/FGF1/SOAT1/SOAT2/SULT2B1/LSS/NR1H4/AKR1D1/CES1/G6PD/CYP27B1 | 20 |
| GO:0016125 | sterol metabolic process | 20/412 | 7.80E-10 | 9.45E-09 | RXRA/PPARD/CYP1B1/POR/SOD1/ACACA/PON1/CYP19A1/NPC1L1/GBA/HMGCR/FGF1/SOAT1/SOAT2/SULT2B1/LSS/NR1H4/AKR1D1/CES1/G6PD | 20 |
| GO:0050806 | positive regulation of synaptic transmission | 20/412 | 9.66E-10 | 1.14E-08 | PTGS2/ADRA1A/ADRB2/GSK3B/DRD1/EGFR/MAPK1/CCL2/CA2/CA7/GRIN1/GRIN2A/GRIN2B/GRIN2C/GRIN2D/CACNA1D/CACNB2/CACNB3/GPER1/PTK2B | 20 |
| GO:1903034 | regulation of response to wounding | 20/412 | 2.96E-09 | 2.99E-08 | PPARD/F7/CDKN1A/PLAU/PRKCA/CAV1/F3/GJA1/DUOX2/NOS3/PLAT/THBD/SERPINE1/NFE2L2/SPP1/F2R/HMGCR/FGF2/HPSE/TGFBR2 | 20 |
| GO:0008217 | regulation of blood pressure | 20/412 | 3.96E-09 | 3.83E-08 | PTGS2/AR/ADRA1A/ADRB2/NOS2/PTGS1/ADRA1B/PPARG/ADRB1/HMOX1/SOD1/GJA1/NOS3/PPARA/ADRB3/F2R/HSD11B2/TRPV1/NPPB/CES1 | 20 |
| GO:0097164 | ammonium ion metabolic process | 20/412 | 3.05E-08 | 2.46E-07 | ACHE/AKR1B1/MAOB/DRD1/SLC6A3/AKR1C3/POR/ODC1/PON1/GBA/SRD5A1/COMT/PLA2G2E/AKR1C1/AKR1C2/GRIN2A/CSNK2A1/CSNK2B/DBI/PLA2G1B | 20 |
| GO:0030073 | insulin secretion | 20/412 | 3.59E-08 | 2.83E-07 | DPP4/NOS2/PPARD/ADRA2C/PPP3CA/RAF1/PRKCA/HIF1A/GJA1/IL1B/IFNG/JAK2/HMGCR/FFAR1/GCK/ITPR1/NR1H4/CACNA1C/CACNA1D/GPER1 | 20 |
| GO:0090276 | regulation of peptide hormone secretion | 20/412 | 3.90E-08 | 3.03E-07 | DPP4/NOS2/PPARD/ADRA2C/PPP3CA/EGFR/PRKCA/HIF1A/GJA1/IL1B/IFNG/JAK2/HMGCR/FFAR1/GCK/ITPR1/NR1H4/CACNA1C/CACNA1D/GPER1 | 20 |
| GO:0043281 | regulation of cysteine-type endopeptidase activity involved in apoptotic process | 20/412 | 6.77E-08 | 4.80E-07 | PTGS2/PPARG/DPEP1/AKT1/BAX/VEGFA/CASP9/MMP9/POR/CASP8/RAF1/MYC/F3/BIRC5/HSF1/CTSD/JAK2/F2R/CSNK2A1/GPER1 | 20 |
| GO:0032869 | cellular response to insulin stimulus | 20/412 | 7.31E-08 | 5.14E-07 | PPARG/GSK3B/RELA/AKT1/STAT1/GSTP1/SLC2A4/INSR/IL1B/PRKCB/PARP1/IGF2/PTPN1/PTPN2/OGT/SRD5A1/GCK/NR1H4/ESRRA/PLA2G1B | 20 |
| GO:0019722 | calcium-mediated signaling | 20/412 | 8.51E-08 | 5.84E-07 | GSK3B/KDR/SELE/VCAM1/PPP3CA/GSTM2/EGFR/CXCL8/ERBB3/ADA/CCR1/NMUR2/ITPR1/GRIN1/GRIN2A/GRIN2B/GRIN2C/GRIN2D/CACNA1C/PTK2B | 20 |
| GO:0070663 | regulation of leukocyte proliferation | 20/412 | 1.15E-07 | 7.61E-07 | BCL2/CASP3/VCAM1/GSTP1/AHR/CDKN1A/IL6ST/ERBB2/IL1B/IL2/IGF2/CD40LG/IRF1/JAK3/ADA/LGALS3/TFRC/TGFBR2/IL6/CEBPB | 20 |
| GO:0030217 | T cell differentiation | 20/412 | 4.07E-07 | 2.36E-06 | IL4R/BCL2/TP53/SOD1/ERBB2/IL2/IFNG/RUNX2/IRF1/JAK3/VAV1/ADA/RORC/PTPN2/TGFBR2/IL6/CLEC4E/RORA/CDK6/RARA | 20 |
| GO:0007611 | learning or memory | 20/412 | 1.13E-06 | 5.85E-06 | PTGS2/DRD1/SLC6A4/JUN/CASP3/INSR/EGFR/FOS/MAPK1/HIF1A/NQO2/HMGCR/COMT/GABRA5/FEN1/GRIN1/GRIN2A/GRIN2B/OPRK1/CEBPB | 20 |
| GO:0050730 | regulation of peptidyl-tyrosine phosphorylation | 20/412 | 1.13E-06 | 5.85E-06 | ADRA1A/NCF1/ICAM1/EGFR/VEGFA/EGF/IL6ST/TP53/CAV1/IL2/IFNG/HSF1/IGF2/ERBB3/PTPN1/JAK2/NOX4/PTPN2/IL6/PTK2B | 20 |
| GO:0006836 | neurotransmitter transport | 20/412 | 2.43E-06 | 1.13E-05 | ADRA1A/CHRM2/SLC6A2/GSK3B/MAOB/DRD1/SLC6A3/SLC6A4/PRKCB/SLC29A1/HSPA8/ADA/BACE1/GRM4/GABRA2/GABRQ/GRIN3A/CACNA1D/CACNB2/GPER1 | 20 |
| GO:0043542 | endothelial cell migration | 20/412 | 3.04E-06 | 1.35E-05 | PTGS2/DPP4/PPARG/KDR/MET/ATP5F1B/AKT1/HMOX1/CYP1B1/VEGFA/PRKCA/HIF1A/NOS3/HSPB1/NFE2L2/FGF1/FGF2/LGALS8/CSNK2B/PTK2B | 20 |
| GO:0090068 | positive regulation of cell cycle process | 20/412 | 1.13E-05 | 4.13E-05 | CDK2/AKT1/BAX/CDK1/CYP1A1/INSR/EGFR/CCND1/CDKN1A/EGF/RB1/TP53/IL1B/CCNB1/IL1A/CHEK2/E2F1/IGF2/FEN1/HSPA2 | 20 |
| GO:0006164 | purine nucleotide biosynthetic process | 20/412 | 1.24E-05 | 4.50E-05 | NOS2/ATP5F1B/INSR/EIF6/TP53/HIF1A/ACACA/NOS3/IFNG/PARP1/PPARA/HK2/ADK/ADA/OGT/GCK/PAPSS1/ESRRB/NPPB/COX5B | 20 |
| GO:0072522 | purine-containing compound biosynthetic process | 20/412 | 2.29E-05 | 7.58E-05 | NOS2/ATP5F1B/INSR/EIF6/TP53/HIF1A/ACACA/NOS3/IFNG/PARP1/PPARA/HK2/ADK/ADA/OGT/GCK/PAPSS1/ESRRB/NPPB/COX5B | 20 |
| GO:0060562 | epithelial tube morphogenesis | 20/412 | 3.43E-05 | 0.000106056 | PGR/AR/ESR1/MET/BCL2/CASP3/VEGFA/EGF/HIF1A/MYC/GJA1/CXCL10/FOLR1/FGF1/FGF2/VDR/TGFBR2/CSNK2B/RARA/RARG | 20 |
| GO:0006790 | sulfur compound metabolic process | 20/412 | 0.000244475 | 0.000584979 | DPEP1/GSTP1/GSTM1/GSTM2/SOD1/ACACA/SULT1E1/NFE2L2/AHCYL2/NOX4/PTGES/COMT/FXN/HPGDS/PAPSS1/SULT2A1/SULT2B1/ACOT13/DBI/G6PD | 20 |
| GO:0001701 | in utero embryonic development | 20/412 | 0.000253185 | 0.000601839 | AR/RXRA/NCOA1/AKT1/EGFR/VEGFA/BCL2L1/MAPK1/TP53/CASP8/HIF1A/GJA1/NOS3/CCNB1/HSF1/IGF2/ADA/POLB/TGFBR2/CEBPB | 20 |
| GO:0030900 | forebrain development | 20/412 | 0.000333234 | 0.000763447 | SCN5A/NCOA1/GSK3B/DRD1/SLC6A3/HSD3B2/HSD3B1/BAX/CASP3/EGFR/HIF1A/COL3A1/E2F1/HDAC1/KDM6B/SRD5A1/GRIN1/CDK6/RARA/RARB | 20 |
| GO:0050808 | synapse organization | 20/412 | 0.000788552 | 0.001586868 | ACHE/GABRA1/MAPK14/DRD1/INSR/ERBB2/F2R/HDAC6/GABRA2/GABRG2/ARF1/GABRB2/GABRB3/GRIN1/GRIN2B/CACNB1/CACNB2/CACNB3/CACNB4/ACTB | 20 |
| GO:1901990 | regulation of mitotic cell cycle phase transition | 20/412 | 0.00216188 | 0.003670671 | CDK2/PKIA/AKT1/BCL2/BAX/CDK1/CYP1A1/PSMD3/EGFR/CCND1/CDKN1A/RB1/TP53/CCL2/CCNB1/CHEK2/E2F1/HSP90AA1/CDK6/HSPA2 | 20 |
| GO:0044282 | small molecule catabolic process | 20/412 | 0.002218748 | 0.003753126 | NOS2/PPARD/AKT1/CYP1A1/AKR1C3/TP53/NOS3/SULT1E1/PPARA/PON1/HK2/ADA/FGF2/TYMP/CDA/GCK/SULT2A1/AKR1D1/HIBCH/CYP27B1 | 20 |
| GO:1902476 | chloride transmembrane transport | 19/412 | 4.97E-14 | 1.55E-12 | GABRA1/CLDN4/GLRA1/GLRA2/GABRA2/GABRA3/GABRA4/GABRA5/GABRA6/GABRG1/GABRG2/GABRG3/GABRB1/GABRB2/GABRB3/GABRD/GABRE/GABRP/GABRQ | 19 |
| GO:0098661 | inorganic anion transmembrane transport | 19/412 | 4.08E-12 | 8.21E-11 | GABRA1/CLDN4/GLRA1/GLRA2/GABRA2/GABRA3/GABRA4/GABRA5/GABRA6/GABRG1/GABRG2/GABRG3/GABRB1/GABRB2/GABRB3/GABRD/GABRE/GABRP/GABRQ | 19 |
| GO:1904064 | positive regulation of cation transmembrane transport | 19/412 | 4.36E-10 | 5.78E-09 | ADRB2/DRD1/KCNH2/BAX/GSTM2/CCL2/IFNG/CXCL11/CXCL10/ATP2A1/F2R/CTSS/ARF1/OPRK1/CACNB2/CACNB3/GPER1/HSPA2/G6PD | 19 |
| GO:0051384 | response to glucocorticoid | 19/412 | 5.54E-10 | 7.04E-09 | PTGS2/MAOB/HSD3B2/HSD3B1/BCL2/CASP3/ICAM1/GSTP1/EGFR/CCND1/FOS/CDKN1A/CASP9/NR3C1/GBA/TYMS/SRD5A1/HSD11B2/IL6 | 19 |
| GO:0061041 | regulation of wound healing | 19/412 | 7.01E-10 | 8.73E-09 | PPARD/F7/CDKN1A/PLAU/PRKCA/CAV1/F3/GJA1/DUOX2/NOS3/PLAT/THBD/SERPINE1/NFE2L2/F2R/HMGCR/FGF2/HPSE/TGFBR2 | 19 |
| GO:0016999 | antibiotic metabolic process | 19/412 | 9.90E-10 | 1.17E-08 | ADH1B/ADH1C/AKR1B1/MAOB/DPEP1/CYP1A2/CYP1A1/AKR1C3/MMP3/EGFR/SOD1/DUOX2/SULT1E1/MPO/HDAC6/HBA1/AKR1C1/AKR1C2/SULT2A1 | 19 |
| GO:1901568 | fatty acid derivative metabolic process | 19/412 | 5.46E-09 | 5.14E-08 | PTGS2/PTGS1/DPEP1/CYP1A2/CYP1A1/CYP1B1/ALOX5/AKR1C3/ACACA/IL1B/PON1/CYP2C19/TBXAS1/PTGES/CES2/HPGDS/AKR1C2/CBR1/PLA2G1B | 19 |
| GO:0009408 | response to heat | 19/412 | 1.31E-08 | 1.14E-07 | PTGS2/HSP90AA2P/GSK3B/AKT1/HMOX1/CDKN1A/MAPK1/SOD1/HSPA5/NOS3/IL1A/HSF1/CXCL10/HSPA8/FGF1/HSP90AA1/TRPV3/TRPV1/HSPA2 | 19 |
| GO:0050796 | regulation of insulin secretion | 19/412 | 1.31E-08 | 1.14E-07 | DPP4/NOS2/PPARD/ADRA2C/PPP3CA/PRKCA/HIF1A/GJA1/IL1B/IFNG/JAK2/HMGCR/FFAR1/GCK/ITPR1/NR1H4/CACNA1C/CACNA1D/GPER1 | 19 |
| GO:0051897 | positive regulation of protein kinase B signaling | 19/412 | 1.31E-08 | 1.14E-07 | ESR1/F7/MET/INSR/AKR1C3/EGFR/EGF/ERBB2/F3/IGF2/ERBB3/VAV1/FGF1/FGF2/HPSE/HSP90AA1/NOX4/AKR1C2/PIK3CG | 19 |
| GO:0042098 | T cell proliferation | 19/412 | 2.71E-08 | 2.23E-07 | BAX/CASP3/VCAM1/IL6ST/TP53/ERBB2/GJA1/IL1B/IL2/IGF2/CD40LG/IRF1/JAK3/LGALS3/TFRC/TGFBR2/IL6/CEBPB/PIK3CG | 19 |
| GO:0050870 | positive regulation of T cell activation | 19/412 | 1.21E-07 | 7.99E-07 | DPP4/IL4R/AKT1/VCAM1/IL6ST/CAV1/IL1B/CCL2/IL2/IFNG/IGF2/CD40LG/JAK3/VAV1/ADA/TFRC/TGFBR2/IL6/RARA | 19 |
| GO:0070555 | response to interleukin-1 | 19/412 | 1.78E-07 | 1.11E-06 | RELA/IKBKB/ICAM1/SELE/HAS2/PSMD3/NFKBIA/PRKCA/HIF1A/IL1B/CCL2/CXCL8/IL1A/CHUK/RPS6KA5/IL6/NFKB1/RORA/CEBPB | 19 |
| GO:0050670 | regulation of lymphocyte proliferation | 19/412 | 1.92E-07 | 1.19E-06 | BCL2/CASP3/VCAM1/AHR/CDKN1A/IL6ST/ERBB2/IL1B/IL2/IGF2/CD40LG/IRF1/JAK3/ADA/LGALS3/TFRC/TGFBR2/IL6/CEBPB | 19 |
| GO:0032944 | regulation of mononuclear cell proliferation | 19/412 | 2.07E-07 | 1.28E-06 | BCL2/CASP3/VCAM1/AHR/CDKN1A/IL6ST/ERBB2/IL1B/IL2/IGF2/CD40LG/IRF1/JAK3/ADA/LGALS3/TFRC/TGFBR2/IL6/CEBPB | 19 |
| GO:0050920 | regulation of chemotaxis | 19/412 | 3.72E-07 | 2.18E-06 | MAPK14/F7/KDR/MET/GSTP1/VEGFA/F3/CCL2/CXCL8/HSPB1/SERPINE1/CXCL10/CYP19A1/CCR1/FGF1/FGF2/PTPN2/IL6/PTK2B | 19 |
| GO:0002526 | acute inflammatory response | 19/412 | 4.59E-07 | 2.61E-06 | PTGS2/OPRM1/IGHG1/PPARG/ICAM1/VCAM1/GSTP1/IL6ST/F3/IL1B/PTGER3/IL1A/PTGES/IL6/ORM1/IGHG2/TRPV1/CEBPB/PIK3CG | 19 |
| GO:0045444 | fat cell differentiation | 19/412 | 5.65E-07 | 3.16E-06 | PTGS2/ADRB2/PPARG/MAPK14/PPARD/ADRB1/AKT1/SLC2A4/CCND1/RUNX1T1/SULT1E1/E2F1/HDAC6/RORC/IL6/RORA/CEBPB/GPER1/MB | 19 |
| GO:0009743 | response to carbohydrate | 19/412 | 9.05E-07 | 4.84E-06 | PTGS2/PPARD/CASP3/ICAM1/GSTP1/PPP3CA/RAF1/HIF1A/GJA1/IL1B/PRKCB/HMGCR/NOX4/GCK/TGFBR2/OPRK1/NR1H4/GPER1/PTK2B | 19 |
| GO:0019318 | hexose metabolic process | 19/412 | 2.96E-06 | 1.32E-05 | NCOA2/MAPK14/GSK3B/PPARD/AKR1B1/AKT1/INSR/TP53/PPARA/IGFBP3/IGF2/HK2/RORC/PTPN2/OGT/GCK/RORA/ESRRB/G6PD | 19 |
| GO:0045926 | negative regulation of growth | 19/412 | 2.96E-06 | 1.32E-05 | ADRB2/ESR2/PPARG/PPARD/ADRB1/SLC6A4/BCL2/CDKN1A/TP53/HIF1A/GJA1/PPARA/SPP1/CDA/FXN/TGFBR2/NPPB/G6PD/CYP27B1 | 19 |
| GO:0051403 | stress-activated MAPK cascade | 19/412 | 2.14E-05 | 7.20E-05 | MAPK14/NCF1/IKBKB/AKT1/MAPK8/GSTP1/EGFR/VEGFA/MAPK1/MYC/IL1B/CHUK/CD40LG/PTPN1/MMP8/HMGCR/NFKB1/OPRK1/PTK2B | 19 |
| GO:0007162 | negative regulation of cell adhesion | 19/412 | 2.48E-05 | 8.11E-05 | IL4R/ATP5F1B/AKT1/CASP3/CYP1B1/VEGFA/ERBB2/IL2/SERPINE1/COL1A1/PPARA/IRF1/ERBB3/RASA1/JAK3/JAK2/LGALS3/PTPN2/CEBPB | 19 |
| GO:0042113 | B cell activation | 19/412 | 6.42E-05 | 0.000181009 | IGHG1/BCL2/BAX/CASP3/VCAM1/AHR/CDKN1A/TP53/CASP8/PRKCB/IL2/CD40LG/JAK3/ADA/PTPN2/TFRC/IL6/IGHG2/PTK2B | 19 |
| GO:0010506 | regulation of autophagy | 19/412 | 0.000129627 | 0.00033692 | ADRA1A/ADRB2/GSK3B/KDR/MET/AKT1/BCL2/CASP3/MAPK8/HMOX1/TP53/HIF1A/HSPB1/IFNG/HK2/GBA/HDAC6/MCL1/ITPR1 | 19 |
| GO:0015849 | organic acid transport | 19/412 | 0.000163922 | 0.000416648 | RXRA/NCOA2/NCOA1/NOS2/PPARG/PPARD/AKT1/ACACA/IL1B/PPARA/FOLR1/FOLR2/SLC46A1/NMUR2/PLA2G2E/AKR1C1/NR1H4/TRPV1/PLA2G1B | 19 |
| GO:0046942 | carboxylic acid transport | 19/412 | 0.000163922 | 0.000416648 | RXRA/NCOA2/NCOA1/NOS2/PPARG/PPARD/AKT1/ACACA/IL1B/PPARA/FOLR1/FOLR2/SLC46A1/NMUR2/PLA2G2E/AKR1C1/NR1H4/TRPV1/PLA2G1B | 19 |
| GO:0007265 | Ras protein signal transduction | 19/412 | 0.005273288 | 0.007880605 | ADRA1A/CDK2/MAPK14/CCNA2/MET/JUN/CDKN1A/RB1/TP53/RAF1/COL3A1/CHUK/RASSF1/RASA1/JAK2/VAV1/F2R/FGF2/OGT | 19 |
| GO:0051346 | negative regulation of hydrolase activity | 19/412 | 0.007907297 | 0.010998871 | PTGS2/GSK3B/DPEP1/IKBKB/AKT1/SLPI/VEGFA/MMP9/TP53/POR/RAF1/BIRC5/NOS3/SERPINE1/SERPINA6/LGALS3/EGLN1/NEIL1/CSNK2A1 | 19 |
| GO:0046209 | nitric oxide metabolic process | 18/412 | 1.71E-13 | 4.61E-12 | PTGS2/NOS2/AKT1/ICAM1/CYP1B1/INSR/POR/CAV1/IL1B/NOS3/IFNG/NQO1/JAK2/MMP8/HSP90AA1/RORA/TRPV1/PTK2B | 18 |
| GO:2001057 | reactive nitrogen species metabolic process | 18/412 | 3.32E-13 | 8.32E-12 | PTGS2/NOS2/AKT1/ICAM1/CYP1B1/INSR/POR/CAV1/IL1B/NOS3/IFNG/NQO1/JAK2/MMP8/HSP90AA1/RORA/TRPV1/PTK2B | 18 |
| GO:0043279 | response to alkaloid | 18/412 | 4.52E-11 | 7.53E-10 | OPRM1/PPARG/CCNA2/RELA/HTR3A/DRD1/SLC6A3/CASP3/ICAM1/GSTM2/BCL2L1/HSPA5/ADA/GRIN1/OPRK1/CACNA1S/TRPV1/PTK2B | 18 |
| GO:0006690 | icosanoid metabolic process | 18/412 | 6.14E-11 | 9.89E-10 | PTGS2/PTGS1/DPEP1/CYP1A2/CYP1A1/CYP1B1/ALOX5/AKR1C3/IL1B/PON1/CYP2C19/TBXAS1/PTGES/CES2/HPGDS/AKR1C2/CBR1/PLA2G1B | 18 |
| GO:0097553 | calcium ion transmembrane import into cytosol | 18/412 | 1.93E-09 | 2.06E-08 | DRD1/BAX/GSTM2/CXCL11/CXCL10/F2R/FGF2/TRPA1/ITPR1/GRIN1/GRIN2A/GRIN2B/GRIN2C/GRIN2D/CACNA1C/TRPV1/GPER1/PTK2B | 18 |
| GO:0009411 | response to UV | 18/412 | 2.17E-09 | 2.27E-08 | PTGS2/CHEK1/RELA/AKT1/BCL2/BAX/CASP3/MAPK8/EGFR/CCND1/CDKN1A/CASP9/TP53/MYC/PARP1/TYR/FEN1/RUVBL2 | 18 |
| GO:0043524 | negative regulation of neuron apoptotic process | 18/412 | 4.27E-09 | 4.11E-08 | JUN/BCL2/BAX/HMOX1/BCL2L1/SOD1/HIF1A/CCL2/ERBB3/RASA1/JAK2/F2R/GABRA5/GABRB2/GABRB3/GRIN1/CEBPB/PTK2B | 18 |
| GO:0008203 | cholesterol metabolic process | 18/412 | 5.91E-09 | 5.52E-08 | RXRA/PPARD/POR/SOD1/ACACA/PON1/NPC1L1/GBA/HMGCR/FGF1/SOAT1/SOAT2/SULT2B1/LSS/NR1H4/AKR1D1/CES1/G6PD | 18 |
| GO:1990845 | adaptive thermogenesis | 18/412 | 9.01E-09 | 8.15E-08 | ACHE/ADRB2/ADRB1/IL4R/VEGFA/RB1/CAV1/GJA1/HSF1/JAK2/ADRB3/KDM6B/OGT/TRPM8/TRPV1/CEBPB/ACOT13/ESRRG | 18 |
| GO:2001236 | regulation of extrinsic apoptotic signaling pathway | 18/412 | 9.99E-09 | 8.94E-08 | AR/RELA/AKT1/BCL2/HMOX1/ICAM1/GSTP1/BCL2L1/CASP8/RAF1/CAV1/IL1B/NOS3/SERPINE1/IL1A/MCL1/LGALS3/GPER1 | 18 |
| GO:0051100 | negative regulation of binding | 18/412 | 3.90E-08 | 3.03E-07 | ADRB2/GSK3B/MET/JUN/AKT1/BAX/MAPK8/HMOX1/PPP3CA/SLPI/NFKBIA/HSPA5/CAV1/PPARA/E2F1/JAK2/ACTB/RUVBL2 | 18 |
| GO:0006937 | regulation of muscle contraction | 18/412 | 4.68E-08 | 3.51E-07 | PTGS2/CHRM3/SCN5A/ADRA1A/CHRM2/ADRB2/ADRA1B/ADRA2C/GSTM2/SOD1/CAV1/ATP2A1/ADA/F2R/NMUR2/CACNA1C/GPER1/PIK3CG | 18 |
| GO:0010821 | regulation of mitochondrion organization | 18/412 | 1.22E-07 | 8.03E-07 | GSK3B/KDR/AKT1/BCL2/BAX/MAPK8/BCL2L1/MMP9/TP53/CASP8/HIF1A/E2F1/NPEPPS/HK2/GBA/HDAC6/FXN/GPER1 | 18 |
| GO:0002064 | epithelial cell development | 18/412 | 8.34E-07 | 4.51E-06 | PGR/AR/ESR1/GSK3B/MET/IKBKB/ICAM1/VEGFA/CDKN1A/SOD1/HIF1A/GJA1/IL1B/TYMS/CDK6/RARA/RARB/RARG | 18 |
| GO:0006006 | glucose metabolic process | 18/412 | 9.60E-07 | 5.07E-06 | NCOA2/MAPK14/GSK3B/PPARD/AKT1/INSR/TP53/PPARA/IGFBP3/IGF2/HK2/RORC/PTPN2/OGT/GCK/RORA/ESRRB/G6PD | 18 |
| GO:0030595 | leukocyte chemotaxis | 18/412 | 2.60E-06 | 1.19E-05 | MAPK14/F7/VEGFA/IL1B/CCL2/CXCL8/SERPINE1/CXCL11/CXCL2/CXCL10/CYP19A1/VAV1/CCR1/LGALS3/IL6/PIK3CG/PTK2B/PLA2G1B | 18 |
| GO:0048738 | cardiac muscle tissue development | 18/412 | 4.54E-06 | 1.89E-05 | RXRA/ADRA1A/MAPK14/CDK1/VEGFA/MAPK1/GJA1/CCNB1/PPARA/ERBB3/FGF2/KDM6B/NOX4/TGFBR2/PIM1/RARA/RARB/G6PD | 18 |
| GO:0042593 | glucose homeostasis | 18/412 | 7.25E-06 | 2.81E-05 | ADRA1B/PPARG/AKT1/ICAM1/SLC2A4/INSR/PPP3CA/RAF1/HIF1A/HK2/HMGCR/FFAR1/NOX4/PTPN2/GCK/OPRK1/NR1H4/GPER1 | 18 |
| GO:0033500 | carbohydrate homeostasis | 18/412 | 7.68E-06 | 2.96E-05 | ADRA1B/PPARG/AKT1/ICAM1/SLC2A4/INSR/PPP3CA/RAF1/HIF1A/HK2/HMGCR/FFAR1/NOX4/PTPN2/GCK/OPRK1/NR1H4/GPER1 | 18 |
| GO:0021537 | telencephalon development | 18/412 | 1.27E-05 | 4.58E-05 | SCN5A/NCOA1/GSK3B/DRD1/HSD3B2/HSD3B1/BAX/CASP3/EGFR/HIF1A/COL3A1/HDAC1/KDM6B/SRD5A1/GRIN1/CDK6/RARA/RARB | 18 |
| GO:0007249 | I-kappaB kinase/NF-kappaB signaling | 18/412 | 3.20E-05 | 0.000101193 | ESR1/RELA/IKBKB/AKT1/STAT1/HMOX1/GSTP1/NFKBIA/CASP8/GJA1/IL1B/PRKCB/HSPB1/CHUK/F2R/HDAC1/RORA/NR1H4 | 18 |
| GO:0046390 | ribose phosphate biosynthetic process | 18/412 | 0.000130134 | 0.000337891 | ATP5F1B/INSR/EIF6/TP53/HIF1A/ACACA/IFNG/PARP1/PPARA/HK2/ADK/OGT/GCK/PAPSS1/ESRRB/NPPB/COX5B/G6PD | 18 |
| GO:0045930 | negative regulation of mitotic cell cycle | 18/412 | 0.00055197 | 0.001175237 | CDK2/CHEK1/BCL2/BAX/CDK1/PSMD3/EGFR/CCND1/BCL2L1/CDKN1A/RB1/TP53/CCL2/CCNB1/TOP2A/CHEK2/E2F1/CDK6 | 18 |
| GO:0033044 | regulation of chromosome organization | 18/412 | 0.000633152 | 0.00131707 | CHEK1/MAPK8/VEGFA/MAPK1/RB1/TP53/MYC/IL1B/CCNB1/TOP2A/PARP1/IGF2/RPS6KA5/HDAC8/PHF8/OGT/FEN1/RUVBL2 | 18 |
| GO:0051260 | protein homooligomerization | 18/412 | 0.000854183 | 0.001701149 | RXRA/SLC6A4/BAX/HMOX1/TP53/ACACA/CAV1/HSF1/GBA/GLRA1/CDA/TK1/TRPA1/TRPM8/AKR1C1/SIGMAR1/TRPV1/ACOT13 | 18 |
| GO:0001654 | eye development | 18/412 | 0.001211296 | 0.002281616 | RXRA/ACHE/JUN/BCL2/BAX/CYP1A1/CYP1B1/EGFR/VEGFA/HIF1A/GJA1/HDAC1/TGFBR2/CACNA1C/RHO/RARA/RARB/RARG | 18 |
| GO:0150063 | visual system development | 18/412 | 0.001369397 | 0.002509421 | RXRA/ACHE/JUN/BCL2/BAX/CYP1A1/CYP1B1/EGFR/VEGFA/HIF1A/GJA1/HDAC1/TGFBR2/CACNA1C/RHO/RARA/RARB/RARG | 18 |
| GO:0048880 | sensory system development | 18/412 | 0.00159136 | 0.002831099 | RXRA/ACHE/JUN/BCL2/BAX/CYP1A1/CYP1B1/EGFR/VEGFA/HIF1A/GJA1/HDAC1/TGFBR2/CACNA1C/RHO/RARA/RARB/RARG | 18 |
| GO:0045089 | positive regulation of innate immune response | 18/412 | 0.002127515 | 0.003619605 | ESR1/RELA/IKBKB/PSMD3/NFKBIA/CASP8/RAF1/CAV1/CHUK/IRF1/RPS6KA5/VAV1/CTSK/CTSS/NFKB1/CLEC4E/NR1H4/HCK | 18 |
| GO:0040013 | negative regulation of locomotion | 18/412 | 0.003211287 | 0.005151829 | PPARG/PPARD/DPEP1/AKT1/BCL2/HMOX1/CYP1B1/GSTP1/CCL2/SERPINE1/NFE2L2/COL3A1/IGFBP3/CYP19A1/ADA/FGF2/PTPN2/CSNK2B | 18 |
| GO:0006809 | nitric oxide biosynthetic process | 17/412 | 7.35E-13 | 1.71E-11 | PTGS2/NOS2/AKT1/ICAM1/CYP1B1/INSR/CAV1/IL1B/NOS3/IFNG/NQO1/JAK2/MMP8/HSP90AA1/RORA/TRPV1/PTK2B | 17 |
| GO:0019233 | sensory perception of pain | 17/412 | 1.19E-10 | 1.82E-09 | PTGS2/OPRM1/ADRA2C/OPRD1/MAPK1/CCL2/F2R/BACE1/NMUR2/TRPA1/COMT/GRIN1/GRIN2A/GRIN2D/OPRK1/CACNB3/TRPV1 | 17 |
| GO:0033559 | unsaturated fatty acid metabolic process | 17/412 | 2.97E-10 | 4.13E-09 | PTGS2/PTGS1/CYP1A2/CYP1A1/CYP1B1/ALOX5/GSTP1/GSTM2/AKR1C3/IL1B/CYP2C19/TBXAS1/PTGES/CES2/HPGDS/AKR1C2/CBR1 | 17 |
| GO:1904019 | epithelial cell apoptotic process | 17/412 | 3.44E-10 | 4.72E-09 | KDR/HMOX1/ICAM1/AKR1C3/BCL2L1/RB1/CCL2/SERPINE1/NFE2L2/E2F1/E2F2/CD40LG/JAK2/TGFBR2/IL6/GPER1/PIK3CG | 17 |
| GO:0031644 | regulation of neurological system process | 17/412 | 9.41E-09 | 8.45E-08 | ADRB2/OPRM1/ADRA2C/OPRD1/NOS3/GBA/F2R/GLRA1/TYMP/NMUR2/CTSC/COMT/GRIN1/GRIN2D/OPRK1/CACNB3/PTK2B | 17 |
| GO:0007586 | digestion | 17/412 | 1.17E-08 | 1.04E-07 | CHRM3/CHRM1/PRSS1/CHRM5/PTGER3/NPC1L1/SLC5A1/SLC46A1/TYMP/SOAT2/VDR/AKR1C1/AKR1C2/OPRK1/AKR1D1/TRPV1/CTRB1 | 17 |
| GO:0106106 | cold-induced thermogenesis | 17/412 | 1.81E-08 | 1.55E-07 | ACHE/ADRB2/ADRB1/IL4R/VEGFA/RB1/CAV1/GJA1/HSF1/JAK2/ADRB3/KDM6B/OGT/TRPM8/CEBPB/ACOT13/ESRRG | 17 |
| GO:0120161 | regulation of cold-induced thermogenesis | 17/412 | 1.81E-08 | 1.55E-07 | ACHE/ADRB2/ADRB1/IL4R/VEGFA/RB1/CAV1/GJA1/HSF1/JAK2/ADRB3/KDM6B/OGT/TRPM8/CEBPB/ACOT13/ESRRG | 17 |
| GO:0002262 | myeloid cell homeostasis | 17/412 | 2.74E-08 | 2.24E-07 | MAPK14/BAX/CASP3/STAT1/HMOX1/VEGFA/RB1/SOD1/HIF1A/JAK3/JAK2/PTPN2/AHSP/IL6/CDK6/MB/G6PD | 17 |
| GO:0071236 | cellular response to antibiotic | 17/412 | 2.74E-08 | 2.24E-07 | RELA/MET/CDK1/CYP1B1/AHR/TP53/HSPA5/NFE2L2/NQO1/HSF1/HDAC6/GLRA1/GLRA2/KDM6B/FXN/IL6/SIGMAR1 | 17 |
| GO:1903169 | regulation of calcium ion transmembrane transport | 17/412 | 4.10E-08 | 3.16E-07 | DRD1/BAX/GSTM2/CXCL11/CXCL10/ATP2A1/F2R/CACNA1C/CACNB1/CACNB2/CACNB3/CACNB4/GPER1/HSPA2/PIK3CG/PTK2B/G6PD | 17 |
| GO:0030168 | platelet activation | 17/412 | 4.99E-08 | 3.69E-07 | ADRA2C/MAPK1/RAF1/PRKCA/PRKCB/NOS3/HSPB1/THBD/COL1A1/COL3A1/CD40LG/VAV1/F2R/ITPR1/IL6/ACTB/PIK3CG | 17 |
| GO:0007259 | JAK-STAT cascade | 17/412 | 6.65E-08 | 4.73E-07 | AKR1B1/STAT1/CYP1B1/IL10RB/EGF/IL6ST/CAV1/CCL2/IL2/IFNG/HSF1/PTPN1/JAK3/JAK2/F2R/PTPN2/IL6 | 17 |
| GO:0043467 | regulation of generation of precursor metabolites and energy | 17/412 | 8.03E-08 | 5.62E-07 | NOS2/GSK3B/AKT1/CDK1/INSR/EIF6/TP53/HIF1A/CCNB1/IFNG/PPARA/IGF2/CISD1/OGT/GCK/ESRRB/COX7A1 | 17 |
| GO:2001242 | regulation of intrinsic apoptotic signaling pathway | 17/412 | 1.52E-07 | 9.75E-07 | PTGS2/AKT1/BCL2/BAX/BCL2L1/MMP9/TP53/SOD1/HIF1A/CAV1/HSPB1/NFE2L2/PARP1/PTPN1/HDAC1/MCL1/PTPN2 | 17 |
| GO:0097696 | STAT cascade | 17/412 | 1.66E-07 | 1.05E-06 | AKR1B1/STAT1/CYP1B1/IL10RB/EGF/IL6ST/CAV1/CCL2/IL2/IFNG/HSF1/PTPN1/JAK3/JAK2/F2R/PTPN2/IL6 | 17 |
| GO:2001257 | regulation of cation channel activity | 17/412 | 4.52E-07 | 2.58E-06 | ADRB2/OPRM1/GRIA2/GSTM2/MMP9/CAV1/CCL2/IFNG/CTSS/GRIN1/GRIN2A/GRIN2B/CACNB1/CACNB2/CACNB3/CACNB4/PTK2B | 17 |
| GO:0071347 | cellular response to interleukin-1 | 17/412 | 4.89E-07 | 2.76E-06 | RELA/IKBKB/ICAM1/HAS2/PSMD3/NFKBIA/HIF1A/IL1B/CCL2/CXCL8/IL1A/CHUK/RPS6KA5/IL6/NFKB1/RORA/CEBPB | 17 |
| GO:0071478 | cellular response to radiation | 17/412 | 1.22E-06 | 6.26E-06 | PTGS2/CHEK1/MAPK14/BAX/BCL2L1/CDKN1A/CASP9/TP53/ELK1/HSPA5/MYC/PARP1/CHEK2/HSF1/NOX4/RUVBL2/RHO | 17 |
| GO:0050731 | positive regulation of peptidyl-tyrosine phosphorylation | 17/412 | 1.31E-06 | 6.63E-06 | ADRA1A/NCF1/ICAM1/VEGFA/EGF/IL6ST/TP53/IL2/IFNG/HSF1/IGF2/ERBB3/PTPN1/JAK2/NOX4/IL6/PTK2B | 17 |
| GO:0045333 | cellular respiration | 17/412 | 1.41E-06 | 7.04E-06 | NOS2/CDK1/CYP1A2/HIF1A/CCNB1/IFNG/CISD1/FXN/COX4I1/COX5A/COX5B/COX6A2/COX6B1/COX6C/COX7B/COX7C/COX8A | 17 |
| GO:0010952 | positive regulation of peptidase activity | 17/412 | 1.87E-06 | 9.00E-06 | PPARG/MAPK14/BAX/CASP9/CASP8/CAV1/MYC/F3/HSF1/CTSD/PCOLCE/JAK2/F2R/GRIN1/GRIN2A/GRIN2B/GPER1 | 17 |
| GO:0046890 | regulation of lipid biosynthetic process | 17/412 | 2.00E-06 | 9.54E-06 | PTGS2/AKT1/AKR1C3/EIF6/POR/SOD1/ACACA/IL1B/IFNG/HMGCR/FGF1/VDR/NFKB1/LSS/NR1H4/GPER1/CYP27B1 | 17 |
| GO:0050679 | positive regulation of epithelial cell proliferation | 17/412 | 3.44E-06 | 1.50E-05 | AR/SCN5A/KDR/JUN/AKT1/HMOX1/HAS2/EGFR/VEGFA/CCND1/PRKCA/HIF1A/ERBB2/MYC/F3/FGF1/FGF2 | 17 |
| GO:0034284 | response to monosaccharide | 17/412 | 3.68E-06 | 1.58E-05 | PTGS2/PPARD/CASP3/ICAM1/GSTP1/PPP3CA/RAF1/HIF1A/GJA1/HMGCR/NOX4/GCK/TGFBR2/OPRK1/NR1H4/GPER1/PTK2B | 17 |
| GO:0009124 | nucleoside monophosphate biosynthetic process | 17/412 | 3.92E-06 | 1.67E-05 | ATP5F1B/INSR/EIF6/TP53/HIF1A/IFNG/PARP1/PPARA/HK2/ADK/ADA/TYMS/TK1/OGT/GCK/ESRRB/COX5B | 17 |
| GO:0097529 | myeloid leukocyte migration | 17/412 | 4.46E-06 | 1.86E-05 | MAPK14/VEGFA/IL1B/CCL2/CXCL8/SERPINE1/CXCL11/CXCL2/CXCL10/CYP19A1/VAV1/CCR1/LGALS3/IL6/PIK3CG/PTK2B/PLA2G1B | 17 |
| GO:0016051 | carbohydrate biosynthetic process | 17/412 | 5.75E-06 | 2.29E-05 | GSK3B/AKR1B1/AKT1/HAS2/INSR/EGF/PPARA/IGF2/PTPN2/OGT/GCK/ISYNA1/NFKB1/ESRRB/GPER1/PTK2B/G6PD | 17 |
| GO:0019935 | cyclic-nucleotide-mediated signaling | 17/412 | 6.12E-06 | 2.43E-05 | ADRA1A/ADRB2/OPRM1/ADRA1B/ADRB1/ADRA2C/ADRA1D/DRD1/AHR/PRKCA/PTGER3/CXCL11/CXCL10/ADRB3/GPER1/PTK2B/NPPB | 17 |
| GO:0010001 | glial cell differentiation | 17/412 | 7.36E-06 | 2.85E-05 | PPARG/RELA/DRD1/AKT1/CDK1/GSTP1/EGFR/MAPK1/IL6ST/SOD1/ERBB2/IL1B/IFNG/ERBB3/HDAC1/IL6/CDK6 | 17 |
| GO:0034404 | nucleobase-containing small molecule biosynthetic process | 17/412 | 1.18E-05 | 4.29E-05 | INSR/EIF6/TP53/HIF1A/IFNG/PPARA/HK2/ADK/ADA/TYMP/CDA/TK1/OGT/GCK/NEIL1/NEIL2/ESRRB | 17 |
| GO:0051054 | positive regulation of DNA metabolic process | 17/412 | 1.33E-05 | 4.74E-05 | CDK2/JUN/AKT1/BAX/CDK1/EGFR/MAPK1/MYC/IL2/PARP1/HSF1/FGF2/HSP90AA1/NOX4/TFRC/IL6/PTK2B | 17 |
| GO:0010594 | regulation of endothelial cell migration | 17/412 | 1.41E-05 | 4.93E-05 | PTGS2/PPARG/KDR/MET/ATP5F1B/AKT1/HMOX1/VEGFA/PRKCA/HIF1A/NOS3/HSPB1/NFE2L2/FGF1/FGF2/CSNK2B/PTK2B | 17 |
| GO:0007050 | cell cycle arrest | 17/412 | 2.19E-05 | 7.29E-05 | CDK2/BAX/CDK1/CCND1/CDKN1A/RB1/TP53/MYC/CXCL8/CCNB1/IFNG/CHEK2/RASSF1/E2F1/IRF1/CDK6/GPER1 | 17 |
| GO:0043122 | regulation of I-kappaB kinase/NF-kappaB signaling | 17/412 | 2.19E-05 | 7.29E-05 | ESR1/RELA/IKBKB/AKT1/STAT1/HMOX1/GSTP1/CASP8/GJA1/IL1B/PRKCB/HSPB1/CHUK/F2R/HDAC1/RORA/NR1H4 | 17 |
| GO:0038093 | Fc receptor signaling pathway | 17/412 | 2.72E-05 | 8.75E-05 | IGHG1/RELA/JUN/IKBKB/MAPK8/PSMD3/PPP3CA/FOS/MAPK1/CHUK/VAV1/HSP90AA1/NFKB1/CLEC4E/IGHG2/ACTB/HCK | 17 |
| GO:0031334 | positive regulation of protein complex assembly | 17/412 | 0.000102047 | 0.000272224 | ESR1/GSK3B/MET/JUN/BAX/MMP1/ICAM1/MMP3/VEGFA/TP53/IFNG/PARP1/HDAC6/HSP90AA1/ARF1/HCK/PTK2B | 17 |
| GO:0061448 | connective tissue development | 17/412 | 0.000127643 | 0.000332448 | MAPK14/PPARD/RELA/POR/HIF1A/COL1A1/RUNX2/TYMS/RORC/FGF2/CTSK/TGFBR2/NR1H4/ESRRA/RARA/RARB/RARG | 17 |
| GO:0009152 | purine ribonucleotide biosynthetic process | 17/412 | 0.000172862 | 0.000434141 | ATP5F1B/INSR/EIF6/TP53/HIF1A/ACACA/IFNG/PARP1/PPARA/HK2/ADK/OGT/GCK/PAPSS1/ESRRB/NPPB/COX5B | 17 |
| GO:0030100 | regulation of endocytosis | 17/412 | 0.000180348 | 0.000451152 | PPARG/SELE/SLC2A4/PPP3CA/VEGFA/EGF/SOD1/CAV1/IL1B/CCL2/SERPINE1/IFNG/PTPN1/LGALS3/ARF1/TF/HCK | 17 |
| GO:0034976 | response to endoplasmic reticulum stress | 17/412 | 0.000213195 | 0.000520966 | GSK3B/JUN/BCL2/BAX/CCND1/BCL2L1/TP53/HSPA5/CAV1/CCL2/CXCL8/NFE2L2/PTPN1/ATP2A1/PTPN2/ITPR1/CEBPB | 17 |
| GO:0051348 | negative regulation of transferase activity | 17/412 | 0.000213195 | 0.000520966 | PPARG/GSK3B/PKIA/AKT1/CASP3/GSTP1/CDKN1A/RB1/TP53/CAV1/IL1B/HSPB1/IFNG/PTPN1/GBA/HMGCR/PTPN2 | 17 |
| GO:0009260 | ribonucleotide biosynthetic process | 17/412 | 0.000294822 | 0.000686628 | ATP5F1B/INSR/EIF6/TP53/HIF1A/ACACA/IFNG/PARP1/PPARA/HK2/ADK/OGT/GCK/PAPSS1/ESRRB/NPPB/COX5B | 17 |
| GO:0002758 | innate immune response-activating signal transduction | 17/412 | 0.000358583 | 0.000809074 | ESR1/RELA/IKBKB/PSMD3/NFKBIA/CASP8/RAF1/CAV1/CHUK/IRF1/RPS6KA5/CTSK/CTSS/NFKB1/CLEC4E/NR1H4/HCK | 17 |
| GO:0006898 | receptor-mediated endocytosis | 17/412 | 0.00069688 | 0.001434372 | ACHE/ADRB2/SELE/INSR/VEGFA/EGF/CAV1/CXCL8/SERPINE1/FOLR1/FOLR2/HSP90AA1/ARF1/HBA1/TF/TFRC/TGFBR2 | 17 |
| GO:0002218 | activation of innate immune response | 17/412 | 0.000773993 | 0.001570051 | ESR1/RELA/IKBKB/PSMD3/NFKBIA/CASP8/RAF1/CAV1/CHUK/IRF1/RPS6KA5/CTSK/CTSS/NFKB1/CLEC4E/NR1H4/HCK | 17 |
| GO:0016485 | protein processing | 17/412 | 0.001050598 | 0.002022565 | IGHG1/F7/CASP3/PLAU/CASP8/F3/IL1B/PLAT/SERPINE1/PARP1/PSENEN/CTSS/OGT/FXN/GRIN2A/IGHG2/CES1 | 17 |
| GO:0030336 | negative regulation of cell migration | 17/412 | 0.001278401 | 0.002374448 | PPARG/PPARD/DPEP1/AKT1/BCL2/HMOX1/CYP1B1/GSTP1/CCL2/SERPINE1/NFE2L2/COL3A1/IGFBP3/CYP19A1/ADA/FGF2/CSNK2B | 17 |
| GO:2000146 | negative regulation of cell motility | 17/412 | 0.002037524 | 0.003487744 | PPARG/PPARD/DPEP1/AKT1/BCL2/HMOX1/CYP1B1/GSTP1/CCL2/SERPINE1/NFE2L2/COL3A1/IGFBP3/CYP19A1/ADA/FGF2/CSNK2B | 17 |
| GO:0051271 | negative regulation of cellular component movement | 17/412 | 0.005352118 | 0.007993693 | PPARG/PPARD/DPEP1/AKT1/BCL2/HMOX1/CYP1B1/GSTP1/CCL2/SERPINE1/NFE2L2/COL3A1/IGFBP3/CYP19A1/ADA/FGF2/CSNK2B | 17 |
| GO:0051604 | protein maturation | 17/412 | 0.007371987 | 0.010385513 | IGHG1/F7/CASP3/PLAU/CASP8/F3/IL1B/PLAT/SERPINE1/PARP1/PSENEN/CTSS/OGT/FXN/GRIN2A/IGHG2/CES1 | 17 |
| GO:0046686 | response to cadmium ion | 16/412 | 3.36E-13 | 8.35E-12 | NCF1/JUN/AKT1/MAPK8/CDK1/HMOX1/CYP1A2/AKR1C3/EGFR/FOS/MMP9/MAPK1/SOD1/HSF1/CHUK/CYB5A | 16 |
| GO:0043627 | response to estrogen | 16/412 | 3.96E-12 | 8.08E-11 | ESR1/PPARG/F7/IL4R/HMOX1/CCND1/MAPK1/CAV1/CA2/GBA/SRD5A1/COMT/TGFBR2/OPRK1/RARA/CYP27B1 | 16 |
| GO:1901216 | positive regulation of neuron death | 16/412 | 2.26E-10 | 3.27E-09 | GSK3B/JUN/BAX/CASP3/FOS/CASP9/TP53/ELK1/CASP8/IFNG/NQO1/PARP1/NQO2/BACE1/MCL1/GRIN2B | 16 |
| GO:2001237 | negative regulation of extrinsic apoptotic signaling pathway | 16/412 | 1.07E-09 | 1.24E-08 | AR/RELA/AKT1/BCL2/HMOX1/ICAM1/GSTP1/BCL2L1/CASP8/RAF1/IL1B/NOS3/SERPINE1/IL1A/MCL1/LGALS3 | 16 |
| GO:0051341 | regulation of oxidoreductase activity | 16/412 | 1.64E-09 | 1.82E-08 | AKT1/EGFR/POR/HIF1A/CAV1/IL1B/NOS3/IFNG/DHFR/HDAC6/HSP90AA1/VDR/FXN/NFKB1/PTK2B/CYP27B1 | 16 |
| GO:0099565 | chemical synaptic transmission, postsynaptic | 16/412 | 1.89E-09 | 2.03E-08 | ADRB2/OPRM1/GSK3B/AKT1/PPP3CA/GLRA1/GLRA2/GABRB3/GRIN1/GRIN2A/GRIN2B/GRIN2C/GRIN2D/CACNB3/TRPV1/PTK2B | 16 |
| GO:0006939 | smooth muscle contraction | 16/412 | 2.49E-09 | 2.57E-08 | PTGS2/CHRM3/ADRA1A/CHRM2/ADRB2/ADRA1B/ADRA2C/DRD1/SOD1/CAV1/PTGER3/ADA/F2R/NMUR2/TRPV1/GPER1 | 16 |
| GO:0043200 | response to amino acid | 16/412 | 3.72E-09 | 3.65E-08 | F7/RELA/CASP3/ICAM1/GSTP1/EGFR/BCL2L1/MMP2/COL1A1/COL3A1/HSF1/CHUK/GLRA1/GLRA2/GRIN1/CEBPB | 16 |
| GO:0006720 | isoprenoid metabolic process | 16/412 | 7.52E-08 | 5.28E-07 | ADH1B/ADH1C/PPARD/AKR1B1/CYP3A4/CYP1A2/CYP1A1/CYP1B1/AKR1C3/EGFR/HMGCR/CYP2C19/SRD5A1/AKR1C1/LSS/RHO | 16 |
| GO:0010675 | regulation of cellular carbohydrate metabolic process | 16/412 | 1.02E-07 | 6.81E-07 | NCOA2/GSK3B/AKT1/INSR/TP53/PPARA/IGFBP3/IGF2/RORC/PTPN2/OGT/GCK/RORA/ESRRB/GPER1/PTK2B | 16 |
| GO:0006140 | regulation of nucleotide metabolic process | 16/412 | 1.50E-07 | 9.69E-07 | NOS2/CDK1/INSR/EIF6/TP53/HIF1A/NOS3/CCNB1/IFNG/PARP1/PPARA/CDA/OGT/GCK/ESRRB/COX7A1 | 16 |
| GO:0035051 | cardiocyte differentiation | 16/412 | 9.44E-07 | 5.01E-06 | RXRA/ADRA1A/CDK1/VCAM1/EGFR/VEGFA/MAPK1/CCNB1/PPARA/FOLR1/KDM6B/NOX4/GPER1/RARA/RARB/G6PD | 16 |
| GO:0050728 | negative regulation of inflammatory response | 16/412 | 1.11E-06 | 5.77E-06 | PPARG/PPARD/TNFAIP6/GSTP1/RB1/SOD1/IL2/PPARA/CYP19A1/ADA/GBA/PTPN2/NFKB1/RORA/NR1H4/GPER1 | 16 |
| GO:0021543 | pallium development | 16/412 | 1.40E-06 | 7.04E-06 | NCOA1/GSK3B/DRD1/HSD3B2/HSD3B1/BAX/CASP3/EGFR/HIF1A/COL3A1/HDAC1/KDM6B/SRD5A1/GRIN1/CDK6/RARA | 16 |
| GO:0010469 | regulation of signaling receptor activity | 16/412 | 1.51E-06 | 7.47E-06 | ADRB2/OPRM1/ESR2/GRIA2/NCF1/ADRA2C/PLAU/EGF/CCL2/SERPINE1/IFNG/HDAC6/GRIN1/GRIN2A/GRIN2B/PTK2B | 16 |
| GO:0051099 | positive regulation of binding | 16/412 | 2.37E-06 | 1.10E-05 | PPARG/GSK3B/MET/MMP9/EGF/RB1/CAV1/IFNG/PARP1/HSF1/PON1/JAK2/MMP8/HDAC8/LGALS3/RARA | 16 |
| GO:2001235 | positive regulation of apoptotic signaling pathway | 16/412 | 2.37E-06 | 1.10E-05 | GSK3B/BCL2/BAX/MAPK8/BCL2L1/MMP9/TP53/CASP8/SOD1/CAV1/E2F1/JAK2/MCL1/PTPN2/CTSC/GPER1 | 16 |
| GO:0002285 | lymphocyte activation involved in immune response | 16/412 | 2.74E-06 | 1.24E-05 | IL4R/ICAM1/TP53/IL2/IFNG/CD40LG/JAK3/ADA/RORC/LGALS3/TFRC/IL6/CLEC4E/RORA/PTK2B/RARA | 16 |
| GO:2000045 | regulation of G1/S transition of mitotic cell cycle | 16/412 | 3.40E-06 | 1.49E-05 | CDK2/AKT1/BCL2/BAX/CDK1/CYP1A1/EGFR/CCND1/CDKN1A/RB1/TP53/CCL2/CCNB1/CHEK2/E2F1/CDK6 | 16 |
| GO:1903708 | positive regulation of hemopoiesis | 16/412 | 3.64E-06 | 1.57E-05 | MAPK14/JUN/IL4R/STAT1/FOS/RB1/CASP8/PRKCA/HIF1A/IL2/IFNG/CA2/ADA/CCR1/TGFBR2/RARA | 16 |
| GO:0043112 | receptor metabolic process | 16/412 | 5.88E-06 | 2.34E-05 | ACHE/PPARG/SELE/VEGFA/EGF/HIF1A/CAV1/CXCL8/IFNG/PPARA/PTPN1/JAK2/HDAC6/HDAC1/ARF1/TFRC | 16 |
| GO:0002685 | regulation of leukocyte migration | 16/412 | 7.65E-06 | 2.95E-05 | MAPK14/F7/AKT1/HMOX1/ICAM1/VEGFA/CCL2/CXCL8/SERPINE1/CXCL10/CYP19A1/ADA/CCR1/LGALS3/IL6/PTK2B | 16 |
| GO:0009746 | response to hexose | 16/412 | 1.12E-05 | 4.12E-05 | PTGS2/PPARD/CASP3/ICAM1/PPP3CA/RAF1/HIF1A/GJA1/HMGCR/NOX4/GCK/TGFBR2/OPRK1/NR1H4/GPER1/PTK2B | 16 |
| GO:1902806 | regulation of cell cycle G1/S phase transition | 16/412 | 1.12E-05 | 4.12E-05 | CDK2/AKT1/BCL2/BAX/CDK1/CYP1A1/EGFR/CCND1/CDKN1A/RB1/TP53/CCL2/CCNB1/CHEK2/E2F1/CDK6 | 16 |
| GO:0032388 | positive regulation of intracellular transport | 16/412 | 2.43E-05 | 7.99E-05 | PTGS2/CHRM1/MAPK14/GSK3B/IL4R/MAPK1/TP53/ERBB2/IL1B/IFNG/NPEPPS/ATP2A1/ARF1/CACNA1D/CACNB2/CACNB3 | 16 |
| GO:0070374 | positive regulation of ERK1 and ERK2 cascade | 16/412 | 2.43E-05 | 7.99E-05 | ADRA1A/OPRM1/KDR/JUN/ICAM1/EGFR/PRKCA/CCL2/NQO2/CCR1/F2R/HMGCR/FGF2/NOX4/GPER1/PTK2B | 16 |
| GO:0043393 | regulation of protein binding | 16/412 | 2.72E-05 | 8.77E-05 | ADRB2/GSK3B/MET/AKT1/BCL2/BAX/MAPK8/SLPI/MMP9/HSPA5/CAV1/PPARA/HSF1/LGALS3/ACTB/RUVBL2 | 16 |
| GO:0031348 | negative regulation of defense response | 16/412 | 8.68E-05 | 0.00023878 | PPARG/PPARD/TNFAIP6/GSTP1/RB1/SOD1/IL2/PPARA/CYP19A1/ADA/GBA/PTPN2/NFKB1/RORA/NR1H4/GPER1 | 16 |
| GO:0072331 | signal transduction by p53 class mediator | 16/412 | 0.000308473 | 0.000711871 | CDK2/CHEK1/MAPK14/AKT1/BCL2/BAX/CDK1/CDKN1A/TP53/CCNB1/CHEK2/E2F1/E2F2/HDAC1/CSNK2A1/CSNK2B | 16 |
| GO:0021700 | developmental maturation | 16/412 | 0.000606868 | 0.001274967 | PGR/PPARG/BCL2/VEGFA/CDKN1A/MMP2/RB1/HIF1A/GJA1/CCNB1/RUNX2/TYMS/PAEP/GRIN1/PTK2B/G6PD | 16 |
| GO:0043010 | camera-type eye development | 16/412 | 0.00173721 | 0.003060463 | RXRA/ACHE/JUN/BAX/CYP1A1/CYP1B1/EGFR/VEGFA/HIF1A/GJA1/HDAC1/TGFBR2/CACNA1C/RHO/RARA/RARG | 16 |
| GO:0050851 | antigen receptor-mediated signaling pathway | 16/412 | 0.001852697 | 0.003232426 | IGHG1/RELA/IKBKB/BCL2/BAX/PSMD3/MAPK1/PRKCB/CHUK/ADA/LGALS3/PTPN2/NFKB1/CACNA1F/CACNB3/IGHG2 | 16 |
| GO:0031589 | cell-substrate adhesion | 16/412 | 0.005597629 | 0.008325992 | GSK3B/PPARD/KDR/BCL2/VCAM1/HAS2/VEGFA/PLAU/SERPINE1/COL1A1/COL3A1/RASA1/JAK2/HPSE/CDK6/PTK2B | 16 |
| GO:0010948 | negative regulation of cell cycle process | 16/412 | 0.006712679 | 0.009633603 | CDK2/CHEK1/BCL2/BAX/CDK1/PSMD3/CCND1/CDKN1A/RB1/TP53/CCL2/CCNB1/CHEK2/E2F1/CDK6/GPER1 | 16 |
| GO:0045861 | negative regulation of proteolysis | 16/412 | 0.007062199 | 0.010016359 | PTGS2/DPEP1/AKT1/SLPI/VEGFA/MMP9/TP53/POR/RAF1/BIRC5/PLAT/SERPINE1/SERPINA6/HDAC6/OGT/CSNK2A1 | 16 |
| GO:0043900 | regulation of multi-organism process | 16/412 | 0.018430437 | 0.022760051 | NOS2/JUN/BCL2/STAT1/SLPI/PRKCA/CAV1/IL1B/CXCL8/IFNG/TOP2A/ADA/HDAC1/GLRA1/HPGDS/PAEP | 16 |
| GO:0002697 | regulation of immune effector process | 16/412 | 0.048393974 | 0.048443324 | IGHG1/MAPK14/IL4R/STAT1/HMOX1/IL1B/IL2/IFNG/CD40LG/JAK3/VAV1/LGALS3/TFRC/IL6/IGHG2/RARA | 16 |
| GO:0071242 | cellular response to ammonium ion | 15/412 | 1.70E-11 | 3.07E-10 | CHRM3/CHRM1/CHRM2/OPRM1/GABRA1/CCNA2/DRD1/CHRM5/CHRM4/CASP3/MAPK1/GABRG2/GABRB1/GABRB2/GABRB3 | 15 |
| GO:0048145 | regulation of fibroblast proliferation | 15/412 | 3.45E-10 | 4.72E-09 | ESR1/PPARG/CCNA2/JUN/BAX/GSTP1/EGFR/CDKN1A/TP53/MYC/CCNB1/E2F1/FTH1/CDK6/PLA2G1B | 15 |
| GO:0048144 | fibroblast proliferation | 15/412 | 4.11E-10 | 5.48E-09 | ESR1/PPARG/CCNA2/JUN/BAX/GSTP1/EGFR/CDKN1A/TP53/MYC/CCNB1/E2F1/FTH1/CDK6/PLA2G1B | 15 |
| GO:1901655 | cellular response to ketone | 15/412 | 1.81E-09 | 1.97E-08 | AR/PPARG/AKR1B1/AKT1/ICAM1/AHR/AKR1C3/EGFR/CASP9/ELK1/ACACA/SPP1/NR3C1/SRD5A1/AKR1C2 | 15 |
| GO:0051591 | response to cAMP | 15/412 | 3.31E-09 | 3.27E-08 | RELA/SLC6A3/JUN/STAT1/AHR/FOS/HSPA5/DUOX2/THBD/COL1A1/TYR/NOX4/SRD5A1/PIK3CG/PTK2B | 15 |
| GO:0120162 | positive regulation of cold-induced thermogenesis | 15/412 | 3.31E-09 | 3.27E-08 | ACHE/ADRB2/ADRB1/IL4R/VEGFA/CAV1/GJA1/HSF1/JAK2/ADRB3/KDM6B/OGT/TRPM8/CEBPB/ESRRG | 15 |
| GO:0060079 | excitatory postsynaptic potential | 15/412 | 5.10E-09 | 4.87E-08 | ADRB2/OPRM1/GSK3B/AKT1/PPP3CA/GLRA1/GLRA2/GRIN1/GRIN2A/GRIN2B/GRIN2C/GRIN2D/CACNB3/TRPV1/PTK2B | 15 |
| GO:0048661 | positive regulation of smooth muscle cell proliferation | 15/412 | 5.87E-09 | 5.50E-08 | PTGS2/AKR1B1/JUN/AKT1/STAT1/HMOX1/EGFR/MMP2/MMP9/JAK2/NQO2/HMGCR/FGF2/TGFBR2/IL6 | 15 |
| GO:0001676 | long-chain fatty acid metabolic process | 15/412 | 1.71E-08 | 1.47E-07 | PTGS2/PTGS1/CYP3A4/CYP1A2/CYP1A1/CYP1B1/ALOX5/GSTP1/GSTM2/AKR1C3/CYP2C19/TBXAS1/PTGES/HPGDS/CBR1 | 15 |
| GO:0046620 | regulation of organ growth | 15/412 | 2.81E-08 | 2.27E-07 | MAPK14/SLC6A4/AKT1/CDK1/MAPK1/POR/SOD1/GJA1/CCNB1/PPARA/FGF2/FXN/TGFBR2/PIM1/G6PD | 15 |
| GO:0046660 | female sex differentiation | 15/412 | 3.57E-08 | 2.82E-07 | PGR/ESR1/BCL2/BAX/CASP3/ICAM1/INSR/VEGFA/BCL2L1/SOD1/HSPA5/NOS3/CYP19A1/SRD5A1/CEBPB | 15 |
| GO:0010822 | positive regulation of mitochondrion organization | 15/412 | 4.52E-08 | 3.41E-07 | GSK3B/KDR/BCL2/BAX/MAPK8/MMP9/TP53/CASP8/HIF1A/E2F1/NPEPPS/HK2/GBA/HDAC6/GPER1 | 15 |
| GO:0006721 | terpenoid metabolic process | 15/412 | 6.37E-08 | 4.55E-07 | ADH1B/ADH1C/PPARD/AKR1B1/CYP3A4/CYP1A2/CYP1A1/CYP1B1/AKR1C3/EGFR/CYP2C19/SRD5A1/AKR1C1/LSS/RHO | 15 |
| GO:0008637 | apoptotic mitochondrial changes | 15/412 | 9.92E-08 | 6.66E-07 | GSK3B/JUN/AKT1/BCL2/BAX/MAPK8/BCL2L1/MMP9/TP53/CASP8/E2F1/HK2/ATP2A1/FXN/GPER1 | 15 |
| GO:0019218 | regulation of steroid metabolic process | 15/412 | 9.92E-08 | 6.66E-07 | AKR1C3/POR/SOD1/ACACA/IL1B/IFNG/HMGCR/RORC/FGF1/VDR/NFKB1/LSS/RORA/NR1H4/CYP27B1 | 15 |
| GO:0051101 | regulation of DNA binding | 15/412 | 9.92E-08 | 6.66E-07 | PPARG/JUN/MAPK8/HMOX1/MMP9/EGF/RB1/NFKBIA/IFNG/PARP1/E2F1/JAK2/MMP8/HDAC8/CSNK2B | 15 |
| GO:0010595 | positive regulation of endothelial cell migration | 15/412 | 1.52E-07 | 9.75E-07 | PTGS2/KDR/MET/ATP5F1B/AKT1/HMOX1/VEGFA/PRKCA/HIF1A/NOS3/HSPB1/NFE2L2/FGF1/FGF2/PTK2B | 15 |
| GO:0001889 | liver development | 15/412 | 3.07E-07 | 1.83E-06 | RELA/MET/JUN/HMOX1/CYP1A1/EGFR/CCND1/ADA/TYMS/FPGS/SRD5A1/CEBPB/CSNK2B/RARA/RARB | 15 |
| GO:0034605 | cellular response to heat | 15/412 | 3.72E-07 | 2.18E-06 | PTGS2/HSP90AA2P/GSK3B/HMOX1/CDKN1A/MAPK1/HSPA5/IL1A/HSF1/CXCL10/HSPA8/FGF1/HSP90AA1/TRPV1/HSPA2 | 15 |
