# Supplementary material for: Mechanism of drug-pairs Astragalus Mongholicus–Largehead Atractylodes on treating knee osteoarthritis investigated by GEO gene chip with network pharmacology and molecular docking
Source: Medicine (Baltimore). 2024 Jul 5;103(27):e38699. doi: 10.1097/MD.0000000000038699 (PMC11224889; doi:10.1097/MD.0000000000038699)
Supplement: Supplementary file 13 [file medi-103-e38699-s013.doc]

# Appendix 13

**Core targets-Signal pathways of KEGG enrichment analysis**

**Table S13. Core targets-Signal pathways of KEGG enrichment analysis.**

| ID | Description | GeneRatio | pvalue | qvalue | geneID | Count |
| --- | --- | --- | --- | --- | --- | --- |
| hsa05022 | Pathways of neurodegeneration - multiple diseases | 57/359 | 2.58E-12 | 1.17E-11 | PTGS2/CHRM3/CHRM1/NOS2/GRIA2/MAPK14/GSK3B/RELA/CHRM5/SLC6A3/ATP5F1B/BCL2/BAX/CASP3/MAPK8/PSMD3/PPP3CA/BCL2L1/CASP9/MAPK1/CASP8/RAF1/SOD1/PRKCA/HSPA5/IL1B/PRKCB/IL1A/ATP2A1/TUBB1/NOX4/ITPR1/GRIN1/GRIN2A/GRIN2B/GRIN2C/GRIN2D/IL6/NFKB1/SIGMAR1/CACNA1C/CACNA1D/CACNA1F/CACNA1S/CSNK2A1/CSNK2B/UBA1/COX4I1/COX5A/COX5B/COX6A2/COX6B1/COX6C/COX7A1/COX7B/COX7C/COX8A | 57 |
| hsa05010 | Alzheimer disease | 51/359 | 8.10E-13 | 4.00E-12 | PTGS2/CHRM3/CHRM1/NOS2/GSK3B/RELA/CHRM5/ATP5F1B/IKBKB/AKT1/CASP3/MAPK8/PSMD3/INSR/PPP3CA/CASP9/MAPK1/CASP8/RAF1/IL1B/IL1A/CHUK/ATP2A1/TUBB1/PSENEN/BACE1/NOX4/ITPR1/GRIN1/GRIN2A/GRIN2B/GRIN2C/GRIN2D/IL6/NFKB1/CACNA1C/CACNA1D/CACNA1F/CACNA1S/CSNK2A1/CSNK2B/COX4I1/COX5A/COX5B/COX6A2/COX6B1/COX6C/COX7A1/COX7B/COX7C/COX8A | 51 |
| hsa05417 | Lipid and atherosclerosis | 49/359 | 2.52E-22 | 1.43E-20 | RXRA/PPARG/MAPK14/GSK3B/RELA/NCF1/OLR1/RXRB/JUN/IKBKB/AKT1/BCL2/BAX/CASP3/MAPK8/MMP1/CYP1A1/ICAM1/SELE/VCAM1/PPP3CA/MMP3/BCL2L1/FOS/CASP9/MMP9/MAPK1/TP53/NFKBIA/CASP8/PRKCA/HSPA5/IL1B/CCL2/CXCL8/NOS3/NFE2L2/CXCL2/CHUK/CD40LG/JAK2/VAV1/HSPA8/HSP90AA1/ITPR1/IL6/NFKB1/HSPA2/RXRG | 49 |
| hsa04080 | Neuroactive ligand-receptor interaction | 49/359 | 1.15E-13 | 6.25E-13 | CHRM3/CHRM1/ADRA1A/CHRM2/ADRB2/OPRM1/GABRA1/PRSS1/ADRA1B/GRIA2/ADRB1/ADRA2C/ADRA1D/DRD1/CHRM5/CHRM4/OPRD1/PTGER3/ADRB3/NR3C1/F2R/GLRA1/GLRA2/NMUR2/GRM4/GABRA2/GABRA3/GABRA4/GABRA5/GABRA6/GABRG1/GABRG2/GABRG3/GABRB1/GABRB2/GABRB3/GABRD/GABRE/GABRP/GABRQ/GRIN1/GRIN2A/GRIN2B/GRIN2C/GRIN2D/GRIN3A/GRIN3B/OPRK1/TRPV1 | 49 |
| hsa05207 | Chemical carcinogenesis - receptor activation | 47/359 | 6.74E-21 | 1.92E-19 | PGR/AR/RXRA/ADRB2/ESR2/ESR1/RELA/ADRB1/RXRB/JUN/AKT1/BCL2/CYP3A4/CYP1A2/CYP1A1/CYP1B1/AHR/NR1I3/GSTM1/GSTM2/EGFR/VEGFA/CCND1/FOS/MAPK1/EGF/RB1/RAF1/PRKCA/MYC/PRKCB/BIRC5/PPARA/E2F1/JAK2/ADRB3/EPHX1/FGF2/HSP90AA1/UGT2B7/VDR/NFKB1/CACNA1C/CACNA1D/CACNA1F/CACNA1S/RXRG | 47 |
| hsa05208 | Chemical carcinogenesis - reactive oxygen species | 46/359 | 4.07E-19 | 9.26E-18 | MAPK14/RELA/NCF1/MET/JUN/ATP5F1B/IKBKB/AKT1/MAPK8/HMOX1/CYP1A2/CYP1A1/CYP1B1/AHR/GSTM1/GSTM2/AKR1C3/EGFR/VEGFA/FOS/MAPK1/EGF/NFKBIA/RAF1/SOD1/HIF1A/NFE2L2/NQO1/CHUK/PTPN1/EPHX1/NOX4/AKR1C1/AKR1C2/NFKB1/CBR1/COX4I1/COX5A/COX5B/COX6A2/COX6B1/COX6C/COX7A1/COX7B/COX7C/COX8A | 46 |
| hsa04010 | MAPK signaling pathway | 46/359 | 2.91E-14 | 2.06E-13 | MAPK14/RELA/KDR/MET/JUN/IKBKB/AKT1/CASP3/MAPK8/INSR/PPP3CA/EGFR/VEGFA/FOS/MAPK1/EGF/TP53/ELK1/RAF1/PRKCA/ERBB2/MYC/IL1B/PRKCB/HSPB1/IL1A/CHUK/IGF2/ERBB3/RASA1/RPS6KA5/HSPA8/FGF1/FGF2/TGFBR2/NFKB1/NFKB2/CACNA1C/CACNA1D/CACNA1F/CACNA1S/CACNB1/CACNB2/CACNB3/CACNB4/HSPA2 | 46 |
| hsa04151 | PI3K-Akt signaling pathway | 45/359 | 9.36E-11 | 2.80E-10 | CHRM1/RXRA/CHRM2/CDK2/GSK3B/RELA/KDR/MET/IL4R/IKBKB/AKT1/BCL2/INSR/EGFR/VEGFA/CCND1/BCL2L1/CDKN1A/CASP9/MAPK1/EGF/TP53/RAF1/PRKCA/ERBB2/MYC/NOS3/IL2/COL1A1/CHUK/SPP1/IGF2/ERBB3/JAK3/JAK1/JAK2/F2R/FGF1/FGF2/HSP90AA1/MCL1/IL6/NFKB1/CDK6/PIK3CG | 45 |
| hsa05020 | Prion disease | 44/359 | 3.75E-14 | 2.37E-13 | MAPK14/GSK3B/NCF1/ATP5F1B/BAX/CASP3/MAPK8/PSMD3/PPP3CA/CASP9/MAPK1/SOD1/HSPA5/CAV1/IL1B/IL1A/TUBB1/HSPA8/ITPR1/GRIN1/GRIN2A/GRIN2B/GRIN2C/GRIN2D/GRIN3A/GRIN3B/IL6/CACNA1C/CACNA1D/CACNA1F/CACNA1S/CSNK2A1/CSNK2B/HSPA2/COX4I1/COX5A/COX5B/COX6A2/COX6B1/COX6C/COX7A1/COX7B/COX7C/COX8A | 44 |
| hsa05167 | Kaposi sarcoma-associated herpesvirus infection | 42/359 | 2.43E-18 | 4.60E-17 | PTGS2/MAPK14/GSK3B/RELA/JUN/IKBKB/AKT1/BAX/CASP3/MAPK8/STAT1/ICAM1/PPP3CA/VEGFA/CCND1/FOS/CDKN1A/CASP9/MAPK1/RB1/IL6ST/TP53/NFKBIA/CASP8/RAF1/HIF1A/MYC/CXCL8/CXCL2/CHUK/E2F1/E2F2/JAK1/JAK2/CCR1/FGF2/ITPR1/IL6/NFKB1/CDK6/HCK/PIK3CG | 42 |
| hsa05163 | Human cytomegalovirus infection | 39/359 | 1.07E-13 | 6.07E-13 | PTGS2/MAPK14/GSK3B/RELA/IKBKB/AKT1/BAX/CASP3/PPP3CA/EGFR/VEGFA/CCND1/CDKN1A/CASP9/MAPK1/IL10RB/RB1/TP53/ELK1/NFKBIA/CASP8/RAF1/PRKCA/MYC/IL1B/CCL2/PTGER3/CXCL8/PRKCB/CHUK/E2F1/E2F2/JAK1/CCR1/ITPR1/IL6/NFKB1/CDK6/PTK2B | 39 |
| hsa05161 | Hepatitis B | 38/359 | 6.16E-18 | 1.00E-16 | CDK2/MAPK14/CCNA2/RELA/JUN/IKBKB/AKT1/BCL2/BAX/CASP3/MAPK8/STAT1/FOS/CDKN1A/CASP9/MMP9/MAPK1/RB1/TP53/ELK1/NFKBIA/CASP8/RAF1/PRKCA/MYC/CXCL8/PRKCB/BIRC5/CHUK/E2F1/E2F2/JAK3/JAK1/JAK2/TGFBR2/IL6/NFKB1/PTK2B | 38 |
| hsa04020 | Calcium signaling pathway | 38/359 | 4.17E-12 | 1.82E-11 | CHRM3/CHRM1/ADRA1A/CHRM2/ADRB2/NOS2/ADRA1B/ADRB1/ADRA1D/DRD1/CHRM5/KDR/MET/PPP3CA/EGFR/VEGFA/EGF/PRKCA/ERBB2/PTGER3/PRKCB/NOS3/ERBB3/ADRB3/ATP2A1/F2R/FGF1/FGF2/ITPR1/GRIN1/GRIN2A/GRIN2C/GRIN2D/CACNA1C/CACNA1D/CACNA1F/CACNA1S/PTK2B | 38 |
| hsa05418 | Fluid shear stress and atherosclerosis | 35/359 | 1.20E-17 | 1.70E-16 | MAPK14/RELA/NCF1/KDR/JUN/IKBKB/AKT1/BCL2/MAPK8/HMOX1/ICAM1/SELE/VCAM1/GSTP1/GSTM1/GSTM2/VEGFA/FOS/MMP2/MMP9/TP53/CAV1/IL1B/CCL2/NOS3/PLAT/THBD/IFNG/IL1A/NFE2L2/NQO1/CHUK/HSP90AA1/NFKB1/ACTB | 35 |
| hsa04933 | AGE-RAGE signaling pathway in diabetic complications | 34/359 | 8.75E-22 | 3.32E-20 | MAPK14/RELA/JUN/AKT1/BCL2/BAX/CASP3/MAPK8/STAT1/ICAM1/SELE/VCAM1/VEGFA/CCND1/MMP2/MAPK1/PRKCA/F3/IL1B/CCL2/CXCL8/PRKCB/NOS3/THBD/SERPINE1/COL1A1/IL1A/COL3A1/JAK2/NOX4/TGFBR2/IL6/NFKB1/PIM1 | 34 |
| hsa05169 | Epstein-Barr virus infection | 33/359 | 4.98E-11 | 1.66E-10 | CDK2/MAPK14/CCNA2/RELA/JUN/IKBKB/AKT1/BCL2/BAX/CASP3/MAPK8/STAT1/ICAM1/PSMD3/CCND1/CDKN1A/CASP9/RB1/TP53/NFKBIA/CASP8/MYC/CXCL10/CHUK/E2F1/E2F2/JAK3/JAK1/HDAC1/IL6/NFKB1/NFKB2/CDK6 | 33 |
| hsa05415 | Diabetic cardiomyopathy | 33/359 | 5.71E-11 | 1.82E-10 | MAPK14/GSK3B/RELA/NCF1/ATP5F1B/AKT1/MAPK8/SLC2A4/INSR/MMP2/MMP9/PRKCA/PRKCB/NOS3/COL1A1/PARP1/COL3A1/PPARA/CTSD/ATP2A1/TGFBR2/NFKB1/COX4I1/COX5A/COX5B/COX6A2/COX6B1/COX6C/COX7A1/COX7B/COX7C/COX8A/G6PD | 33 |
| hsa05166 | Human T-cell leukemia virus 1 infection | 33/359 | 6.48E-10 | 1.67E-09 | CDK2/CHEK1/CCNA2/RELA/JUN/IKBKB/AKT1/BAX/MAPK8/ICAM1/PPP3CA/CCND1/BCL2L1/FOS/CDKN1A/MAPK1/RB1/TP53/ELK1/NFKBIA/MYC/IL2/CHEK2/CHUK/E2F1/E2F2/JAK3/JAK1/POLB/TGFBR2/IL6/NFKB1/NFKB2 | 33 |
| hsa05014 | Amyotrophic lateral sclerosis | 33/359 | 6.99E-05 | 6.73E-05 | NOS2/GRIA2/MAPK14/ATP5F1B/BCL2/BAX/CASP3/PSMD3/PPP3CA/BCL2L1/CASP9/TP53/SOD1/HSPA5/TUBB1/HDAC6/GRIN1/GRIN2A/GRIN2B/GRIN2C/GRIN2D/SIGMAR1/ACTB/COX4I1/COX5A/COX5B/COX6A2/COX6B1/COX6C/COX7A1/COX7B/COX7C/COX8A | 33 |
| hsa04659 | Th17 cell differentiation | 31/359 | 1.58E-17 | 2.00E-16 | RXRA/MAPK14/RELA/RXRB/JUN/IL4R/IKBKB/MAPK8/STAT1/AHR/PPP3CA/FOS/MAPK1/IL6ST/NFKBIA/HIF1A/IL1B/IL2/IFNG/CHUK/JAK3/JAK1/JAK2/RORC/HSP90AA1/TGFBR2/IL6/NFKB1/RORA/RARA/RXRG | 31 |
| hsa05162 | Measles | 31/359 | 3.58E-14 | 2.37E-13 | CDK2/GSK3B/RELA/JUN/IKBKB/AKT1/BCL2/BAX/CASP3/MAPK8/STAT1/CCND1/BCL2L1/FOS/CASP9/TP53/NFKBIA/CASP8/IL1B/IL2/IL1A/CHUK/JAK3/JAK1/HSPA8/IL6/NFKB1/CDK6/CSNK2A1/CSNK2B/HSPA2 | 31 |
| hsa05160 | Hepatitis C | 31/359 | 1.18E-12 | 5.57E-12 | RXRA/CDK2/GSK3B/RELA/IKBKB/AKT1/BAX/CASP3/STAT1/EGFR/CCND1/CDKN1A/CASP9/MAPK1/EGF/RB1/TP53/NFKBIA/CASP8/RAF1/MYC/IFNG/CLDN4/PPARA/CXCL10/CHUK/E2F1/E2F2/JAK1/NFKB1/CDK6 | 31 |
| hsa05205 | Proteoglycans in cancer | 31/359 | 1.43E-09 | 3.46E-09 | ESR1/MAPK14/KDR/MET/AKT1/CASP3/EGFR/VEGFA/CCND1/CDKN1A/PLAU/MMP2/MMP9/MAPK1/TP53/ELK1/RAF1/PRKCA/HIF1A/ERBB2/CAV1/MYC/PRKCB/COL1A1/IGF2/ERBB3/VAV1/FGF2/HPSE/ITPR1/ACTB | 31 |
| hsa04024 | cAMP signaling pathway | 31/359 | 7.36E-09 | 1.47E-08 | CHRM1/CHRM2/ADRB2/GRIA2/RELA/ADRB1/DRD1/JUN/AKT1/MAPK8/FOS/MAPK1/NFKBIA/RAF1/PTGER3/PPARA/VAV1/ATP2A1/F2R/GRIN1/GRIN2A/GRIN2B/GRIN2C/GRIN2D/GRIN3A/GRIN3B/NFKB1/CACNA1C/CACNA1D/CACNA1F/CACNA1S | 31 |
| hsa05206 | MicroRNAs in cancer | 31/359 | 1.69E-05 | 1.90E-05 | PTGS2/MET/IKBKB/BCL2/CASP3/HMOX1/CYP1B1/EGFR/VEGFA/CCND1/CDKN1A/PLAU/MMP9/MAPK1/TP53/RAF1/PRKCA/ERBB2/MYC/PRKCB/RASSF1/E2F1/E2F2/ERBB3/RPS6KA5/ABCB1/HDAC1/MCL1/NFKB1/CDK6/PIM1 | 31 |
| hsa04932 | Non-alcoholic fatty liver disease | 30/359 | 4.75E-12 | 2.00E-11 | RXRA/PPARG/MAPK14/GSK3B/RELA/JUN/IKBKB/AKT1/BAX/CASP3/MAPK8/INSR/FOS/CASP8/IL1B/CXCL8/IL1A/PPARA/IL6/NFKB1/COX4I1/COX5A/COX5B/COX6A2/COX6B1/COX6C/COX7A1/COX7B/COX7C/COX8A | 30 |
| hsa05215 | Prostate cancer | 29/359 | 5.33E-17 | 6.06E-16 | AR/CDK2/GSK3B/RELA/IKBKB/AKT1/BCL2/GSTP1/MMP3/EGFR/CCND1/CDKN1A/CASP9/PLAU/MMP9/MAPK1/EGF/RB1/TP53/NFKBIA/RAF1/ERBB2/PLAT/CHUK/E2F1/E2F2/HSP90AA1/SRD5A2/NFKB1 | 29 |
| hsa04218 | Cellular senescence | 29/359 | 3.10E-11 | 1.14E-10 | CDK2/CHEK1/MAPK14/CCNA2/RELA/AKT1/CDK1/PPP3CA/CCND1/CDKN1A/MAPK1/RB1/TP53/RAF1/MYC/CXCL8/CCNB1/SERPINE1/IL1A/CHEK2/E2F1/E2F2/IGFBP3/ITPR1/TGFBR2/IL6/NFKB1/CACNA1D/CDK6 | 29 |
| hsa05165 | Human papillomavirus infection | 29/359 | 0.000331027 | 0.000291725 | PTGS2/CDK2/GSK3B/CCNA2/RELA/IKBKB/AKT1/BAX/CASP3/STAT1/EGFR/VEGFA/CCND1/CDKN1A/MAPK1/EGF/RB1/TP53/CASP8/RAF1/COL1A1/CHUK/SPP1/E2F1/IRF1/JAK1/HDAC1/NFKB1/CDK6 | 29 |
| hsa04210 | Apoptosis | 28/359 | 5.25E-12 | 2.13E-11 | RELA/JUN/IKBKB/AKT1/BCL2/BAX/CASP3/MAPK8/BCL2L1/FOS/CASP9/MAPK1/TP53/NFKBIA/CASP8/RAF1/BIRC5/PARP1/CHUK/CTSD/MCL1/CTSK/CTSS/CTSC/CTSF/ITPR1/NFKB1/ACTB | 28 |
| hsa04723 | Retrograde endocannabinoid signaling | 28/359 | 4.46E-11 | 1.54E-10 | PTGS2/GABRA1/GRIA2/MAPK14/MAPK8/MAPK1/PRKCA/PRKCB/GABRA2/GABRA3/GABRA4/GABRA5/GABRA6/GABRG1/GABRG2/GABRG3/ITPR1/GABRB1/GABRB2/GABRB3/GABRD/GABRE/GABRP/GABRQ/CACNA1C/CACNA1D/CACNA1F/CACNA1S | 28 |
| hsa05225 | Hepatocellular carcinoma | 28/359 | 9.69E-10 | 2.45E-09 | GSK3B/MET/AKT1/BAX/HMOX1/GSTP1/GSTM1/GSTM2/EGFR/CCND1/BCL2L1/CDKN1A/MAPK1/RB1/TP53/ELK1/RAF1/PRKCA/MYC/PRKCB/NFE2L2/NQO1/E2F1/E2F2/IGF2/TGFBR2/ACTB/CDK6 | 28 |
| hsa05164 | Influenza A | 28/359 | 1.69E-09 | 4.00E-09 | PRSS1/RELA/IKBKB/AKT1/BAX/CASP3/STAT1/ICAM1/CASP9/MAPK1/NFKBIA/CASP8/RAF1/PRKCA/IL1B/CCL2/CXCL8/PRKCB/IFNG/IL1A/CXCL10/CHUK/JAK1/JAK2/IL6/NFKB1/ACTB/CDK6 | 28 |
| hsa05152 | Tuberculosis | 28/359 | 4.85E-09 | 1.04E-08 | NOS2/MAPK14/RELA/AKT1/BCL2/BAX/CASP3/MAPK8/STAT1/PPP3CA/CASP9/MAPK1/IL10RB/CASP8/RAF1/IL1B/IFNG/IL1A/CTSD/JAK1/JAK2/CTSS/VDR/IL6/NFKB1/CLEC4E/CEBPB/CYP27B1 | 28 |
| hsa05202 | Transcriptional misregulation in cancer | 28/359 | 2.09E-08 | 3.82E-08 | RXRA/PPARG/CCNA2/RELA/RXRB/MET/BAX/MMP3/BCL2L1/CDKN1A/PLAU/MMP9/TP53/RUNX1T1/MYC/CXCL8/PLAT/MPO/RUNX2/IGFBP3/HDAC1/TGFBR2/IL6/NFKB1/CEBPB/RARA/RXRG/HOXA10 | 28 |
| hsa04014 | Ras signaling pathway | 28/359 | 1.19E-06 | 1.67E-06 | RELA/KDR/MET/IKBKB/AKT1/MAPK8/INSR/EGFR/VEGFA/BCL2L1/MAPK1/EGF/ELK1/RAF1/PRKCA/PRKCB/CHUK/RASSF1/IGF2/RASA1/FGF1/FGF2/PLA2G2E/GRIN1/GRIN2A/GRIN2B/NFKB1/PLA2G1B | 28 |
| hsa04668 | TNF signaling pathway | 27/359 | 2.30E-13 | 1.19E-12 | PTGS2/MAPK14/RELA/JUN/IKBKB/AKT1/CASP3/MAPK8/ICAM1/SELE/VCAM1/MMP3/FOS/MMP9/MAPK1/NFKBIA/CASP8/IL1B/CCL2/CXCL2/CXCL10/CHUK/IRF1/RPS6KA5/IL6/NFKB1/CEBPB | 27 |
| hsa05012 | Parkinson disease | 27/359 | 4.61E-05 | 4.86E-05 | MAOB/DRD1/SLC6A3/ATP5F1B/BAX/CASP3/MAPK8/PSMD3/BCL2L1/CASP9/TP53/SOD1/HSPA5/NFE2L2/TUBB1/ITPR1/UBA1/COX4I1/COX5A/COX5B/COX6A2/COX6B1/COX6C/COX7A1/COX7B/COX7C/COX8A | 27 |
| hsa05222 | Small cell lung cancer | 26/359 | 1.06E-14 | 8.59E-14 | PTGS2/RXRA/NOS2/CDK2/RELA/RXRB/IKBKB/AKT1/BCL2/BAX/CASP3/CCND1/BCL2L1/CDKN1A/CASP9/RB1/TP53/NFKBIA/MYC/CHUK/E2F1/E2F2/NFKB1/CDK6/RARB/RXRG | 26 |
| hsa04657 | IL-17 signaling pathway | 26/359 | 1.87E-14 | 1.42E-13 | PTGS2/MAPK14/GSK3B/RELA/JUN/IKBKB/CASP3/MAPK8/MMP1/MMP3/FOS/MMP9/MAPK1/NFKBIA/CASP8/IL1B/CCL2/CXCL8/IFNG/CXCL2/CXCL10/CHUK/HSP90AA1/IL6/NFKB1/CEBPB | 26 |
| hsa05226 | Gastric cancer | 26/359 | 1.42E-09 | 3.46E-09 | RXRA/CDK2/GSK3B/RXRB/MET/AKT1/BCL2/BAX/EGFR/CCND1/CDKN1A/MAPK1/EGF/RB1/TP53/RAF1/ERBB2/MYC/E2F1/E2F2/ABCB1/FGF1/FGF2/TGFBR2/RARB/RXRG | 26 |
| hsa05170 | Human immunodeficiency virus 1 infection | 26/359 | 2.13E-06 | 2.92E-06 | CHEK1/MAPK14/RELA/JUN/IKBKB/AKT1/BCL2/BAX/CASP3/MAPK8/CDK1/PPP3CA/BCL2L1/FOS/CASP9/MAPK1/NFKBIA/CASP8/RAF1/PRKCA/PRKCB/CCNB1/CHUK/ITPR1/NFKB1/PTK2B | 26 |
| hsa05016 | Huntington disease | 26/359 | 0.001058023 | 0.000871598 | GRIA2/PPARG/ATP5F1B/BAX/CASP3/MAPK8/PSMD3/CASP9/TP53/CASP8/SOD1/TUBB1/HDAC1/ITPR1/GRIN1/GRIN2B/COX4I1/COX5A/COX5B/COX6A2/COX6B1/COX6C/COX7A1/COX7B/COX7C/COX8A | 26 |
| hsa05212 | Pancreatic cancer | 25/359 | 6.49E-16 | 6.15E-15 | RELA/IKBKB/AKT1/BAX/MAPK8/STAT1/EGFR/VEGFA/CCND1/BCL2L1/CDKN1A/CASP9/MAPK1/EGF/RB1/TP53/RAF1/ERBB2/CHUK/E2F1/E2F2/JAK1/TGFBR2/NFKB1/CDK6 | 25 |
| hsa05145 | Toxoplasmosis | 25/359 | 1.15E-11 | 4.52E-11 | NOS2/MAPK14/RELA/IKBKB/AKT1/BCL2/CASP3/MAPK8/STAT1/ALOX5/BCL2L1/CASP9/MAPK1/IL10RB/NFKBIA/CASP8/IFNG/CHUK/CD40LG/JAK1/JAK2/HSPA8/NFKB1/HSPA2/PIK3CG | 25 |
| hsa05224 | Breast cancer | 25/359 | 5.16E-09 | 1.09E-08 | PGR/NCOA1/ESR2/ESR1/GSK3B/JUN/AKT1/BAX/EGFR/CCND1/FOS/CDKN1A/MAPK1/EGF/RB1/TP53/RAF1/ERBB2/MYC/E2F1/E2F2/FGF1/FGF2/NFKB2/CDK6 | 25 |
| hsa05203 | Viral carcinogenesis | 25/359 | 3.41E-06 | 4.41E-06 | CDK2/CHEK1/CCNA2/RELA/JUN/BAX/CASP3/CDK1/CCND1/CDKN1A/MAPK1/RB1/IL6ST/TP53/NFKBIA/CASP8/JAK3/JAK1/HDAC6/HDAC8/HDAC1/POLB/NFKB1/NFKB2/CDK6 | 25 |
| hsa05132 | Salmonella infection | 25/359 | 0.000105513 | 9.83E-05 | MAPK14/RELA/JUN/IKBKB/AKT1/BCL2/BAX/CASP3/MAPK8/FOS/MAPK1/NFKBIA/CASP8/RAF1/MYC/IL1B/CXCL8/CHUK/TUBB1/HSP90AA1/ARF1/IL6/NFKB1/ACTB/PIK3CG | 25 |
| hsa05033 | Nicotine addiction | 24/359 | 5.21E-23 | 5.92E-21 | GABRA1/GRIA2/GABRA2/GABRA3/GABRA4/GABRA5/GABRA6/GABRG1/GABRG2/GABRG3/GABRB1/GABRB2/GABRB3/GABRD/GABRE/GABRP/GABRQ/GRIN1/GRIN2A/GRIN2B/GRIN2C/GRIN2D/GRIN3A/GRIN3B | 24 |
| hsa05223 | Non-small cell lung cancer | 24/359 | 1.79E-15 | 1.56E-14 | RXRA/RXRB/MET/AKT1/BAX/EGFR/CCND1/CDKN1A/CASP9/MAPK1/EGF/RB1/TP53/RAF1/PRKCA/ERBB2/PRKCB/RASSF1/E2F1/E2F2/JAK3/CDK6/RARB/RXRG | 24 |
| hsa04066 | HIF-1 signaling pathway | 24/359 | 4.11E-11 | 1.46E-10 | NOS2/RELA/AKT1/BCL2/HMOX1/INSR/EGFR/VEGFA/CDKN1A/MAPK1/EGF/PRKCA/HIF1A/ERBB2/PRKCB/NOS3/SERPINE1/IFNG/HK2/EGLN1/TF/TFRC/IL6/NFKB1 | 24 |
| hsa04921 | Oxytocin signaling pathway | 24/359 | 6.10E-08 | 1.02E-07 | PTGS2/JUN/PPP3CA/EGFR/CCND1/FOS/CDKN1A/MAPK1/ELK1/RAF1/PRKCA/PRKCB/NOS3/ITPR1/CACNA1C/CACNA1D/CACNA1F/CACNA1S/CACNB1/CACNB2/CACNB3/CACNB4/ACTB/PIK3CG | 24 |
| hsa04062 | Chemokine signaling pathway | 24/359 | 3.79E-06 | 4.79E-06 | GSK3B/RELA/NCF1/IKBKB/AKT1/STAT1/MAPK1/NFKBIA/RAF1/CCL2/CXCL8/PRKCB/CXCL11/CXCL2/CXCL10/CHUK/JAK3/JAK2/VAV1/CCR1/NFKB1/HCK/PIK3CG/PTK2B | 24 |
| hsa05171 | Coronavirus disease - COVID-19 | 24/359 | 9.08E-05 | 8.53E-05 | MAPK14/RELA/JUN/IKBKB/MAPK8/MMP1/STAT1/MMP3/EGFR/FOS/MAPK1/IL6ST/NFKBIA/PRKCA/IL1B/CCL2/CXCL8/PRKCB/IL2/CXCL10/CHUK/JAK1/IL6/NFKB1 | 24 |
| hsa04915 | Estrogen signaling pathway | 23/359 | 3.20E-08 | 5.60E-08 | PGR/OPRM1/NCOA2/NCOA1/ESR2/ESR1/JUN/AKT1/BCL2/EGFR/FOS/MMP2/MMP9/MAPK1/RAF1/NOS3/CTSD/HSPA8/HSP90AA1/ITPR1/GPER1/HSPA2/RARA | 23 |
| hsa05131 | Shigellosis | 23/359 | 0.000590336 | 0.0005046 | MAPK14/GSK3B/RELA/JUN/IKBKB/AKT1/BCL2/BAX/MAPK8/EGFR/BCL2L1/MAPK1/TP53/NFKBIA/IL1B/CXCL8/CHUK/HK2/RPS6KA5/ARF1/ITPR1/NFKB1/ACTB | 23 |
| hsa04727 | GABAergic synapse | 22/359 | 2.39E-11 | 9.06E-11 | GABRA1/PRKCA/PRKCB/GABRA2/GABRA3/GABRA4/GABRA5/GABRA6/GABRG1/GABRG2/GABRG3/GABRB1/GABRB2/GABRB3/GABRD/GABRE/GABRP/GABRQ/CACNA1C/CACNA1D/CACNA1F/CACNA1S | 22 |
| hsa01522 | Endocrine resistance | 22/359 | 1.83E-10 | 5.07E-10 | ESR2/ESR1/MAPK14/JUN/AKT1/BCL2/BAX/MAPK8/EGFR/CCND1/FOS/CDKN1A/MMP2/MMP9/MAPK1/RB1/TP53/RAF1/ERBB2/E2F1/E2F2/GPER1 | 22 |
| hsa04625 | C-type lectin receptor signaling pathway | 22/359 | 6.20E-10 | 1.64E-09 | PTGS2/MAPK14/RELA/JUN/IKBKB/AKT1/MAPK8/STAT1/PPP3CA/MAPK1/NFKBIA/CASP8/RAF1/IL1B/IL2/CHUK/IRF1/ITPR1/IL6/NFKB1/NFKB2/CLEC4E | 22 |
| hsa04919 | Thyroid hormone signaling pathway | 22/359 | 1.24E-08 | 2.39E-08 | RXRA/NCOA2/NCOA1/ESR1/GSK3B/RXRB/AKT1/STAT1/DIO1/CCND1/CASP9/MAPK1/TP53/RAF1/PRKCA/HIF1A/MYC/PRKCB/ATP2A1/HDAC1/ACTB/RXRG | 22 |
| hsa04380 | Osteoclast differentiation | 22/359 | 3.61E-08 | 6.13E-08 | PPARG/MAPK14/RELA/NCF1/JUN/IKBKB/AKT1/MAPK8/STAT1/PPP3CA/FOS/MAPK1/NFKBIA/IL1B/IFNG/IL1A/CHUK/JAK1/CTSK/TGFBR2/NFKB1/NFKB2 | 22 |
| hsa04261 | Adrenergic signaling in cardiomyocytes | 22/359 | 6.48E-07 | 9.32E-07 | SCN5A/ADRA1A/ADRB2/ADRA1B/MAPK14/ADRB1/ADRA1D/AKT1/BCL2/MAPK1/PRKCA/RPS6KA5/ATP2A1/CACNA1C/CACNA1D/CACNA1F/CACNA1S/CACNB1/CACNB2/CACNB3/CACNB4/PIK3CG | 22 |
| hsa04630 | JAK-STAT signaling pathway | 22/359 | 2.45E-06 | 3.27E-06 | IL4R/AKT1/BCL2/STAT1/EGFR/CCND1/BCL2L1/CDKN1A/IL10RB/EGF/IL6ST/RAF1/MYC/IL2/IFNG/JAK3/JAK1/JAK2/MCL1/PTPN2/IL6/PIM1 | 22 |
| hsa04022 | cGMP-PKG signaling pathway | 22/359 | 4.08E-06 | 5.04E-06 | ADRA1A/ADRB2/ADRA1B/ADRB1/ADRA2C/ADRA1D/OPRD1/AKT1/INSR/PPP3CA/MAPK1/RAF1/NOS3/ADRB3/ATP2A1/ITPR1/CACNA1C/CACNA1D/CACNA1F/CACNA1S/PIK3CG/NPPB | 22 |
| hsa05130 | Pathogenic Escherichia coli infection | 22/359 | 5.71E-05 | 5.75E-05 | MAPK14/RELA/JUN/IKBKB/BAX/CASP3/MAPK8/FOS/CASP9/MAPK1/NFKBIA/CASP8/IL1B/CXCL8/CLDN4/CHUK/TUBB1/F2R/ARF1/IL6/NFKB1/ACTB | 22 |
| hsa04510 | Focal adhesion | 22/359 | 7.74E-05 | 7.39E-05 | GSK3B/KDR/MET/JUN/AKT1/BCL2/MAPK8/EGFR/VEGFA/CCND1/MAPK1/EGF/ELK1/RAF1/PRKCA/ERBB2/CAV1/PRKCB/COL1A1/SPP1/VAV1/ACTB | 22 |
| hsa00140 | Steroid hormone biosynthesis | 21/359 | 5.19E-14 | 3.11E-13 | HSD3B2/HSD3B1/CYP3A4/CYP1A2/CYP1A1/CYP1B1/AKR1C3/SULT1E1/CYP19A1/HSD11B1/CYP17A1/SRD5A1/SRD5A2/UGT2B7/COMT/AKR1C1/AKR1C2/HSD11B2/HSD17B1/SULT2B1/AKR1D1 | 21 |
| hsa05235 | PD-L1 expression and PD-1 checkpoint pathway in cancer | 21/359 | 1.77E-10 | 5.03E-10 | MAPK14/RELA/JUN/IKBKB/AKT1/STAT1/PPP3CA/EGFR/FOS/MAPK1/EGF/NFKBIA/RAF1/HIF1A/IFNG/CHUK/JAK1/JAK2/NFKB1/CSNK2A1/CSNK2B | 21 |
| hsa05142 | Chagas disease | 21/359 | 2.59E-09 | 5.90E-09 | NOS2/MAPK14/RELA/JUN/IKBKB/AKT1/MAPK8/FOS/MAPK1/NFKBIA/CASP8/IL1B/CCL2/CXCL8/IL2/SERPINE1/IFNG/CHUK/TGFBR2/IL6/NFKB1 | 21 |
| hsa04926 | Relaxin signaling pathway | 21/359 | 1.95E-07 | 2.96E-07 | NOS2/MAPK14/RELA/JUN/AKT1/MAPK8/MMP1/EGFR/VEGFA/FOS/MMP2/MMP9/MAPK1/NFKBIA/RAF1/PRKCA/NOS3/COL1A1/COL3A1/TGFBR2/NFKB1 | 21 |
| hsa04621 | NOD-like receptor signaling pathway | 21/359 | 6.12E-05 | 6.08E-05 | MAPK14/RELA/JUN/IKBKB/BCL2/MAPK8/STAT1/BCL2L1/MAPK1/NFKBIA/CASP8/IL1B/CCL2/CXCL8/CXCL2/CHUK/JAK1/HSP90AA1/ITPR1/IL6/NFKB1 | 21 |
| hsa05219 | Bladder cancer | 20/359 | 5.94E-17 | 6.13E-16 | MMP1/EGFR/VEGFA/CCND1/CDKN1A/MMP2/MMP9/MAPK1/EGF/RB1/TP53/RAF1/ERBB2/MYC/CXCL8/RASSF1/E2F1/E2F2/RPS6KA5/TYMP | 20 |
| hsa05220 | Chronic myeloid leukemia | 20/359 | 5.76E-11 | 1.82E-10 | RELA/IKBKB/AKT1/BAX/CCND1/BCL2L1/CDKN1A/MAPK1/RB1/TP53/NFKBIA/RAF1/MYC/CHUK/E2F1/E2F2/HDAC1/TGFBR2/NFKB1/CDK6 | 20 |
| hsa01521 | EGFR tyrosine kinase inhibitor resistance | 20/359 | 1.24E-10 | 3.61E-10 | GSK3B/KDR/MET/AKT1/BCL2/BAX/EGFR/VEGFA/BCL2L1/MAPK1/EGF/RAF1/PRKCA/ERBB2/PRKCB/ERBB3/JAK1/JAK2/FGF2/IL6 | 20 |
| hsa05032 | Morphine addiction | 20/359 | 1.87E-09 | 4.34E-09 | OPRM1/GABRA1/DRD1/PRKCA/PRKCB/GABRA2/GABRA3/GABRA4/GABRA5/GABRA6/GABRG1/GABRG2/GABRG3/GABRB1/GABRB2/GABRB3/GABRD/GABRE/GABRP/GABRQ | 20 |
| hsa04064 | NF-kappa B signaling pathway | 20/359 | 2.15E-08 | 3.82E-08 | PTGS2/RELA/IKBKB/BCL2/ICAM1/VCAM1/BCL2L1/PLAU/NFKBIA/IL1B/CXCL8/PRKCB/PARP1/CXCL2/CHUK/CD40LG/NFKB1/NFKB2/CSNK2A1/CSNK2B | 20 |
| hsa04620 | Toll-like receptor signaling pathway | 20/359 | 2.15E-08 | 3.82E-08 | MAPK14/RELA/JUN/IKBKB/AKT1/MAPK8/STAT1/FOS/MAPK1/NFKBIA/CASP8/IL1B/CXCL8/CXCL11/CXCL10/CHUK/SPP1/CTSK/IL6/NFKB1 | 20 |
| hsa04726 | Serotonergic synapse | 20/359 | 1.25E-07 | 1.97E-07 | PTGS2/PTGS1/MAOB/HTR3A/SLC6A4/CASP3/ALOX5/MAPK1/RAF1/PRKCA/PRKCB/CYP2C19/ITPR1/GABRB1/GABRB2/GABRB3/CACNA1C/CACNA1D/CACNA1F/CACNA1S | 20 |
| hsa05135 | Yersinia infection | 20/359 | 2.27E-06 | 3.07E-06 | MAPK14/GSK3B/RELA/JUN/IKBKB/AKT1/MAPK8/FOS/MAPK1/NFKBIA/IL1B/CCL2/CXCL8/IL2/CHUK/VAV1/IL6/NFKB1/ACTB/PTK2B | 20 |
| hsa04015 | Rap1 signaling pathway | 20/359 | 0.001006388 | 0.000835112 | MAPK14/KDR/MET/AKT1/INSR/EGFR/VEGFA/MAPK1/EGF/RAF1/PRKCA/PRKCB/VAV1/F2R/FGF1/FGF2/GRIN1/GRIN2A/GRIN2B/ACTB | 20 |
| hsa05031 | Amphetamine addiction | 19/359 | 7.41E-11 | 2.28E-10 | GRIA2/MAOB/DRD1/SLC6A3/JUN/PPP3CA/FOS/PRKCA/PRKCB/HDAC1/GRIN1/GRIN2A/GRIN2B/GRIN2C/GRIN2D/GRIN3A/GRIN3B/CACNA1C/CACNA1D | 19 |
| hsa05210 | Colorectal cancer | 19/359 | 4.37E-09 | 9.55E-09 | GSK3B/JUN/AKT1/BCL2/BAX/CASP3/MAPK8/EGFR/CCND1/FOS/CDKN1A/CASP9/MAPK1/EGF/TP53/RAF1/MYC/BIRC5/TGFBR2 | 19 |
| hsa04260 | Cardiac muscle contraction | 19/359 | 5.37E-09 | 1.11E-08 | ATP2A1/CACNA1C/CACNA1D/CACNA1F/CACNA1S/CACNB1/CACNB2/CACNB3/CACNB4/COX4I1/COX5A/COX5B/COX6A2/COX6B1/COX6C/COX7A1/COX7B/COX7C/COX8A | 19 |
| hsa04725 | Cholinergic synapse | 19/359 | 4.49E-07 | 6.62E-07 | CHRM3/CHRM1/ACHE/CHRM2/CHRM5/CHRM4/AKT1/BCL2/FOS/MAPK1/PRKCA/PRKCB/JAK2/ITPR1/CACNA1C/CACNA1D/CACNA1F/CACNA1S/PIK3CG | 19 |
| hsa04934 | Cushing syndrome | 19/359 | 5.17E-05 | 5.34E-05 | CDK2/GSK3B/HSD3B2/HSD3B1/AHR/EGFR/CCND1/CDKN1A/MAPK1/RB1/E2F1/E2F2/CYP17A1/ITPR1/CACNA1C/CACNA1D/CACNA1F/CACNA1S/CDK6 | 19 |
| hsa05140 | Leishmaniasis | 18/359 | 4.30E-09 | 9.55E-09 | PTGS2/NOS2/MAPK14/RELA/NCF1/JUN/STAT1/FOS/MAPK1/ELK1/NFKBIA/IL1B/PRKCB/IFNG/IL1A/JAK1/JAK2/NFKB1 | 18 |
| hsa04658 | Th1 and Th2 cell differentiation | 18/359 | 8.38E-08 | 1.35E-07 | MAPK14/RELA/JUN/IL4R/IKBKB/MAPK8/STAT1/PPP3CA/FOS/MAPK1/NFKBIA/IL2/IFNG/CHUK/JAK3/JAK1/JAK2/NFKB1 | 18 |
| hsa04660 | T cell receptor signaling pathway | 18/359 | 5.81E-07 | 8.46E-07 | MAPK14/GSK3B/RELA/JUN/IKBKB/AKT1/MAPK8/PPP3CA/FOS/MAPK1/NFKBIA/RAF1/IL2/IFNG/CHUK/CD40LG/VAV1/NFKB1 | 18 |
| hsa04728 | Dopaminergic synapse | 18/359 | 1.92E-05 | 2.12E-05 | GRIA2/MAPK14/GSK3B/MAOB/DRD1/SLC6A3/AKT1/MAPK8/PPP3CA/FOS/PRKCA/PRKCB/COMT/ITPR1/GRIN2A/GRIN2B/CACNA1C/CACNA1D | 18 |
| hsa04917 | Prolactin signaling pathway | 17/359 | 6.26E-09 | 1.27E-08 | ESR2/ESR1/MAPK14/GSK3B/RELA/AKT1/MAPK8/STAT1/CCND1/FOS/MAPK1/RAF1/IRF1/JAK2/CYP17A1/GCK/NFKB1 | 17 |
| hsa04115 | p53 signaling pathway | 17/359 | 1.24E-08 | 2.39E-08 | CDK2/CHEK1/BCL2/BAX/CASP3/CDK1/CCND1/BCL2L1/CDKN1A/CASP9/TP53/CASP8/CCNB1/SERPINE1/CHEK2/IGFBP3/CDK6 | 17 |
| hsa04935 | Growth hormone synthesis, secretion and action | 17/359 | 1.76E-05 | 1.96E-05 | MAPK14/GSK3B/AKT1/MAPK8/STAT1/FOS/MAPK1/RAF1/PRKCA/PRKCB/IGFBP3/JAK2/ITPR1/CACNA1C/CACNA1D/CACNA1F/CACNA1S | 17 |
| hsa04068 | FoxO signaling pathway | 17/359 | 6.22E-05 | 6.10E-05 | CDK2/MAPK14/IKBKB/AKT1/MAPK8/SLC2A4/INSR/EGFR/CCND1/CDKN1A/MAPK1/EGF/RAF1/CCNB1/CHUK/TGFBR2/IL6 | 17 |
| hsa05034 | Alcoholism | 17/359 | 0.003836863 | 0.002927455 | MAOB/DRD1/SLC6A3/PKIA/MAPK1/RAF1/SLC29A1/HDAC6/HDAC8/HDAC1/GRIN1/GRIN2A/GRIN2B/GRIN2C/GRIN2D/GRIN3A/GRIN3B | 17 |
| hsa05204 | Chemical carcinogenesis - DNA adducts | 16/359 | 3.59E-08 | 6.13E-08 | PTGS2/CYP3A4/CYP1A2/CYP1A1/CYP1B1/GSTP1/GSTM1/GSTM2/HSD11B1/EPHX1/CYP2C19/UGT2B7/HPGDS/AKR1C2/SULT2A1/CBR1 | 16 |
| hsa05218 | Melanoma | 16/359 | 6.83E-08 | 1.13E-07 | MET/AKT1/BAX/EGFR/CCND1/CDKN1A/MAPK1/EGF/RB1/TP53/RAF1/E2F1/E2F2/FGF1/FGF2/CDK6 | 16 |
| hsa01524 | Platinum drug resistance | 16/359 | 8.40E-08 | 1.35E-07 | AKT1/BCL2/BAX/CASP3/GSTP1/GSTM1/GSTM2/BCL2L1/CDKN1A/CASP9/MAPK1/TP53/CASP8/ERBB2/BIRC5/TOP2A | 16 |
| hsa00980 | Metabolism of xenobiotics by cytochrome P450 | 16/359 | 2.24E-07 | 3.35E-07 | ADH1B/ADH1C/CYP3A4/CYP1A2/CYP1A1/CYP1B1/GSTP1/GSTM1/GSTM2/HSD11B1/EPHX1/UGT2B7/HPGDS/AKR1C1/SULT2A1/CBR1 | 16 |
| hsa04912 | GnRH signaling pathway | 16/359 | 2.69E-06 | 3.56E-06 | MAPK14/JUN/MAPK8/EGFR/MMP2/MAPK1/ELK1/RAF1/PRKCA/PRKCB/ITPR1/CACNA1C/CACNA1D/CACNA1F/CACNA1S/PTK2B | 16 |
| hsa04931 | Insulin resistance | 16/359 | 1.96E-05 | 2.14E-05 | GSK3B/PYGM/RELA/IKBKB/AKT1/MAPK8/SLC2A4/INSR/NFKBIA/PRKCB/NOS3/PPARA/PTPN1/OGT/IL6/NFKB1 | 16 |
| hsa04724 | Glutamatergic synapse | 16/359 | 3.89E-05 | 4.13E-05 | GRIA2/PPP3CA/MAPK1/PRKCA/PRKCB/GRM4/ITPR1/GRIN1/GRIN2A/GRIN2B/GRIN2C/GRIN2D/GRIN3A/GRIN3B/CACNA1C/CACNA1D | 16 |
| hsa04110 | Cell cycle | 16/359 | 0.000132441 | 0.00012241 | CDK2/CHEK1/GSK3B/CCNA2/CDK1/CCND1/CDKN1A/RB1/TP53/MYC/CCNB1/CHEK2/E2F1/E2F2/HDAC1/CDK6 | 16 |
| hsa05017 | Spinocerebellar ataxia | 16/359 | 0.000570795 | 0.000491593 | GRIA2/AKT1/MAPK8/PSMD3/PRKCA/PRKCB/ATP2A1/ITPR1/GRIN1/GRIN2A/GRIN2B/GRIN2C/GRIN2D/GRIN3A/GRIN3B/RORA | 16 |
| hsa05134 | Legionellosis | 15/359 | 1.52E-08 | 2.84E-08 | RELA/CASP3/CASP9/NFKBIA/CASP8/IL1B/CXCL8/CXCL2/HSF1/HSPA8/ARF1/IL6/NFKB1/NFKB2/HSPA2 | 15 |
| hsa05221 | Acute myeloid leukemia | 15/359 | 1.59E-07 | 2.45E-07 | CCNA2/PPARD/RELA/IKBKB/AKT1/CCND1/MAPK1/RAF1/RUNX1T1/MYC/MPO/CHUK/NFKB1/PIM1/RARA | 15 |
| hsa05214 | Glioma | 15/359 | 7.59E-07 | 1.08E-06 | AKT1/BAX/EGFR/CCND1/CDKN1A/MAPK1/EGF/RB1/TP53/RAF1/PRKCA/PRKCB/E2F1/E2F2/CDK6 | 15 |
| hsa04012 | ErbB signaling pathway | 15/359 | 3.99E-06 | 4.98E-06 | GSK3B/JUN/AKT1/MAPK8/EGFR/CDKN1A/MAPK1/EGF/ELK1/RAF1/PRKCA/ERBB2/MYC/PRKCB/ERBB3 | 15 |
| hsa04928 | Parathyroid hormone synthesis, secretion and action | 15/359 | 6.15E-05 | 6.08E-05 | RXRA/RXRB/BCL2/EGFR/FOS/CDKN1A/MAPK1/RAF1/PRKCA/PRKCB/RUNX2/VDR/ITPR1/RXRG/CYP27B1 | 15 |
| hsa04071 | Sphingolipid signaling pathway | 15/359 | 0.000232252 | 0.000207901 | MAPK14/RELA/OPRD1/AKT1/BCL2/BAX/MAPK8/MAPK1/TP53/RAF1/PRKCA/PRKCB/NOS3/CTSD/NFKB1 | 15 |
| hsa04722 | Neurotrophin signaling pathway | 15/359 | 0.000232252 | 0.000207901 | MAPK14/GSK3B/RELA/JUN/IKBKB/AKT1/BCL2/BAX/MAPK8/MAPK1/TP53/NFKBIA/RAF1/RPS6KA5/NFKB1 | 15 |
| hsa04270 | Vascular smooth muscle contraction | 15/359 | 0.000775156 | 0.000652763 | ADRA1A/ADRA1B/ADRA1D/MAPK1/RAF1/PRKCA/PRKCB/ITPR1/PLA2G2E/CACNA1C/CACNA1D/CACNA1F/CACNA1S/NPPB/PLA2G1B | 15 |
| hsa04217 | Necroptosis | 15/359 | 0.004567585 | 0.003393871 | PYGM/BCL2/BAX/MAPK8/STAT1/CASP8/IL1B/IFNG/IL1A/PARP1/JAK3/JAK1/JAK2/HSP90AA1/FTH1 | 15 |
| hsa05030 | Cocaine addiction | 14/359 | 1.46E-08 | 2.76E-08 | GRIA2/MAOB/RELA/DRD1/SLC6A3/JUN/GRIN1/GRIN2A/GRIN2B/GRIN2C/GRIN2D/GRIN3A/GRIN3B/NFKB1 | 14 |
| hsa05213 | Endometrial cancer | 14/359 | 1.52E-07 | 2.37E-07 | GSK3B/AKT1/BAX/EGFR/CCND1/CDKN1A/CASP9/MAPK1/EGF/TP53/ELK1/RAF1/ERBB2/MYC | 14 |
| hsa05133 | Pertussis | 14/359 | 4.97E-06 | 6.01E-06 | NOS2/MAPK14/RELA/JUN/CASP3/MAPK8/FOS/MAPK1/IL1B/CXCL8/IL1A/IRF1/IL6/NFKB1 | 14 |
| hsa04662 | B cell receptor signaling pathway | 14/359 | 1.25E-05 | 1.42E-05 | GSK3B/RELA/JUN/IKBKB/AKT1/PPP3CA/FOS/MAPK1/NFKBIA/RAF1/PRKCB/CHUK/VAV1/NFKB1 | 14 |
| hsa05323 | Rheumatoid arthritis | 14/359 | 5.40E-05 | 5.53E-05 | JUN/MMP1/ICAM1/MMP3/VEGFA/FOS/IL1B/CCL2/CXCL8/IFNG/IL1A/CXCL2/CTSK/IL6 | 14 |
| hsa04713 | Circadian entrainment | 14/359 | 8.66E-05 | 8.21E-05 | GRIA2/FOS/MAPK1/PRKCA/PRKCB/RPS6KA5/ITPR1/GRIN1/GRIN2A/GRIN2B/GRIN2C/GRIN2D/CACNA1C/CACNA1D | 14 |
| hsa05146 | Amoebiasis | 14/359 | 0.000150632 | 0.000138101 | NOS2/RELA/CASP3/PRKCA/IL1B/CXCL8/PRKCB/HSPB1/COL1A1/IFNG/COL3A1/CXCL2/IL6/NFKB1 | 14 |
| hsa04910 | Insulin signaling pathway | 14/359 | 0.002947461 | 0.002326943 | GSK3B/PYGM/IKBKB/AKT1/MAPK8/SLC2A4/INSR/MAPK1/ELK1/RAF1/ACACA/HK2/PTPN1/GCK | 14 |
| hsa01523 | Antifolate resistance | 13/359 | 2.07E-10 | 5.62E-10 | RELA/IKBKB/IL1B/ABCG2/CHUK/TYMS/DHFR/FOLR1/FOLR2/SLC46A1/FPGS/IL6/NFKB1 | 13 |
| hsa04370 | VEGF signaling pathway | 13/359 | 1.31E-06 | 1.82E-06 | PTGS2/MAPK14/KDR/AKT1/PPP3CA/VEGFA/CASP9/MAPK1/RAF1/PRKCA/PRKCB/NOS3/HSPB1 | 13 |
| hsa04929 | GnRH secretion | 13/359 | 3.48E-06 | 4.45E-06 | ESR2/AKT1/MAPK1/RAF1/PRKCA/PRKCB/SPP1/ITPR1/CACNA1C/CACNA1D/CACNA1F/CACNA1S/GPER1 | 13 |
| hsa04720 | Long-term potentiation | 13/359 | 5.96E-06 | 7.13E-06 | GRIA2/PPP3CA/MAPK1/RAF1/PRKCA/PRKCB/ITPR1/GRIN1/GRIN2A/GRIN2B/GRIN2C/GRIN2D/CACNA1C | 13 |
| hsa04920 | Adipocytokine signaling pathway | 13/359 | 8.38E-06 | 9.92E-06 | RXRA/RELA/RXRB/IKBKB/AKT1/MAPK8/SLC2A4/NFKBIA/PPARA/CHUK/JAK2/NFKB1/RXRG | 13 |
| hsa05120 | Epithelial cell signaling in Helicobacter pylori infection | 13/359 | 9.88E-06 | 1.15E-05 | MAPK14/RELA/MET/JUN/IKBKB/CASP3/MAPK8/EGFR/NFKBIA/CXCL8/CXCL2/CHUK/NFKB1 | 13 |
| hsa04072 | Phospholipase D signaling pathway | 13/359 | 0.01413099 | 0.009505742 | AKT1/INSR/EGFR/MAPK1/EGF/RAF1/PRKCA/CXCL8/F2R/GRM4/ARF1/PIK3CG/PTK2B | 13 |
| hsa05321 | Inflammatory bowel disease | 12/359 | 2.32E-05 | 2.51E-05 | RELA/JUN/IL4R/STAT1/IL1B/IL2/IFNG/IL1A/RORC/IL6/NFKB1/RORA | 12 |
| hsa05230 | Central carbon metabolism in cancer | 12/359 | 5.01E-05 | 5.22E-05 | MET/AKT1/EGFR/MAPK1/TP53/RAF1/HIF1A/ERBB2/MYC/HK2/GCK/G6PD | 12 |
| hsa04540 | Gap junction | 12/359 | 0.000470918 | 0.000411815 | ADRB1/DRD1/CDK1/EGFR/MAPK1/EGF/RAF1/PRKCA/GJA1/PRKCB/TUBB1/ITPR1 | 12 |
| hsa04670 | Leukocyte transendothelial migration | 12/359 | 0.004512126 | 0.00337472 | MAPK14/NCF1/ICAM1/VCAM1/MMP2/MMP9/PRKCA/PRKCB/CLDN4/VAV1/ACTB/PTK2B | 12 |
| hsa04611 | Platelet activation | 12/359 | 0.008768055 | 0.006151867 | PTGS1/MAPK14/AKT1/MAPK1/NOS3/COL1A1/COL3A1/F2R/TBXAS1/ITPR1/ACTB/PIK3CG | 12 |
| hsa04550 | Signaling pathways regulating pluripotency of stem cells | 12/359 | 0.024813626 | 0.016305882 | MAPK14/GSK3B/AKT1/MAPK1/IL6ST/RAF1/MYC/JAK3/JAK1/JAK2/FGF2/ESRRB | 12 |
| hsa05144 | Malaria | 11/359 | 8.82E-06 | 1.03E-05 | MET/ICAM1/SELE/VCAM1/IL1B/CCL2/CXCL8/IFNG/CD40LG/HBA1/IL6 | 11 |
| hsa04913 | Ovarian steroidogenesis | 11/359 | 1.08E-05 | 1.24E-05 | PTGS2/HSD3B2/HSD3B1/CYP1A1/CYP1B1/ALOX5/INSR/AKR1C3/CYP19A1/CYP17A1/HSD17B1 | 11 |
| hsa00590 | Arachidonic acid metabolism | 11/359 | 6.37E-05 | 6.19E-05 | PTGS2/PTGS1/ALOX5/AKR1C3/CYP2C19/TBXAS1/PTGES/HPGDS/PLA2G2E/CBR1/PLA2G1B | 11 |
| hsa00982 | Drug metabolism - cytochrome P450 | 11/359 | 0.000297136 | 0.000263904 | ADH1B/ADH1C/MAOB/CYP3A4/CYP1A2/GSTP1/GSTM1/GSTM2/CYP2C19/UGT2B7/HPGDS | 11 |
| hsa05412 | Arrhythmogenic right ventricular cardiomyopathy | 11/359 | 0.000538736 | 0.000467525 | GJA1/ATP2A1/CACNA1C/CACNA1D/CACNA1F/CACNA1S/CACNB1/CACNB2/CACNB3/CACNB4/ACTB | 11 |
| hsa00983 | Drug metabolism - other enzymes | 11/359 | 0.000750142 | 0.000636413 | CYP3A4/GSTP1/GSTM1/GSTM2/MPO/TYMP/CDA/TK1/UGT2B7/CES2/CES1 | 11 |
| hsa04742 | Taste transduction | 11/359 | 0.001381481 | 0.001121804 | CHRM3/GABRA1/HTR3A/GRM4/TAS2R31/GABRA2/GABRA3/GABRA4/GABRA5/GABRA6/CACNA1C | 11 |
| hsa04976 | Bile secretion | 11/359 | 0.001831827 | 0.001466548 | RXRA/CYP3A4/ABCG2/ABCB1/SLC5A1/CA2/EPHX1/HMGCR/UGT2B7/SULT2A1/NR1H4 | 11 |
| hsa05410 | Hypertrophic cardiomyopathy | 11/359 | 0.002006139 | 0.001594869 | ATP2A1/IL6/CACNA1C/CACNA1D/CACNA1F/CACNA1S/CACNB1/CACNB2/CACNB3/CACNB4/ACTB | 11 |
| hsa05414 | Dilated cardiomyopathy | 11/359 | 0.00335683 | 0.002596044 | ADRB1/ATP2A1/CACNA1C/CACNA1D/CACNA1F/CACNA1S/CACNB1/CACNB2/CACNB3/CACNB4/ACTB | 11 |
| hsa05231 | Choline metabolism in cancer | 11/359 | 0.003942096 | 0.002987694 | JUN/AKT1/MAPK8/EGFR/FOS/MAPK1/EGF/RAF1/PRKCA/HIF1A/PRKCB | 11 |
| hsa04914 | Progesterone-mediated oocyte maturation | 11/359 | 0.005356028 | 0.003878317 | PGR/CDK2/MAPK14/CCNA2/AKT1/MAPK8/CDK1/MAPK1/RAF1/CCNB1/HSP90AA1 | 11 |
| hsa05216 | Thyroid cancer | 10/359 | 3.10E-06 | 4.05E-06 | RXRA/PPARG/RXRB/BAX/CCND1/CDKN1A/MAPK1/TP53/MYC/RXRG | 10 |
| hsa04622 | RIG-I-like receptor signaling pathway | 10/359 | 0.000957079 | 0.000800035 | MAPK14/RELA/IKBKB/MAPK8/NFKBIA/CASP8/CXCL8/CXCL10/CHUK/NFKB1 | 10 |
| hsa04520 | Adherens junction | 10/359 | 0.001071037 | 0.000875971 | MET/INSR/EGFR/MAPK1/ERBB2/PTPN1/TGFBR2/ACTB/CSNK2A1/CSNK2B | 10 |
| hsa03320 | PPAR signaling pathway | 10/359 | 0.001819455 | 0.001466548 | RXRA/PPARG/PPARD/OLR1/RXRB/MMP1/PPARA/DBI/RXRG/FABP6 | 10 |
| hsa04970 | Salivary secretion | 10/359 | 0.007925738 | 0.005631446 | CHRM3/ADRA1A/ADRB2/ADRA1B/ADRB1/ADRA1D/PRKCA/PRKCB/ADRB3/ITPR1 | 10 |
| hsa04750 | Inflammatory mediator regulation of TRP channels | 10/359 | 0.011312171 | 0.00774708 | MAPK14/MAPK8/PRKCA/IL1B/PRKCB/TRPA1/TRPM8/ITPR1/TRPV3/TRPV1 | 10 |
| hsa04061 | Viral protein interaction with cytokine and cytokine receptor | 10/359 | 0.012937234 | 0.008806941 | IL10RB/IL6ST/CCL2/CXCL8/IL2/CXCL11/CXCL2/CXCL10/CCR1/IL6 | 10 |
| hsa04972 | Pancreatic secretion | 10/359 | 0.014731979 | 0.009794113 | CHRM3/PRSS1/PRKCA/PRKCB/ATP2A1/CA2/ITPR1/PLA2G2E/CTRB1/PLA2G1B | 10 |
| hsa05143 | African trypanosomiasis | 9/359 | 2.48E-05 | 2.66E-05 | ICAM1/SELE/VCAM1/PRKCA/IL1B/PRKCB/IFNG/HBA1/IL6 | 9 |
| hsa04930 | Type II diabetes mellitus | 9/359 | 0.000154236 | 0.000140273 | IKBKB/MAPK8/SLC2A4/INSR/MAPK1/HK2/GCK/CACNA1C/CACNA1D | 9 |
| hsa04924 | Renin secretion | 9/359 | 0.003233462 | 0.002517764 | ADRB2/ADRB1/PPP3CA/ADRB3/ITPR1/CACNA1C/CACNA1D/CACNA1F/CACNA1S | 9 |
| hsa05211 | Renal cell carcinoma | 9/359 | 0.003233462 | 0.002517764 | MET/JUN/AKT1/VEGFA/CDKN1A/MAPK1/RAF1/HIF1A/EGLN1 | 9 |
| hsa04137 | Mitophagy - animal | 9/359 | 0.00432285 | 0.003254568 | RELA/JUN/MAPK8/BCL2L1/TP53/HIF1A/E2F1/CSNK2A1/CSNK2B | 9 |
| hsa04911 | Insulin secretion | 9/359 | 0.013605305 | 0.009206597 | CHRM3/PRKCA/PRKCB/FFAR1/GCK/CACNA1C/CACNA1D/CACNA1F/CACNA1S | 9 |
| hsa04925 | Aldosterone synthesis and secretion | 9/359 | 0.02940257 | 0.019210391 | HSD3B2/HSD3B1/PRKCA/PRKCB/ITPR1/CACNA1C/CACNA1D/CACNA1F/CACNA1S | 9 |
| hsa04215 | Apoptosis - multiple species | 8/359 | 5.72E-05 | 5.75E-05 | BCL2/BAX/CASP3/MAPK8/BCL2L1/CASP9/CASP8/BIRC5 | 8 |
| hsa04923 | Regulation of lipolysis in adipocytes | 8/359 | 0.003413045 | 0.002621684 | PTGS2/ADRB2/PTGS1/ADRB1/AKT1/INSR/PTGER3/ADRB3 | 8 |
| hsa05416 | Viral myocarditis | 8/359 | 0.004710082 | 0.003477026 | CASP3/ICAM1/CCND1/CASP9/CASP8/CAV1/CD40LG/ACTB | 8 |
| hsa04623 | Cytosolic DNA-sensing pathway | 8/359 | 0.006355529 | 0.004572932 | RELA/IKBKB/NFKBIA/IL1B/CXCL10/CHUK/IL6/NFKB1 | 8 |
| hsa04927 | Cortisol synthesis and secretion | 8/359 | 0.007672996 | 0.005486154 | HSD3B2/HSD3B1/CYP17A1/ITPR1/CACNA1C/CACNA1D/CACNA1F/CACNA1S | 8 |
| hsa04664 | Fc epsilon RI signaling pathway | 8/359 | 0.01002116 | 0.006946632 | MAPK14/AKT1/MAPK8/ALOX5/MAPK1/RAF1/PRKCA/VAV1 | 8 |
| hsa00910 | Nitrogen metabolism | 7/359 | 4.22E-06 | 5.16E-06 | CA2/CA1/CA7/CA12/CA14/CA9/CA4 | 7 |
| hsa04960 | Aldosterone-regulated sodium reabsorption | 6/359 | 0.005290343 | 0.003855311 | INSR/MAPK1/PRKCA/PRKCB/HSD11B2/NR3C2 | 6 |
| hsa04216 | Ferroptosis | 6/359 | 0.008820524 | 0.006151867 | HMOX1/TP53/CP/FTH1/TF/TFRC | 6 |
| hsa00790 | Folate biosynthesis | 5/359 | 0.005082843 | 0.003727994 | AKR1B1/AKR1C3/DHFR/FPGS/CBR1 | 5 |
| hsa00591 | Linoleic acid metabolism | 5/359 | 0.008230339 | 0.00581155 | CYP3A4/CYP1A2/CYP2C19/PLA2G2E/PLA2G1B | 5 |
| hsa03410 | Base excision repair | 5/359 | 0.014236656 | 0.009520488 | PARP1/FEN1/NEIL1/NEIL2/POLB | 5 |
| hsa00350 | Tyrosine metabolism | 5/359 | 0.020302383 | 0.013418956 | ADH1B/ADH1C/MAOB/TYR/COMT | 5 |
| hsa00100 | Steroid biosynthesis | 4/359 | 0.010488541 | 0.007226554 | SOAT1/SOAT2/LSS/CYP27B1 | 4 |
