# Supplementary material for: Mechanism of drug-pairs Astragalus Mongholicus–Largehead Atractylodes on treating knee osteoarthritis investigated by GEO gene chip with network pharmacology and molecular docking
Source: Medicine (Baltimore). 2024 Jul 5;103(27):e38699. doi: 10.1097/MD.0000000000038699 (PMC11224889; doi:10.1097/MD.0000000000038699)
Supplement: Supplementary file 14 [file medi-103-e38699-s014.doc]

# **Appendix 14**

**The results of drug and disease molecular docking**

**Table S14. The results of drug and disease molecular docking.**

| Ingredient | Molecular Formula | Molecular Weight | Crystal structure | Target | Affinity energy/(kj·mol^-1^) |
| --- | --- | --- | --- | --- | --- |
| kaempferol | C_15_H_10_O_6_ | 286.24g/mol | 6y3v | JUN | -6.3 |
| quercetin | C_15_H_10_O_7_ | 302.23g/mol |  |  | -6.4 |
| quercetin | C_15_H_10_O_7_ | 302.23g/mol | 4zzn | MAPK1 | -7.5 |
| 7-O-methylisomucronulatol | C_18_H_20_O_5_ | 316.3g/mol | 3k3i | MAPK14 | -7.9 |
| Mucronulatol | C_17_H_18_O_5_ | 302.32g/mol |  |  | -7.8 |
| Calycosin | C_16_H_12_O_5_ | 284.26g/mol |  |  | -7.8 |
| isorhamnetin | C_16_H_12_O_7_ | 316.26g/mol |  |  | -8.2 |
| quercetin | C_15_H_10_O_7_ | 302.23g/moll | 4y7r | MYC | -8.0 |
| quercetin | C_15_H_10_O_7_ | 302.23g/moll | 6wqx | TP53 | -8.0 |
