# Supplementary material for: Mechanism of drug-pairs Astragalus Mongholicus–Largehead Atractylodes on treating knee osteoarthritis investigated by GEO gene chip with network pharmacology and molecular docking
Source: Medicine (Baltimore). 2024 Jul 5;103(27):e38699. doi: 10.1097/MD.0000000000038699 (PMC11224889; doi:10.1097/MD.0000000000038699)
Supplement: Supplementary file 15 [file medi-103-e38699-s015.doc]

# Appendix 15

**The diagram of core signaling pathway**

**Figure S1. hsa04010:MAPK signaling pathway in diabetic complications.**

**
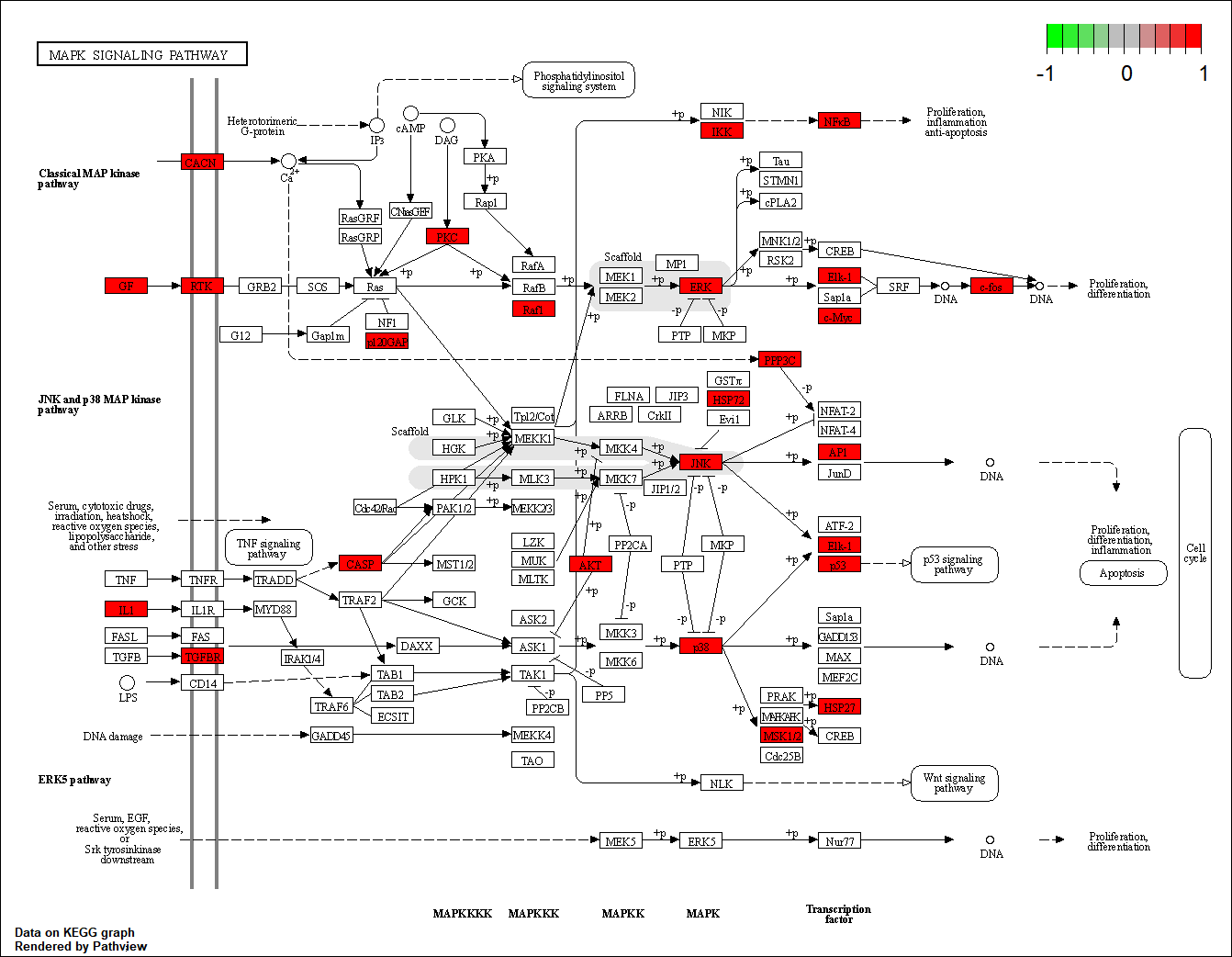
**
