# Supplementary figures and images for: Mechanism of drug-pairs Astragalus Mongholicus–Largehead Atractylodes on treating knee osteoarthritis investigated by GEO gene chip with network pharmacology and molecular docking
Source: Medicine (Baltimore). 2024 Jul 5;103(27):e38699. doi: 10.1097/MD.0000000000038699 (PMC11224889; doi:10.1097/MD.0000000000038699)

# Appendix 16

**Figure S2. hsa04151:PI3K-Akt signaling pathway.**

**
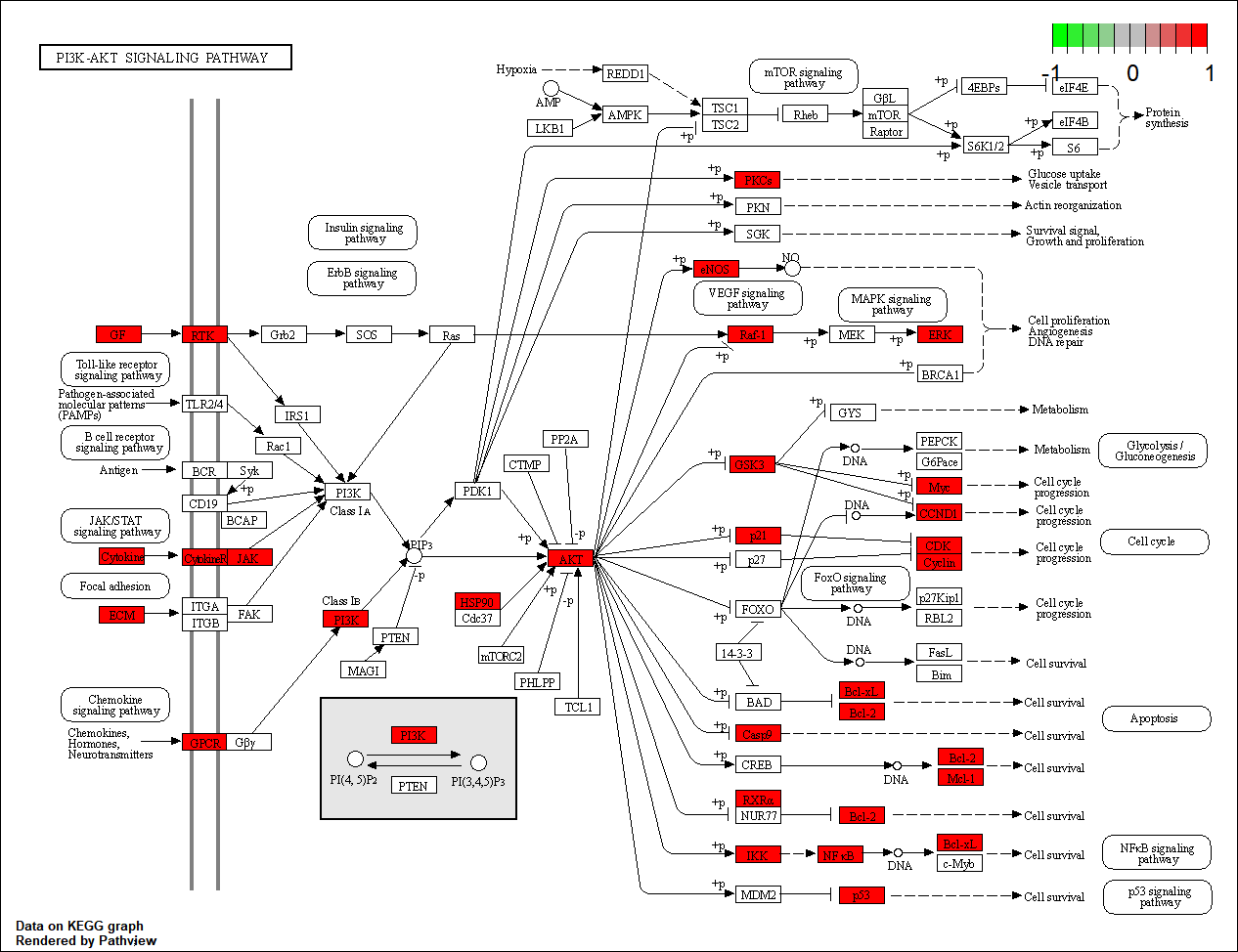
**

Supplement: Supplementary file 16 [file medi-103-e38699-s016.doc]

# Appendix 17

**Figure S3. hsa04020:Calcium signaling pathway.**

**
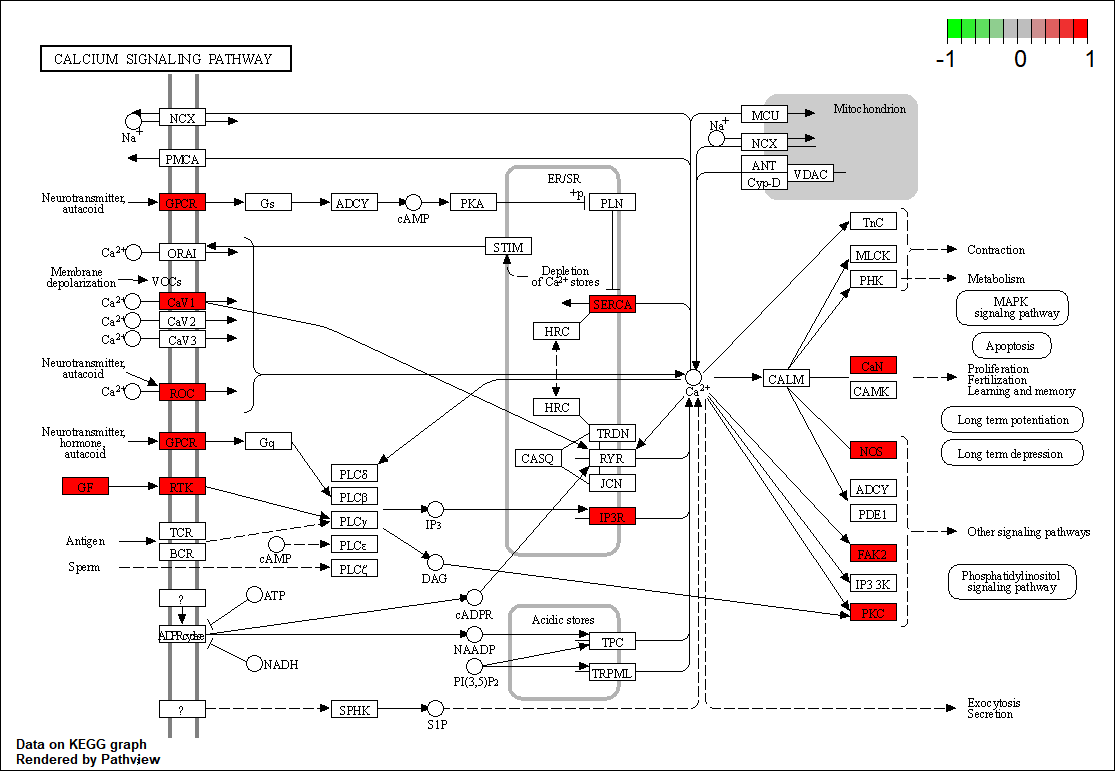
**

Supplement: Supplementary file 17 [file medi-103-e38699-s017.doc]

# Appendix 18

**Figure S4. hsa04933:AGE-RAGE signaling pathway.**

**
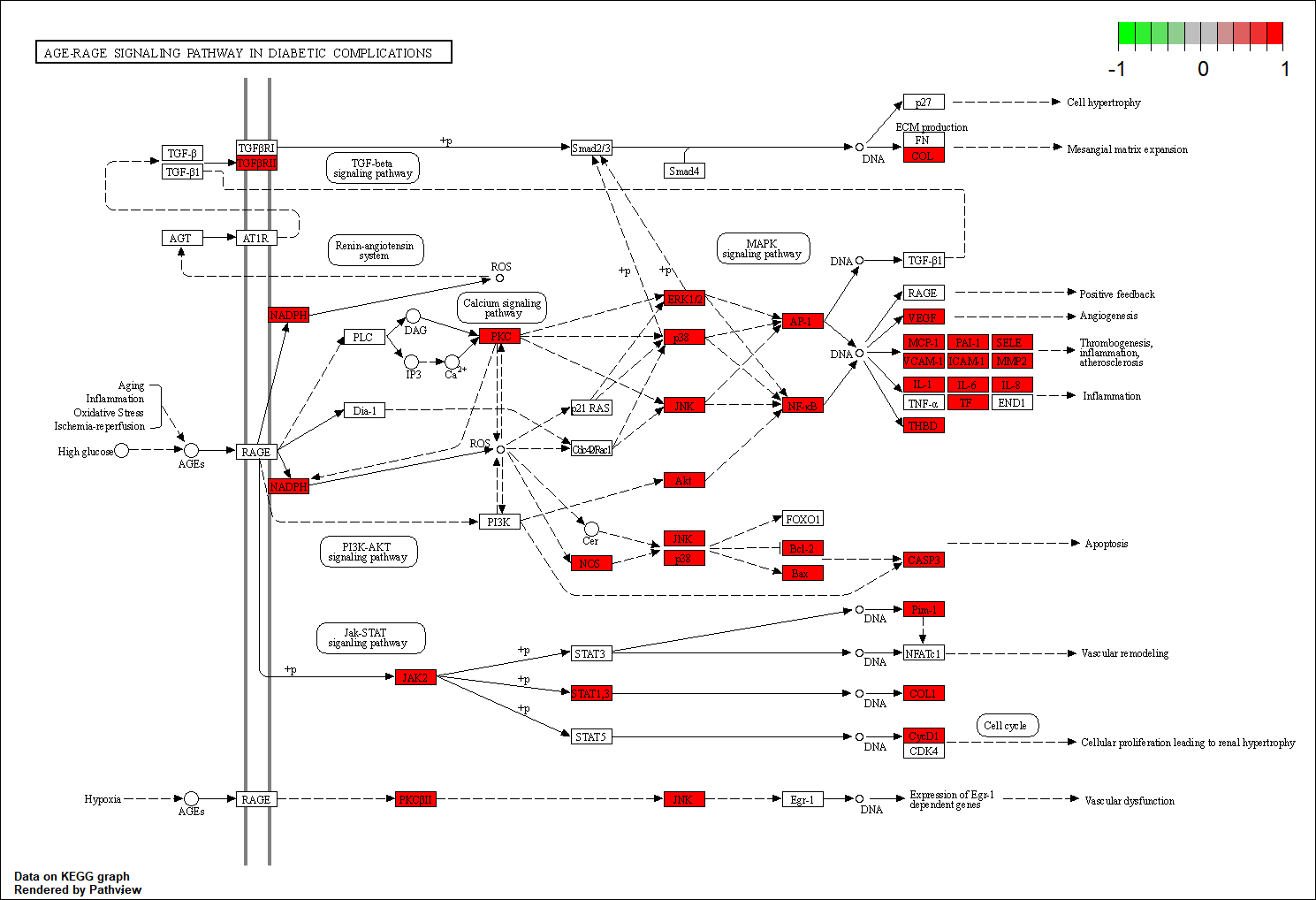
**

Supplement: Supplementary file 18 [file medi-103-e38699-s018.doc]

# Appendix 19

**Figure S5. hsa04668:TNF signaling pathway.**

**
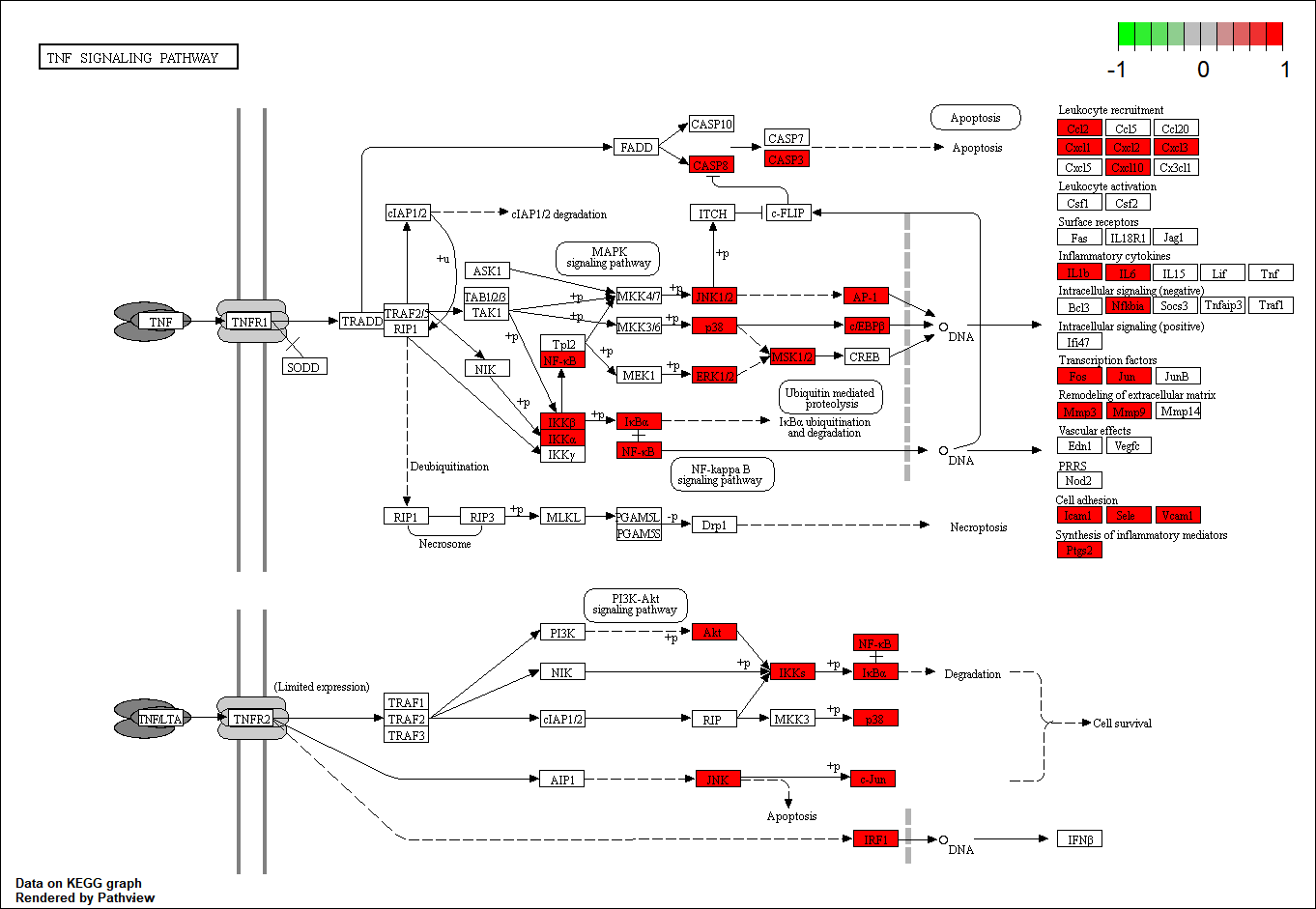
**

Supplement: Supplementary file 19 [file medi-103-e38699-s019.doc]

# Appendix 20

**Figure S6. hsa04657:IL-17 signaling pathway.**

**
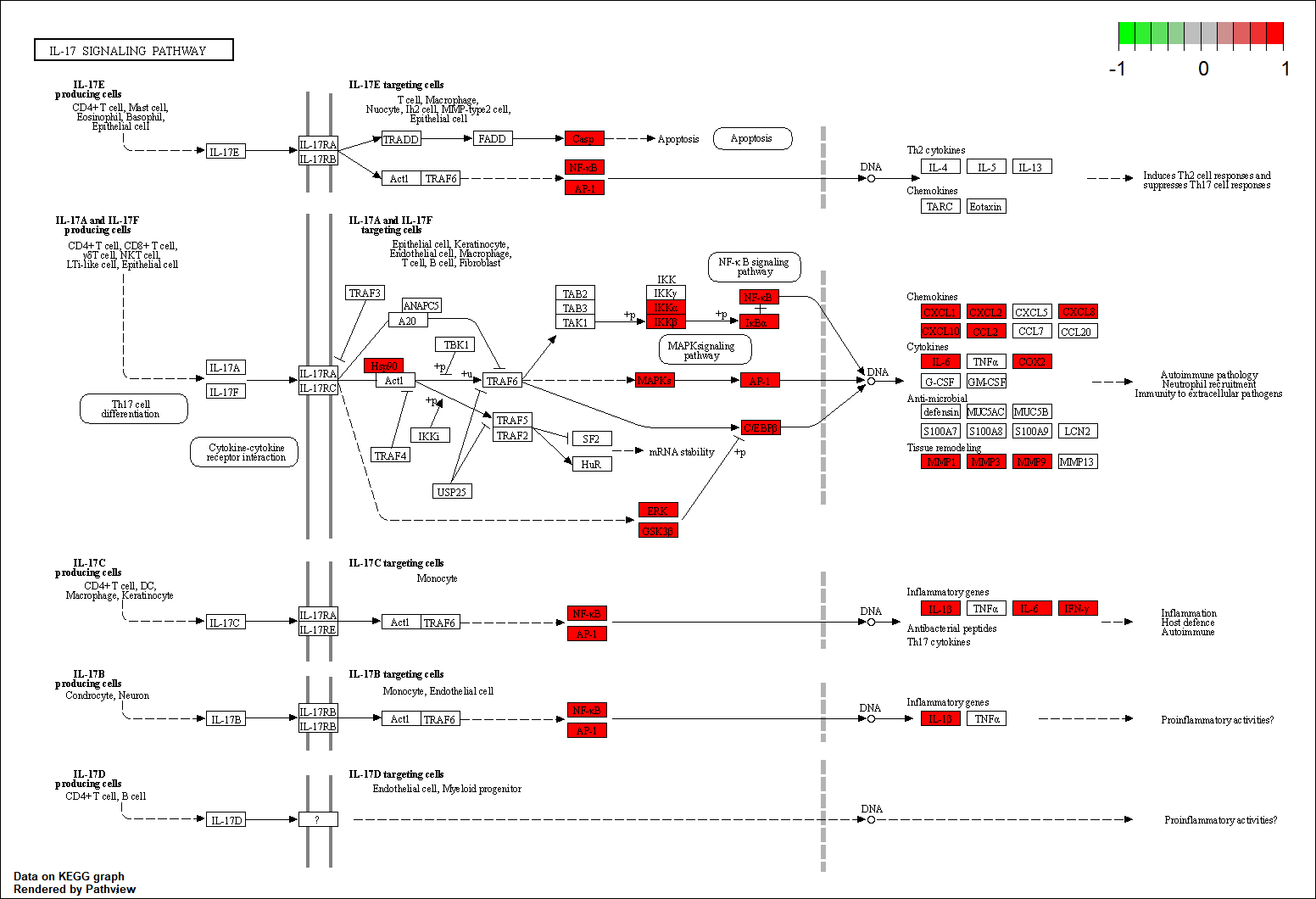
**

Supplement: Supplementary file 20 [file medi-103-e38699-s020.doc]

# Appendix 21

**Figure S7. hsa04066:HIF-1 signaling pathway.**

**
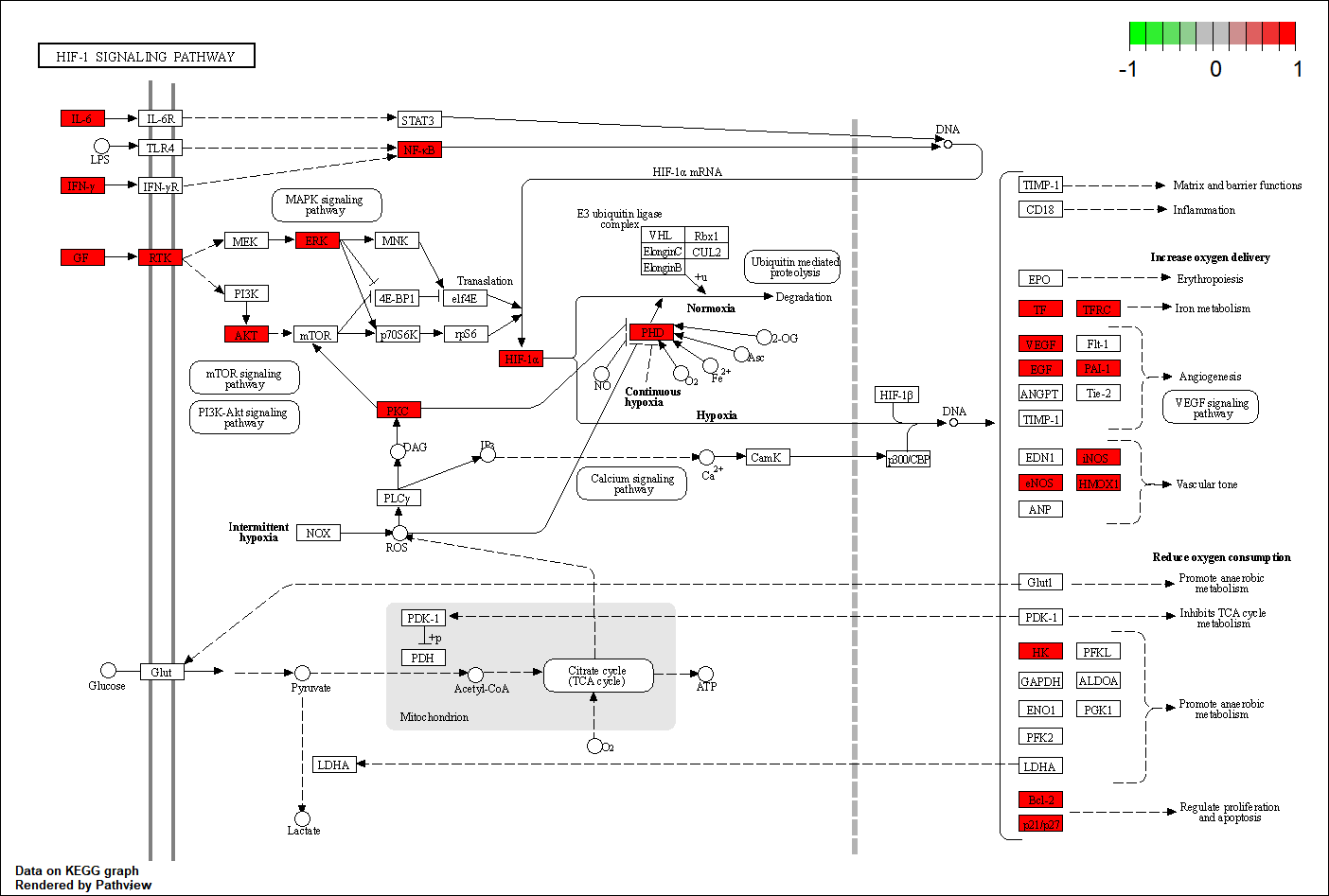
**

Supplement: Supplementary file 21 [file medi-103-e38699-s021.doc]
